# Supplementary material for: Proteomic Analysis of HCC-1954 and MCF-7 Cell Lines Highlights Crosstalk between αv and β1 Integrins, E-Cadherin and HER-2
Source: Int J Mol Sci. 2022 Sep 5;23(17):10194. doi: 10.3390/ijms231710194 (PMC9456615; doi:10.3390/ijms231710194)
Supplement: Supplementary file 1 [file ijms-23-10194-s001.zip › Supplementary File 1_02082022_Pereira D de A et al.pdf]

**Proteomic Analysis of HCC-1954 and MCF-7 Cell Lines highlight crosstalk between αv and β1 integrins, E-cadherin and HER-2**

Pereira D. de A., Sandim V., Fernandes T. F. B., Almeida V. H., Rocha M. R., do Amaral R. J. F. C., Rossi M. I. D., Kalume D. E., Zingali R. B.

**Additional File 2 – Supplementary Data**

**Figures S1, S2; S4-S9**

**Tables S1; S3-S5**

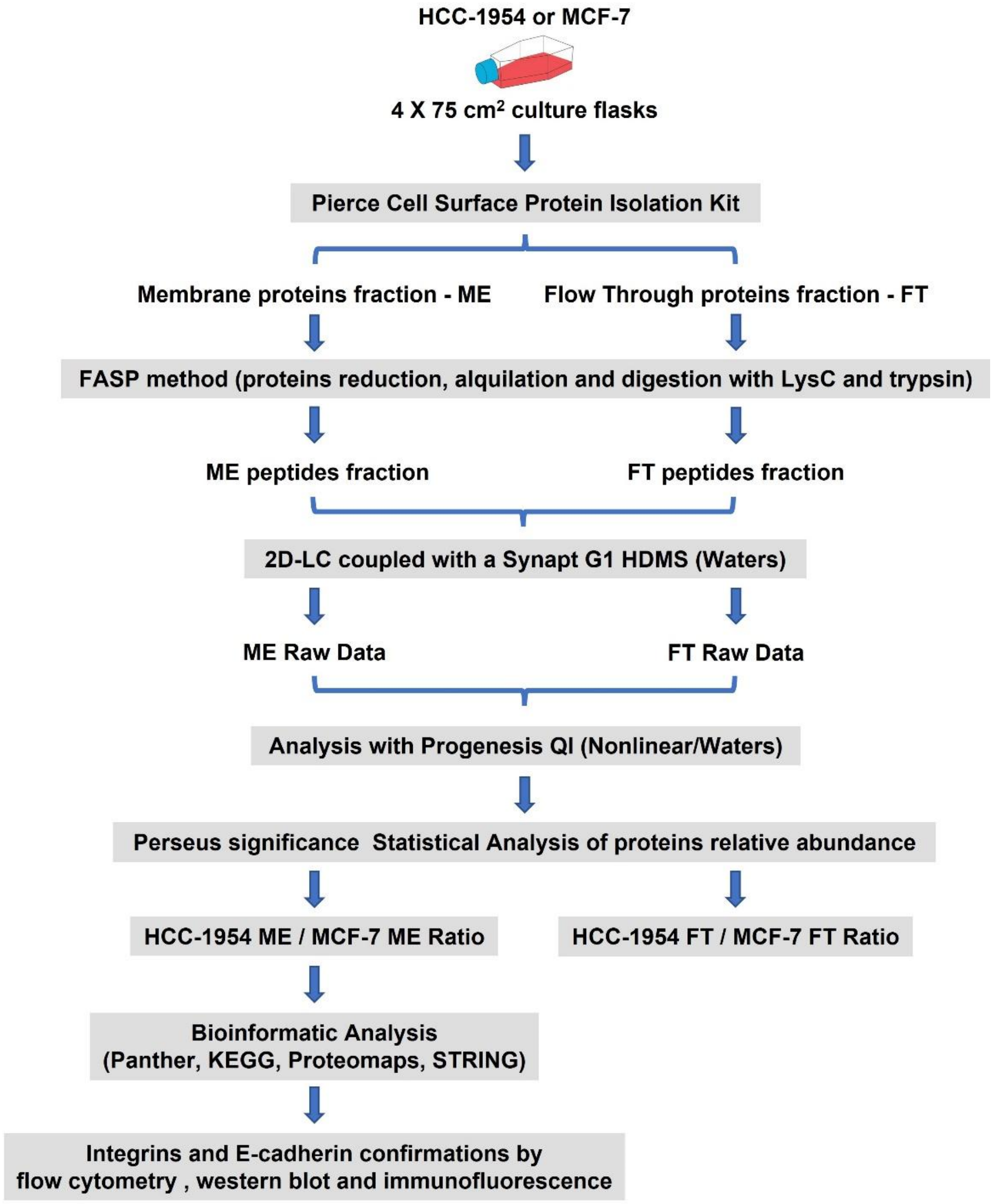


**Supplementary Figure S1 –** Schematic diagram showing the workflow used in this study. The Biotinylate Membrane (ME) and Flow Through (FT) fractions of HCC-1954 or MCF-7 cell lines were obtained using the Pierce cell surface protein isolation kit. The FASP methodology was used for proteins reduction, alquilation and digestion. 2D-LC label free mass spectrometry was used to identify and quantify proteins from both ME and FT fractions. ME and FT raw data were analysed with progenesis QI software. Perseus was used to generate volcano plots for analysis of statistically significant proteins abundance of the HCC-1954/MCF-7 ME or HCC-1954/MCF-7 FT ratios. Proteins of ME fractions, with relative fold change FC>2, were used for bioinformatic analysis. Flow cytometry, western blot and immunofluorescence confirmed the higher abundance of integrins and E-cadherin in membrane of HCC-1954 cell line.

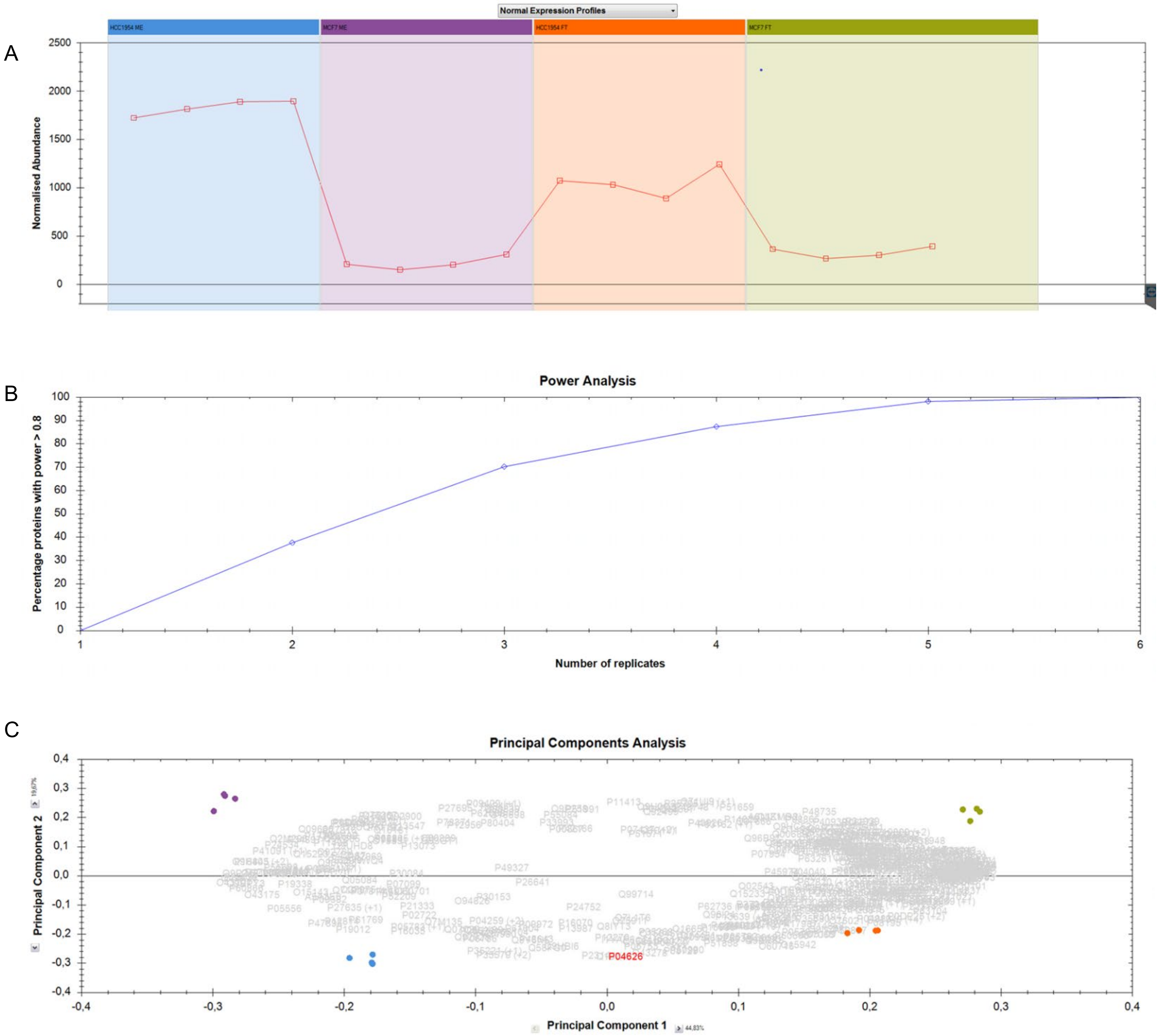


**Supplementary Figure S2 –** Data Quality. (A) Expression profile showing the normalized abundance of the HER-2 protein in the four experimental replicates of HCC-1954 ME (blue), HCC-1954 FT (purple), MCF-7 ME (orange) and MCF-7 FT (green) cell lines sample fractions. (B) Power analysis showing that approximately 90% of our data had a power ≥ 0.8 with the four replicates analysed. (C) Principal component analysis (PCA) showing the 453 proteins identified and quantified by the Progenesis QI Program for ANOVA of p ≤0.05 distribution between the four experimental replicates of HCC-1954 ME (blue), HCC-1954 FT (purple), MCF-7 ME (orange) and MCF-7 FT (green) fractions. In red on PCA are the Uniprot protein ID for HER-2 (P04626) showing its distribution between samples.

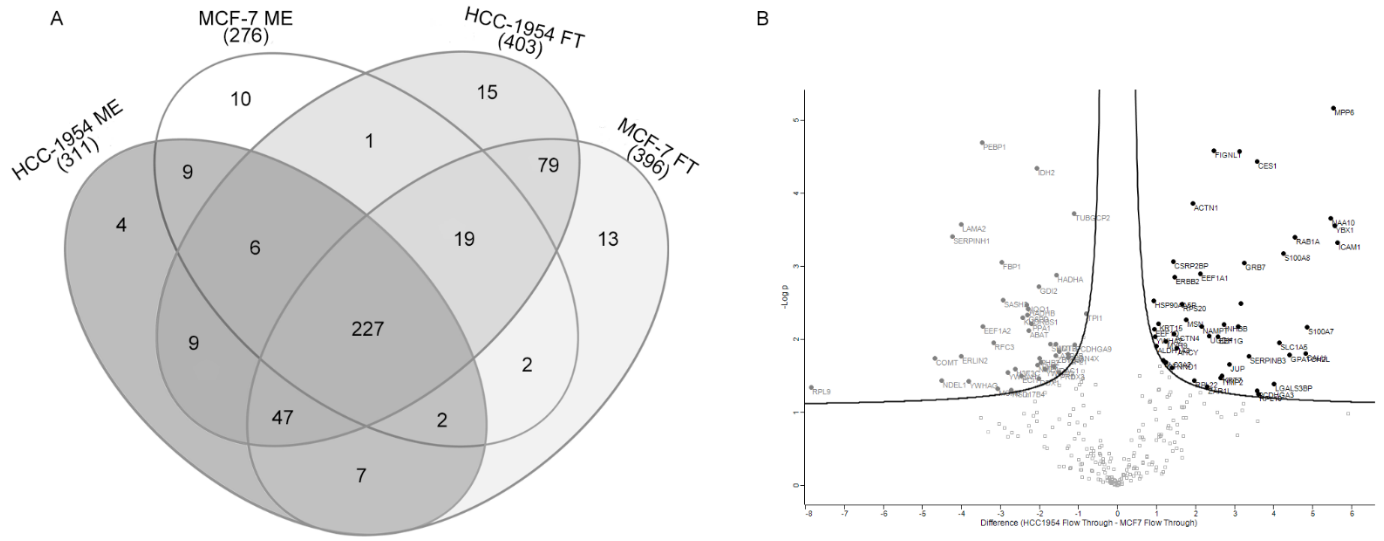


**Supplementary Figure S4 –Proteomic data Analysis** (A) Interact Venn Diagram of the 450 proteins identified and quantified with 95% confidence (ANOVA p <0.05) in the Progenesis QI analysis of our label free proteomic data of HCC-1954 and MCF-7 ME and FT fractions. (E) Volcano plots of the total proteins from HCC-1954 and MCF-7 cells in FT fraction. Proteins with increased fold change are indicated by black circles whereas the gray circles denote those proteins presenting a decreased fold change.

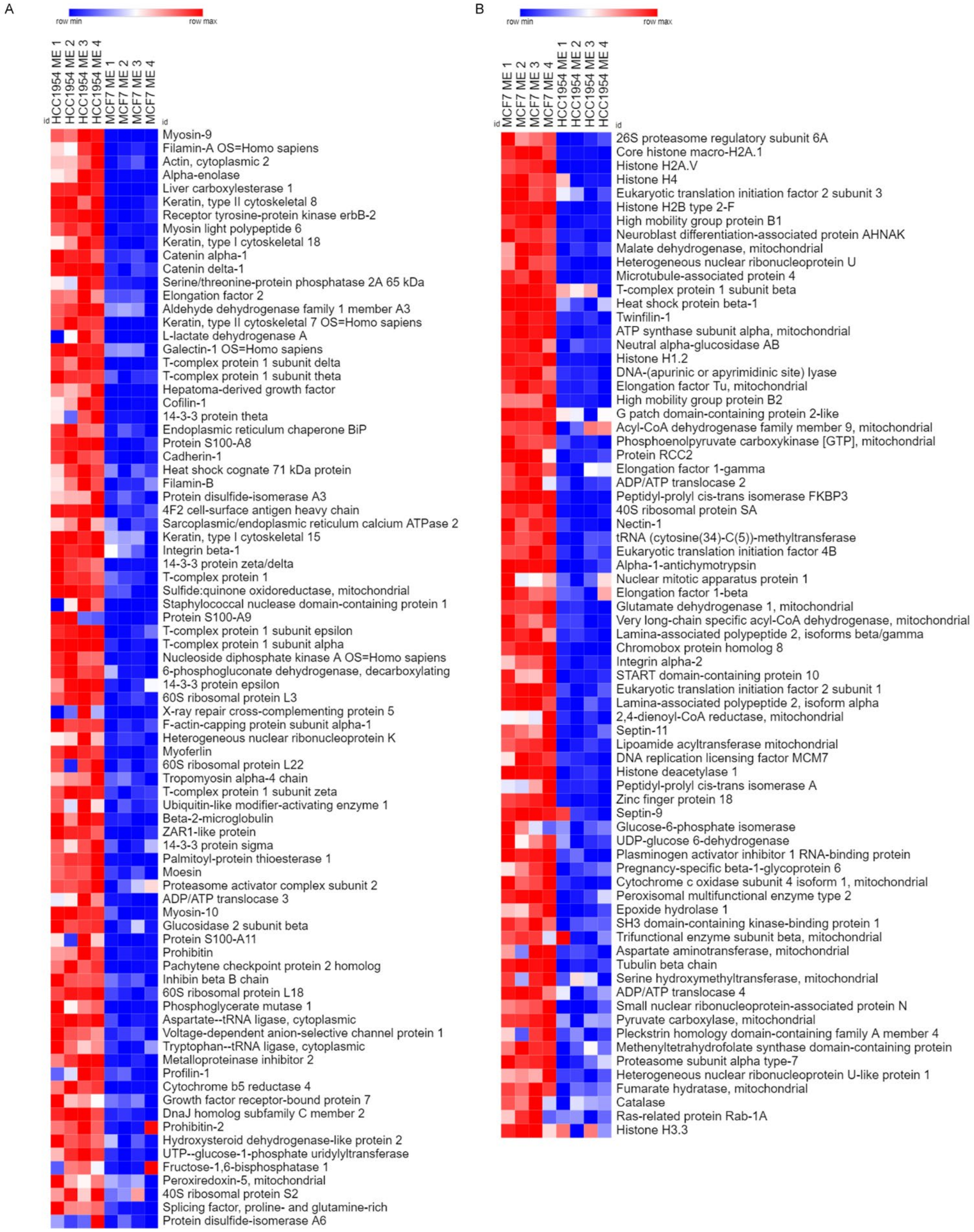


**Supplementary Figure S5 –** Heat map analysis membrane fraction. (**A**) 82 proteins increased in HCC-1954 ME and (**B**) 73 proteins increased in MCF-7 ME.

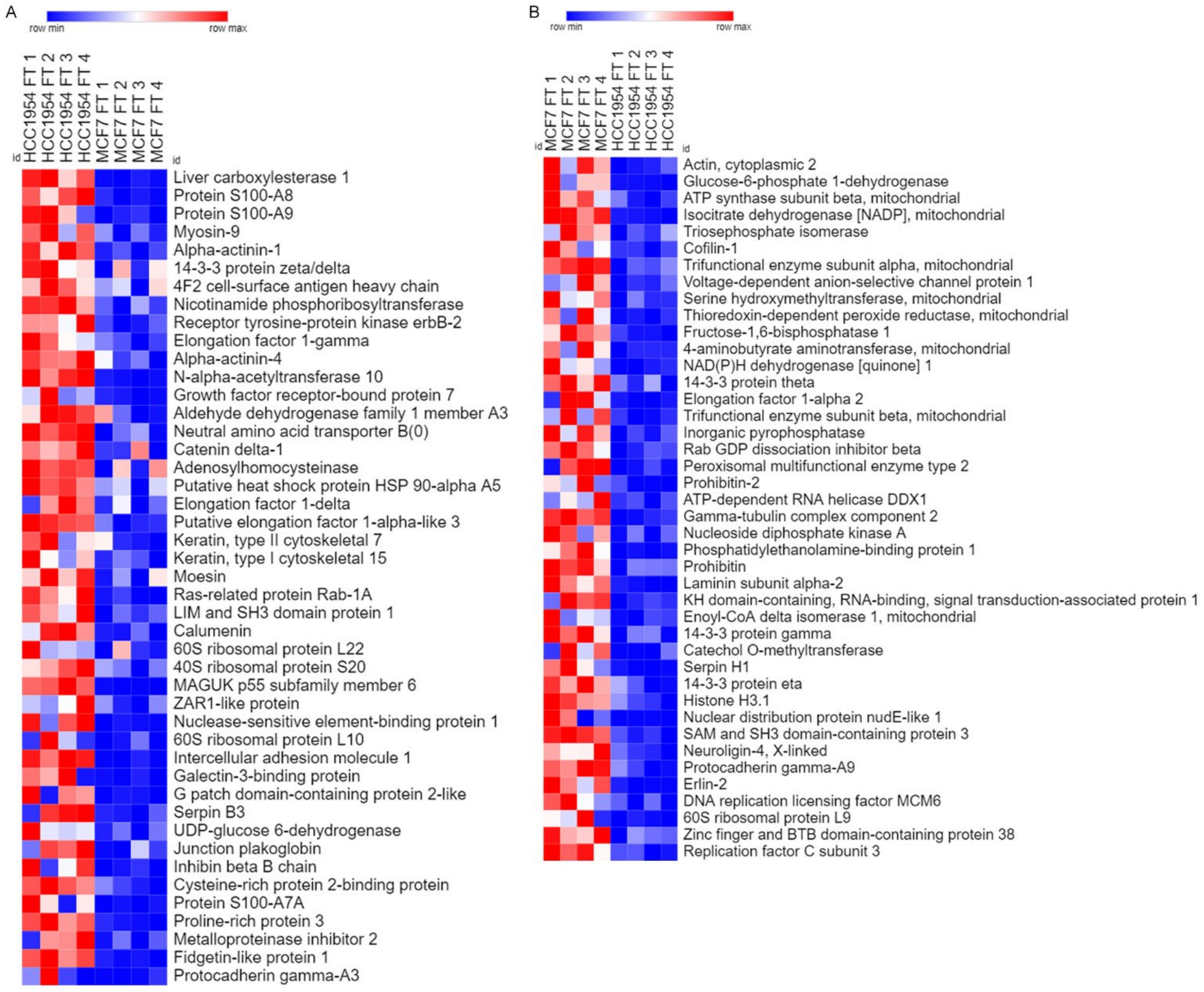


**Supplementary Figure S6 –** Heat map analysis flow through fraction. (**A**) 46 proteins increased in HCC-1954 FT and (**B**) 42 proteins increased in MCF-7 FT.

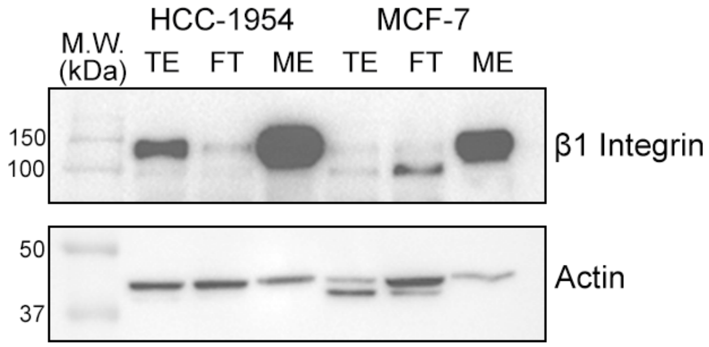


**Supplementary Figure S7 – Western blot analysis of β1-integrin expression level in HCC-1954 and MCF-7 cell line fractions (TE total extract; FT flow-through; ME membrane enriched)**. The ME fraction of both HCC-1954 and MCF-7 cell lines showed a higher signal intensity of β1-integrin (~140-150 kDa), a known cell membrane receptor for extracellular matrix (ECM) proteins compared to the TE and FT fractions. This demonstrates the efficiency of the method for membrane protein enrichment. Actin (42 kDa, clone C4 MAB 1501, millipore) was used as loading control. Raw data are presented in Supplementary Figure S9D. TE extracts were obtained with RIPA buffer. ME and FT extracts were obtained with cell membrane isolation kit (Pierce®, cat. number 89881).

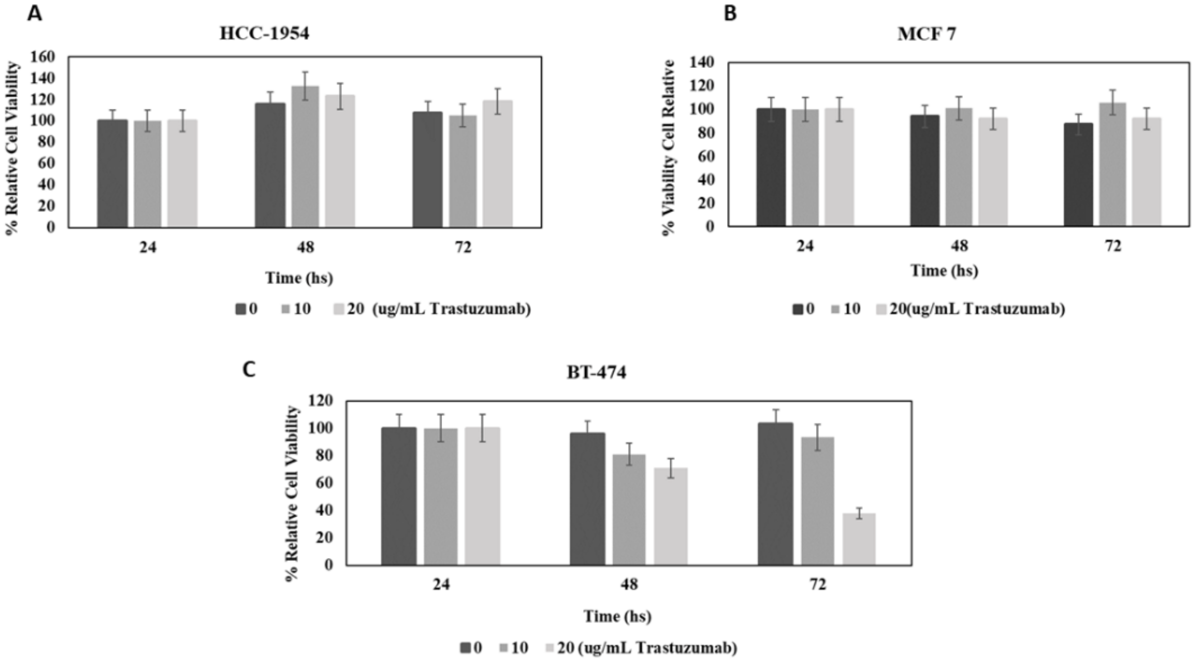


**Supplementary Figure S8 -** Viability assay of cells in trastuzumab presence: (A) HCC-1954, (B) MCF-7 and (C) -474 cell lines. Viability assay was done with MTT (bromide de [3-(4,5-dimetiltiazol-2yl)-2,5-difenil tetrazolium]). HCC-1954 and MCF-7 were not affected by trastuzumab treatment. However, BT-474 lose cell viability in 72 h of treatment with 20 µg/mL of trastuzumab, so this condition was selected for treatment.

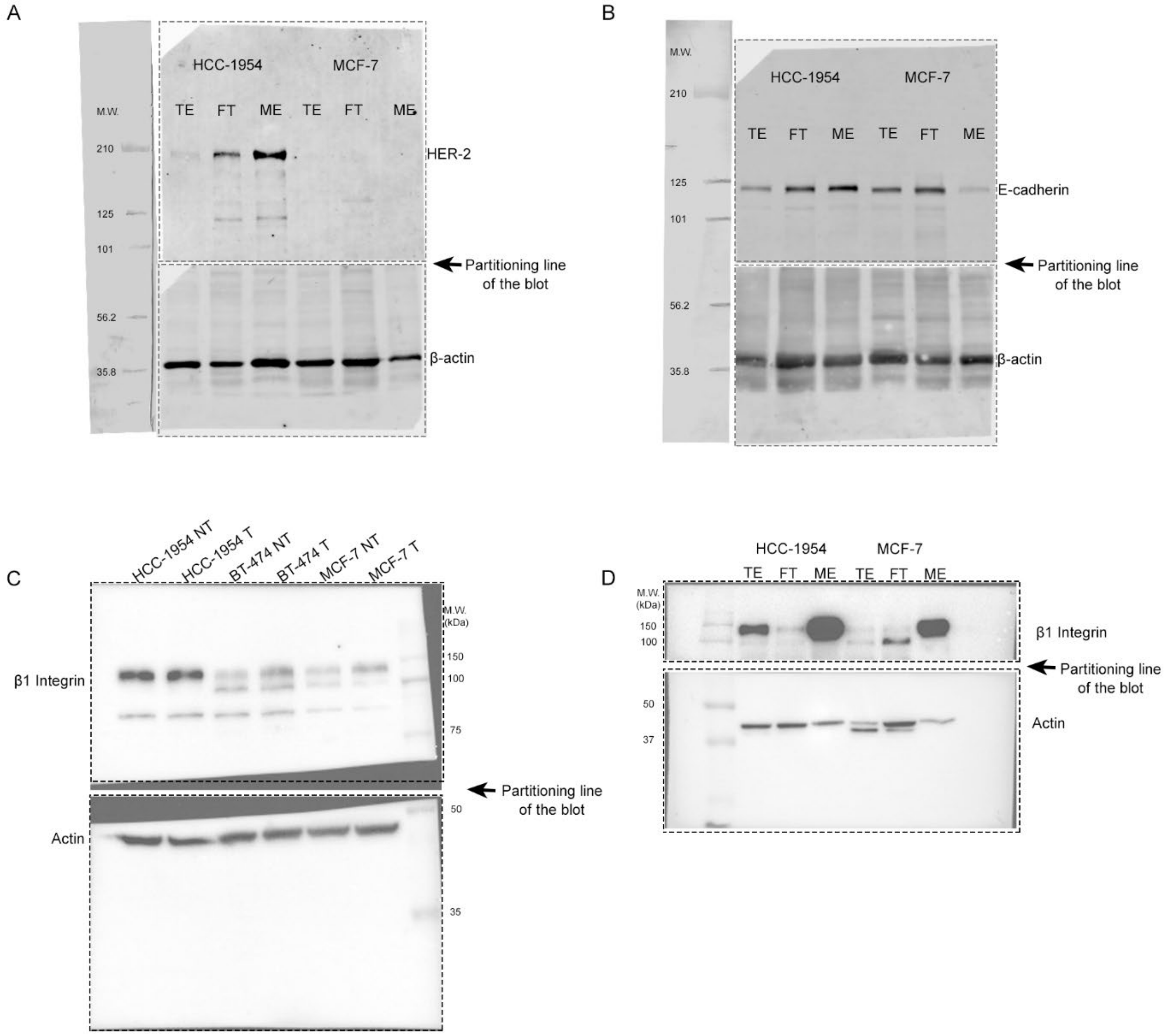


**Supplementary Figure S9** – Raw data western blot used for Figure 1**(A),** Figure 4 **(B),** Figure 5 **(C)** and Supplementary Figure S**7.** A dotted line between top and the bottom parts of the membranes indicates the line of incision that divided them into two parts for separately probed **(A)** anti-rabbit HER-2 (185 kDa, ab-2428, Abcam) and anti-mouse β-actin (42 kDa, A2228 Sigma) antibodies and **(B)** anti-mouse E-cadherin (120 kDa, 610182, BD) and anti-mouse β-actin (42 kDa, A2228 Sigma) in total extract (TE), flow through (FT) and membrane enriched (ME) fractions of HCC-1954 and MCF-7 cell lines. **(C)** anti-rabbit β1 integrin (~140-150 kDa, ab52971, Abcam) and anti-mouse actin (42 kDa, clone C4 MAB 1501, millipore) in HCC-1954, MCF-7 and BT-474 cell lines treated (T) or not (NT) with trastuzumab. **(D)** anti-rabbit β1 integrin and anti-mouse actin in the total extract (TE), flow through (FT) and membrane enriched (ME) fractions of HCC-1954 and MCF-7 cell lines. TE extracts were obtained with RIPA buffer. ME and FT extracts were obtained with cell membrane isolation kit (Pierce®, cat. number 89881).

**Supplementary Table S1 –** Characteristics of Breast Cancer Cell Lines

| **Cell line** | **Classification** | **Receptor Expression** | **Tumor source** | **Aggressivity** | **Invasivity** |
|---|---|---|---|---|---|
| HCC-1954 | HER2+ | HER2 overexpressed | Primary tumor | Highly | Highly |
| MCF-7 | Luminal A | ER and PR expressed | Metastatic site | Low | Low |
| BT-474 | HER2+ | HER2 overexpressed | Primary tumor | Highly | Highly |

**Supplementary Table S3** - Proteins only Identified in ME and/or FT fractions of HCC1954 and MCF7 cell lines.

| Fractions | HCC1954 | MCF7 |
|---|---|---|
| ME | Inactive caspase-12 (CASP12); Actin, aortic smooth muscle (ACTA2); CD166 antigen (ALCAM); 40S ribosomal protein S27 (RPS27) | Histone H1.5 (H1-5);<br>Protein S100-A13 (S100A13),<br>Kelch-like protein 22 (KLHL22);<br>Regulator of nonsense transcripts 1 (UPF1);<br>V-type proton ATPase subunit B (ATP6V1B1), kidney isoform;<br>Flap endonuclease 1 (FEN1);<br>Septin-7 (SEPTIN7);<br>Sex comb on midleg-like protein 2 (SCML2);<br>Barrier-to-autointegration factor (BANF1);<br>Testicular haploid expressed gene protein-like (THEGL); |
| FT | DnaJ homolog subfamily B member 8 (DNAJB8);<br>Baculoviral IAP repeat-containing protein 2 (BIRC2);<br>Transcription factor SOX-6 (SOX6);<br>Transcription factor AP-2-delta (TFAP2D);<br>Annexin A3 (ANXA3);<br>Double-stranded RNA-specific adenosine deaminase (ADAR);<br>Golgi reassembly-stacking protein 2 (GORASP2);<br>Tumor-associated calcium signal transducer 2 (TACSTD2);<br>F-box only protein 39 (FBXO39);<br>Regulating synaptic membrane exocytosis protein 2 (RIMS2);<br>Histone-lysine N-methyltransferase KMT5B (KMT5B);<br>Endoplasmic reticulum resident protein 44 (ERP44);<br>Vasodilator-stimulated phosphoprotein (VASP);<br>Ornithine aminotransferase, mitochondrial (OAT);<br>Tumour necrosis factor ligand superfamily member 9 (TNFSF9); | Very long-chain acyl-CoA synthetase (SLC27A2);<br>Succinate-semialdehyde dehydrogenase, mitochondrial (ALDH5A1);<br>Acylpyruvase FAHD1 mitochondrial (FAHD1);<br>Type 1 phosphatidylinositol 4,5-bisphosphate 4-phosphatase (PIP4P1);<br>Lon protease homolog, mitochondrial (LONP1);<br>Transcription elongation factor A protein 1 (TCEA1);<br>Dynein light chain 1 (DYNLL1), cytoplasmic;<br>3-ketoacyl-CoA thiolase, peroxisomal (ACAA1);<br>Nucleosome assembly protein 1-like 1 (NAP1L1);<br>40S ribosomal protein S4 (RPS4X), X isoform;<br>Poly(U)-binding-splicing factor PUF60 (PUF60)<br>Splicing factor 3B subunit 1 (SF3B1);<br>Adenine phosphoribosyltransferase (APRT); |
| ME/FT | Elongation factor 1-alpha 1 (EEF1A1); Reticulocalbin-1 (RCN1); Integrin alpha-V (ITGAV); CD59 glycoprotein (CD59); Nucleoprotein TPR (TPR); Protein-glutamine gamma-glutamyltransferase 2 (TGM2); Putative Ras-related protein Rab-1C (RAB1C); NKAP-like protein (NKAPL); Cytochrome P450 4F11 (CYP4F11) | Ras-related protein Rap-1b-like protein (RAP1B);<br>Ubiquitin carboxyl-terminal hydrolase 46 (USP46) |

**Supplementary Table S4** – Proteins increased in fold change and only detected in HCC-1954, classified in top pathways of HCC-1954. Proteins that belong to the pathway are indicated in green and red squares show the proteins not included in that pathway. *(NA – Not Applied)*

| Protein | HCC-1954 | Pathways | | | | | | |
|---|---|---|---|---|---|---|---|---|
| | (Fold Change) | Tight junction | Regulation of actin cytoskeleton | Leukocyte transendothelial migration | Focal adhesion | Adherens junction | Proteoglycans in cancer | Pathways in cancer |
| ACTG1; actin gamma 1 | 2.50 (ME) | | | | | | | |
| ACTN1; actinin alpha 1 | 3.80 (ME) | | | | | | | |
| ACTN4; actinin alpha 4 | 2.73 (ME) | | | | | | | |
| CDH1; cadherin 1 | 13.39 (ME) | | | | | | | |
| CFL1; cofilin 1 | 9.94 (ME) | | | | | | | |
| CTNNA1; catenin alpha 1 | 2.47 (ME) | | | | | | | |
| CTNND1; catenin delta 1 | 3.17 (ME) | | | | | | | |
| ERBB2; erb-b2 receptor tyrosine kinase 2 | 10.27 (ME) | | | | | | | |
| FLNA; filamin A | 1.72 (ME) | | | | | | | |
| FLNB; filamin B | 3.56 (ME) | | | | | | | |
| ICAM1; CD54 intercellular adhesion molecule 1 | 49.5 (FT) | | | | | | | |
| ITGAV; integrin subunit alpha V | NA | | | | | | | |
| ITGB1; integrin subunit beta 1 | 2.24 (ME) | | | | | | | |
| JUP; junction plakoglobin | 7.26 (ME) | | | | | | | |
| MSN; moesin | 2.66 (ME) | | | | | | | |
| MYH10; myosin heavy chain 10 | 6.86 (ME) | | | | | | | |
| MYH9; myosin heavy chain 9 | 13.95 (ME) | | | | | | | |
| MYL6; myosin light chain 6 | 7.90 ME) | | | | | | | |
| PFN1; profilin 1 | 1.94 (ME) | | | | | | | |
| PPP2R1A; protein phosphatase 2 scaffold subunit A | NA | | | | | | | |
| TPR; translocated promoter region, nuclear basket protein | NA | | | | | | | |
| VASP; vasodilator stimulated phosphoprotein | NA | | | | | | | |

**Supplementary Table S5** – Proteins increased in fold change and only detected in MCF-7, classified in top pathways of HCC-1954. In (Green) proteins that belong to the pathway and in (Red) not included in that pathway.

| Protein | MCF-7 | Pathways | | | | | | |
|---|---|---|---|---|---|---|---|---|
| | (Fold Change) | Tight junction | Regulation of actin cytoskeleton | Leukocyte transendothelial migration | Focal adhesion | Adherens junction | Proteoglycans in cancer | Pathways in cancer |
| ACTG1; actin gamma 1 | 2.98 (FT) | Green | Green | Green | Green | Green | Green | Red |
| CFL1; cofilin 1 | 2.40 (FT) | Red | Green | Red | Red | Red | Red | Red |
| EIF4B; eukaryotic translation initiation factor 4B | 3.71 (ME) | Red | Red | Red | Red | Red | Green | Red |
| FH; fumarate hydratase | 11.21 (FT) | Red | Red | Red | Red | Red | Red | Green |
| HDAC1; histone deacetylase 1 | 6.58 (ME) | Red | Red | Red | Red | Red | Red | Green |
| ITGA2; integrin subunit alpha 2 | 17.27 (ME) | Red | Green | Red | Green | Red | Green | Green |
| LAMA2; laminin subunit alpha 2 | 18.63 (ME) | Red | Red | Red | Green | Red | Red | Green |
| NAD(P)H quinone dehydrogenase 1 | 5.0 (FT) | Red | Red | Red | Red | Red | Red | Green |
| NECTIN1; nectin cell adhesion molecule 1 | 7.15 (ME) | Red | Red | Red | Red | Green | Red | Red |
