# Supplementary material for: Proteomic Analysis of HCC-1954 and MCF-7 Cell Lines Highlights Crosstalk between αv and β1 Integrins, E-Cadherin and HER-2
Source: Int J Mol Sci. 2022 Sep 5;23(17):10194. doi: 10.3390/ijms231710194 (PMC9456615; doi:10.3390/ijms231710194)
Supplement: Supplementary file 1 [file ijms-23-10194-s001.zip › Supplementary File 2_Pereira D de A et al..pdf]

Proteomic Analysis of HCC-1954 and MCF-7 Cell Lines highlight crosstalk between αv and β1 integrins, E-cadherin and HER-2

Pereira D. de A., Sandim V., Fernandes T. F. B., Almeida V. H., Rocha M. R., do Amaral R. J. F. C., Rossi M. I. D., Kalume D. E., Zingali R. B.

Additional File 2 – Supplementary Figure S3 – Mass Spectrometry Data Quality

Data quality analysis of the four samples (HCC-1954FT, HCC-1954 ME, MCF-7 FT and MCF-7 ME) showed a dynamic range of 3 logs of abundance for the FT fractions whereas a dynamic range of 4 logs of abundance was observed for the ME fractions. This difference was likely due to the membrane protein enrichment step, which could contribute to higher protein recovery in these fractions. Peptides were eluted in the 2nd and 3rd quartile of the chromatography, indicating a successful separation process. Most peptide ions were identified with one trypsin missed cleavage and had a charge of +2 and +3, a mass error <10 ppm and m/z 400-1200 ppm.

HCC1954 X MCF7 STEP1

QC Metrics reported by Progenesis QI for proteomics v2.0.5556.29015
Date: 10-Jan-2020 at 09:17
Experiment design: HCC1954 X MCF7 STEP1

This QC report contains **all metrics** for the experiment named above.

Reported metrics

0 of the 19 metrics reported have been manually flagged for attention.

- Identifications overview
- LC peak width
- Peptide ion dynamic range
- Precursor m/z
- Precursor retention time
- Mass errors
- Precursor charges
- Missed cleavages (assuming trypsin)
- Peptides per protein (overview)
- Modifications
- Missed cleavages
- Abundance dynamic range
- Mass accuracy
- Scan rates
- Proteins
- Peptides
- Peptides per protein (by condition)
- Percentage of peptide ions identified
- Protein overlap

Identifications overview

The chart below provides the number of identifications per run by category. Data are plotted for all runs in the current experiment design. The box represents the interquartile range and the whiskers the maximum and minimum.

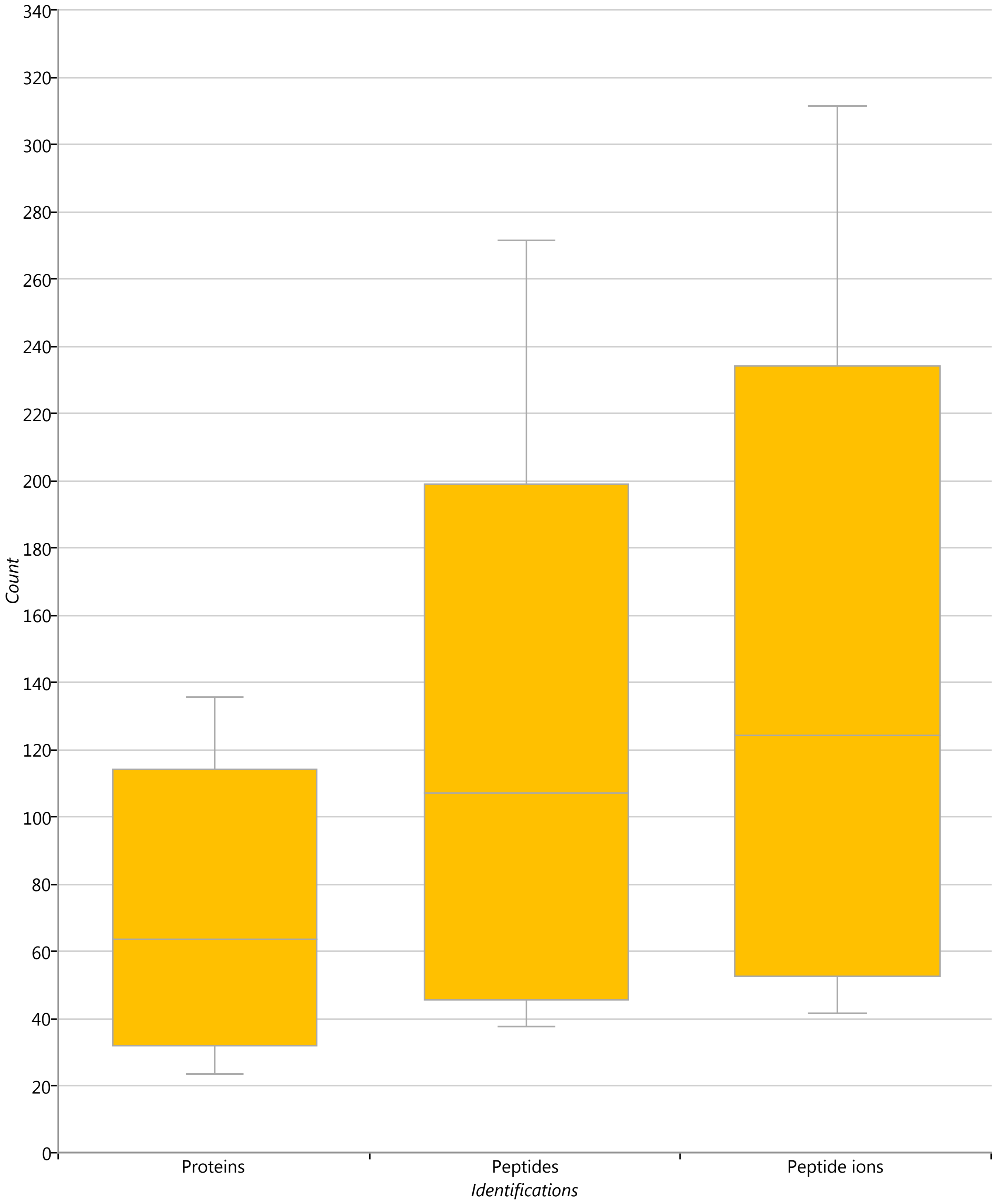

LC peak width

This chart shows the proportional distribution of peak widths in retention time, for all the detected peptide ions in the experiment. Widths are binned into categories for simple comparison. Identified peptide ions are shown in yellow.

Potential problems:

- A peak width that is different from that expected for your setup may indicate a problem with the chromatographic separation.

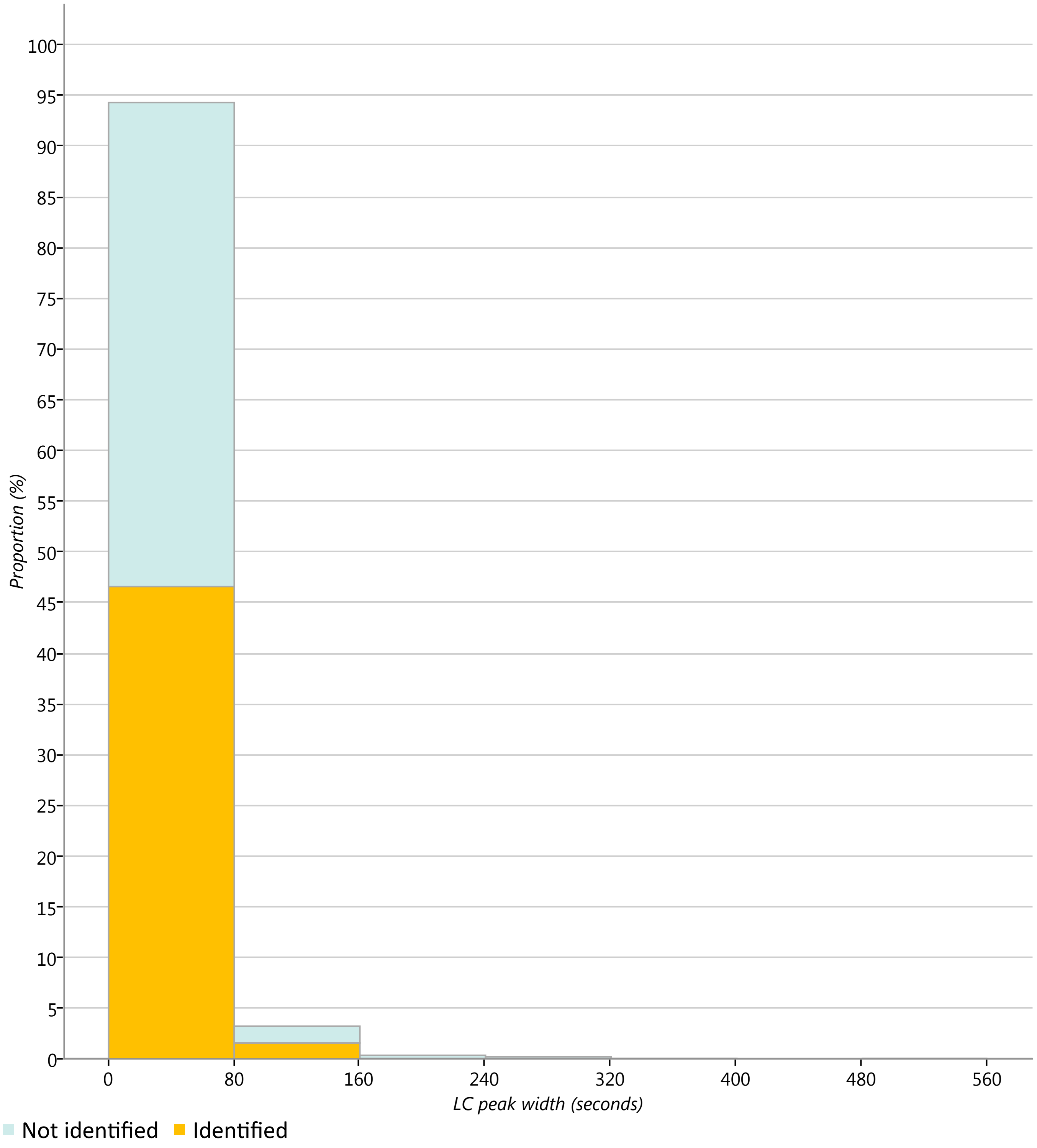

Peptide ion dynamic range

This chart shows log abundance for peptide ions, calculated using the current experiment design. Each peptide ion is assigned its abundance based on the highest mean value for any condition's runs. Data are binned and shown as a proportion. Identified peptide ions are shown in yellow.

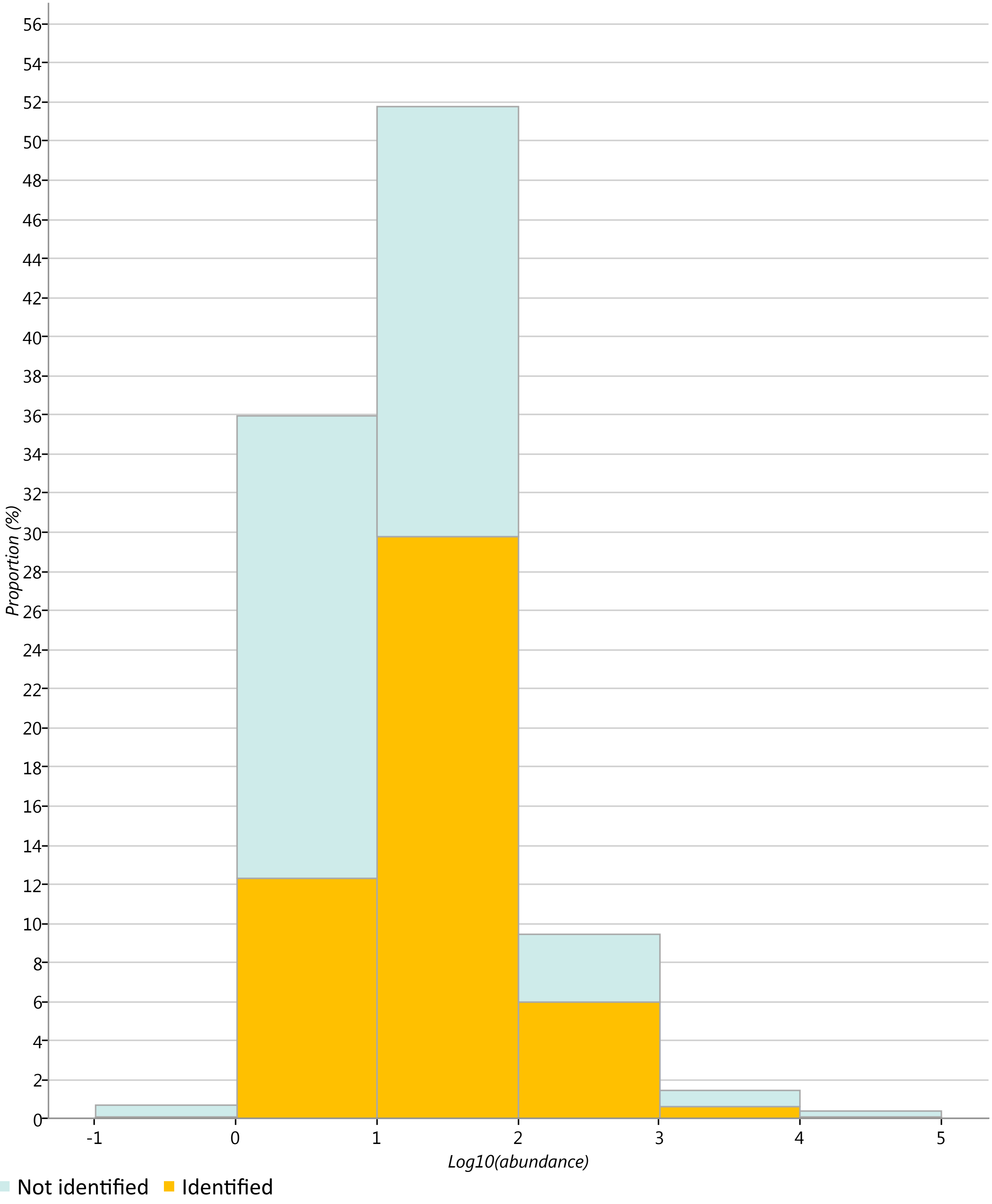

Precursor m/z

This chart shows the distribution of m/z values for all peptide ions in the experiment. Identified peptide ions are shown in yellow.

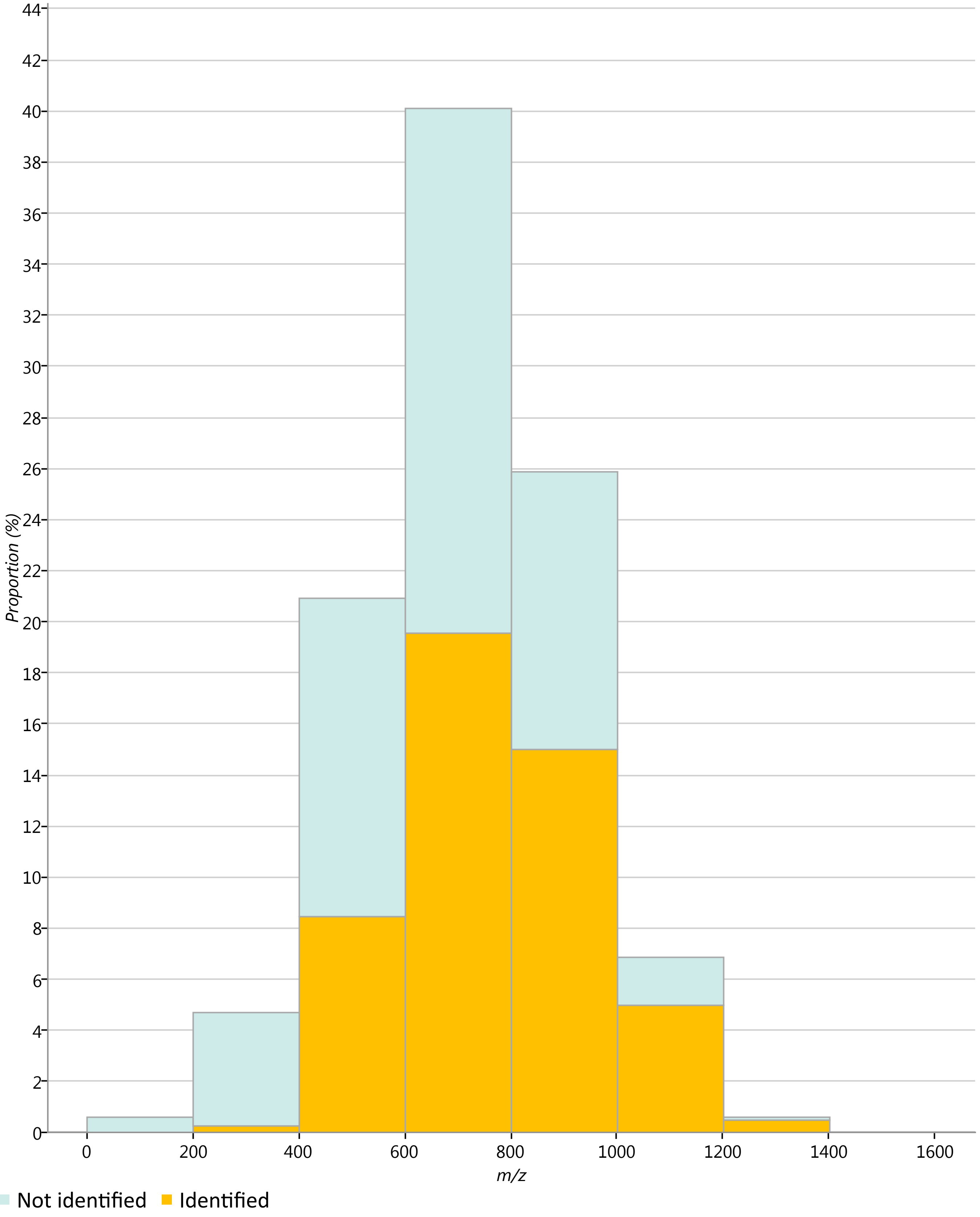

Precursor retention time

This chart shows the proportion of precursor peptide ions eluting into each quartile of the LC run. Identified peptide ions are shown in yellow.

Potential problems:

- A majority of peptide ions eluting at the start or end of the run may indicate problems with the chromatographic separation or that the setup is not ideal for a successful separation.

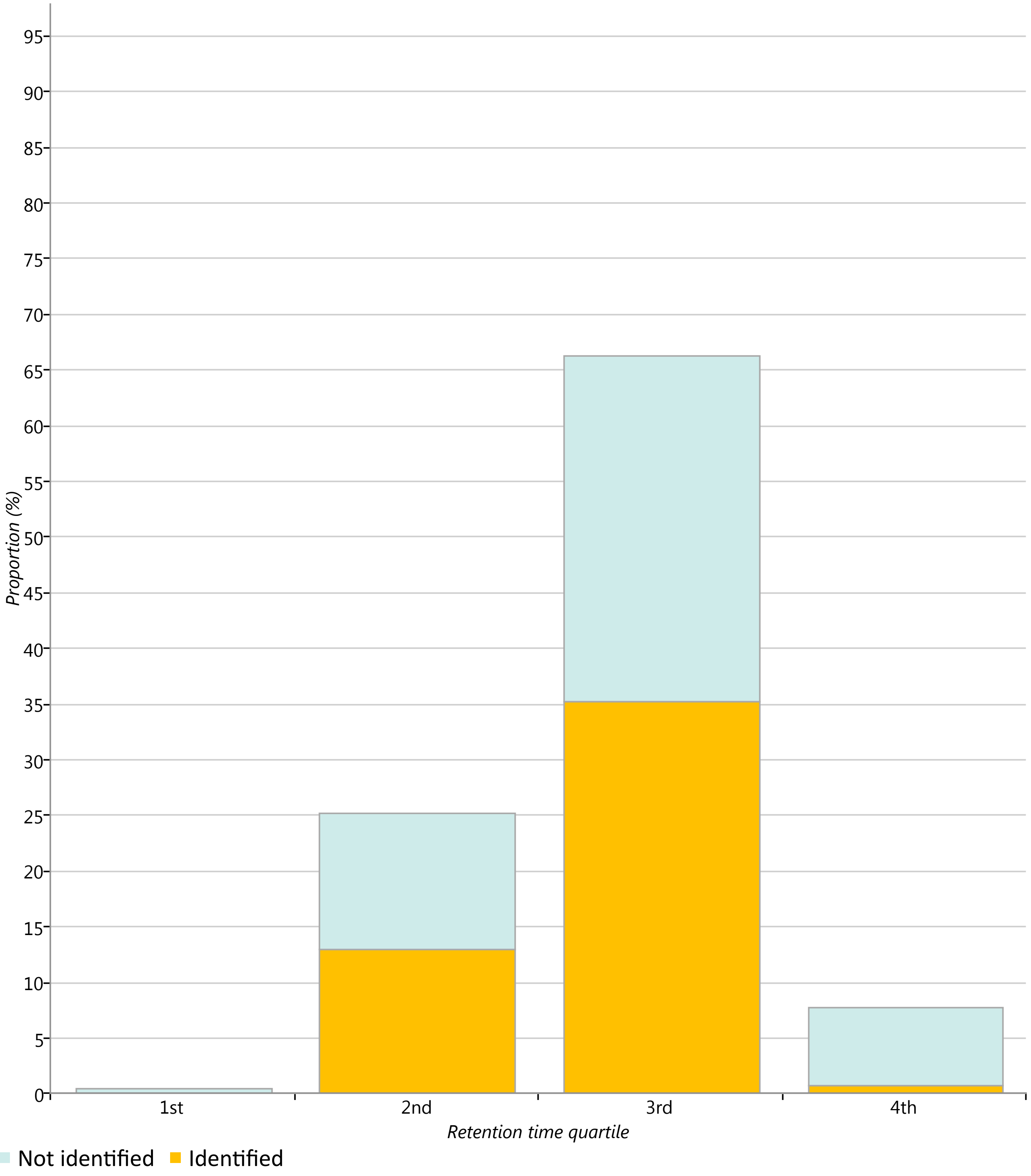

Mass errors

This chart shows the mass error calculated for identified peptide ions. The proportion of peptide ions with a given binned error value is plotted. For a more detailed plot, see the *Mass accuracy* metric.

Potential problems:

- A larger than expected spread could indicate that search parameters are too lenient.
- A bias of the peak from zero ppm could indicate instrument bias.

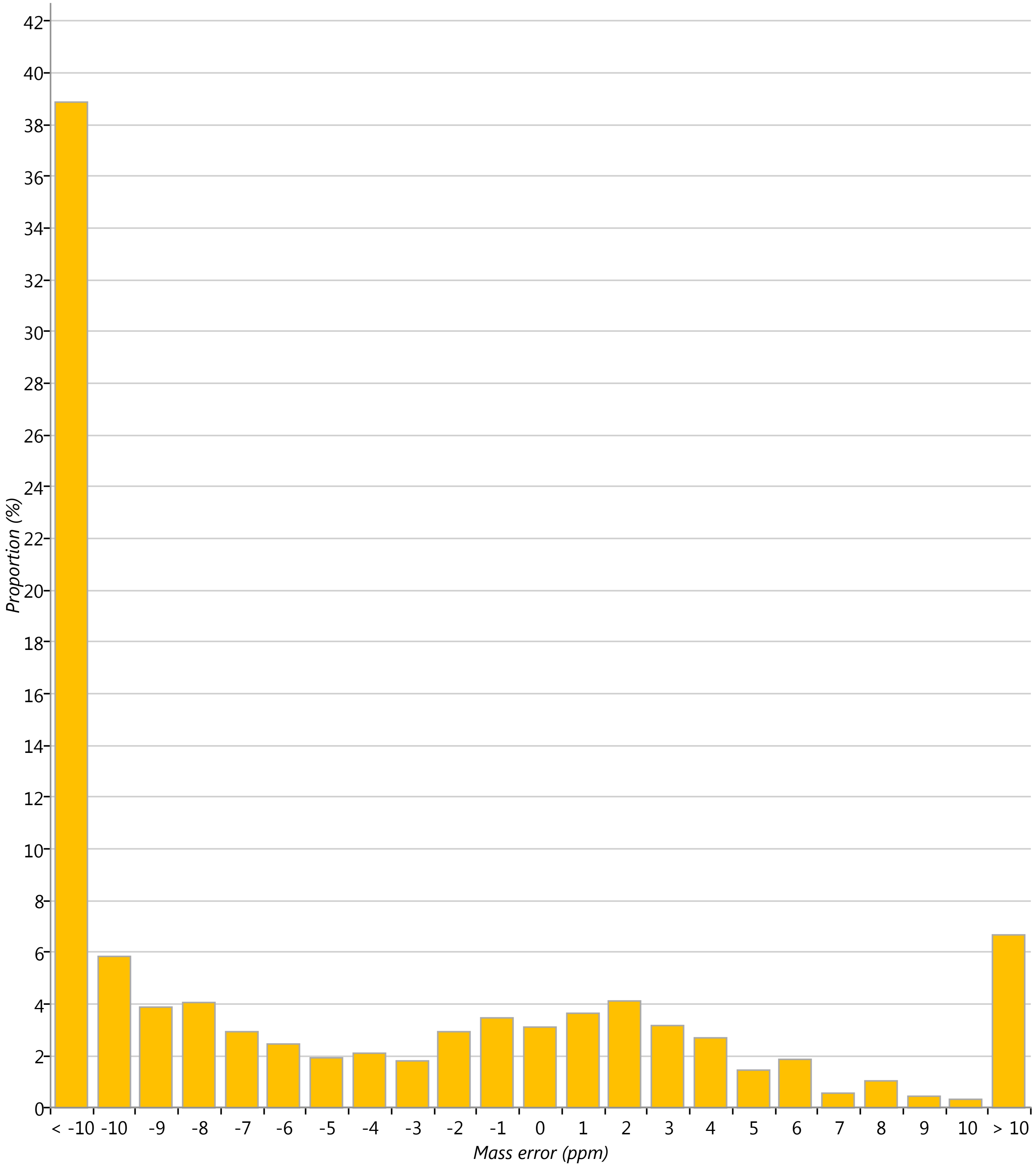

Precursor charges

This chart shows the percentage of peptide ions with various charge states.

Potential problems:

- A skew towards low charge peptide ions may indicate poor ionisation, suggesting that you may need to raise the collision voltage.
- A skew towards high charge peptide ions may indicate excessive ionisation, suggesting that you may need to lower the collision voltage.

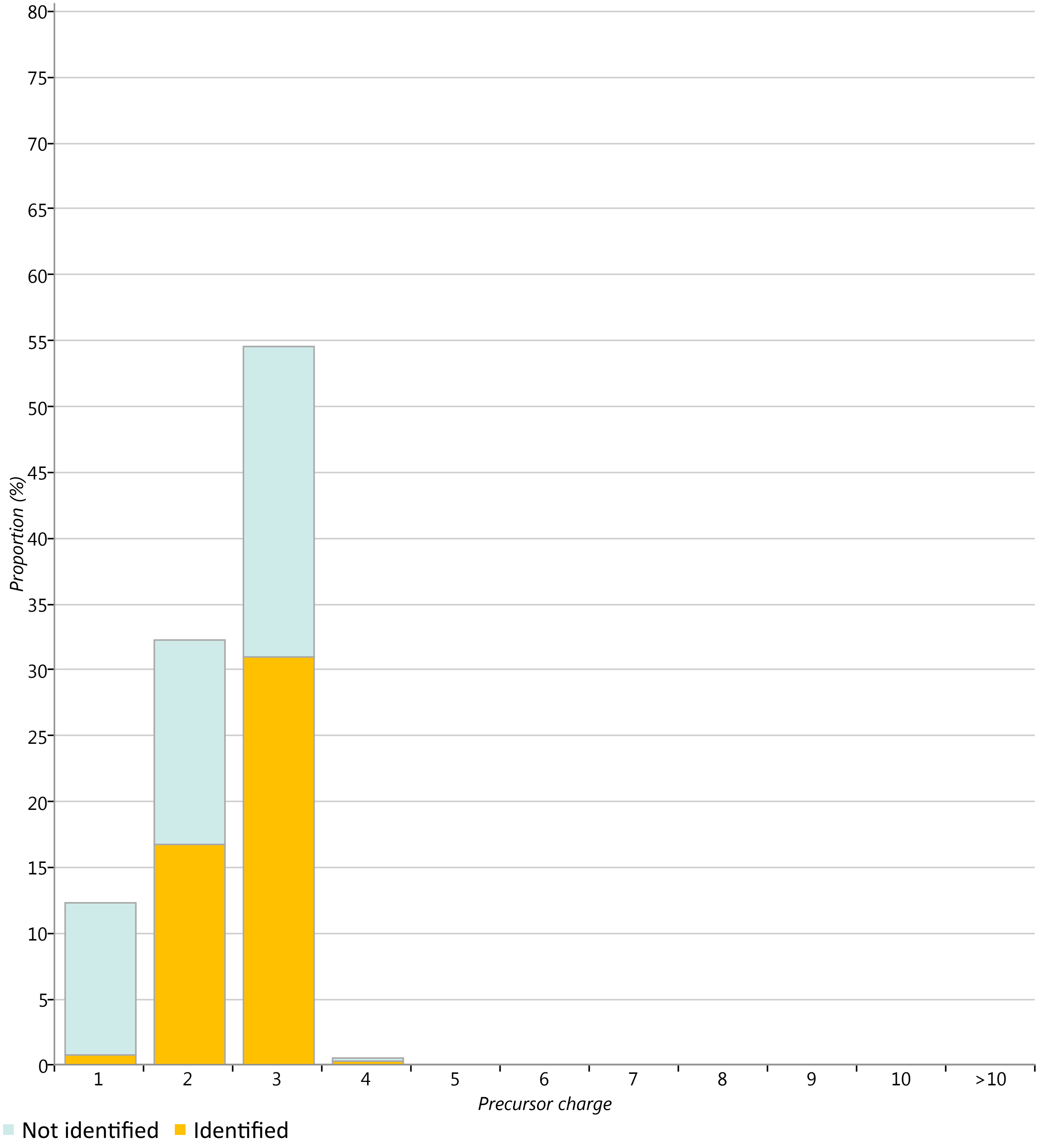

Missed cleavages (assuming trypsin)

This chart shows the distribution of missed cleavages per identified peptide ion across the whole experiment, assuming the proteins were treated with trypsin and using each peptide ion's highest scoring identification. For an alternative visualisation showing the data subdivided by experimental condition, see the *Missed cleavages* metric.

Potential problems:

- A high proportion of missed cleavages may indicate problems in trypsinization.
- An absence of missed cleavages may indicate that the database search parameters were too stringent.

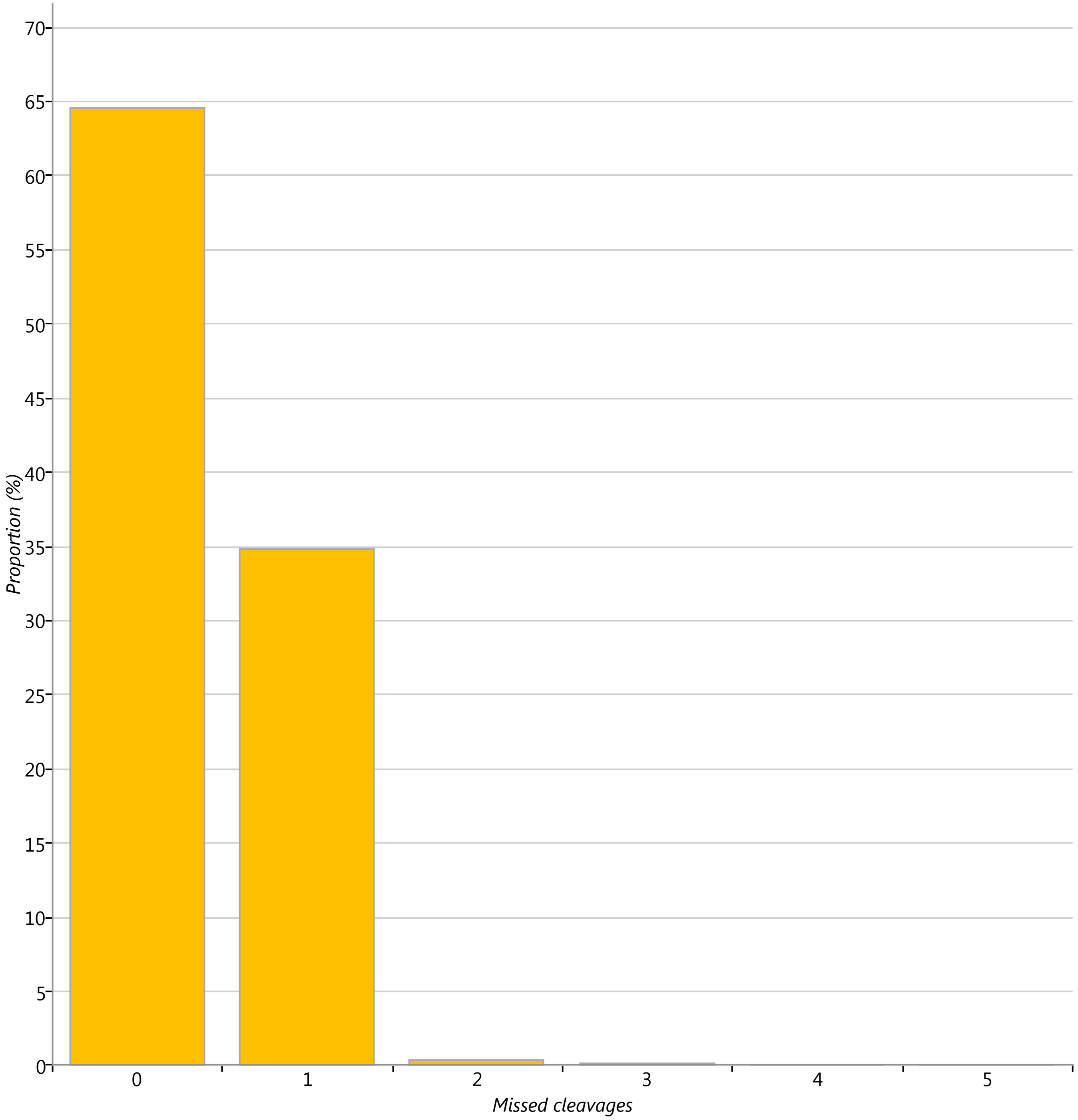

Peptides per protein (overview)

This chart shows the proportional distribution of the number of peptides identified per protein, combined across all runs in the experiment. For an alternative visualisation dividing these data by experimental condition, see the *Peptides per protein (by condition)* metric.

Potential problems:

- An unexpected bias to high or low numbers of peptides may indicate problems with sample preparation or peptide search settings.

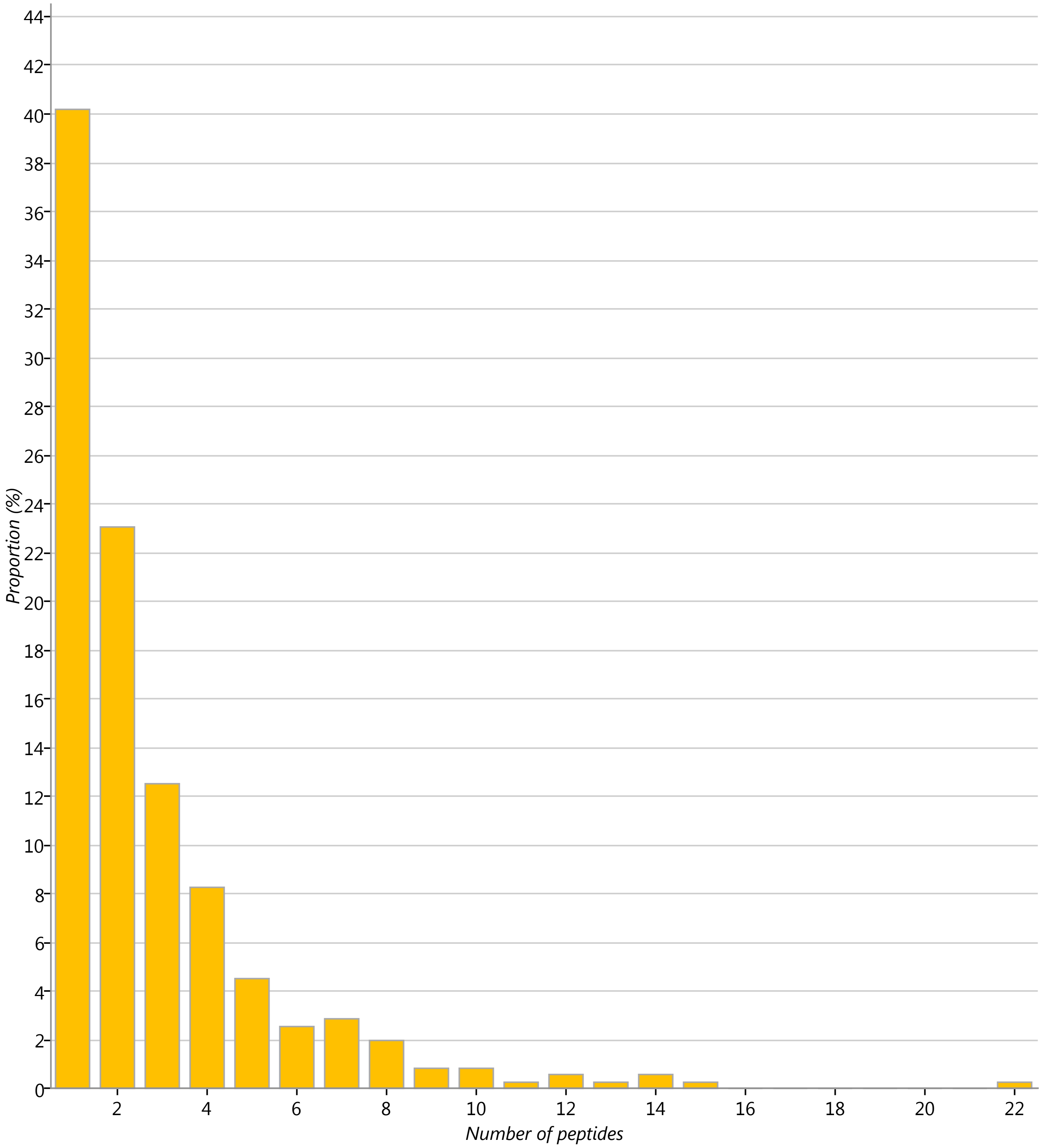

Modifications

The chart below shows the mean number of modifications per peptide ion for runs in the selected experiment design, based on each peptide ion's highest scoring identification. Each run is coloured according to its experimental condition.

Potential problems:

- Unexpectedly low numbers of modifications may indicate that one or more modifications were mistakenly not selected when searching for identifications.
- Variable numbers of modifications across the runs, especially within a given condition, may indicate problems with sample preparation.

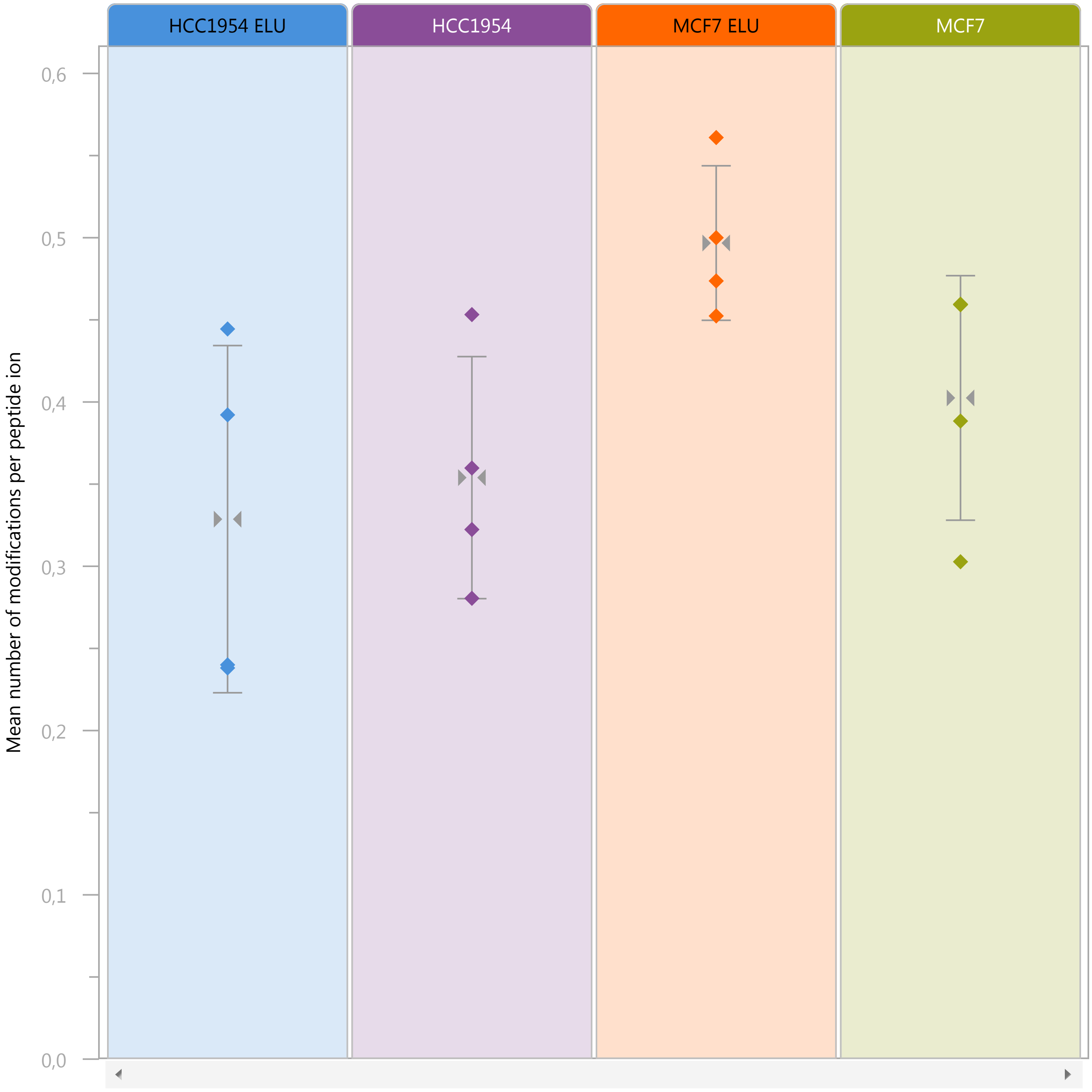

Included modifications Acetyl N-TERM Carbamidomethyl C Carbamyl N-TERM Deamidation N Dean

Missed cleavages

The chart below shows the mean number of missed cleavages per peptide ion for runs within the selected experiment design, based on each peptide ion's highest scoring identification. The chart assumes the use of trypsin for digestion. Each run is coloured according to its experimental condition.

Potential problems:

- A high number of missed cleavages may indicate problems in trypsinization.
- An absence of missed cleavages may indicate that database search parameters were too stringent.

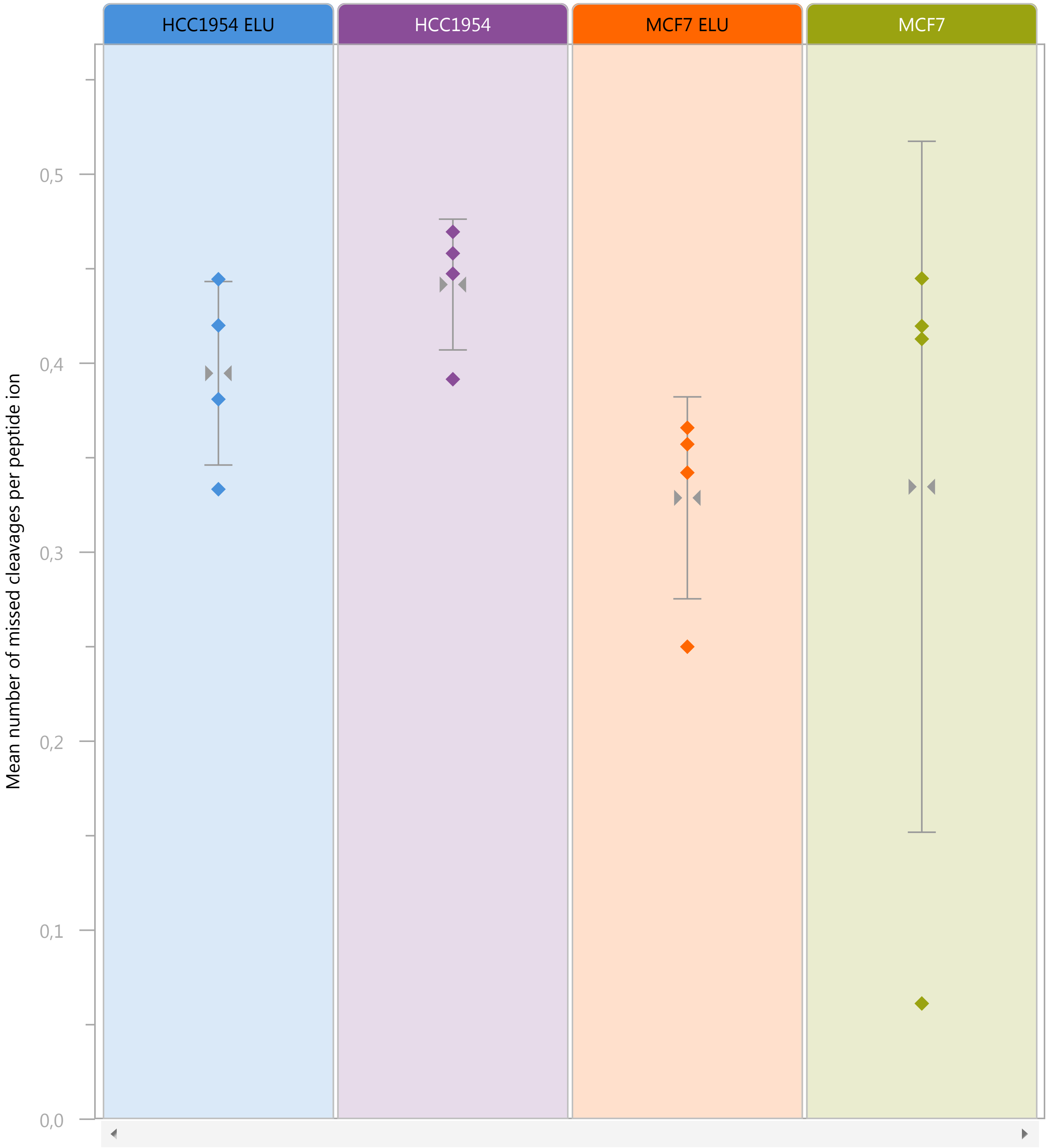

Abundance dynamic range

The charts below show abundance histograms for peptide ions, calculated using the current experiment design. Each chart shows the mean abundance for peptide ions within an experimental condition's runs. Data are binned by Log10 of their normalised abundance and shown as a proportion of the total. Identified peptide ions are shown in yellow.

Potential problems:

- A heavily right-skewed histogram of *identified* peptide ions indicates you may be missing identifications with low abundance.
- A heavily right skewed total (summed identified and unidentified) histogram may indicate saturation at high abundance, which will affect the accuracy of quantitation.

HCC1954 ELU

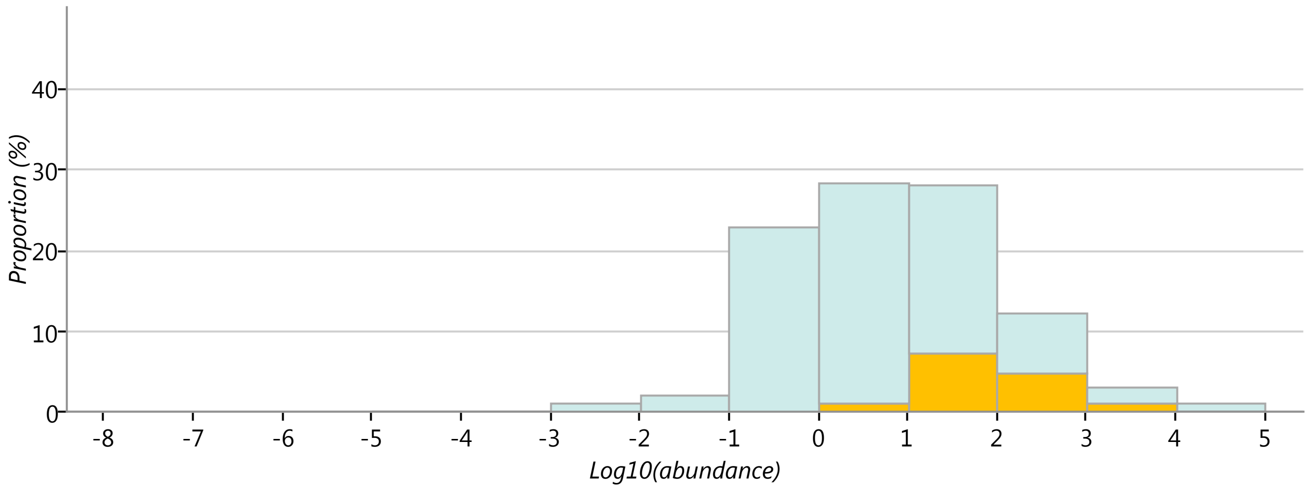

HCC1954

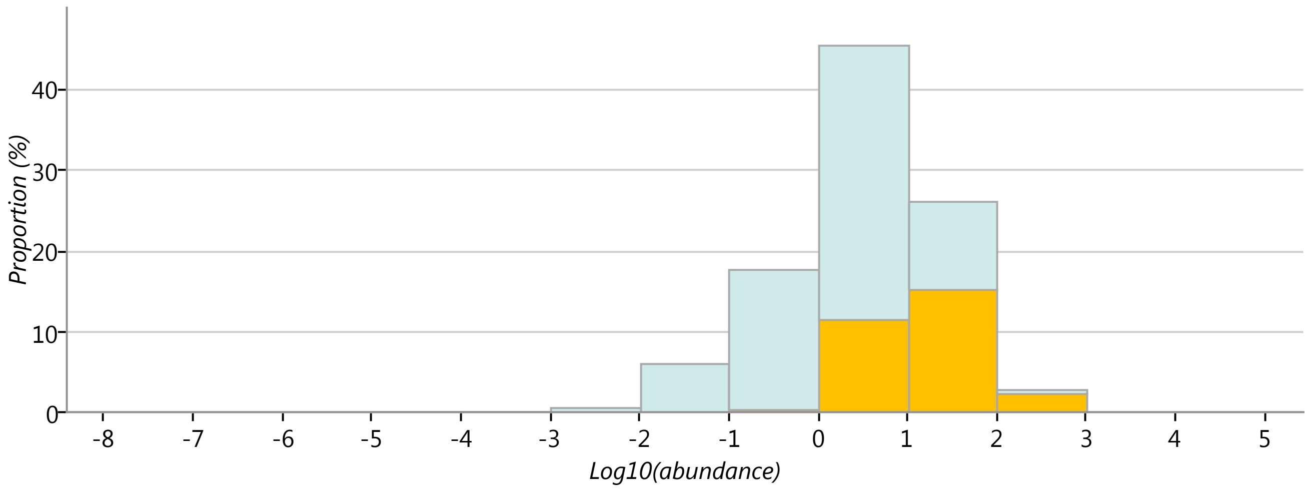

Not identified ■ Identified

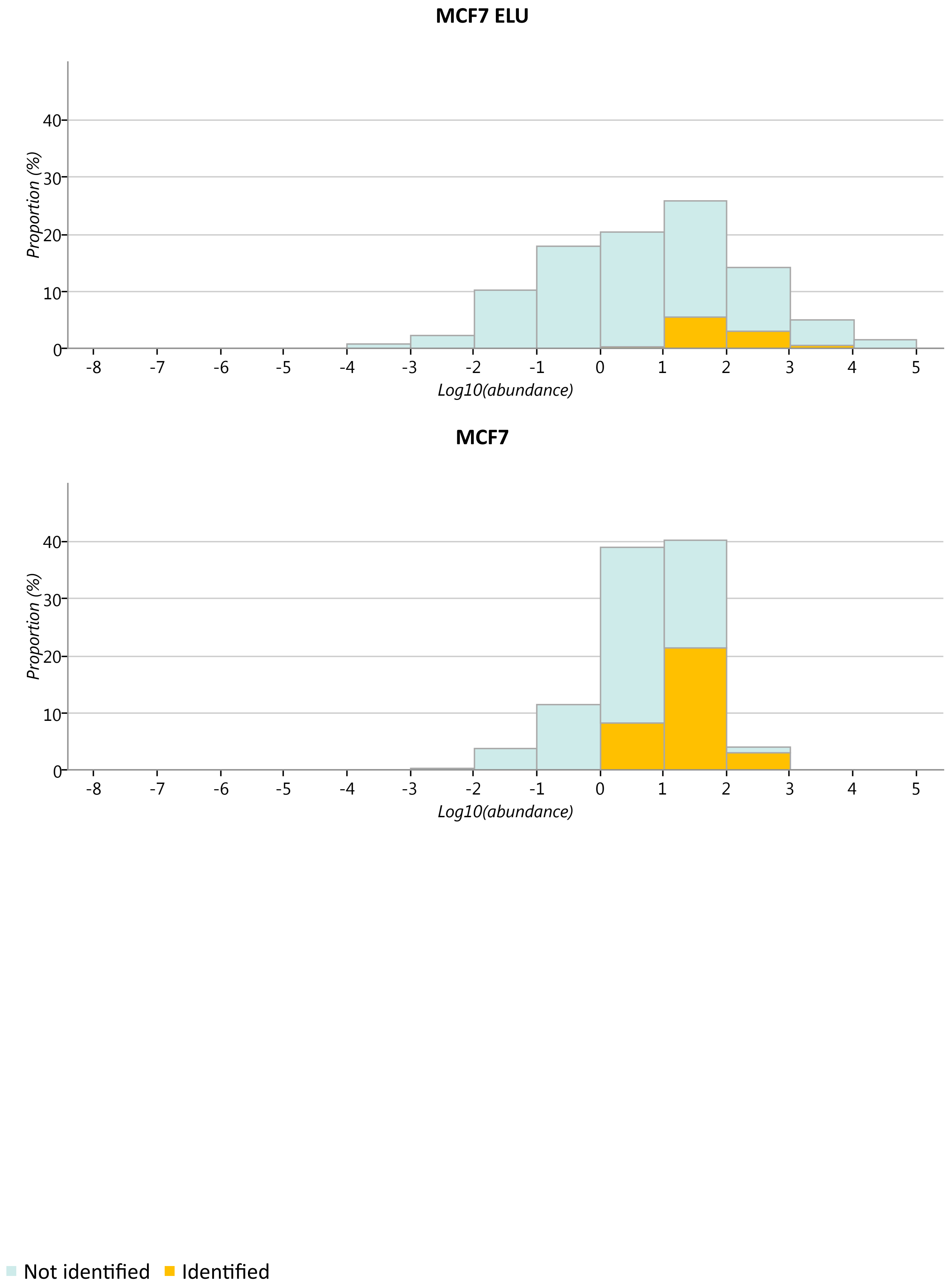
MCF7 ELU
Proportion (%)
Log10(abundance)
MCF7
Proportion (%)
Log10(abundance)
Not identified
Identified

Mass accuracy

The chart below shows all identified peptide ions in your experiment, plotting peptide ion m/z against the ppm error in its highest scoring identification. Blue points indicate peptide ions which have a mass error within 2 standard deviations of the mean, yellow points indicate peptide ions with a mass error more than 2 standard deviations from the mean.

Potential problems:

- A dependence of ppm mass error on m/z may indicate poor calibration.
- If the standard deviation of the data is large in ppm terms, this may indicate that your search parameters are too lenient.

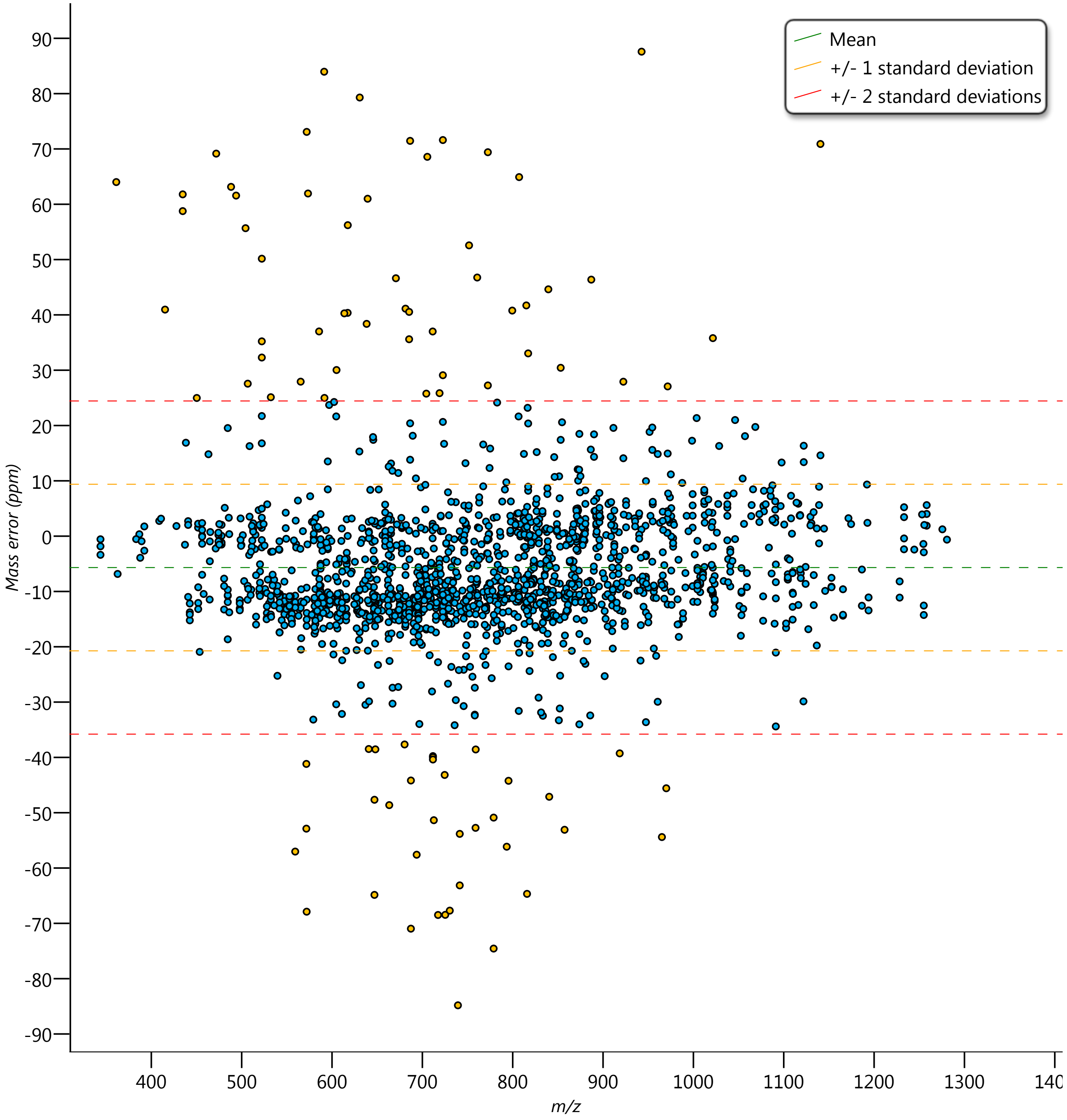

Scan rates

The charts below show the MS1 scan rate over the duration of your runs, using the current experiment design. Each chart shows the scan rate variation within an experimental condition's runs. The named run is highlighted in black. The variability between runs is shown as a grey area. The y-axis shows scans per second in a 30-second window centred on each x-axis time point.

Potential problems:

- A highly varying scan rate means quantitation accuracy may be negatively affected.

HCC1954 ELU **Run:** 20140724_DAP_HCC1954_ELUIDO_2ug_inj5_STEP1

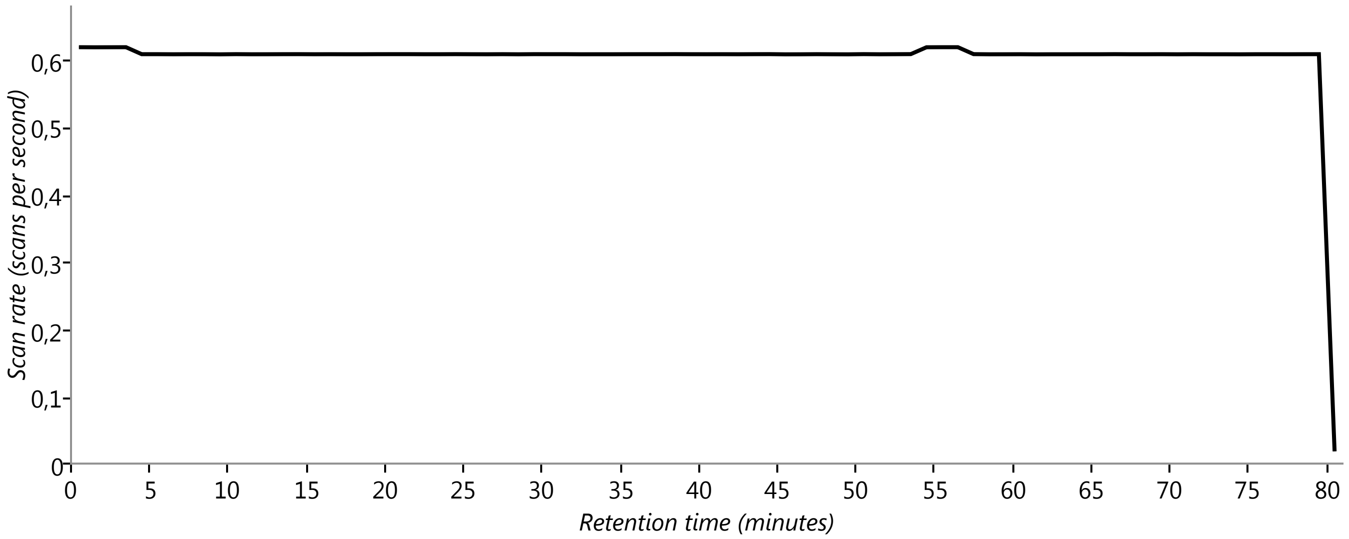

HCC1954 **Run:** 20140801_DAP_HCC1954_NAO_RETIDO_2ug_inj3_STEP1

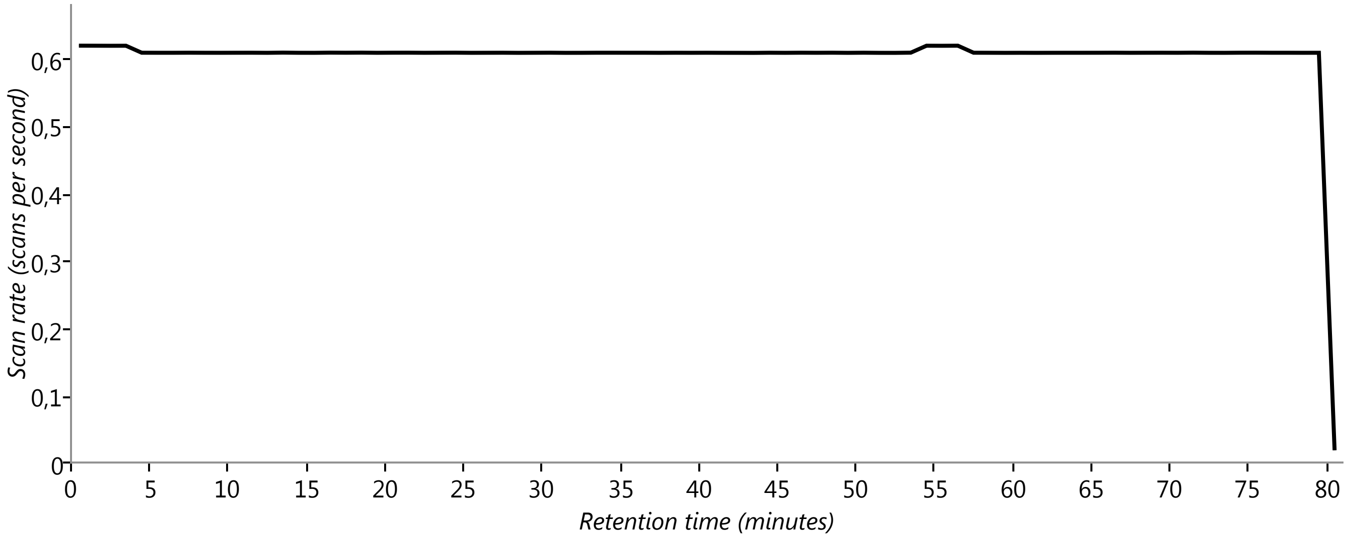

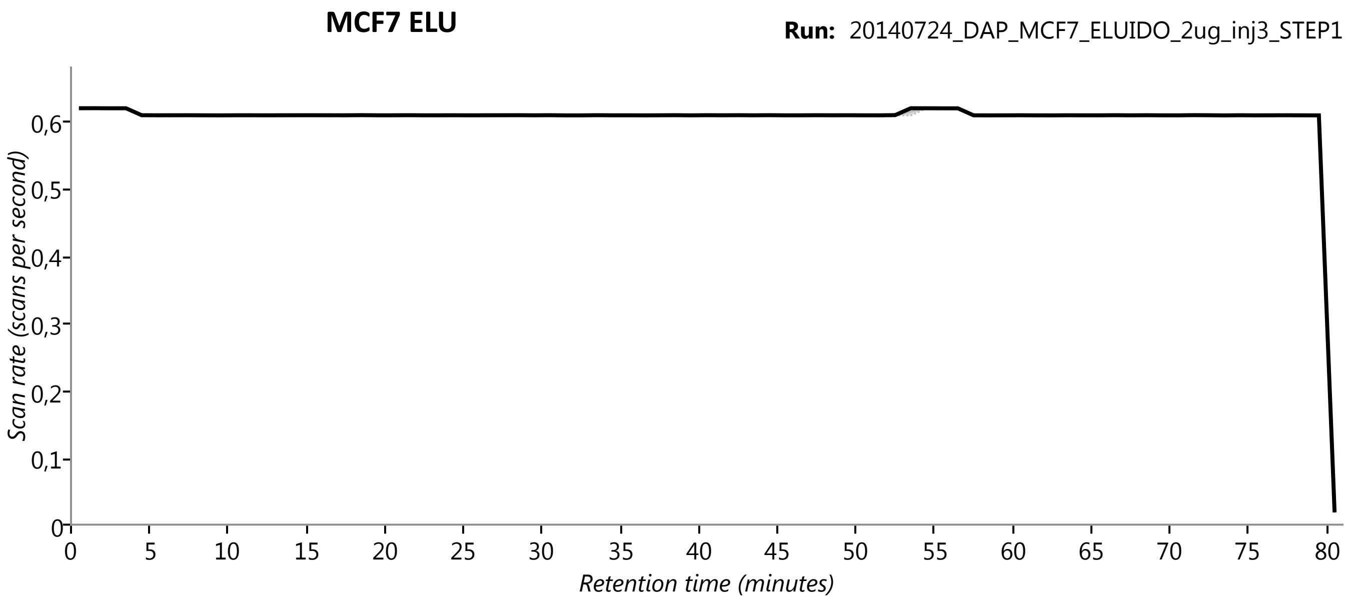

MCF7 ELU
Run: 20140724_DAP_MCF7_ELUIDO_2ug_inj3_STEP1
Scan rate (scans per second)
0,6
0,5
0,4
0,3
0,2
0,1
0
0 5 10 15 20 25 30 35 40 45 50 55 60 65 70 75 80
Retention time (minutes)

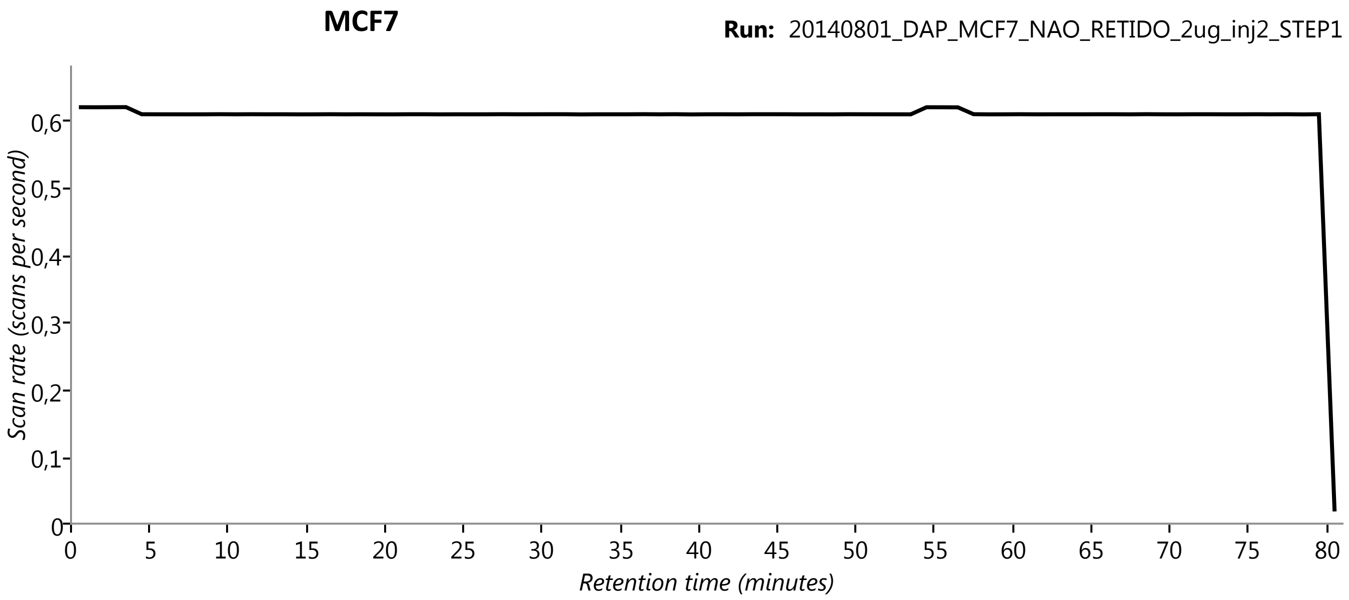

MCF7
Run: 20140801_DAP_MCF7_NAO_RETIDO_2ug_inj2_STEP1
Scan rate (scans per second)
0,6
0,5
0,4
0,3
0,2
0,1
0
0 5 10 15 20 25 30 35 40 45 50 55 60 65 70 75 80
Retention time (minutes)

Proteins

The chart below shows the number of proteins identified in each of the runs within the selected experiment design. Each run is coloured according to its experimental condition.

Potential problems:

- Any exceptionally high or low values may indicate problems with sample preparation, chromatography, or instrument settings.

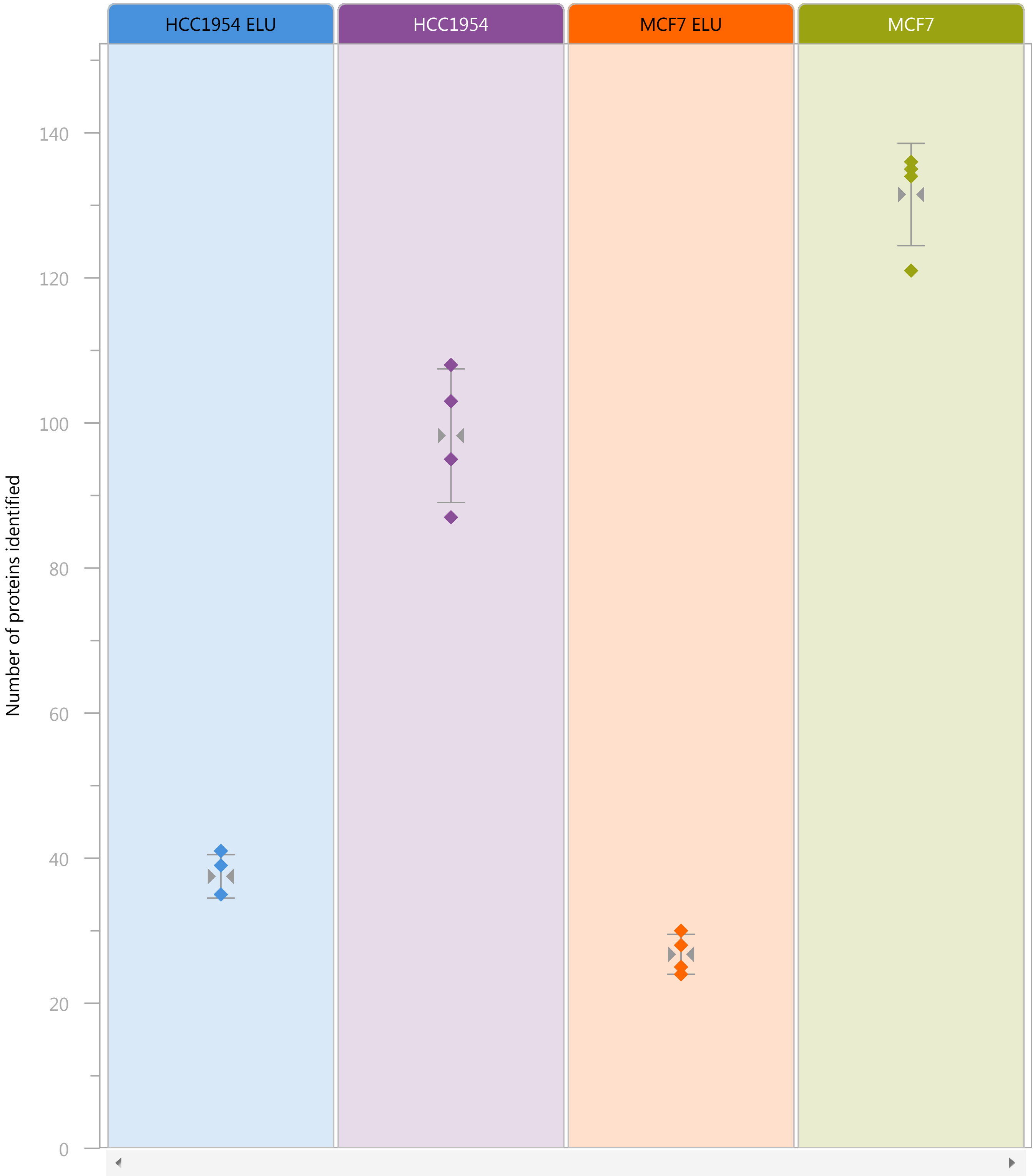

Peptides

The chart below shows the number of peptides identified in each of the experiment's runs. Each run is coloured according to its experimental condition.

Potential problems:

- Any exceptionally high or low values may indicate problems with sample preparation, chromatography, or instrument settings.

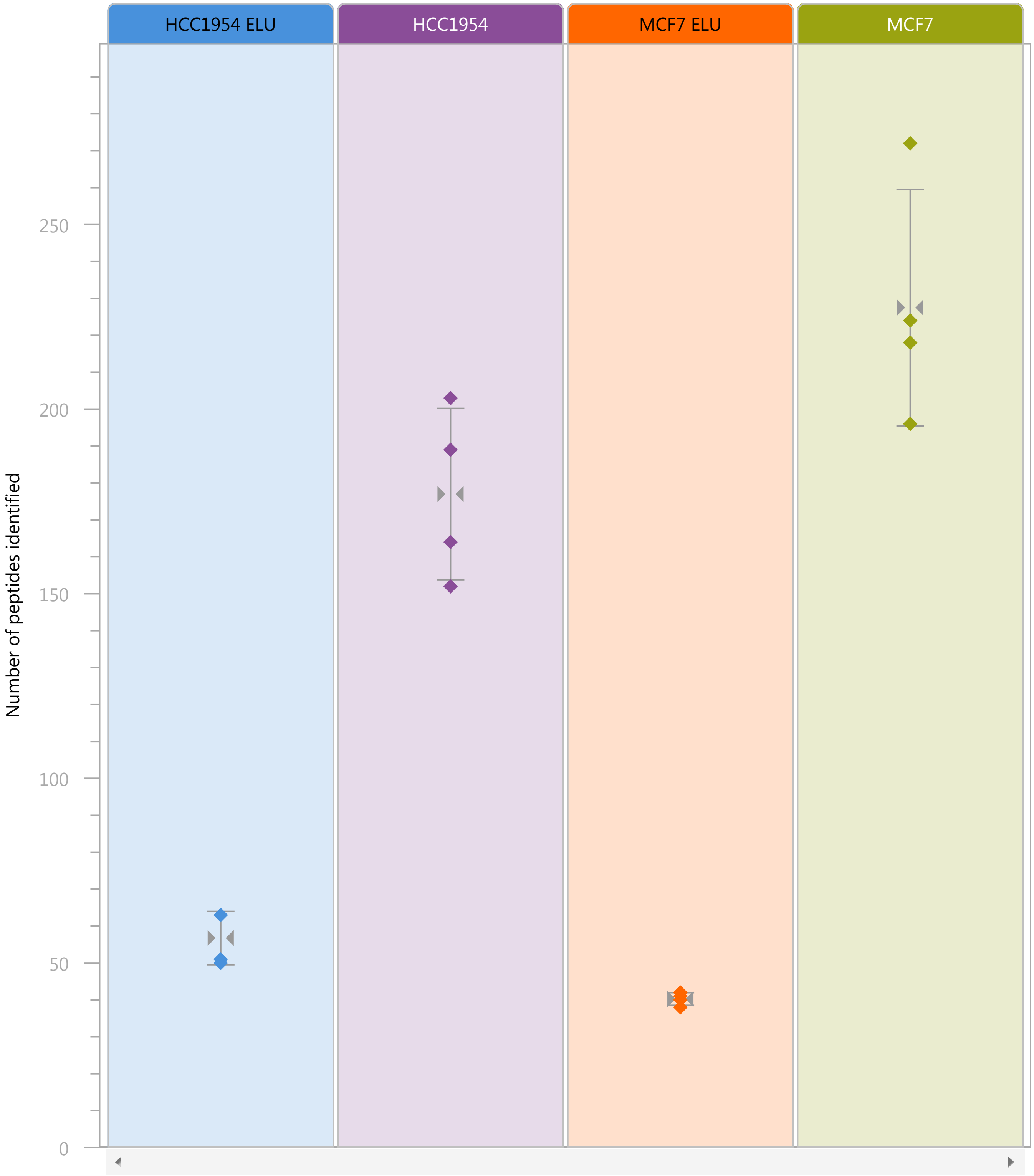

Peptides per protein (by condition)

The chart below shows the average number of peptides per protein in each run within the selected experiment design. For a given run, the value shown is calculated using only identifications from that run.

Potential problems:

- Any exceptionally high or low values may indicate problems with sample preparation or peptide search settings.

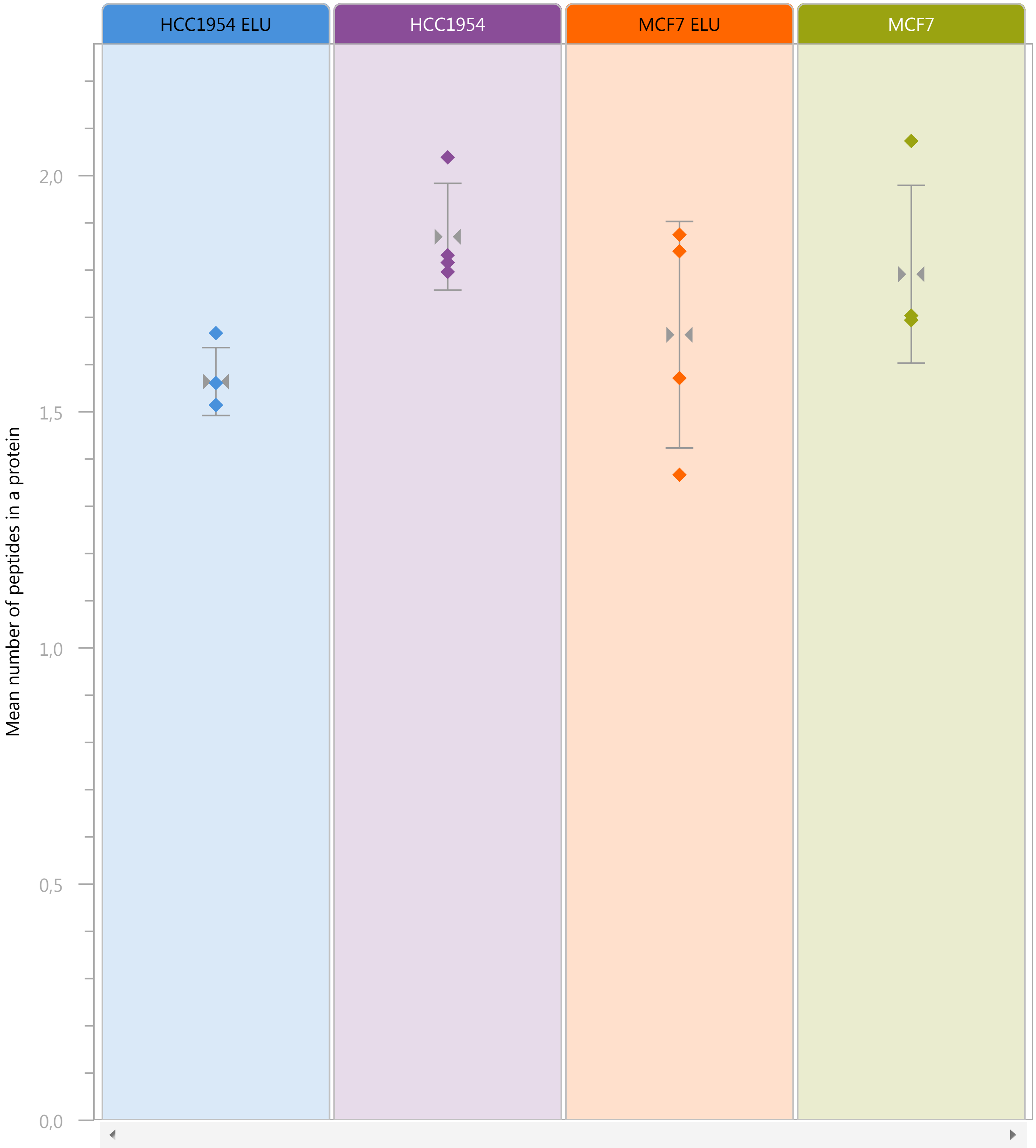

Percentage of peptide ions identified

The chart below shows the number of peptide ions identified in each of the runs within the selected experiment design, expressed as a percentage of the total number of peptide ions. Each run is coloured according to its experimental condition.

Potential problems:

- Any exceptionally high or low values may indicate problems with sample preparation, chromatography, or instrument settings.
- Very low values across all runs may indicate a problem with the database that was searched for identifications, or with other search parameters.

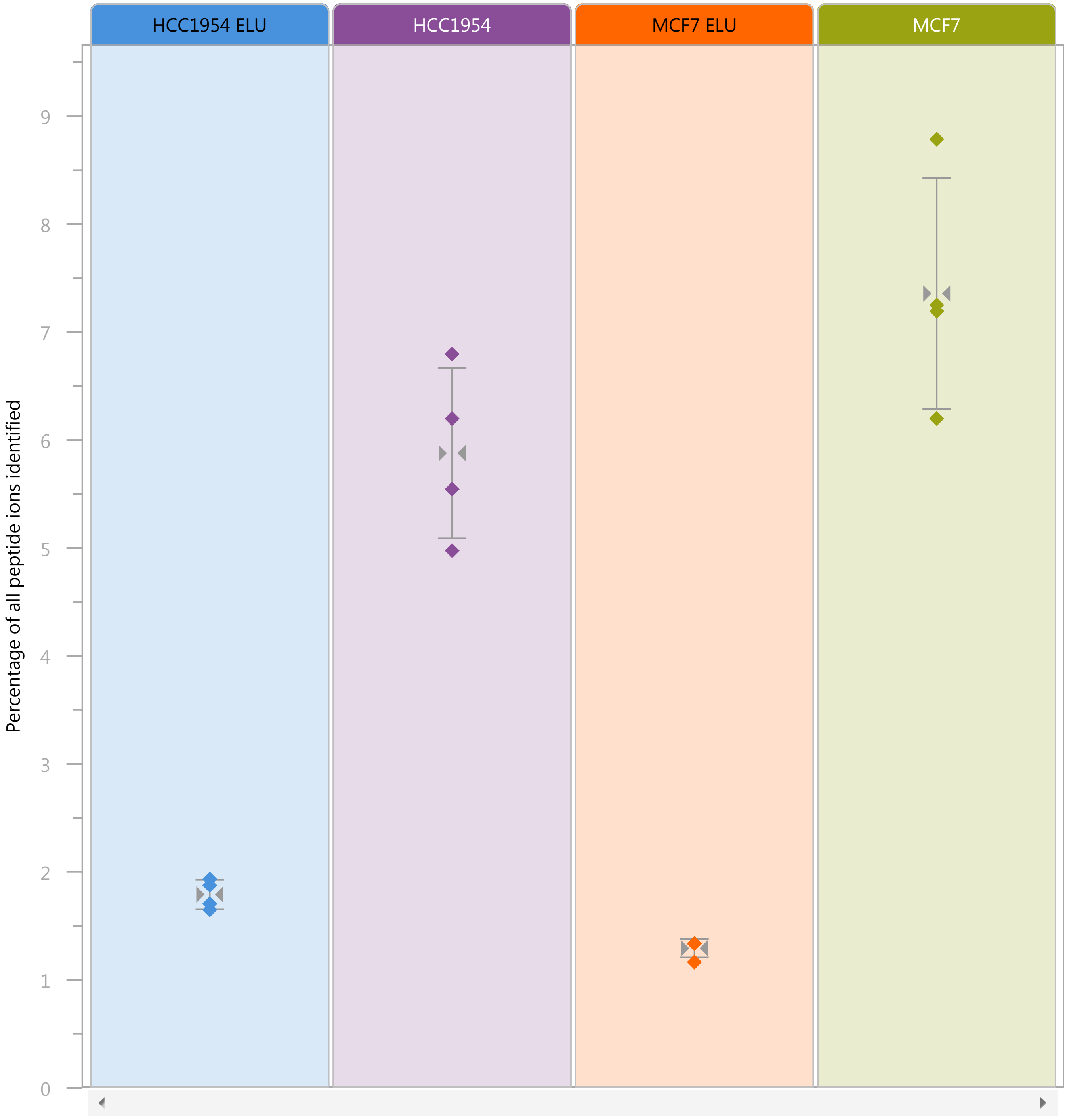

Protein overlap

The Venn diagram shows the number of proteins identified uniquely in the selected conditions and the number that are shared between conditions. Use the list on the right to select two or three conditions to explore.

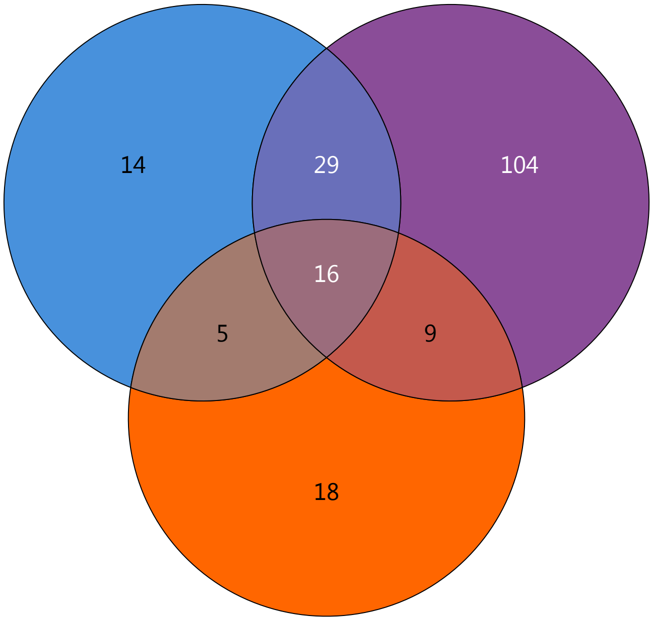

Select experimental conditions

- HCC1954 ELU
- HCC1954
- MCF7 ELU

HCC1954 X MCF7 STEP2

QC Metrics reported by Progenesis QI for proteomics v2.0.5556.29015
Date: 10-Jan-2020 at 09:20
Experiment design: HCC1954 X MCF7 STEP2

This QC report contains **all metrics** for the experiment named above.

Reported metrics

0 of the 19 metrics reported have been manually flagged for attention.

- Identifications overview
- LC peak width
- Peptide ion dynamic range
- Precursor m/z
- Precursor retention time
- Mass errors
- Precursor charges
- Missed cleavages (assuming trypsin)
- Peptides per protein (overview)
- Modifications
- Missed cleavages
- Abundance dynamic range
- Mass accuracy
- Scan rates
- Proteins
- Peptides
- Peptides per protein (by condition)
- Percentage of peptide ions identified
- Protein overlap

Identifications overview

The chart below provides the number of identifications per run by category. Data are plotted for all runs in the current experiment design. The box represents the interquartile range and the whiskers the maximum and minimum.

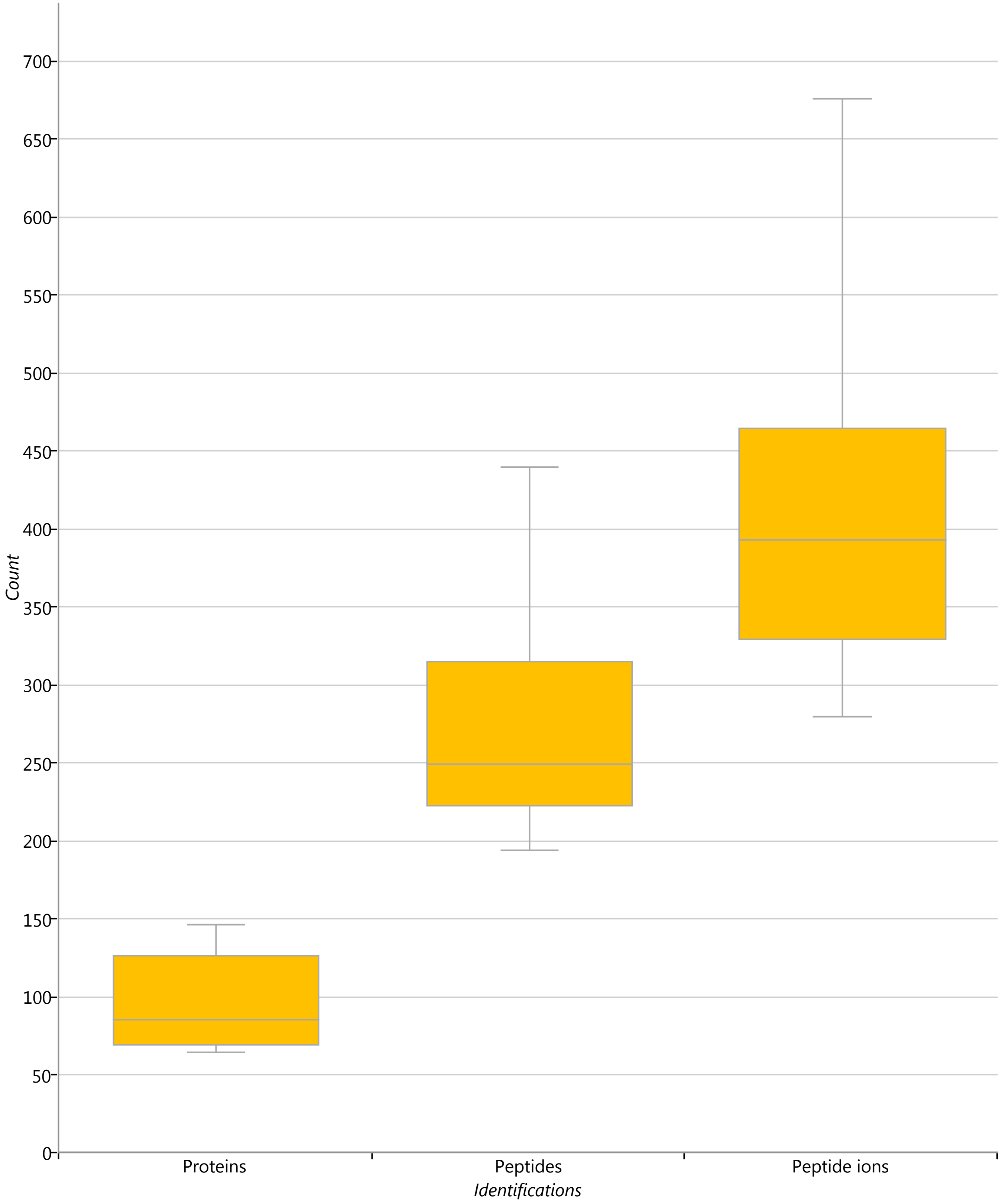

LC peak width

This chart shows the proportional distribution of peak widths in retention time, for all the detected peptide ions in the experiment. Widths are binned into categories for simple comparison. Identified peptide ions are shown in yellow.

Potential problems:

- A peak width that is different from that expected for your setup may indicate a problem with the chromatographic separation.

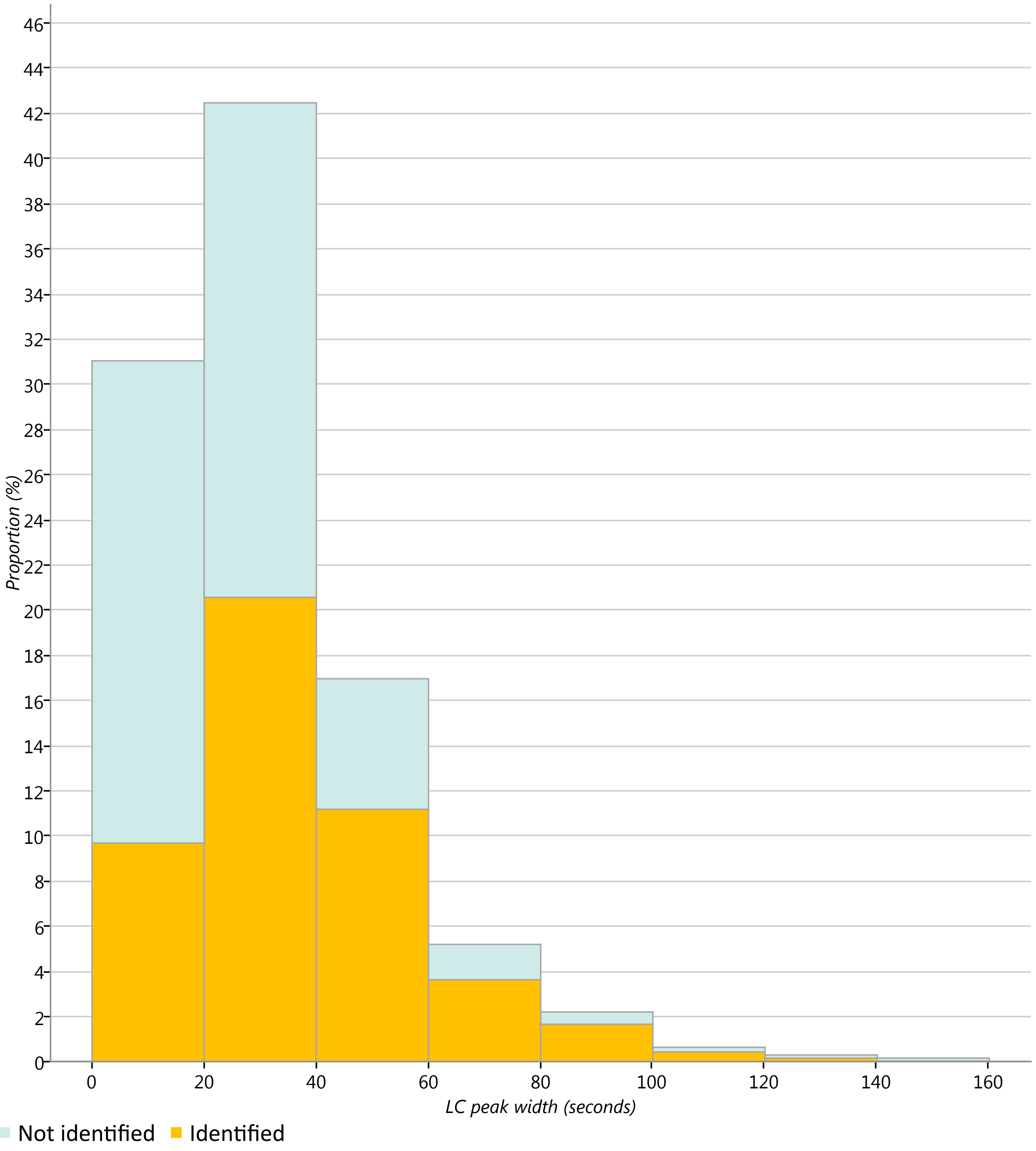

Peptide ion dynamic range

This chart shows log abundance for peptide ions, calculated using the current experiment design. Each peptide ion is assigned its abundance based on the highest mean value for any condition's runs. Data are binned and shown as a proportion. Identified peptide ions are shown in yellow.

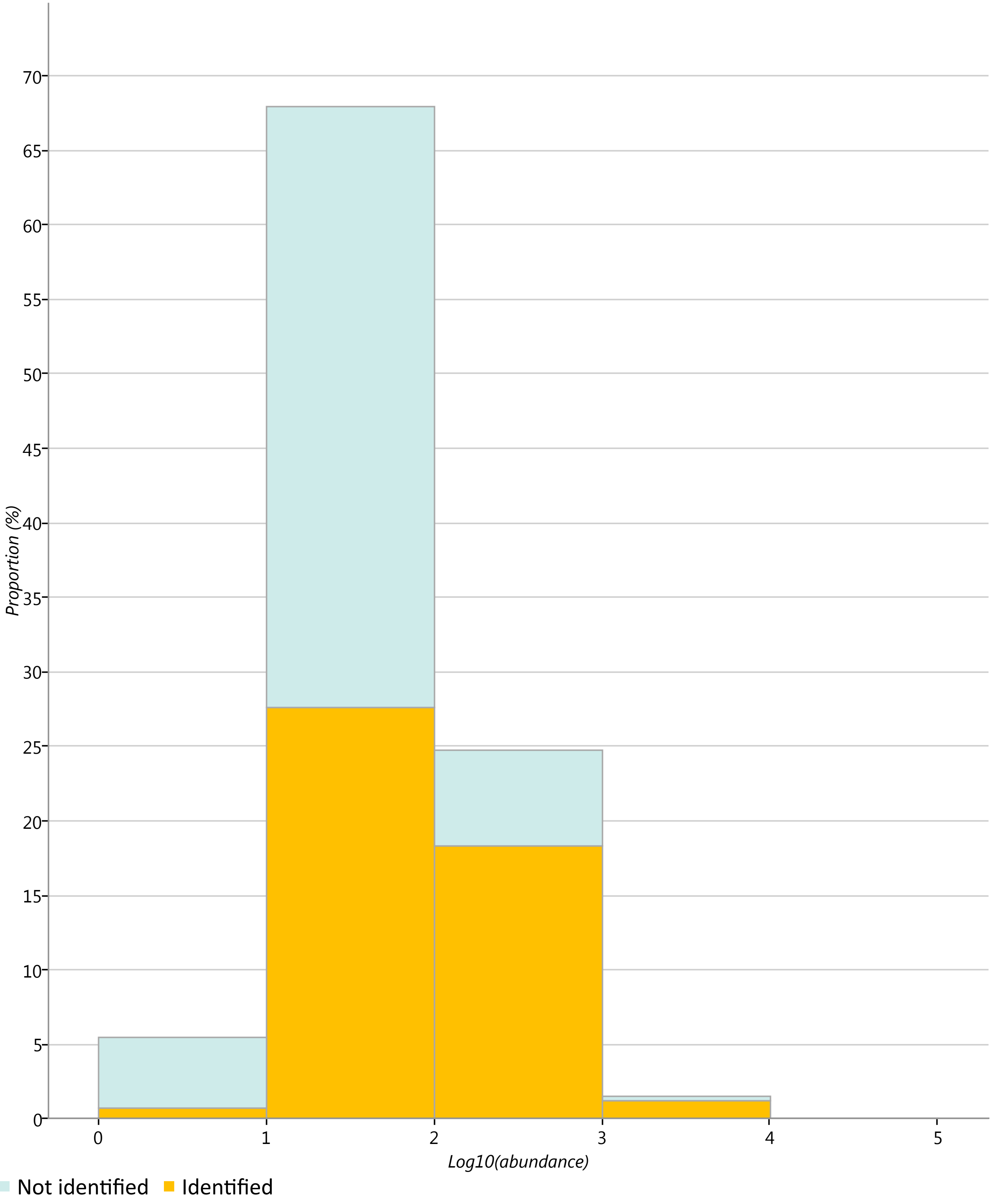

Precursor m/z

This chart shows the distribution of m/z values for all peptide ions in the experiment. Identified peptide ions are shown in yellow.

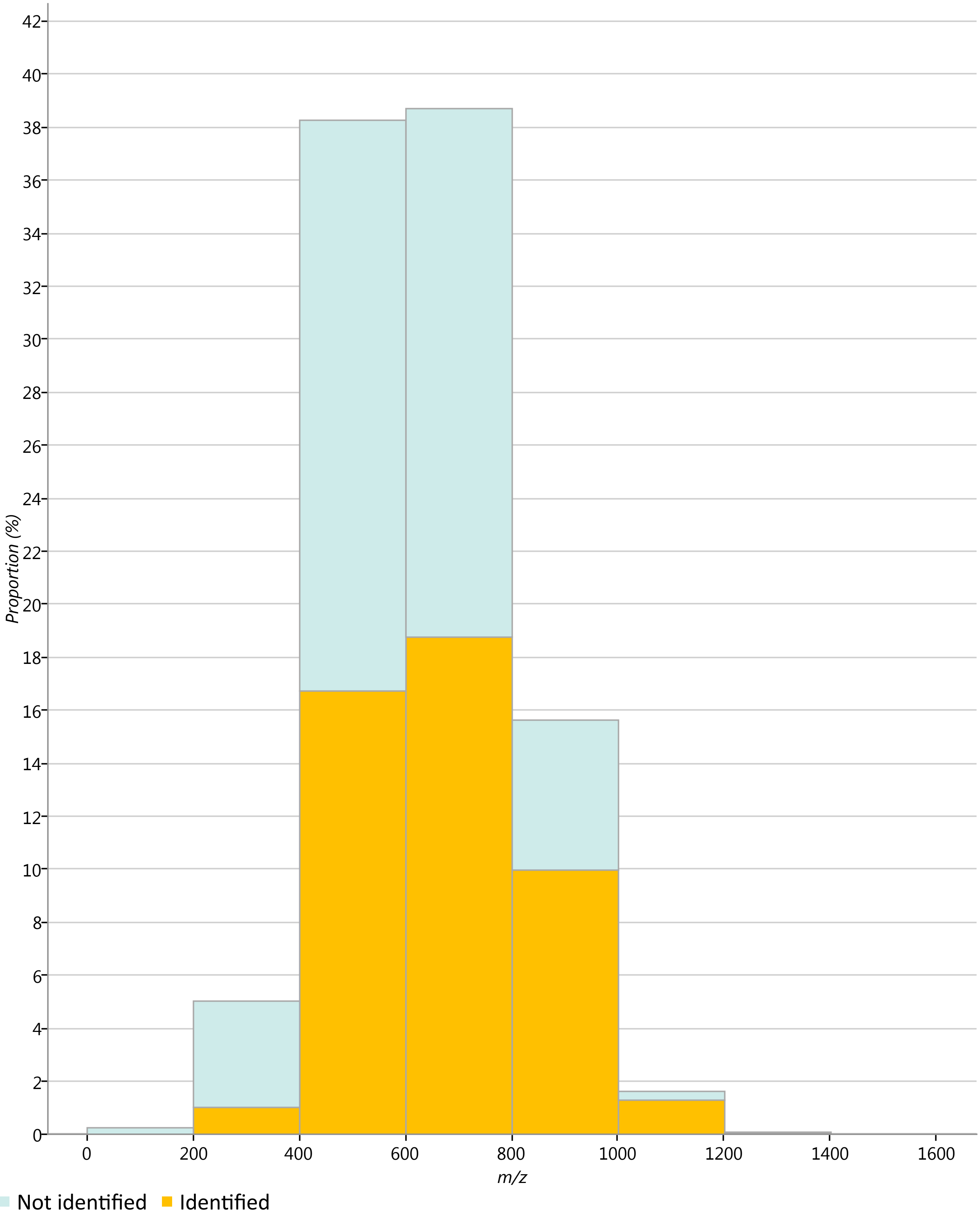

Precursor retention time

This chart shows the proportion of precursor peptide ions eluting into each quartile of the LC run. Identified peptide ions are shown in yellow.

Potential problems:

- A majority of peptide ions eluting at the start or end of the run may indicate problems with the chromatographic separation or that the setup is not ideal for a successful separation.

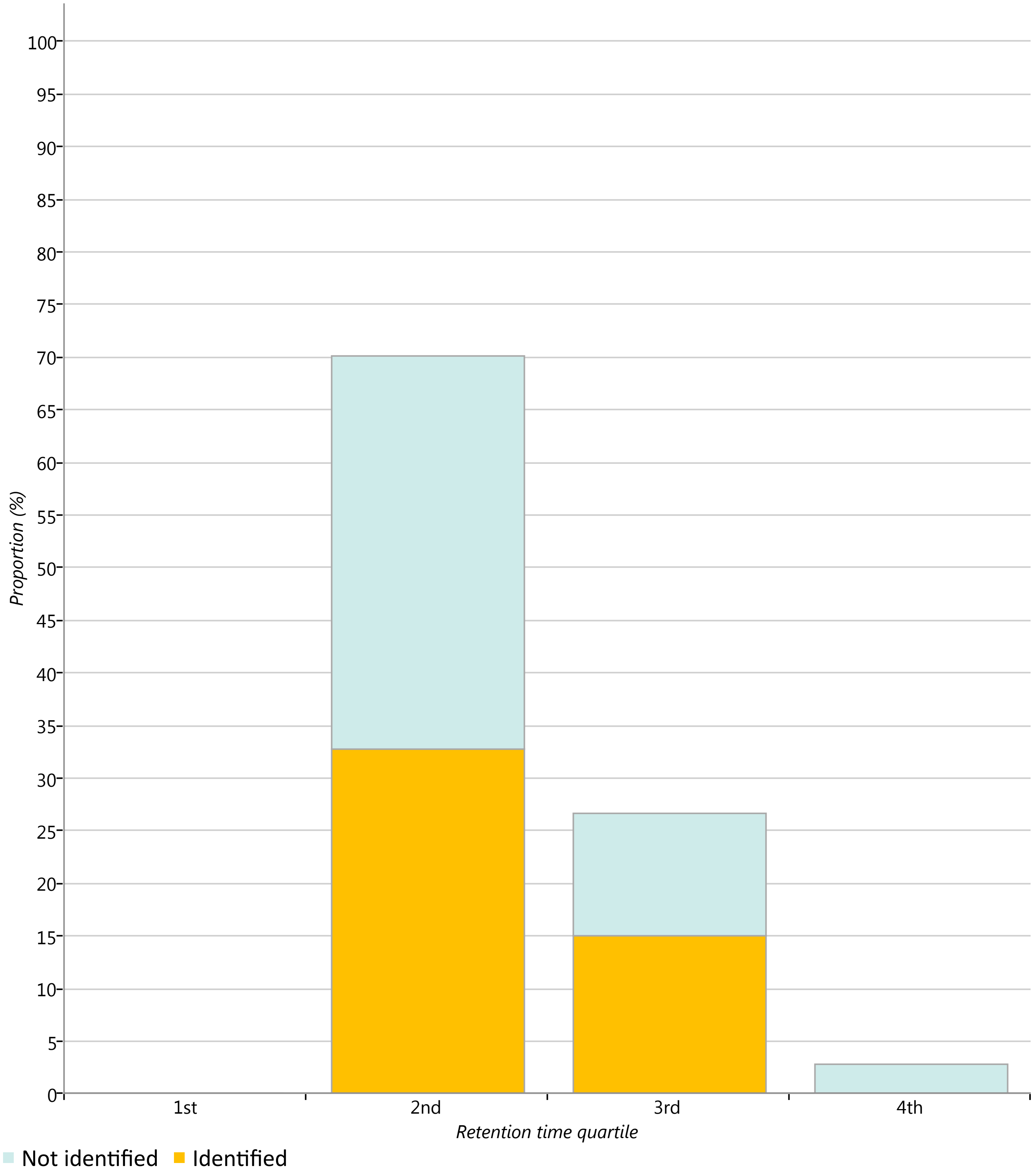

Mass errors

This chart shows the mass error calculated for identified peptide ions. The proportion of peptide ions with a given binned error value is plotted. For a more detailed plot, see the *Mass accuracy* metric.

Potential problems:

- A larger than expected spread could indicate that search parameters are too lenient.
- A bias of the peak from zero ppm could indicate instrument bias.

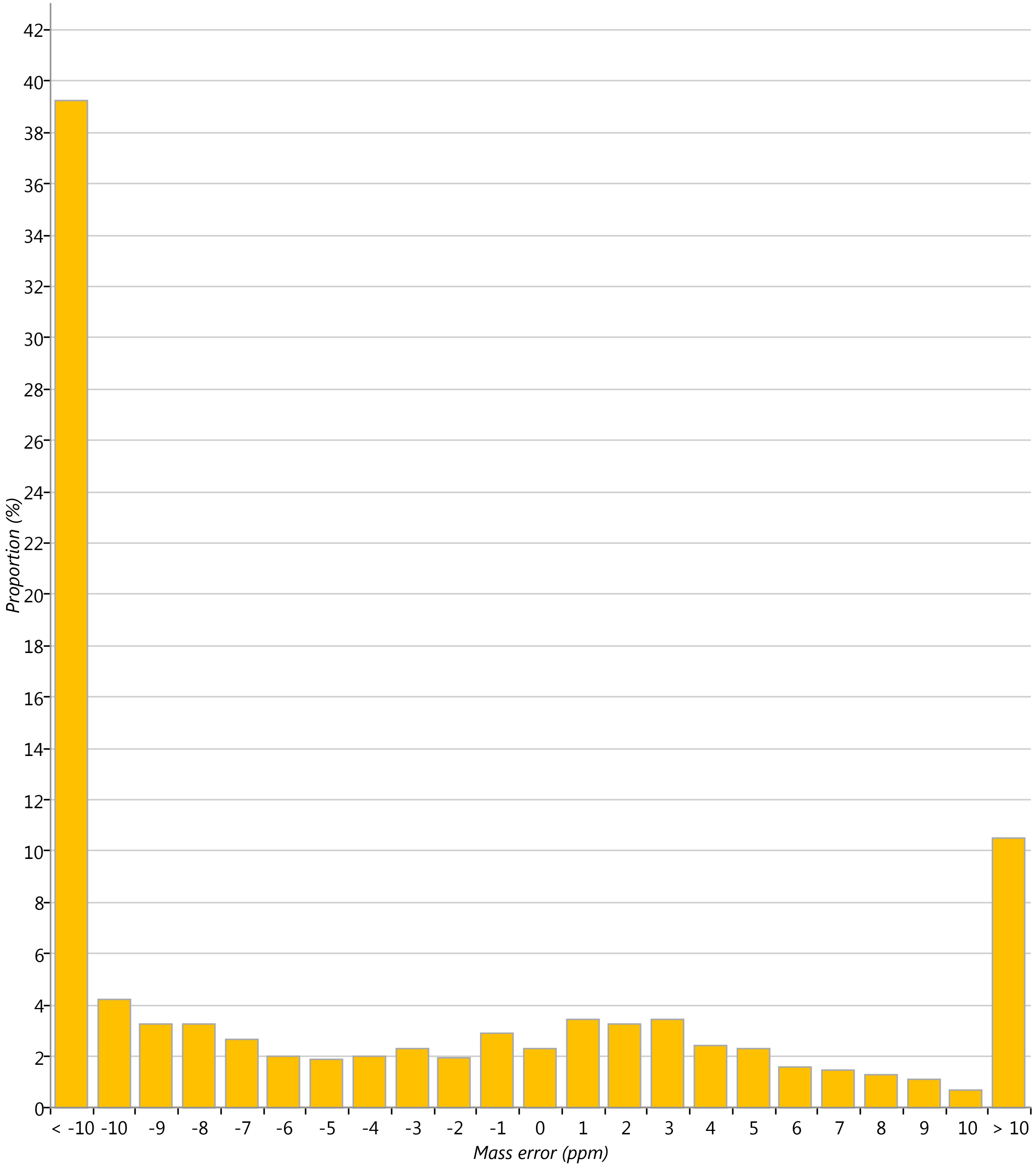

Precursor charges

This chart shows the percentage of peptide ions with various charge states.

Potential problems:

- A skew towards low charge peptide ions may indicate poor ionisation, suggesting that you may need to raise the collision voltage.
- A skew towards high charge peptide ions may indicate excessive ionisation, suggesting that you may need to lower the collision voltage.

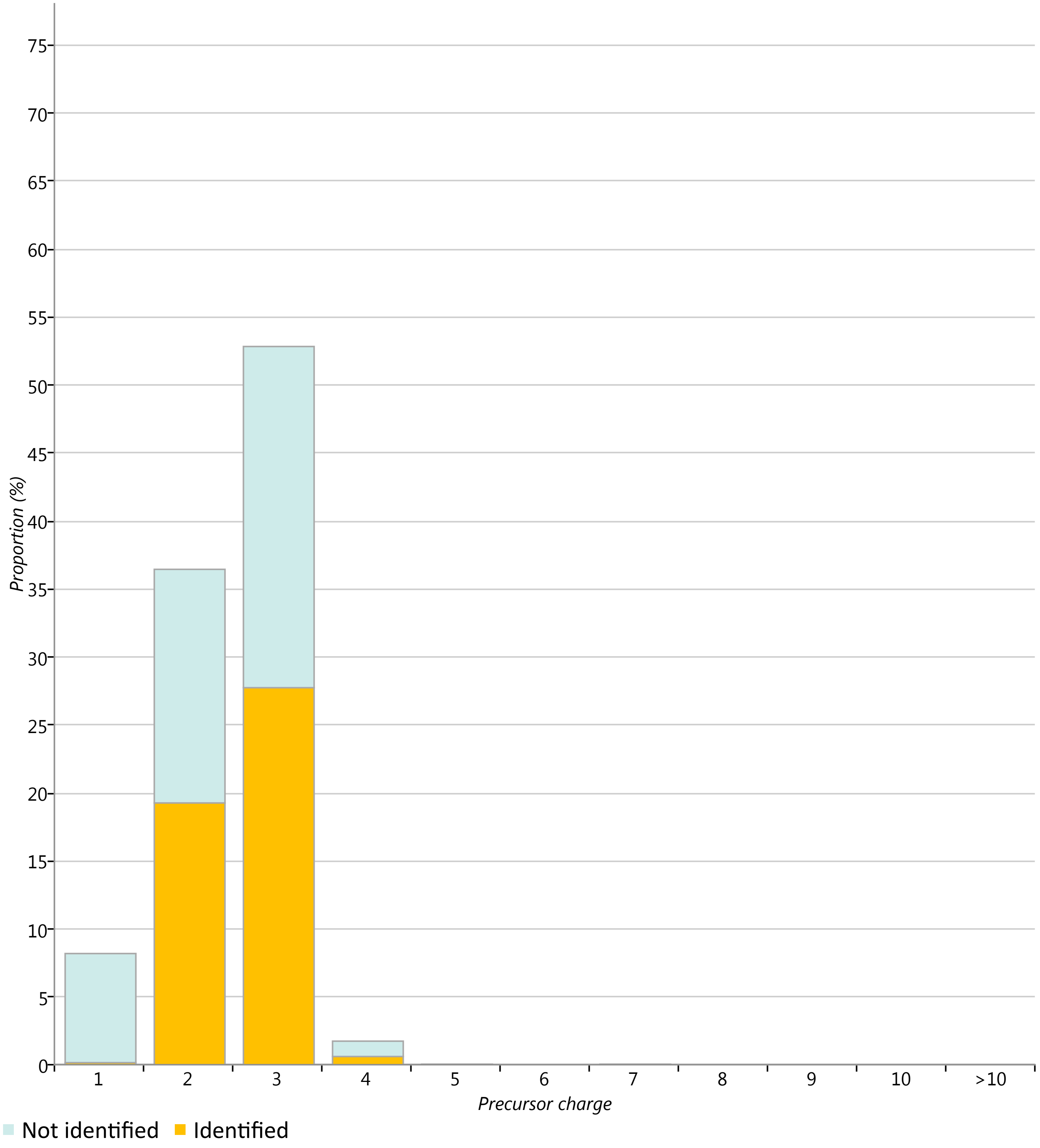

Missed cleavages (assuming trypsin)

This chart shows the distribution of missed cleavages per identified peptide ion across the whole experiment, assuming the proteins were treated with trypsin and using each peptide ion's highest scoring identification. For an alternative visualisation showing the data subdivided by experimental condition, see the *Missed cleavages* metric.

Potential problems:

- A high proportion of missed cleavages may indicate problems in trypsinization.
- An absence of missed cleavages may indicate that the database search parameters were too stringent.

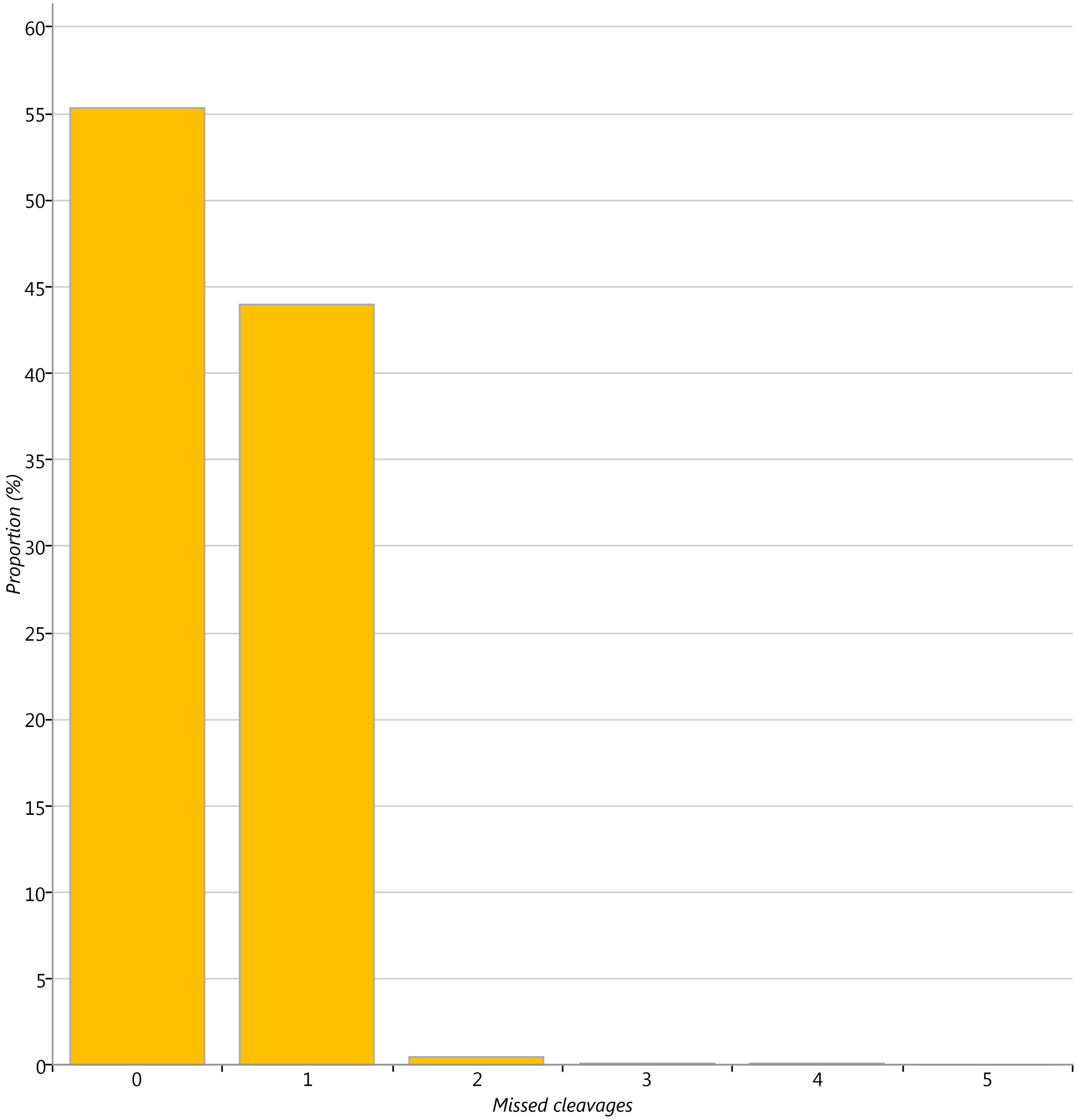

Peptides per protein (overview)

This chart shows the proportional distribution of the number of peptides identified per protein, combined across all runs in the experiment. For an alternative visualisation dividing these data by experimental condition, see the *Peptides per protein (by condition)* metric.

Potential problems:

- An unexpected bias to high or low numbers of peptides may indicate problems with sample preparation or peptide search settings.

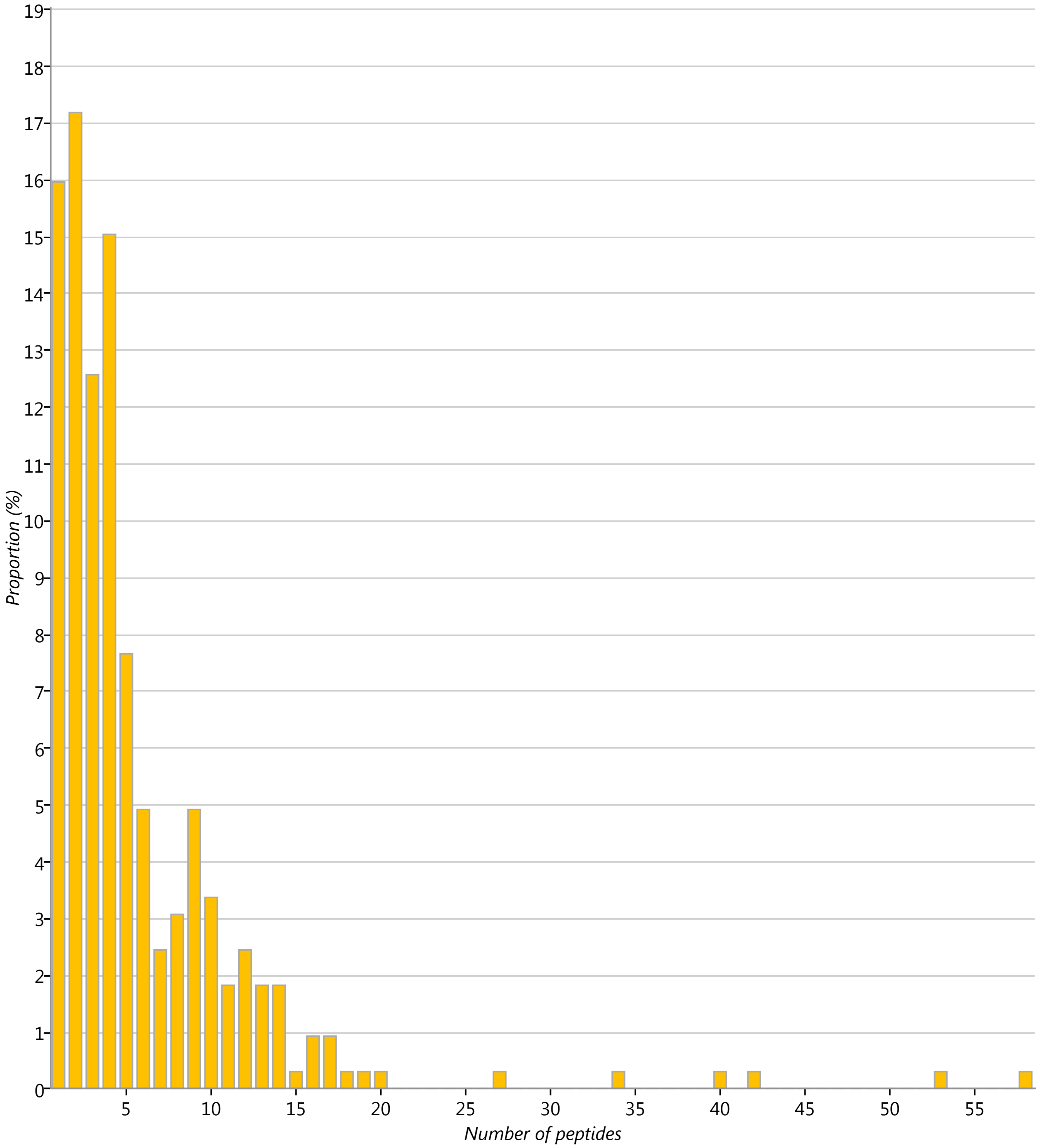

Modifications

The chart below shows the mean number of modifications per peptide ion for runs in the selected experiment design, based on each peptide ion's highest scoring identification. Each run is coloured according to its experimental condition.

Potential problems:

- Unexpectedly low numbers of modifications may indicate that one or more modifications were mistakenly not selected when searching for identifications.
- Variable numbers of modifications across the runs, especially within a given condition, may indicate problems with sample preparation.

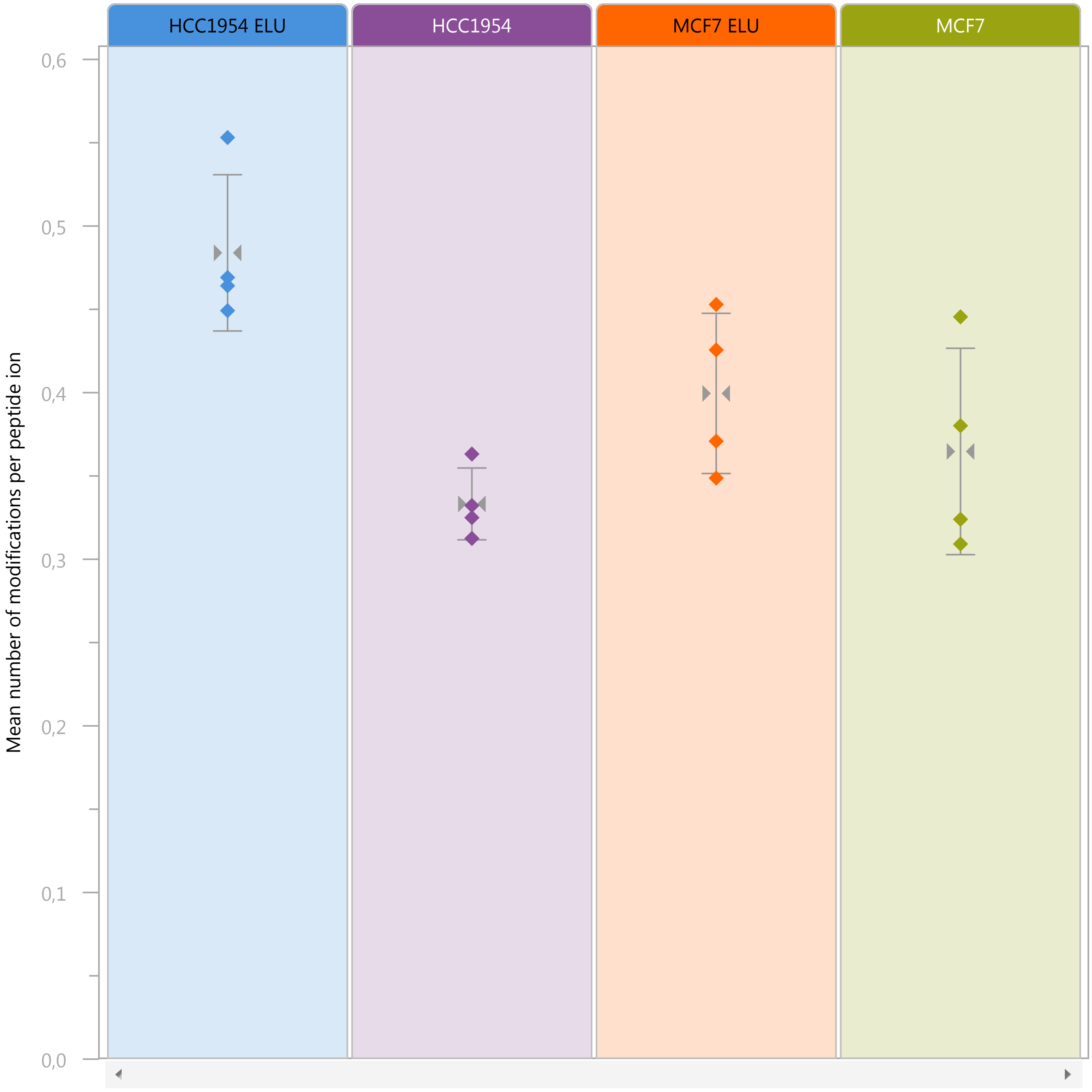

Included modifications Acetyl N-TERM Carbamidomethyl C Carbamyl N-TERM Deamidation N Dean

Missed cleavages

The chart below shows the mean number of missed cleavages per peptide ion for runs within the selected experiment design, based on each peptide ion's highest scoring identification. The chart assumes the use of trypsin for digestion. Each run is coloured according to its experimental condition.

Potential problems:

- A high number of missed cleavages may indicate problems in trypsinization.
- An absence of missed cleavages may indicate that database search parameters were too stringent.

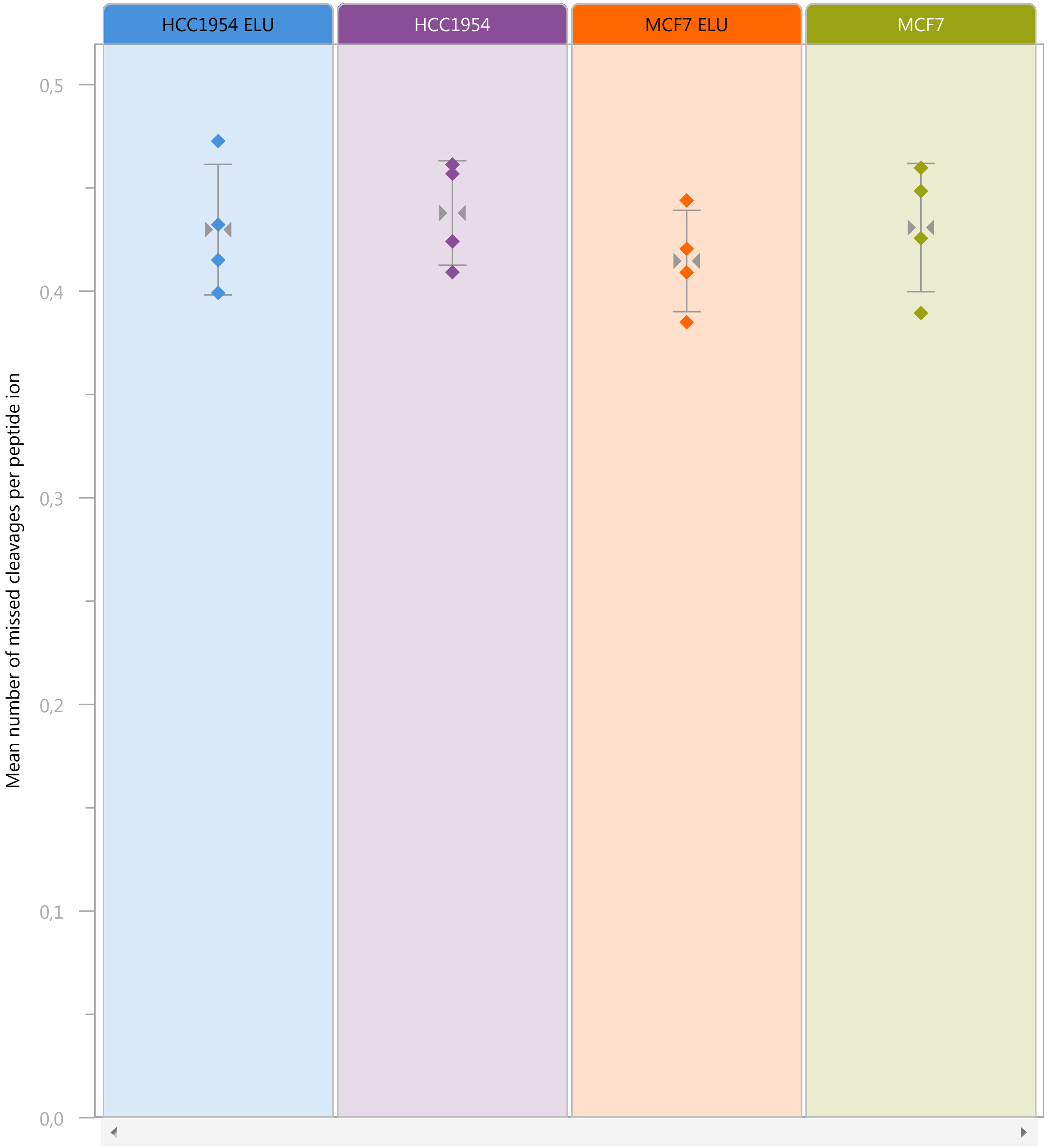

Abundance dynamic range

The charts below show abundance histograms for peptide ions, calculated using the current experiment design. Each chart shows the mean abundance for peptide ions within an experimental condition's runs. Data are binned by Log10 of their normalised abundance and shown as a proportion of the total. Identified peptide ions are shown in yellow.

Potential problems:

- A heavily right-skewed histogram of *identified* peptide ions indicates you may be missing identifications with low abundance.
- A heavily right skewed total (summed identified and unidentified) histogram may indicate saturation at high abundance, which will affect the accuracy of quantitation.

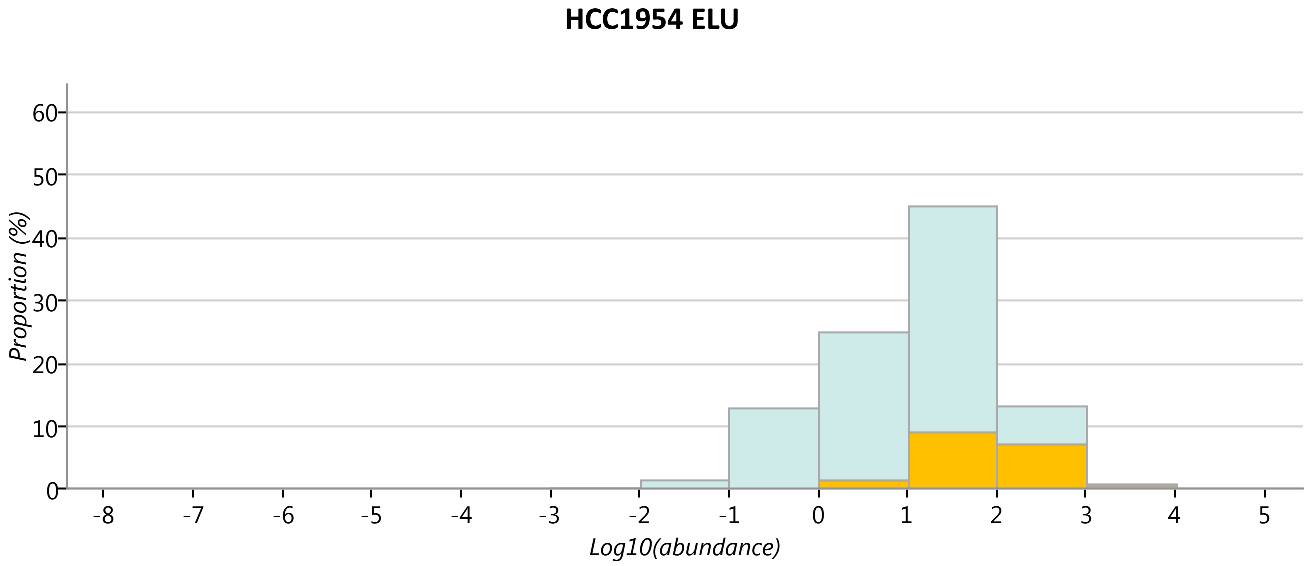

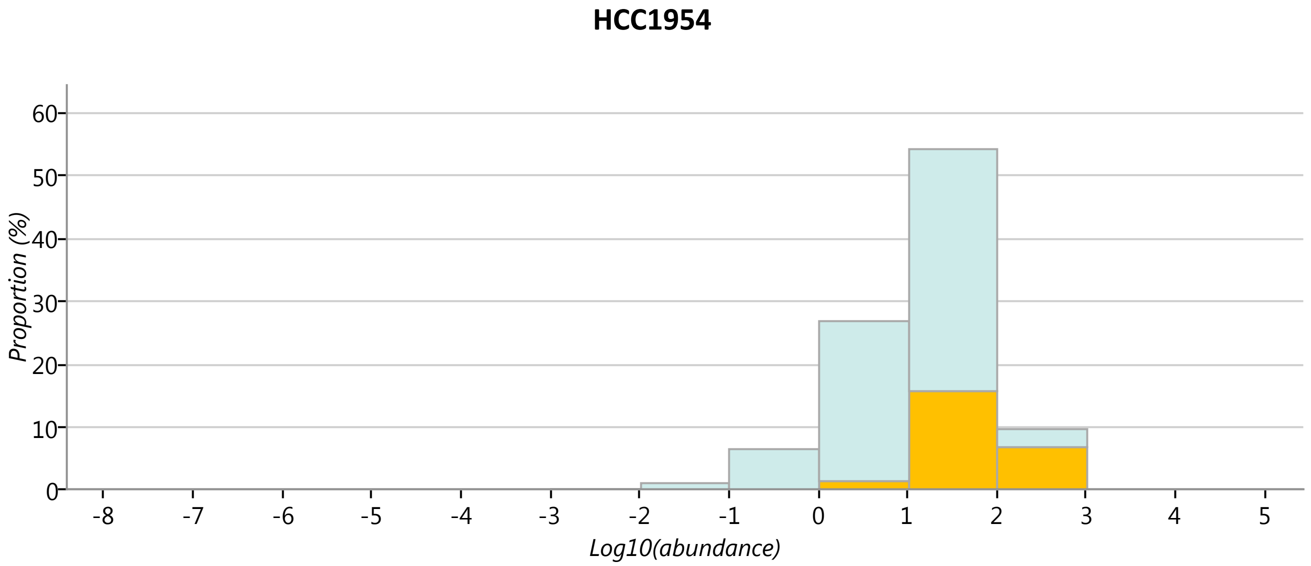

Not identified ■ Identified

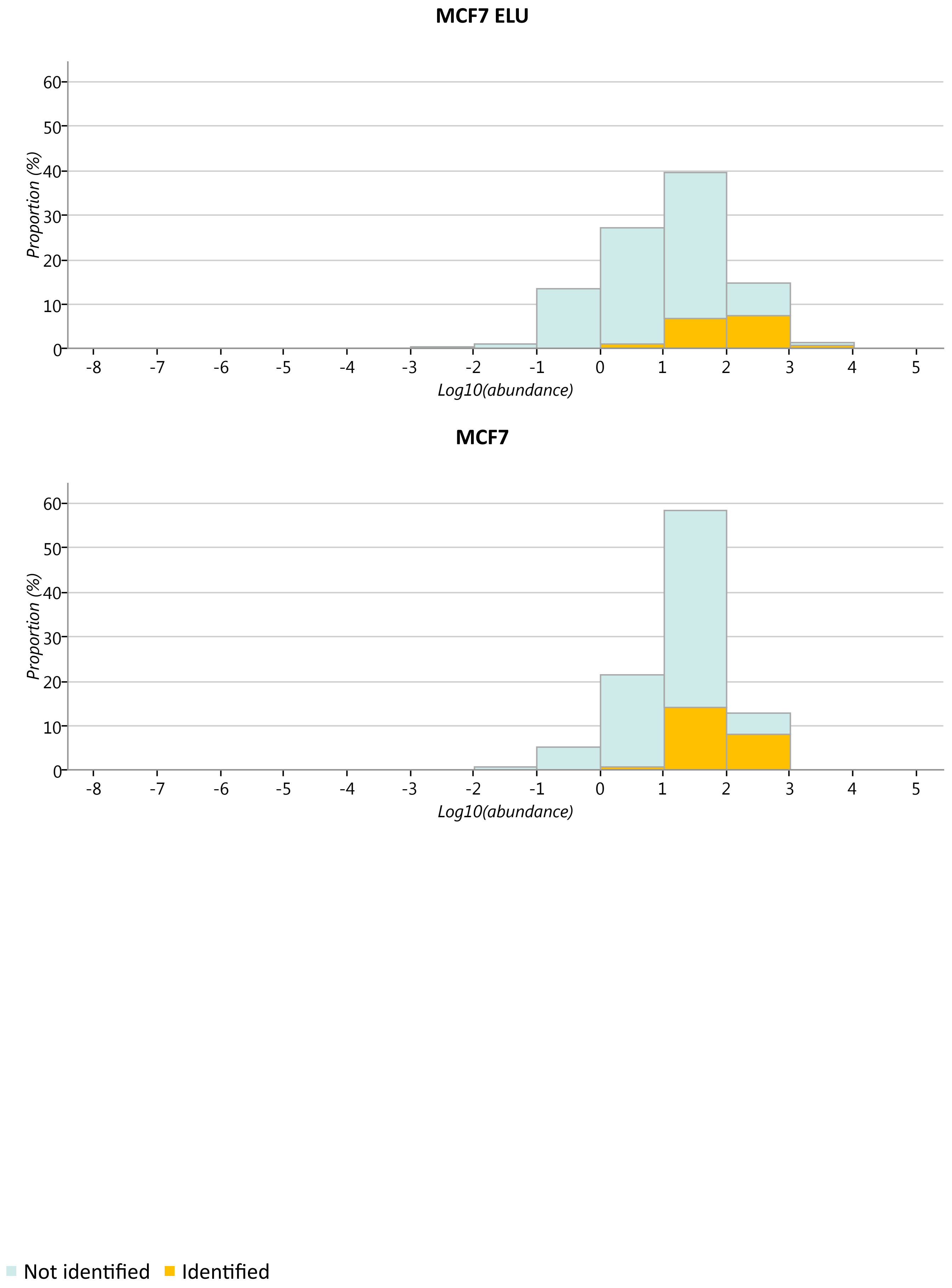
MCF7 ELU
Proportion (%)
0
10
20
30
40
50
60
-8
-7
-6
-5
-4
-3
-2
-1
0
1
2
3
4
5
Log10(abundance)
MCF7
Proportion (%)
0
10
20
30
40
50
60
-8
-7
-6
-5
-4
-3
-2
-1
0
1
2
3
4
5
Log10(abundance)
Not identified
Identified

Mass accuracy

The chart below shows all identified peptide ions in your experiment, plotting peptide ion m/z against the ppm error in its highest scoring identification. Blue points indicate peptide ions which have a mass error within 2 standard deviations of the mean, yellow points indicate peptide ions with a mass error more than 2 standard deviations from the mean.

Potential problems:

- A dependence of ppm mass error on m/z may indicate poor calibration.
- If the standard deviation of the data is large in ppm terms, this may indicate that your search parameters are too lenient.

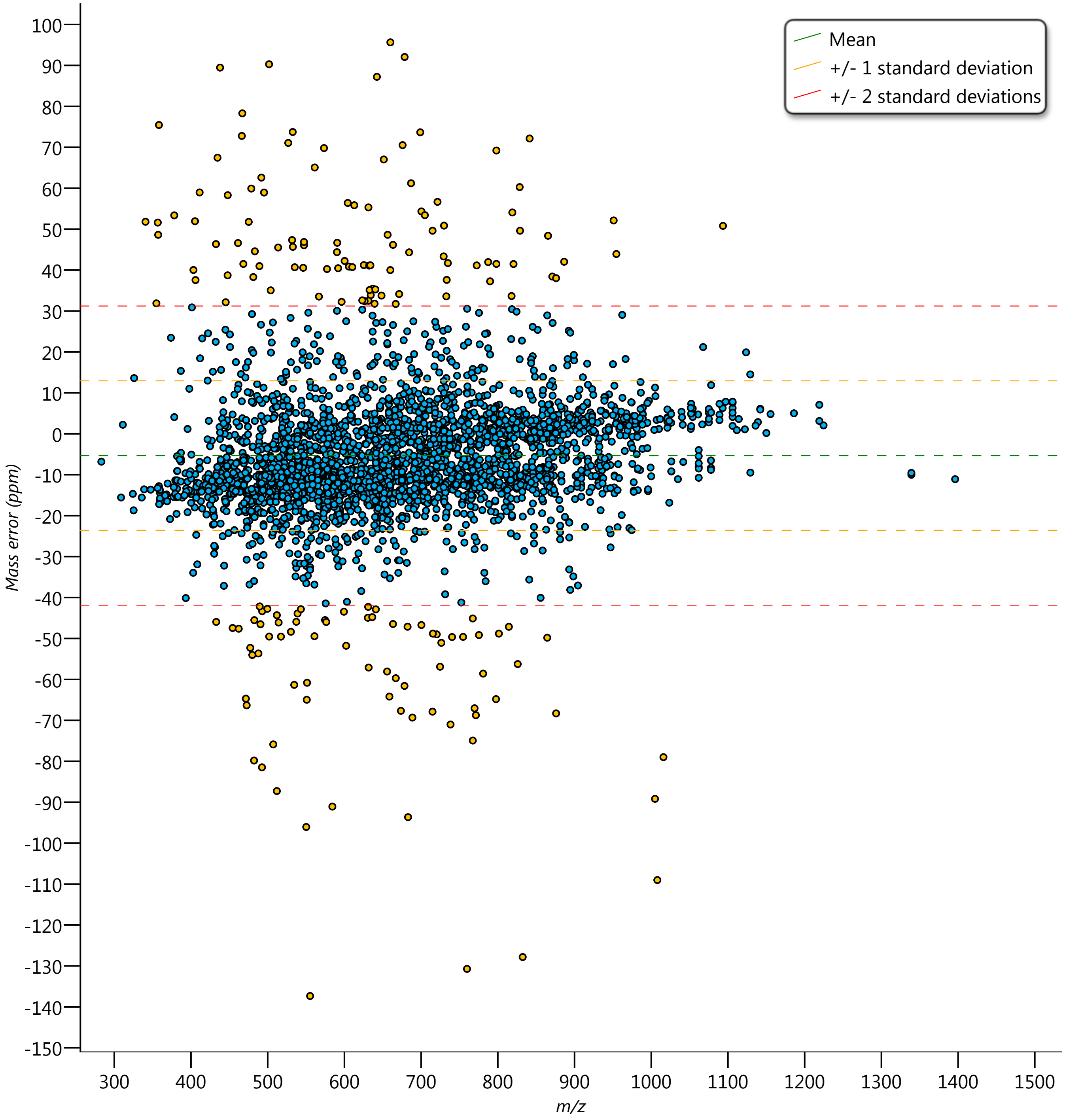

Scan rates

The charts below show the MS1 scan rate over the duration of your runs, using the current experiment design. Each chart shows the scan rate variation within an experimental condition's runs. The named run is highlighted in black. The variability between runs is shown as a grey area. The y-axis shows scans per second in a 30-second window centred on each x-axis time point.

Potential problems:

- A highly varying scan rate means quantitation accuracy may be negatively affected.

HCC1954 ELU

Run: 20140724_DAP_HCC1954_ELUIDO_2ug_inj5_STEP2

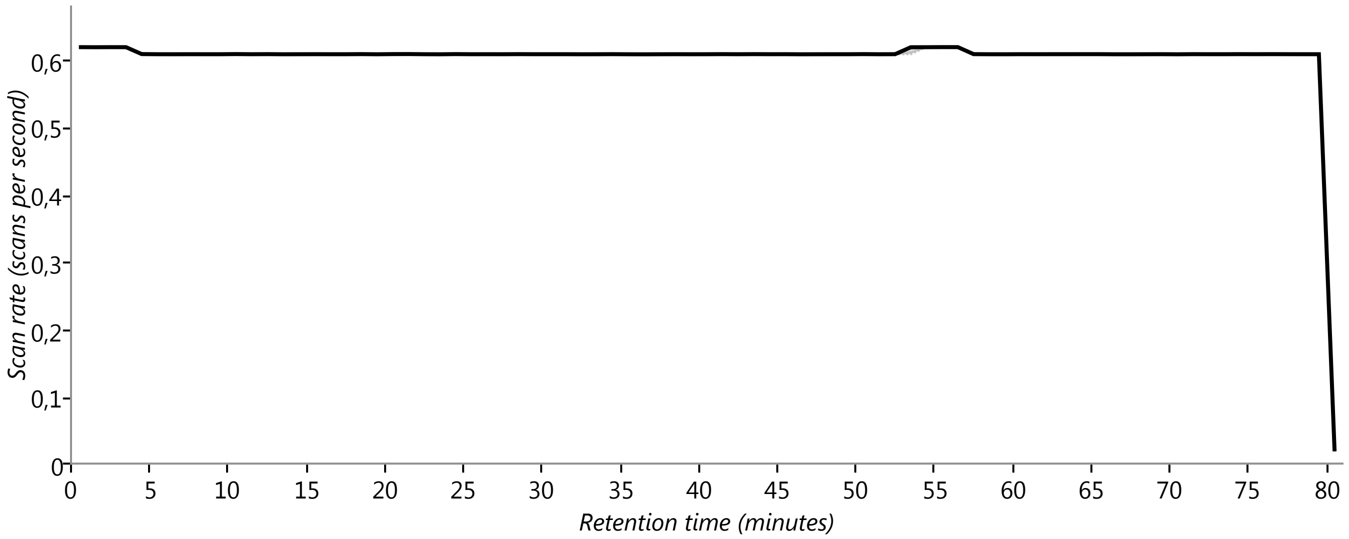

HCC1954

Run: 20140801_DAP_HCC1954_NAO_RETIDO_2ug_inj2_STEP2

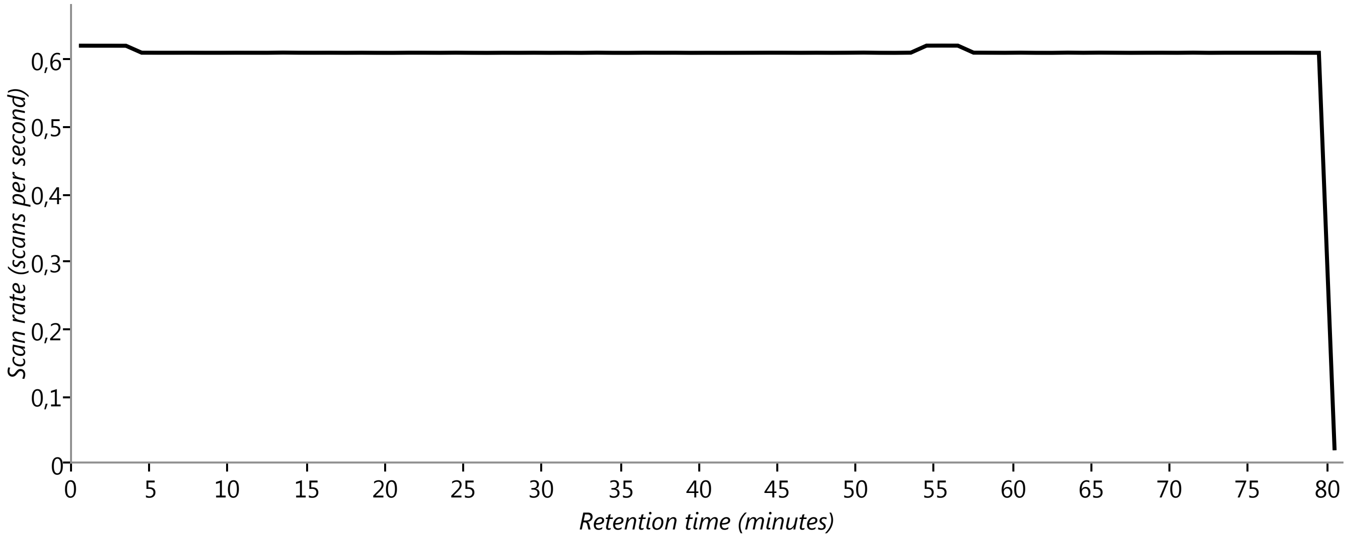

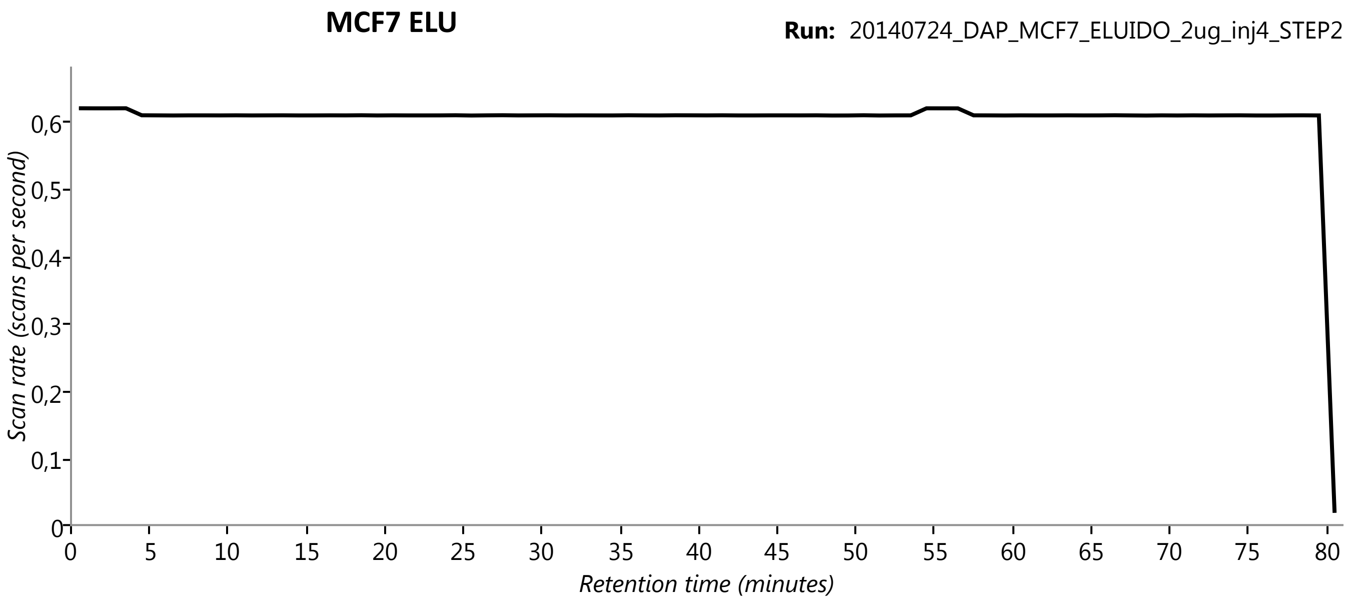
MCF7 ELU
Run: 20140724_DAP_MCF7_ELUIDO_2ug_inj4_STEP2
Scan rate (scans per second)
0,6
0,5
0,4
0,3
0,2
0,1
0
0 5 10 15 20 25 30 35 40 45 50 55 60 65 70 75 80
Retention time (minutes)

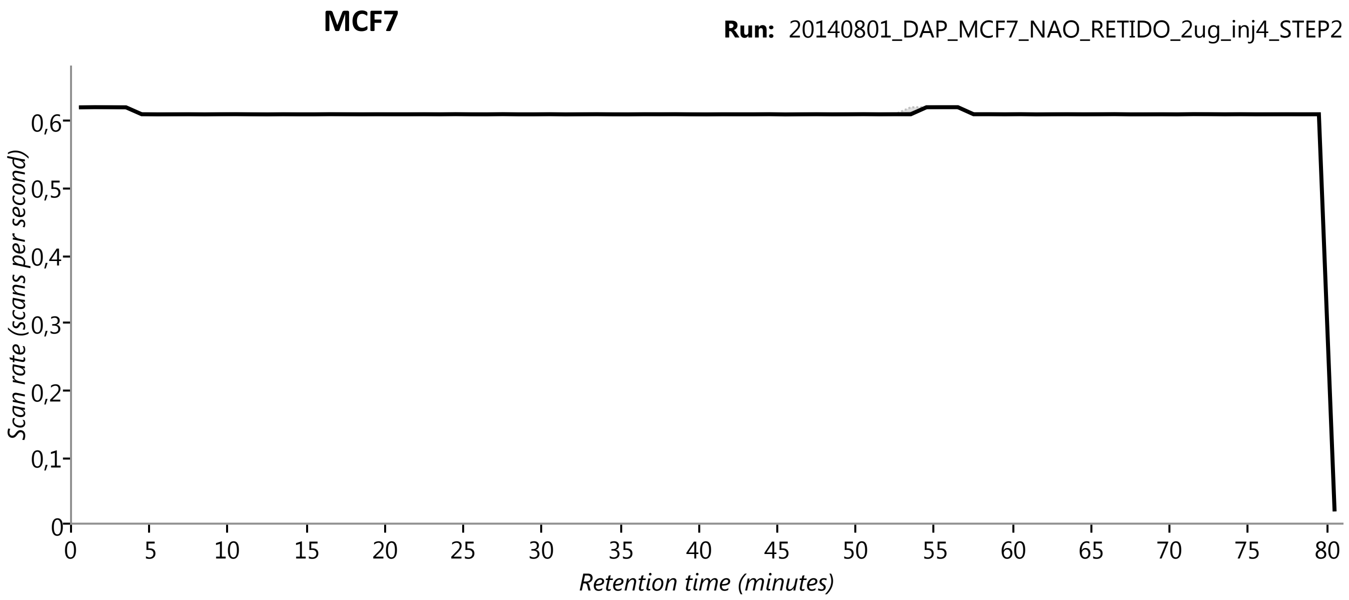
MCF7
Run: 20140801_DAP_MCF7_NAO_RETIDO_2ug_inj4_STEP2
Scan rate (scans per second)
0,6
0,5
0,4
0,3
0,2
0,1
0
0 5 10 15 20 25 30 35 40 45 50 55 60 65 70 75 80
Retention time (minutes)

Proteins

The chart below shows the number of proteins identified in each of the runs within the selected experiment design. Each run is coloured according to its experimental condition.

Potential problems:

- Any exceptionally high or low values may indicate problems with sample preparation, chromatography, or instrument settings.

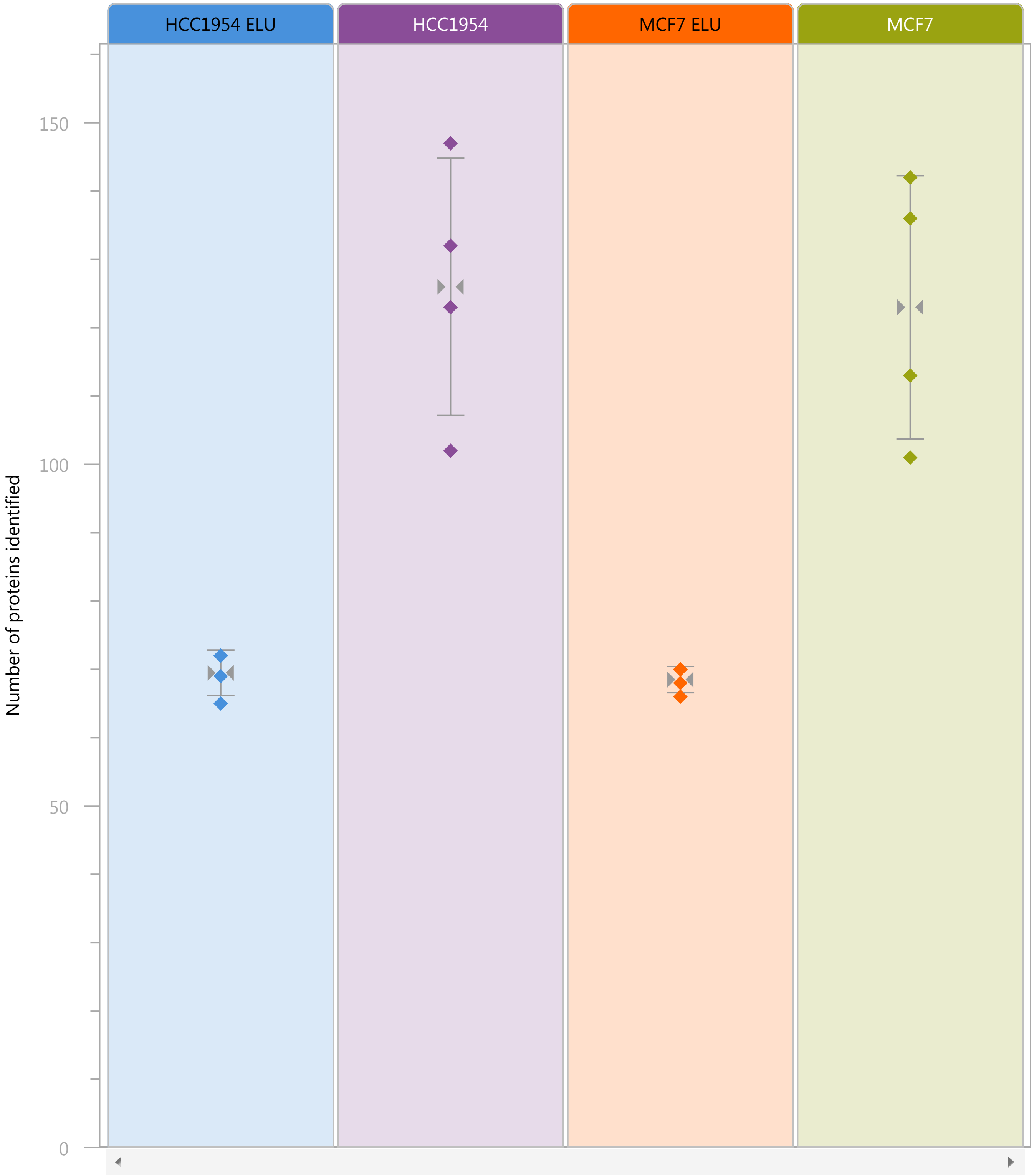

Peptides

The chart below shows the number of peptides identified in each of the experiment's runs. Each run is coloured according to its experimental condition.

Potential problems:

- Any exceptionally high or low values may indicate problems with sample preparation, chromatography, or instrument settings.

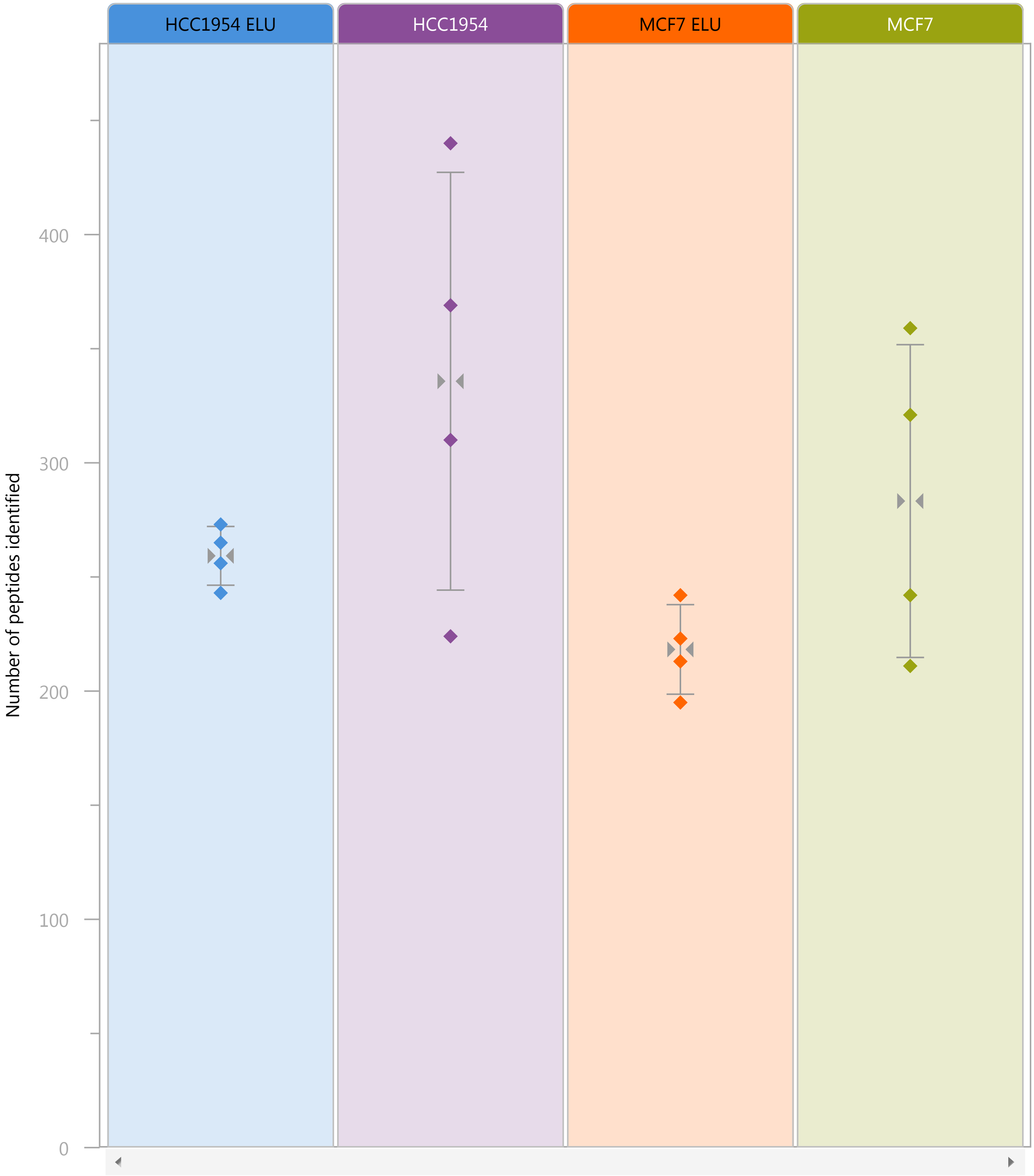

Peptides per protein (by condition)

The chart below shows the average number of peptides per protein in each run within the selected experiment design. For a given run, the value shown is calculated using only identifications from that run.

Potential problems:

- Any exceptionally high or low values may indicate problems with sample preparation or peptide search settings.

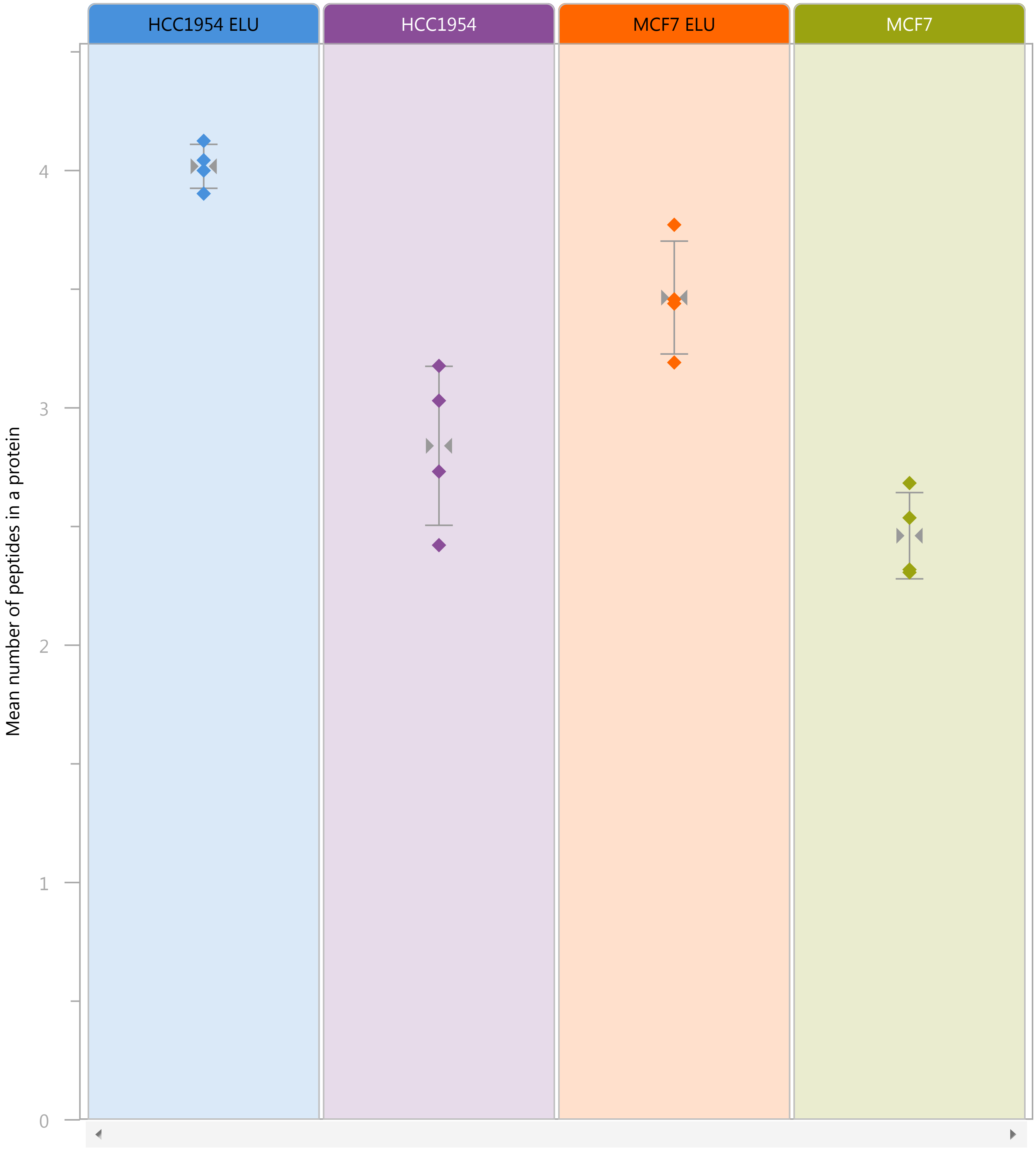

Percentage of peptide ions identified

The chart below shows the number of peptide ions identified in each of the runs within the selected experiment design, expressed as a percentage of the total number of peptide ions. Each run is coloured according to its experimental condition.

Potential problems:

- Any exceptionally high or low values may indicate problems with sample preparation, chromatography, or instrument settings.
- Very low values across all runs may indicate a problem with the database that was searched for identifications, or with other search parameters.

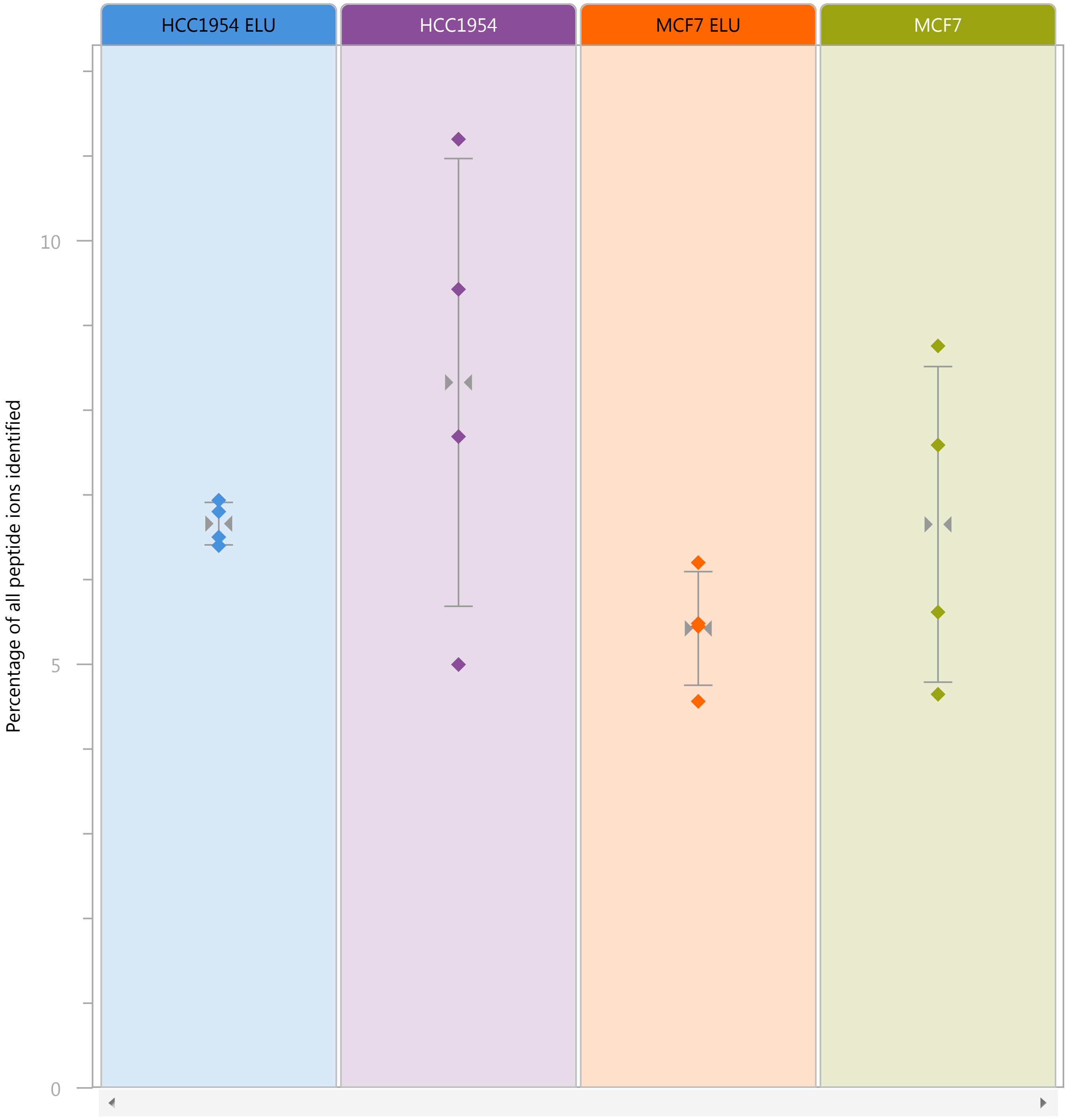

Protein overlap

The Venn diagram shows the number of proteins identified uniquely in the selected conditions and the number that are shared between conditions. Use the list on the right to select two or three conditions to explore.

Select experimental conditions

- HCC1954 ELU
- HCC1954
- MCF7 ELU

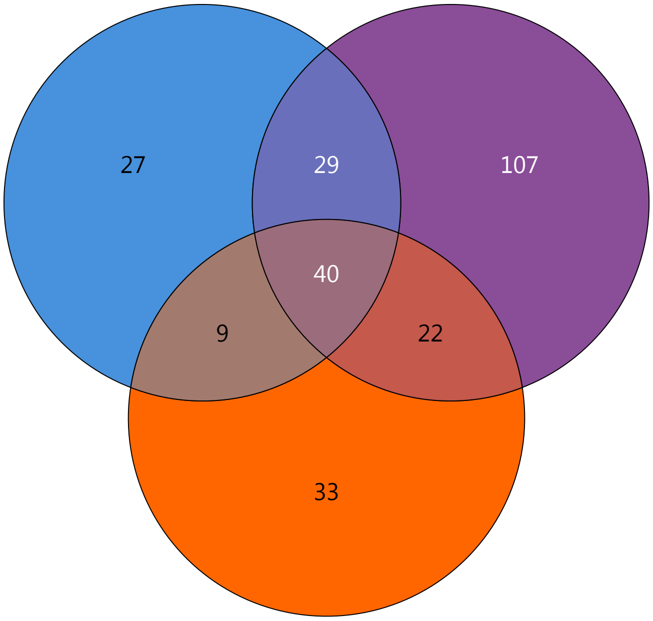

HCC1954 X MCF7 STEP3

QC Metrics reported by Progenesis QI for proteomics v2.0.5556.29015
Date: 10-Jan-2020 at 09:24
Experiment design: HCC1954 x MCF7 step3

This QC report contains **all metrics** for the experiment named above.

Reported metrics

0 of the 19 metrics reported have been manually flagged for attention.

- Identifications overview
- LC peak width
- Peptide ion dynamic range
- Precursor m/z
- Precursor retention time
- Mass errors
- Precursor charges
- Missed cleavages (assuming trypsin)
- Peptides per protein (overview)
- Modifications
- Missed cleavages
- Abundance dynamic range
- Mass accuracy
- Scan rates
- Proteins
- Peptides
- Peptides per protein (by condition)
- Percentage of peptide ions identified
- Protein overlap

Identifications overview

The chart below provides the number of identifications per run by category. Data are plotted for all runs in the current experiment design. The box represents the interquartile range and the whiskers the maximum and minimum.

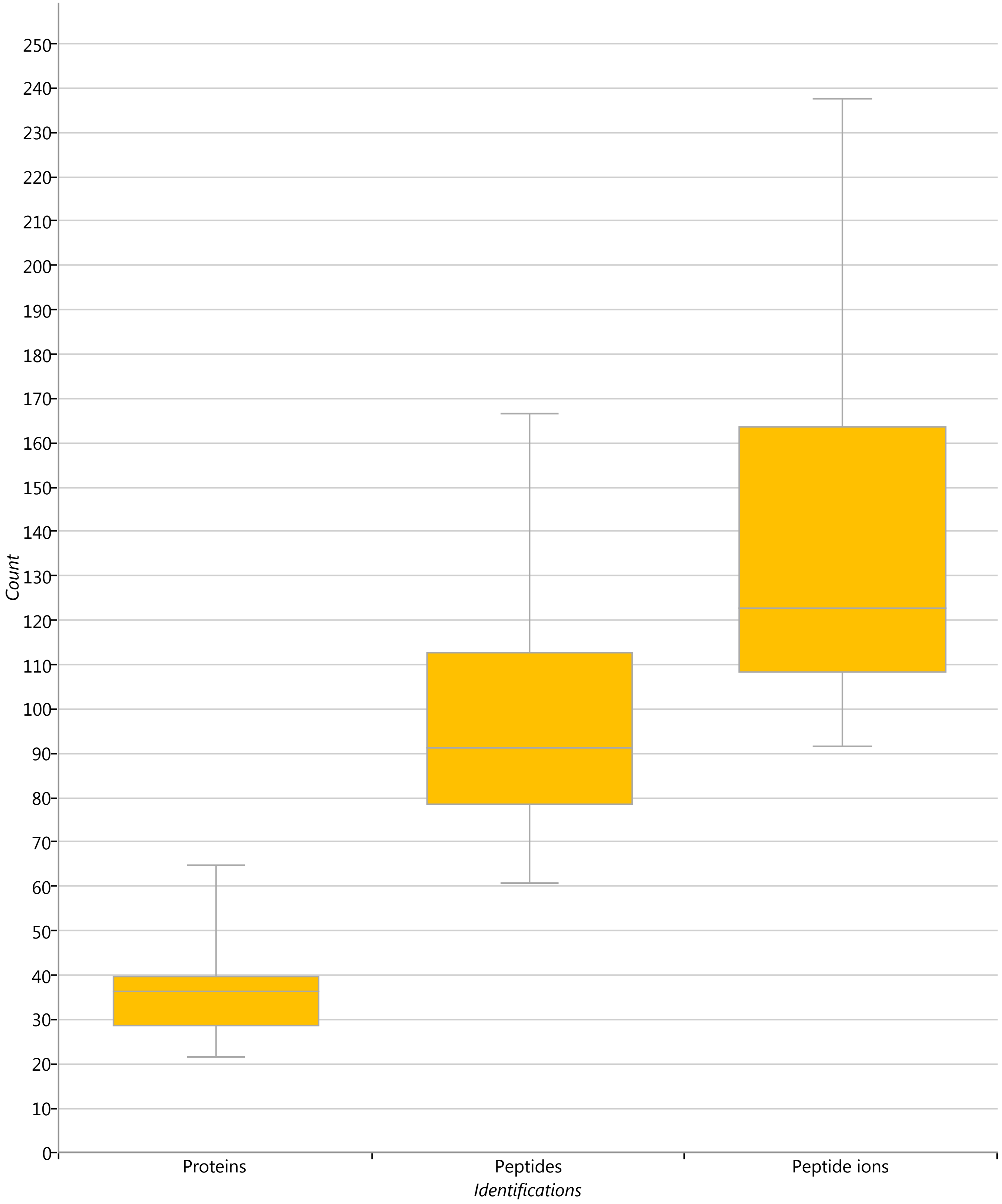

LC peak width

This chart shows the proportional distribution of peak widths in retention time, for all the detected peptide ions in the experiment. Widths are binned into categories for simple comparison. Identified peptide ions are shown in yellow.

Potential problems:

- A peak width that is different from that expected for your setup may indicate a problem with the chromatographic separation.

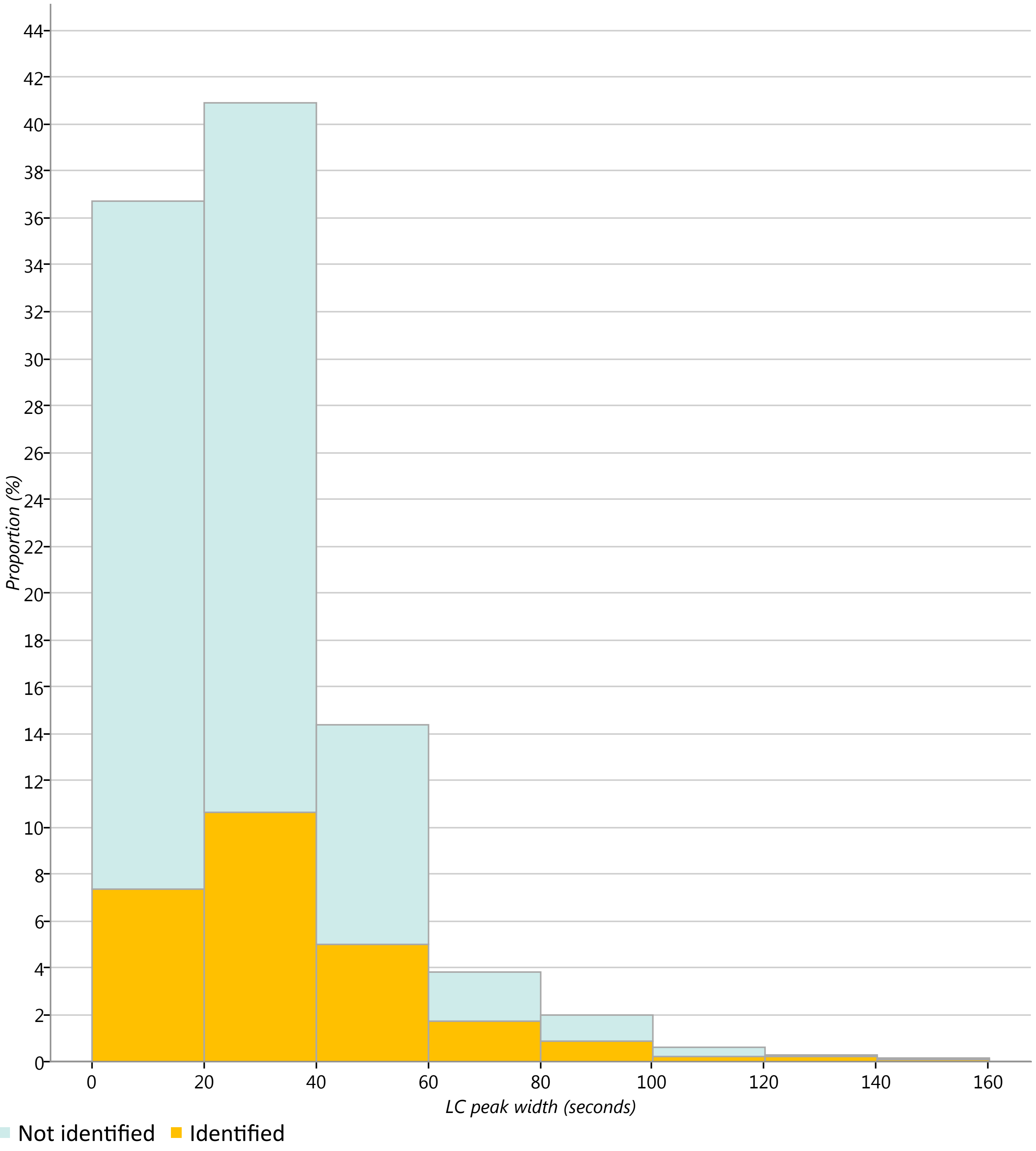

Peptide ion dynamic range

This chart shows log abundance for peptide ions, calculated using the current experiment design. Each peptide ion is assigned its abundance based on the highest mean value for any condition's runs. Data are binned and shown as a proportion. Identified peptide ions are shown in yellow.

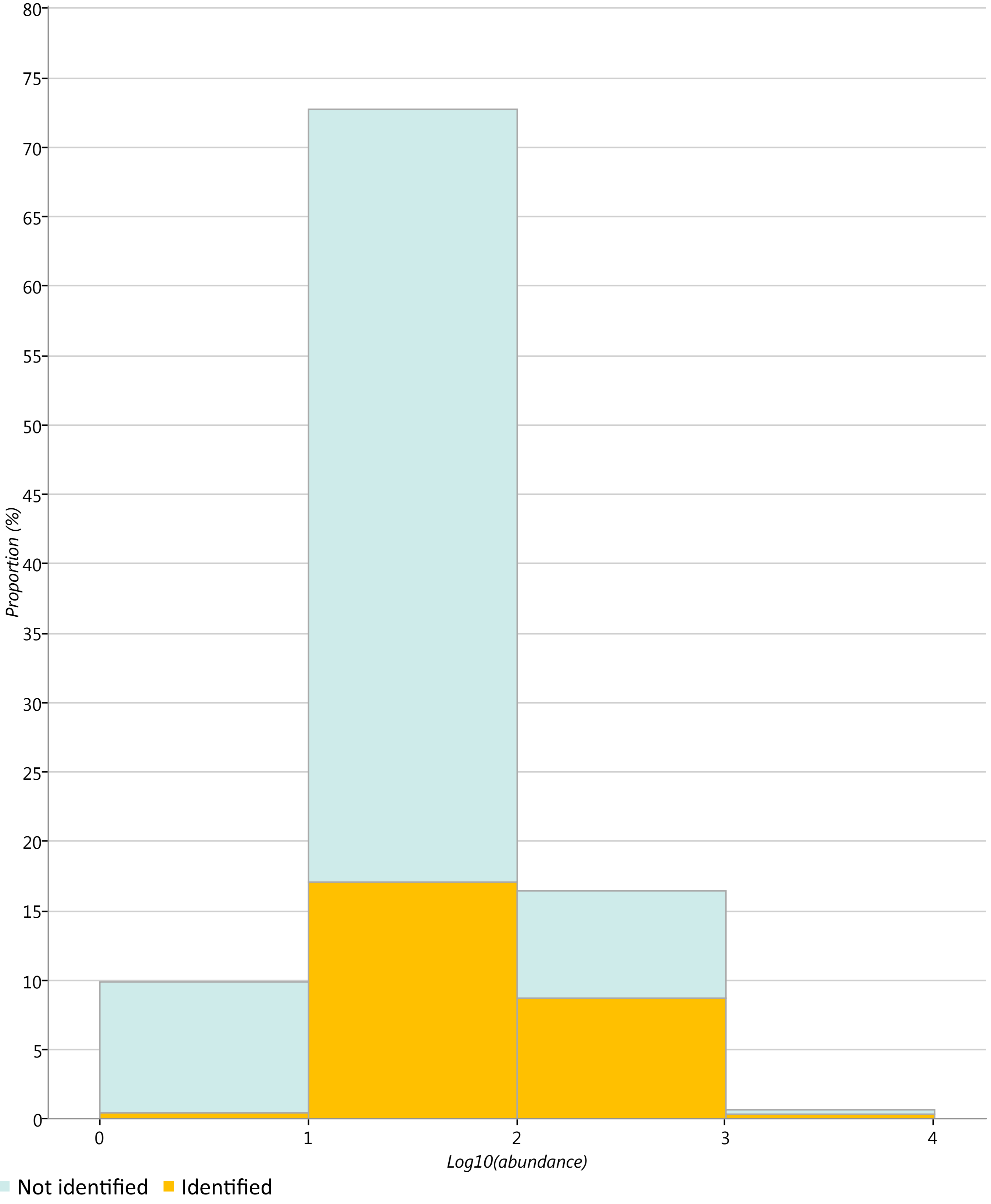

Precursor m/z

This chart shows the distribution of m/z values for all peptide ions in the experiment. Identified peptide ions are shown in yellow.

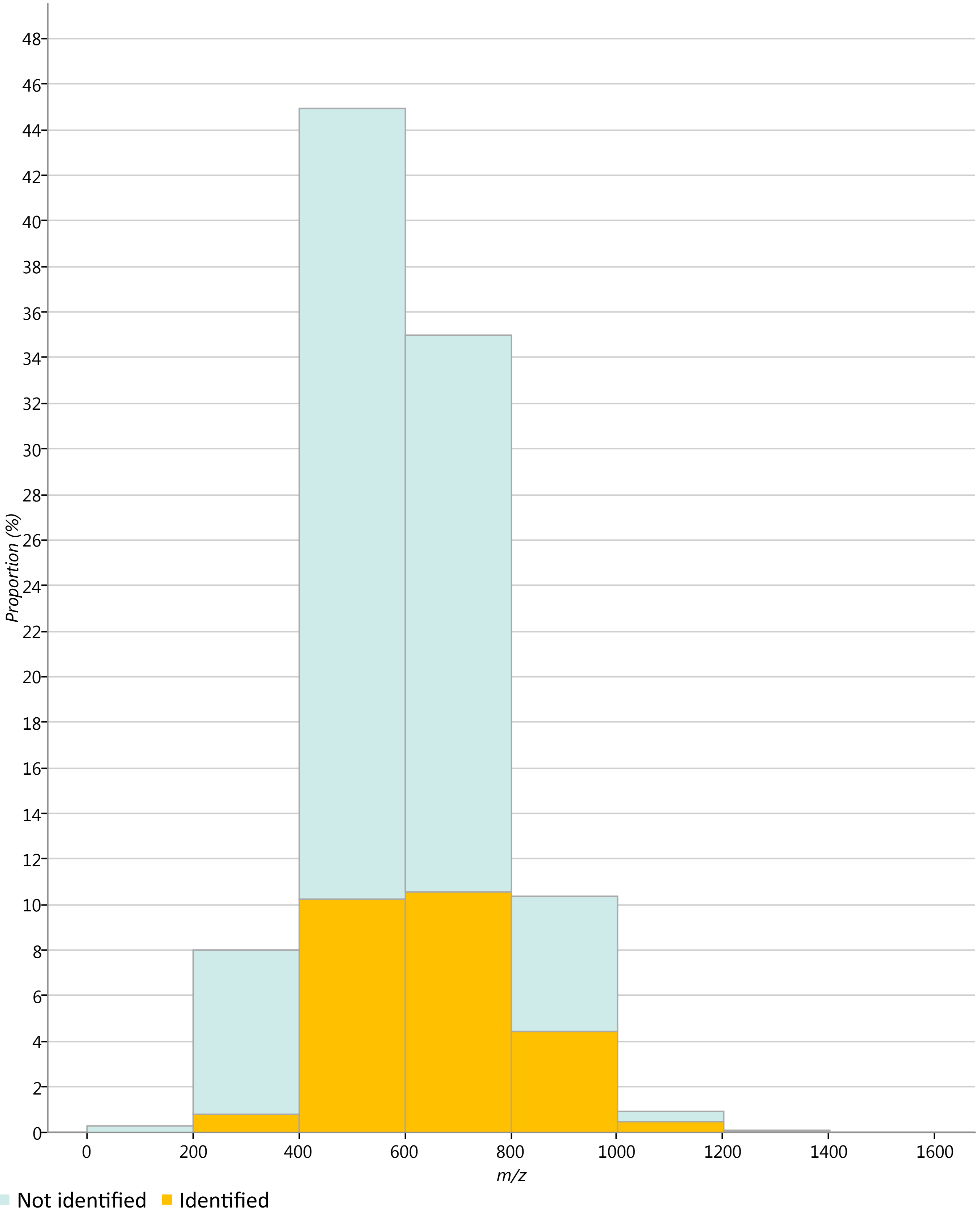

Precursor retention time

This chart shows the proportion of precursor peptide ions eluting into each quartile of the LC run. Identified peptide ions are shown in yellow.

Potential problems:

- A majority of peptide ions eluting at the start or end of the run may indicate problems with the chromatographic separation or that the setup is not ideal for a successful separation.

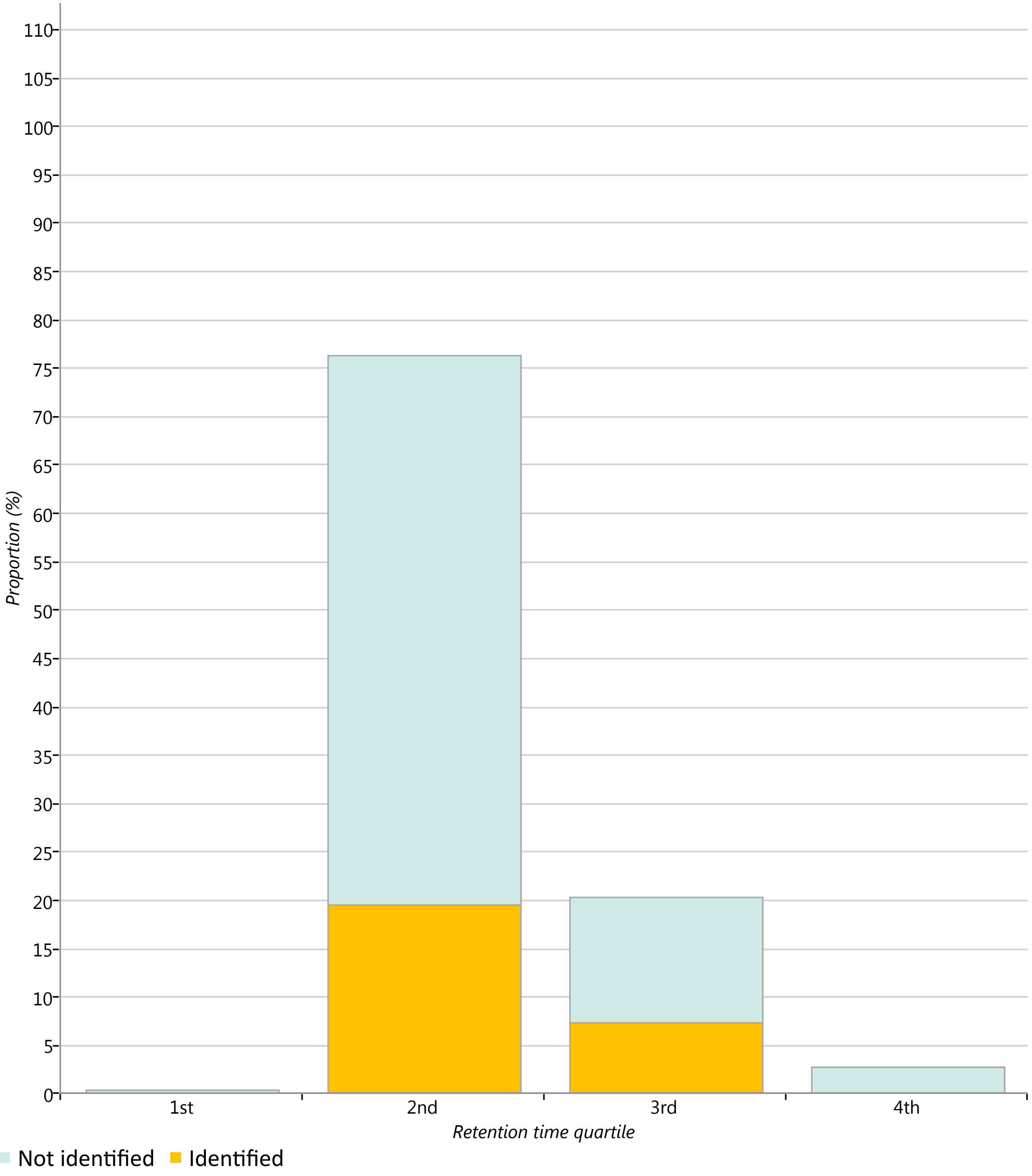

Mass errors

This chart shows the mass error calculated for identified peptide ions. The proportion of peptide ions with a given binned error value is plotted. For a more detailed plot, see the *Mass accuracy* metric.

Potential problems:

- A larger than expected spread could indicate that search parameters are too lenient.
- A bias of the peak from zero ppm could indicate instrument bias.

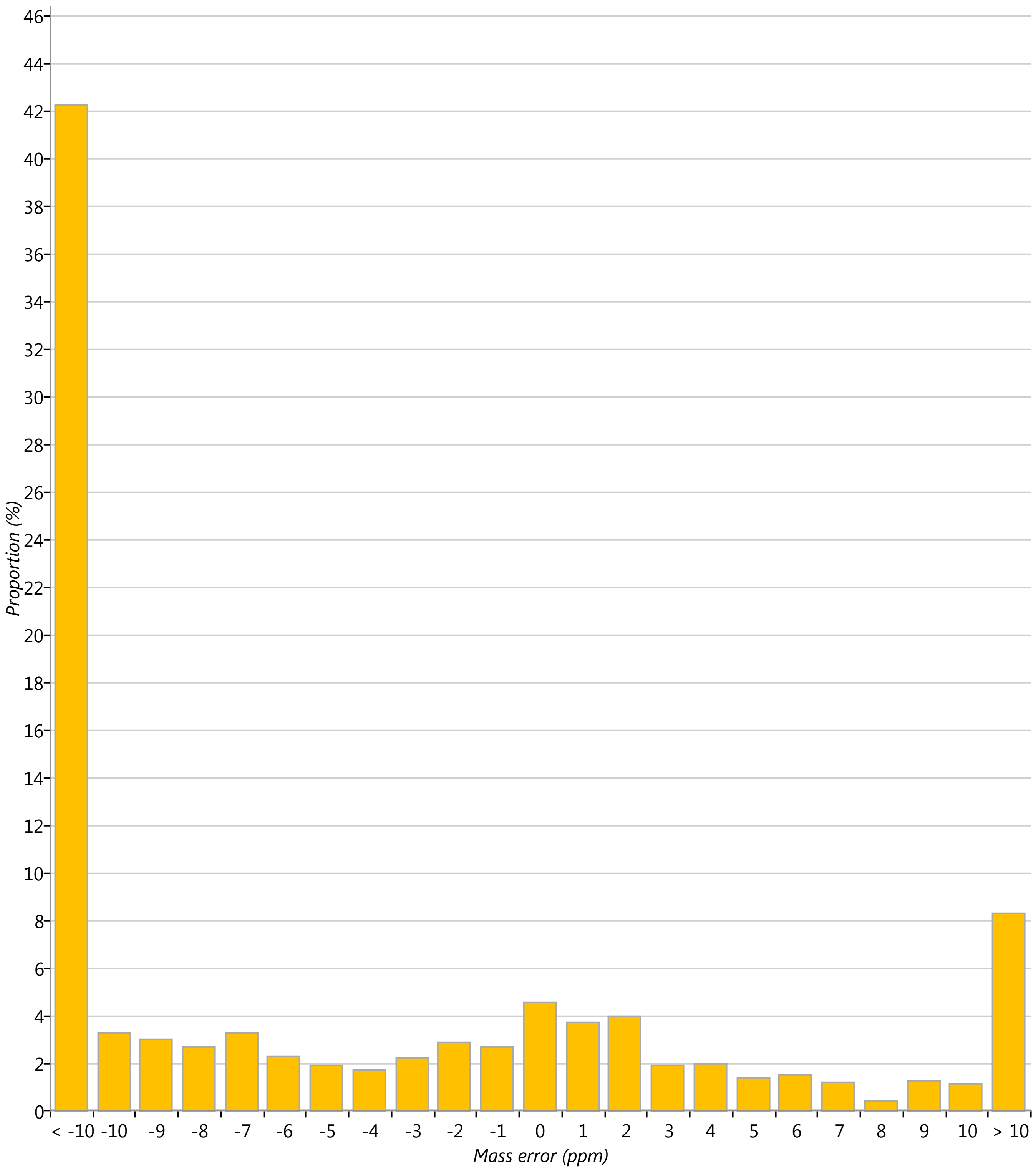

Precursor charges

This chart shows the percentage of peptide ions with various charge states.

Potential problems:

- A skew towards low charge peptide ions may indicate poor ionisation, suggesting that you may need to raise the collision voltage.
- A skew towards high charge peptide ions may indicate excessive ionisation, suggesting that you may need to lower the collision voltage.

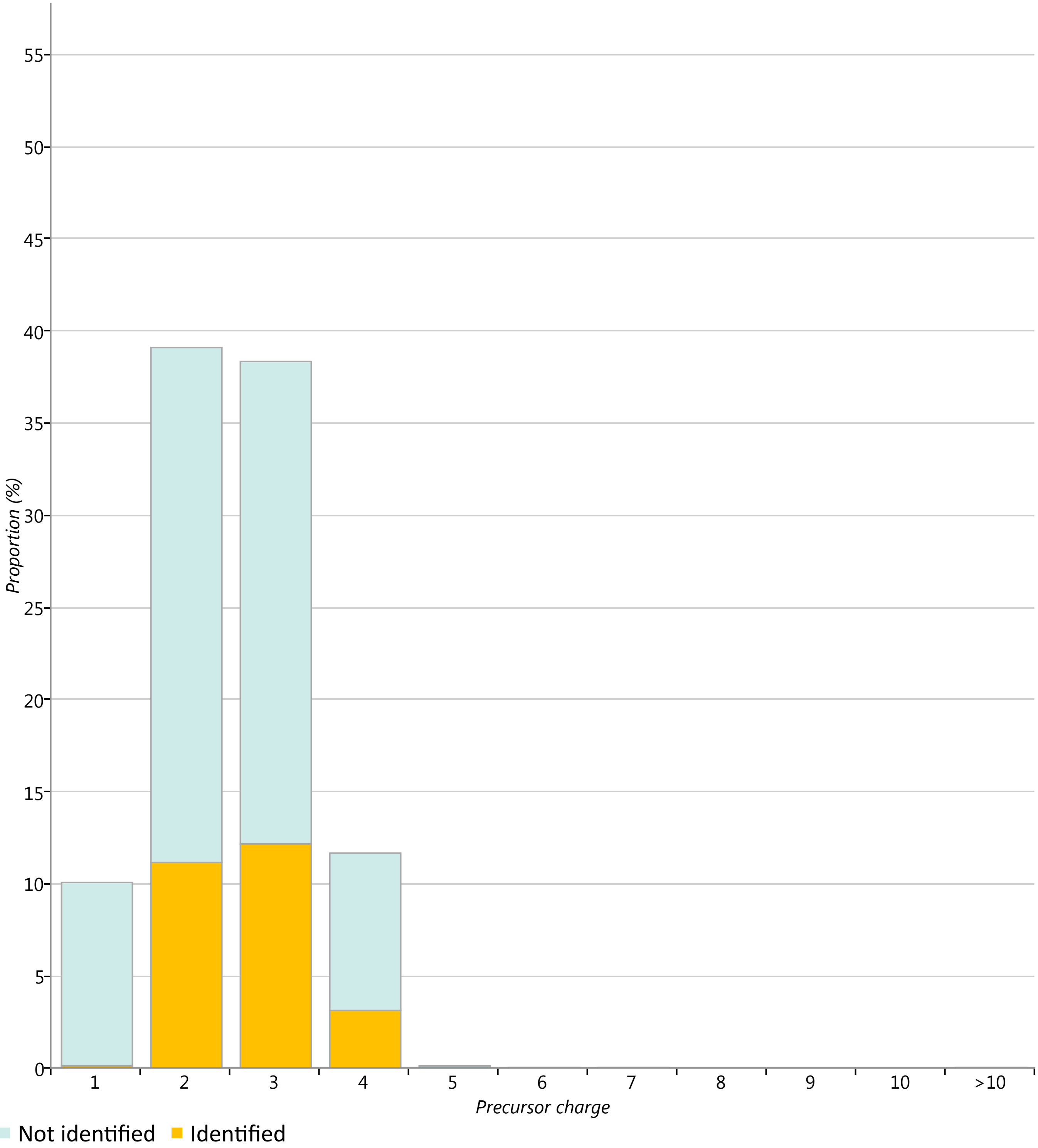

Missed cleavages (assuming trypsin)

This chart shows the distribution of missed cleavages per identified peptide ion across the whole experiment, assuming the proteins were treated with trypsin and using each peptide ion's highest scoring identification. For an alternative visualisation showing the data subdivided by experimental condition, see the *Missed cleavages* metric.

Potential problems:

- A high proportion of missed cleavages may indicate problems in trypsinization.
- An absence of missed cleavages may indicate that the database search parameters were too stringent.

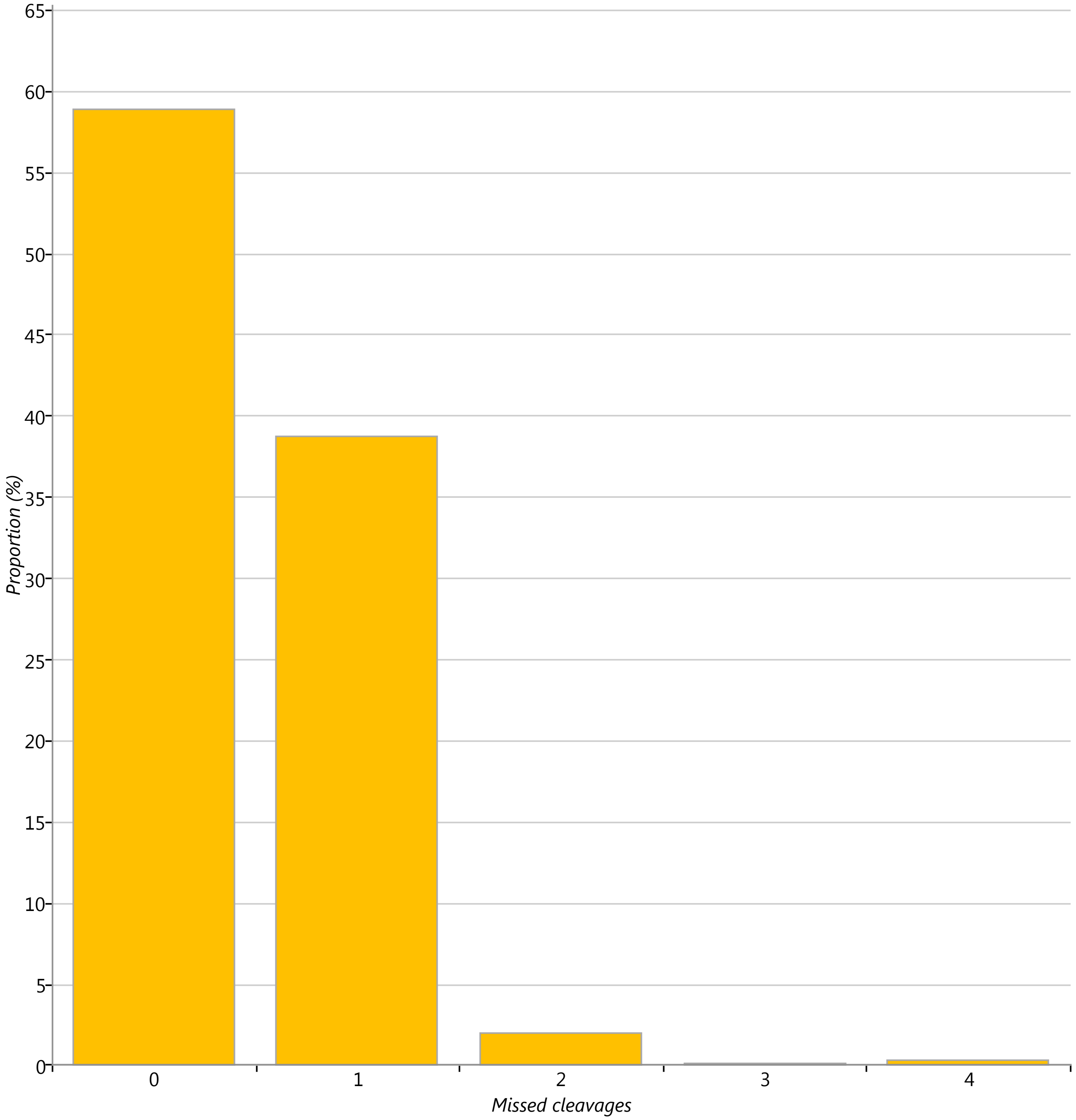

Peptides per protein (overview)

This chart shows the proportional distribution of the number of peptides identified per protein, combined across all runs in the experiment. For an alternative visualisation dividing these data by experimental condition, see the *Peptides per protein (by condition)* metric.

Potential problems:

- An unexpected bias to high or low numbers of peptides may indicate problems with sample preparation or peptide search settings.

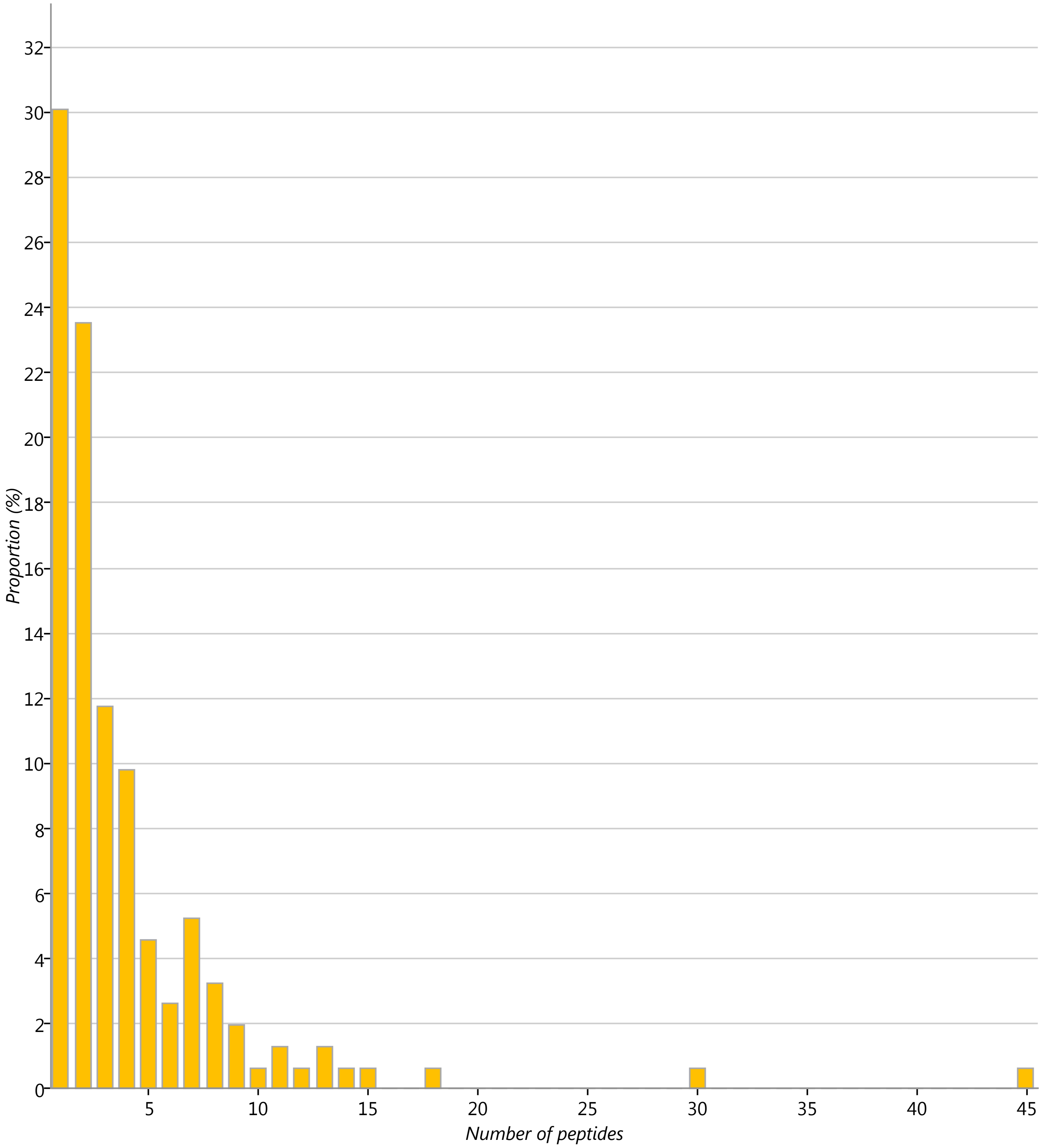

Modifications

The chart below shows the mean number of modifications per peptide ion for runs in the selected experiment design, based on each peptide ion's highest scoring identification. Each run is coloured according to its experimental condition.

Potential problems:

- Unexpectedly low numbers of modifications may indicate that one or more modifications were mistakenly not selected when searching for identifications.
- Variable numbers of modifications across the runs, especially within a given condition, may indicate problems with sample preparation.

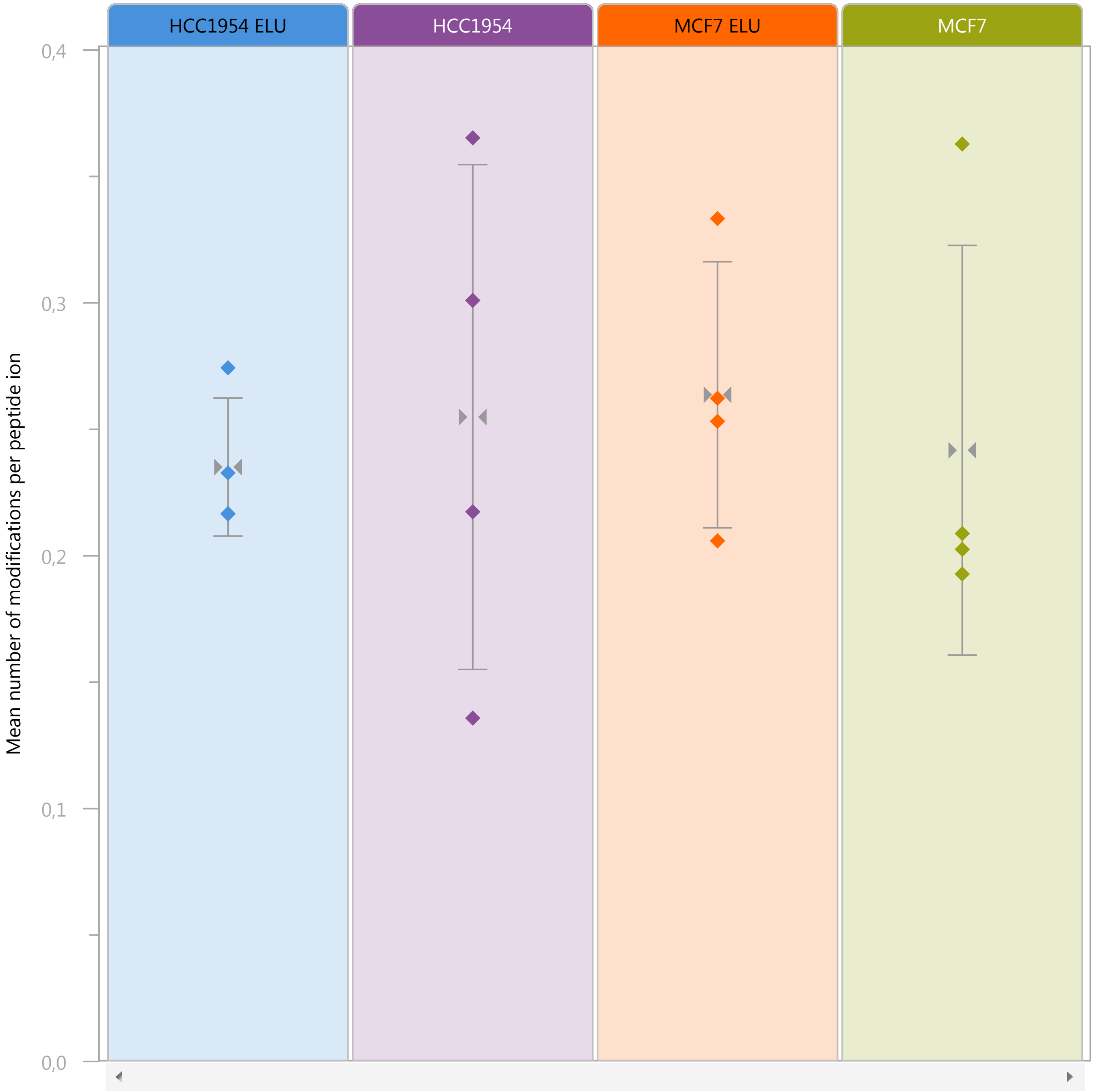

Included modifications Acetyl N-TERM Carbamidomethyl C Carbamyl N-TERM Deamidation N Dean

Missed cleavages

The chart below shows the mean number of missed cleavages per peptide ion for runs within the selected experiment design, based on each peptide ion's highest scoring identification. The chart assumes the use of trypsin for digestion. Each run is coloured according to its experimental condition.

Potential problems:

- A high number of missed cleavages may indicate problems in trypsinization.
- An absence of missed cleavages may indicate that database search parameters were too stringent.

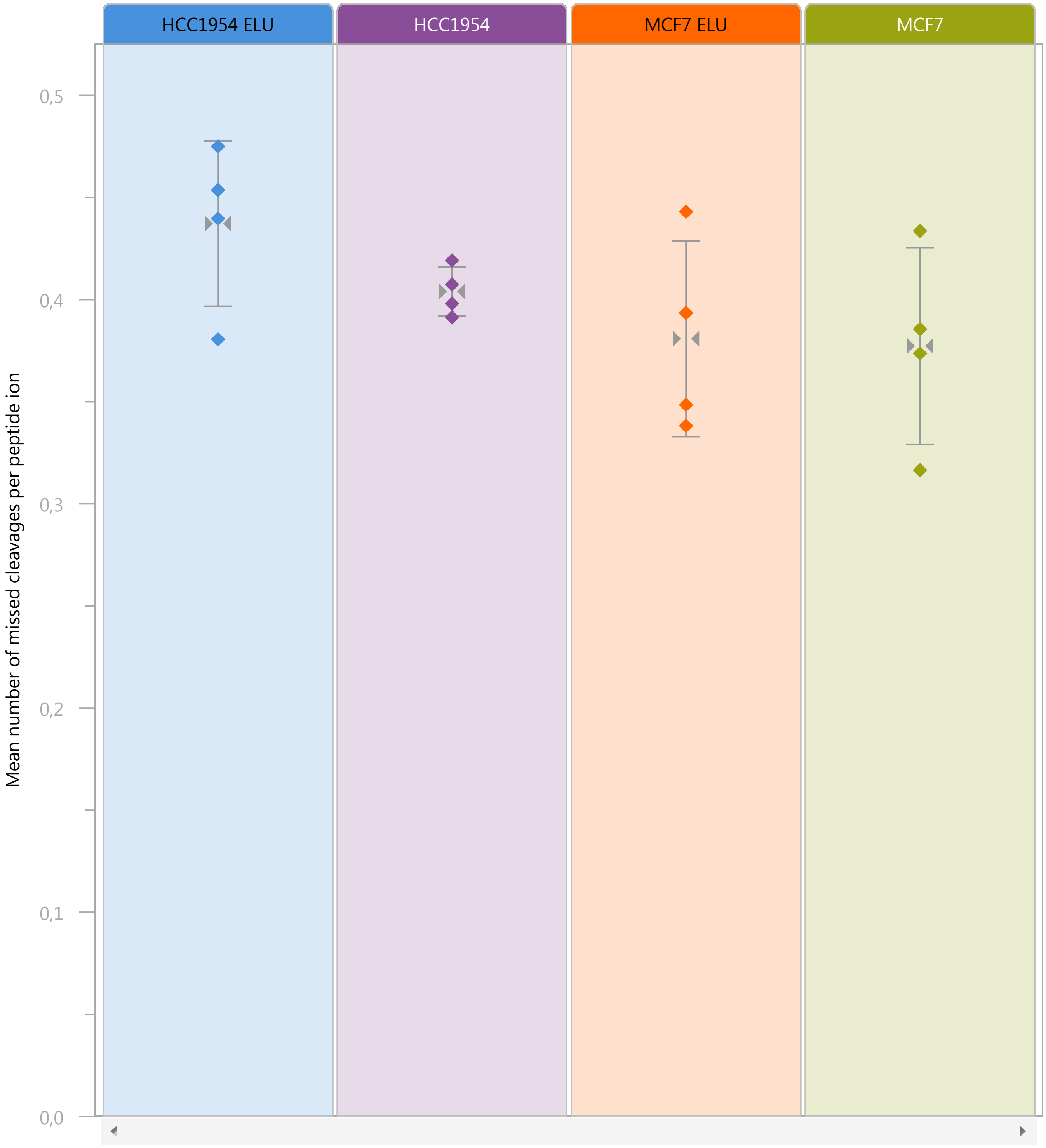

Abundance dynamic range

The charts below show abundance histograms for peptide ions, calculated using the current experiment design. Each chart shows the mean abundance for peptide ions within an experimental condition's runs. Data are binned by Log10 of their normalised abundance and shown as a proportion of the total. Identified peptide ions are shown in yellow.

Potential problems:

- A heavily right-skewed histogram of *identified* peptide ions indicates you may be missing identifications with low abundance.
- A heavily right skewed total (summed identified and unidentified) histogram may indicate saturation at high abundance, which will affect the accuracy of quantitation.

HCC1954 ELU

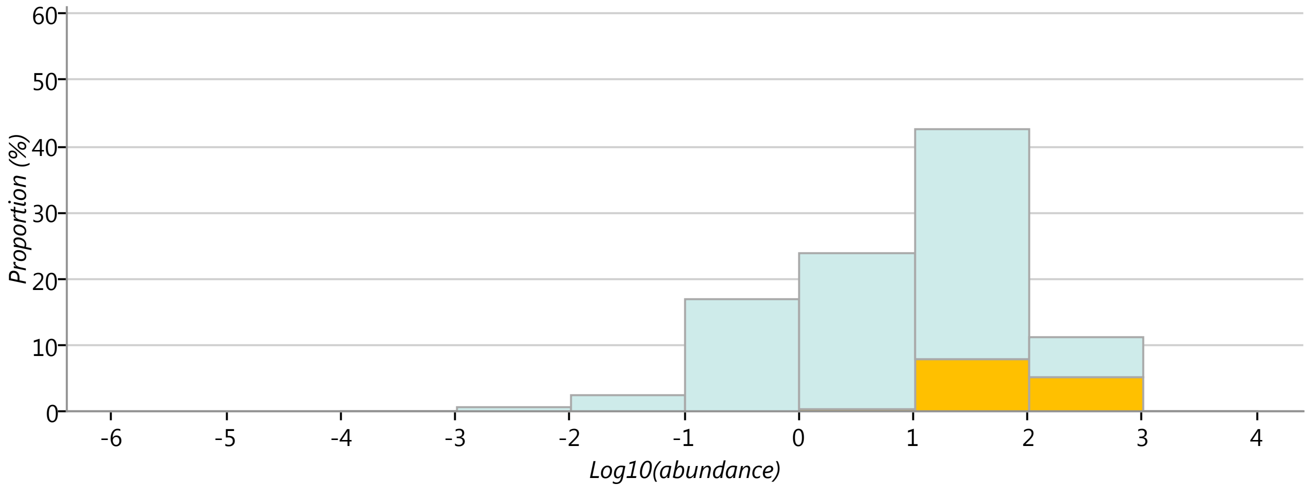

HCC1954

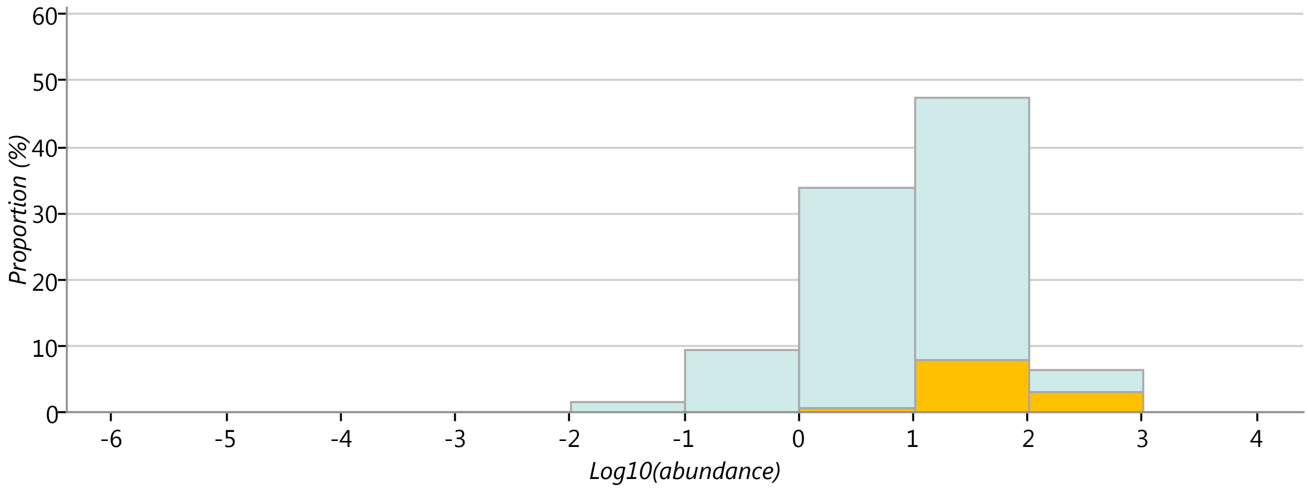

Not identified Identified

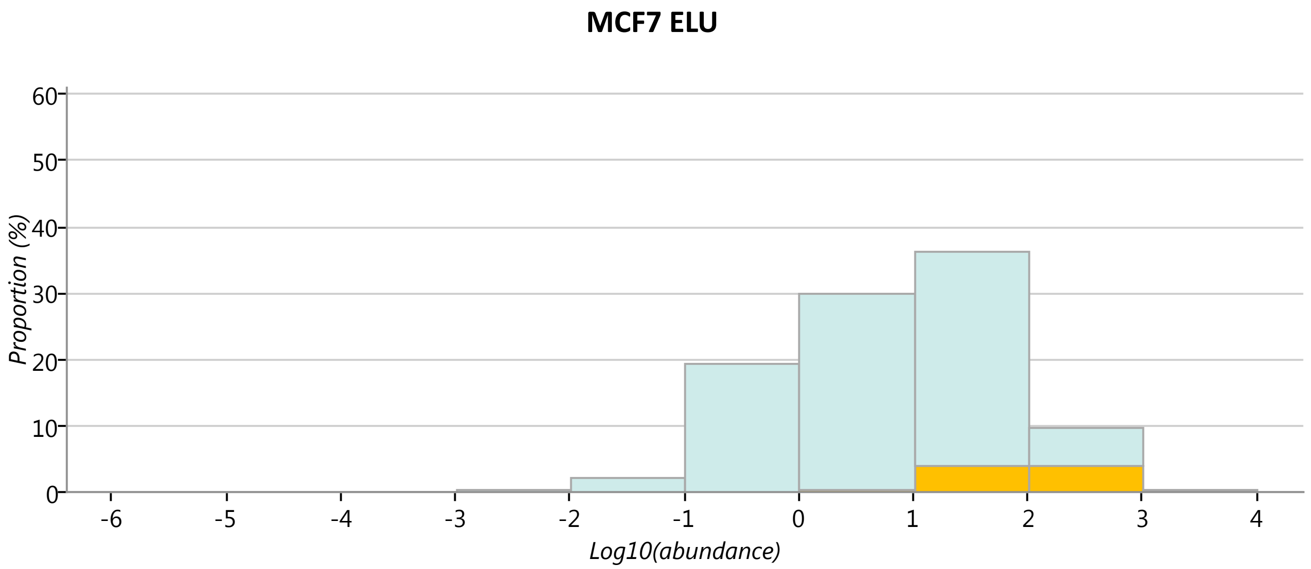

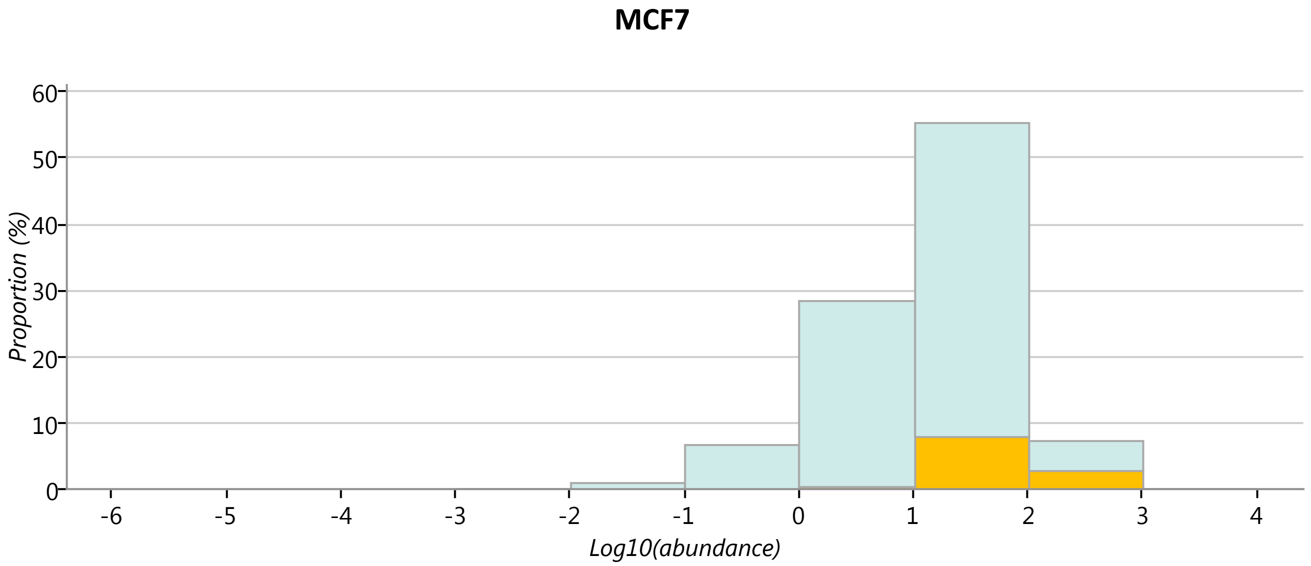

Not identified Identified

Mass accuracy

The chart below shows all identified peptide ions in your experiment, plotting peptide ion m/z against the ppm error in its highest scoring identification. Blue points indicate peptide ions which have a mass error within 2 standard deviations of the mean, yellow points indicate peptide ions with a mass error more than 2 standard deviations from the mean.

Potential problems:

- A dependence of ppm mass error on m/z may indicate poor calibration.
- If the standard deviation of the data is large in ppm terms, this may indicate that your search parameters are too lenient.

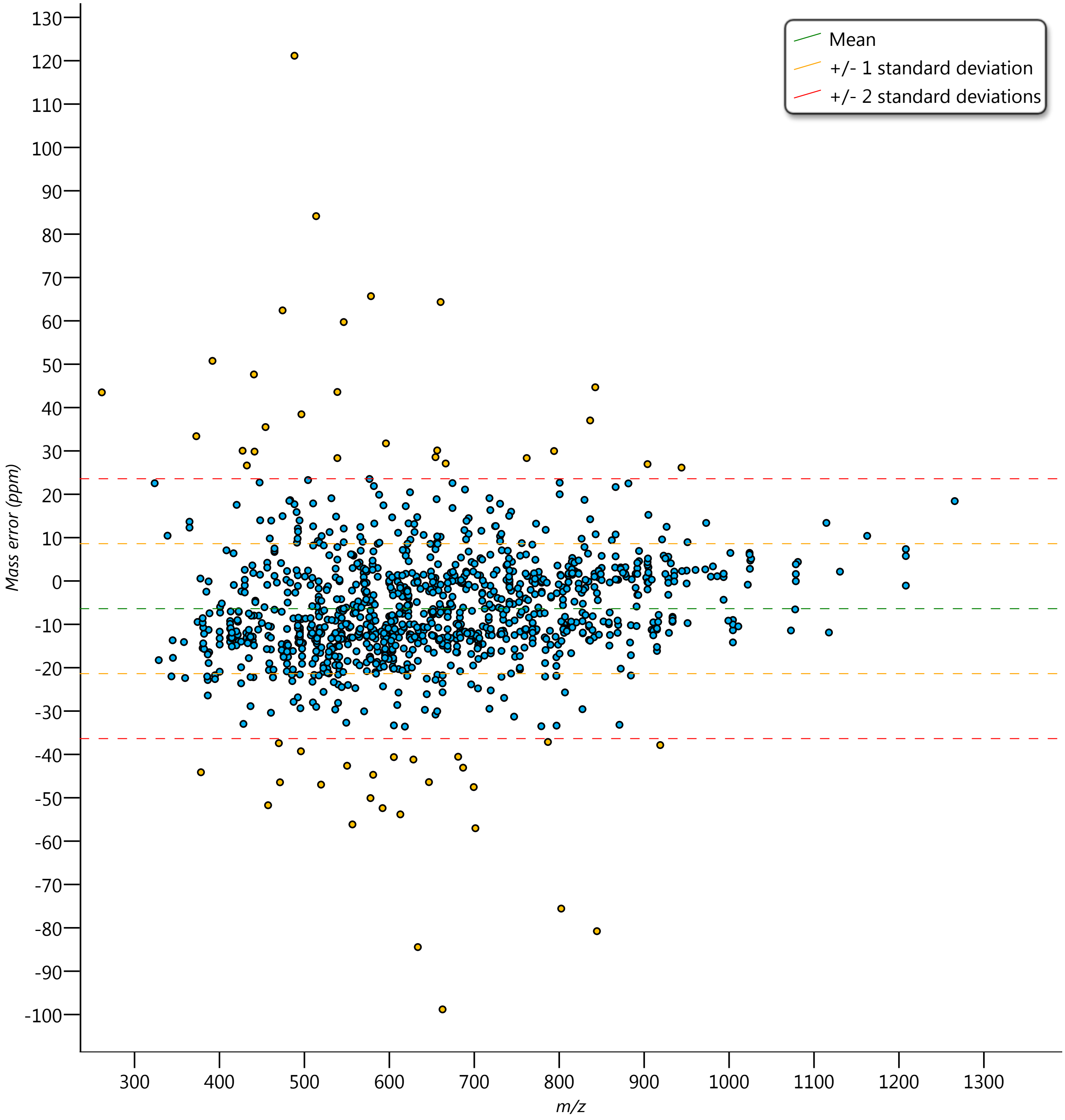

Scan rates

The charts below show the MS1 scan rate over the duration of your runs, using the current experiment design. Each chart shows the scan rate variation within an experimental condition's runs. The named run is highlighted in black. The variability between runs is shown as a grey area. The y-axis shows scans per second in a 30-second window centred on each x-axis time point.

Potential problems:

- A highly varying scan rate means quantitation accuracy may be negatively affected.

HCC1954 ELU **Run:** 20140724_DAP_HCC1954_ELUIDO_2ug_inj5_STEP3

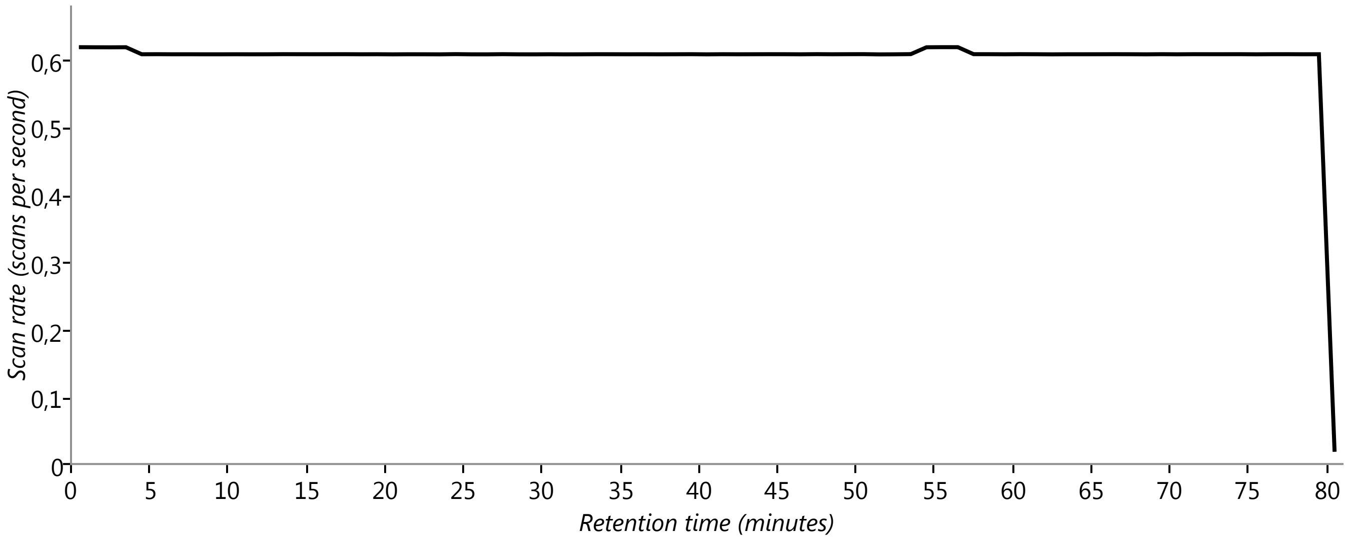

HCC1954 **Run:** 20140801_DAP_HCC1954_NAO_RETIDO_2ug_inj2_STEP3

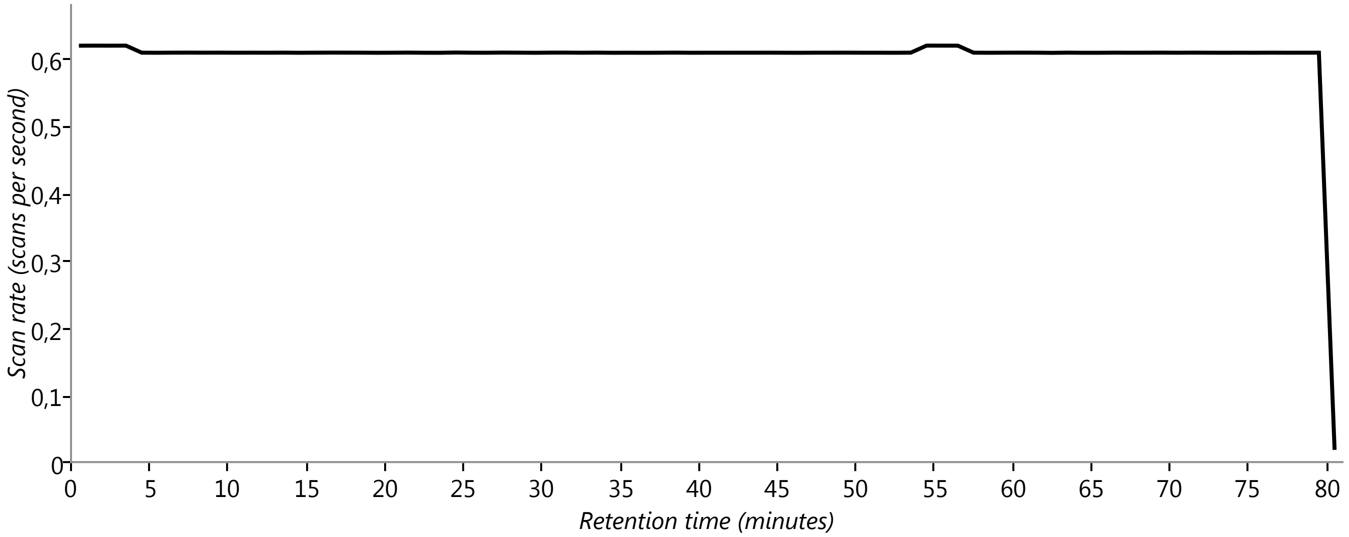

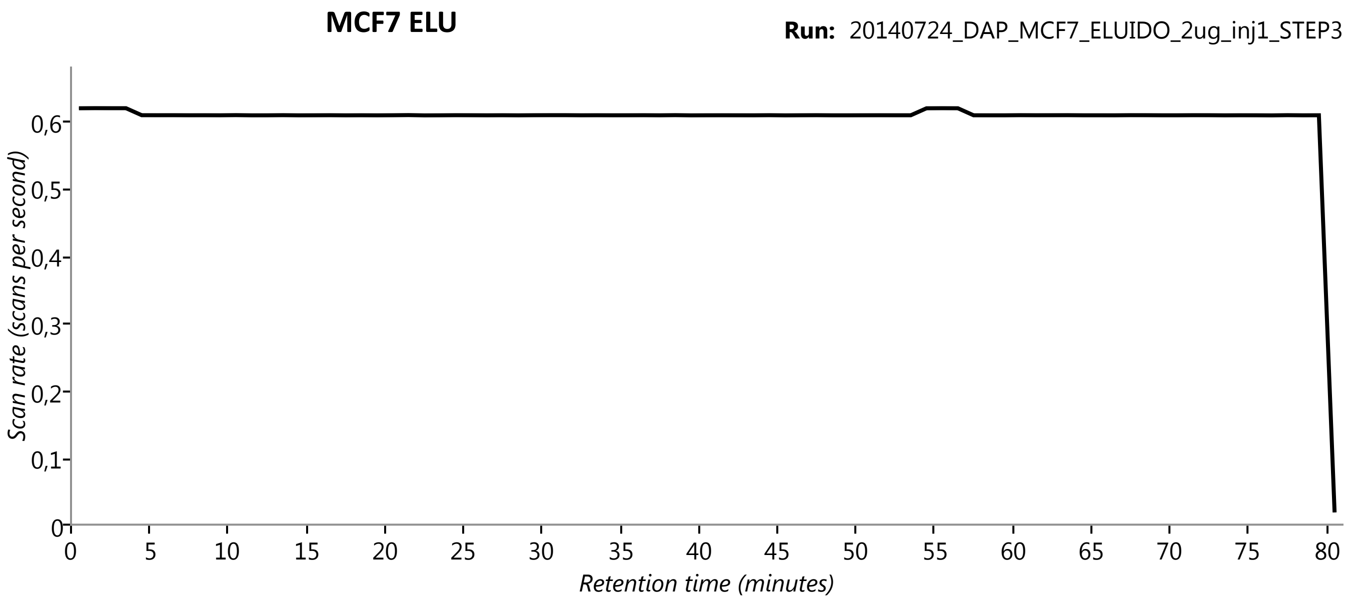

MCF7 ELU
Run: 20140724_DAP_MCF7_ELUIDO_2ug_inj1_STEP3
Scan rate (scans per second)
0,6
0,5
0,4
0,3
0,2
0,1
0
0 5 10 15 20 25 30 35 40 45 50 55 60 65 70 75 80
Retention time (minutes)

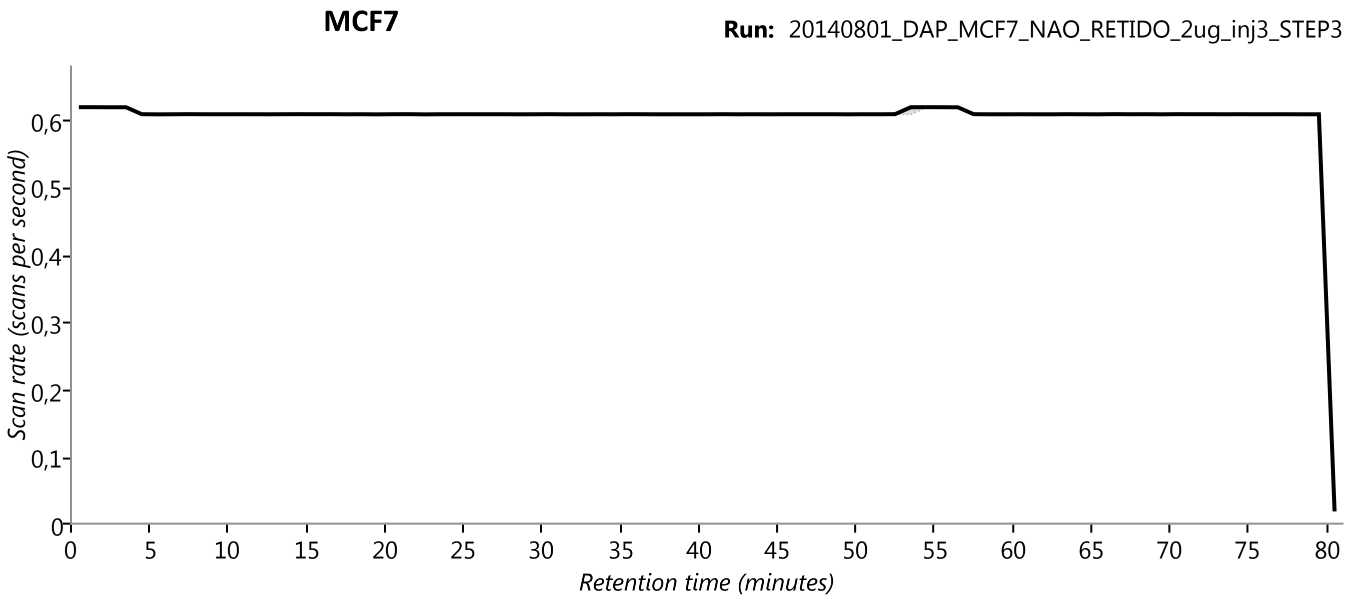

MCF7
Run: 20140801_DAP_MCF7_NAO_RETIDO_2ug_inj3_STEP3
Scan rate (scans per second)
0,6
0,5
0,4
0,3
0,2
0,1
0
0 5 10 15 20 25 30 35 40 45 50 55 60 65 70 75 80
Retention time (minutes)

Proteins

The chart below shows the number of proteins identified in each of the runs within the selected experiment design. Each run is coloured according to its experimental condition.

Potential problems:

- Any exceptionally high or low values may indicate problems with sample preparation, chromatography, or instrument settings.

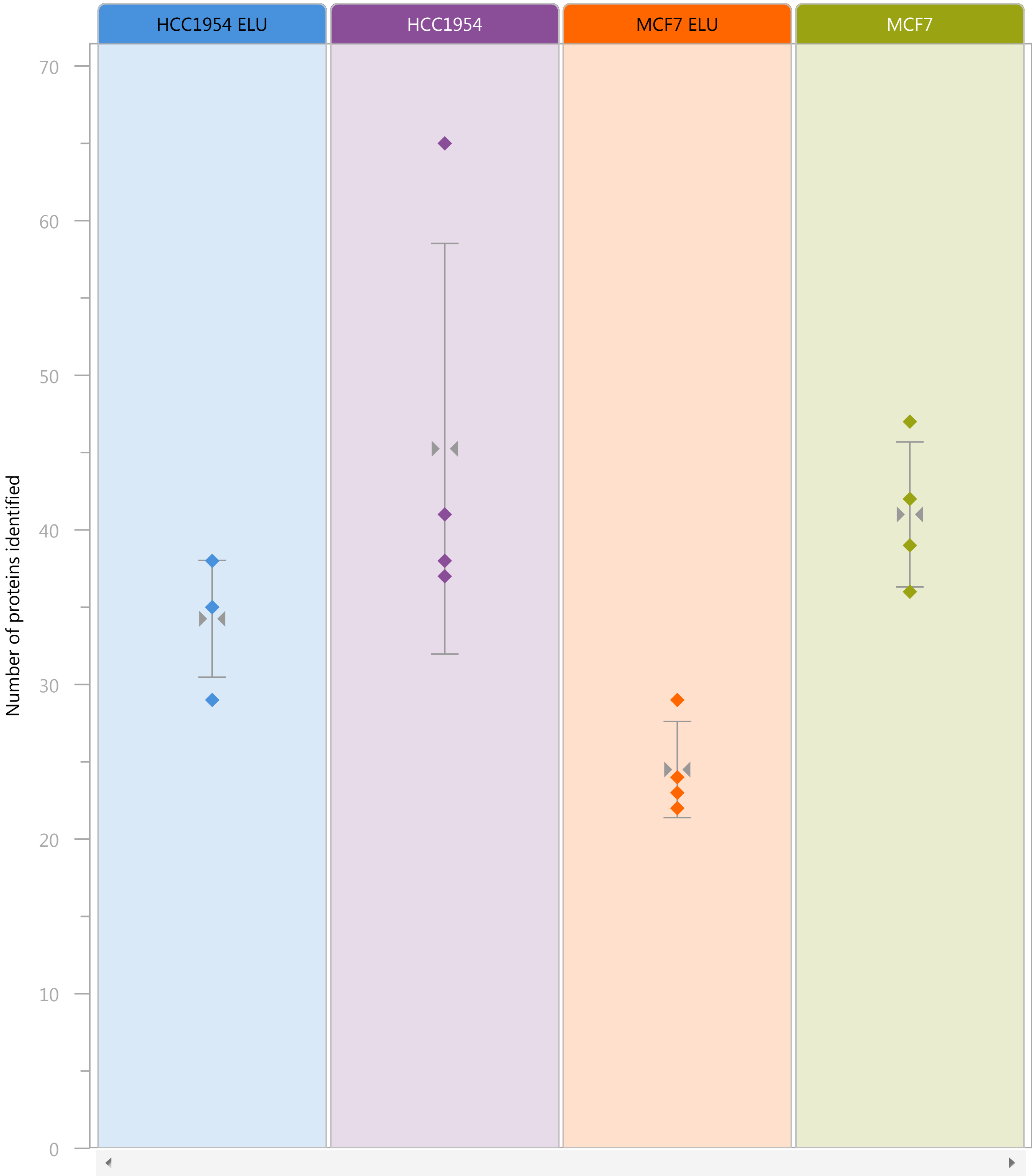

Peptides

The chart below shows the number of peptides identified in each of the experiment's runs. Each run is coloured according to its experimental condition.

Potential problems:

- Any exceptionally high or low values may indicate problems with sample preparation, chromatography, or instrument settings.

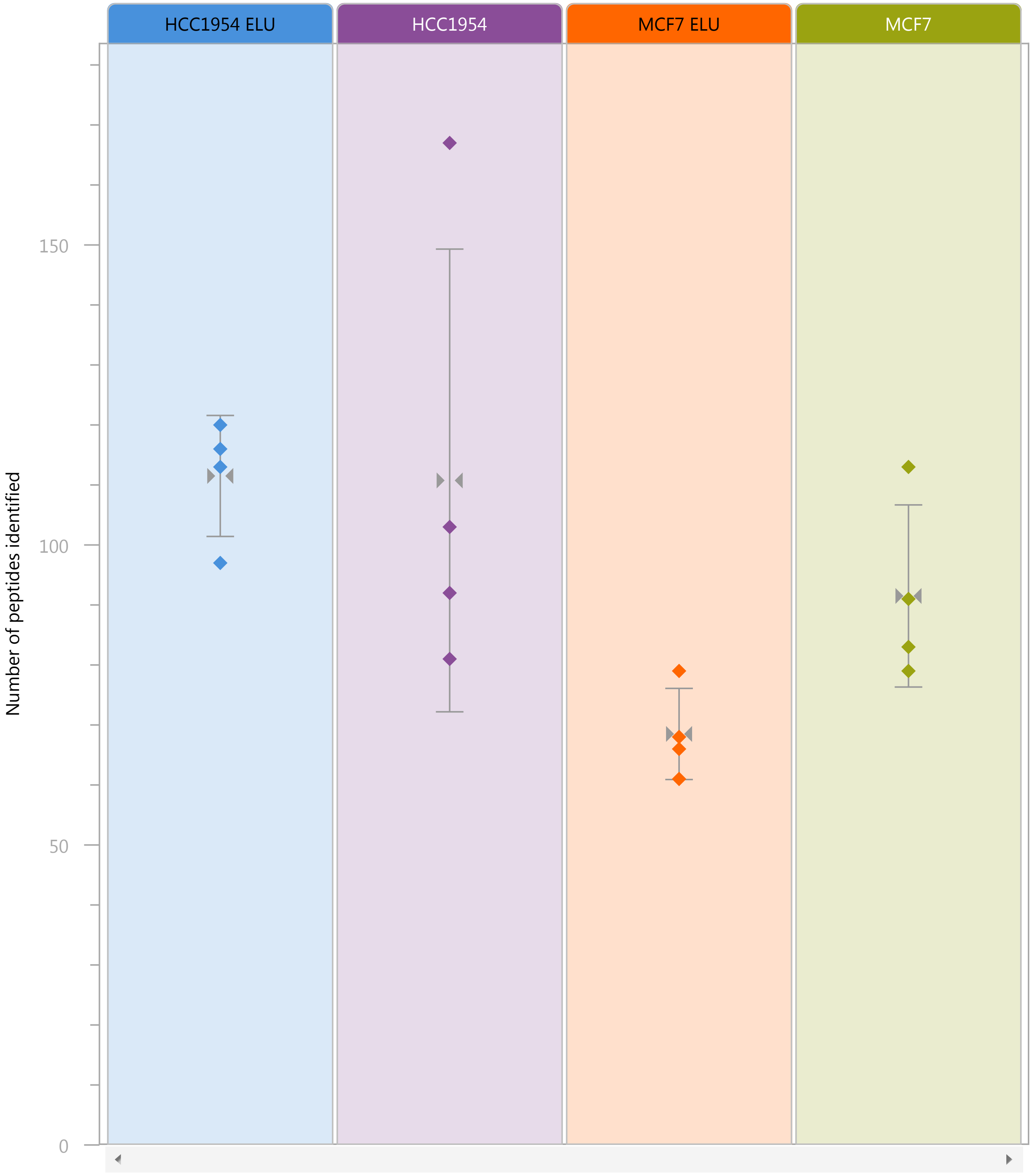

Peptides per protein (by condition)

The chart below shows the average number of peptides per protein in each run within the selected experiment design. For a given run, the value shown is calculated using only identifications from that run.

Potential problems:

- Any exceptionally high or low values may indicate problems with sample preparation or peptide search settings.

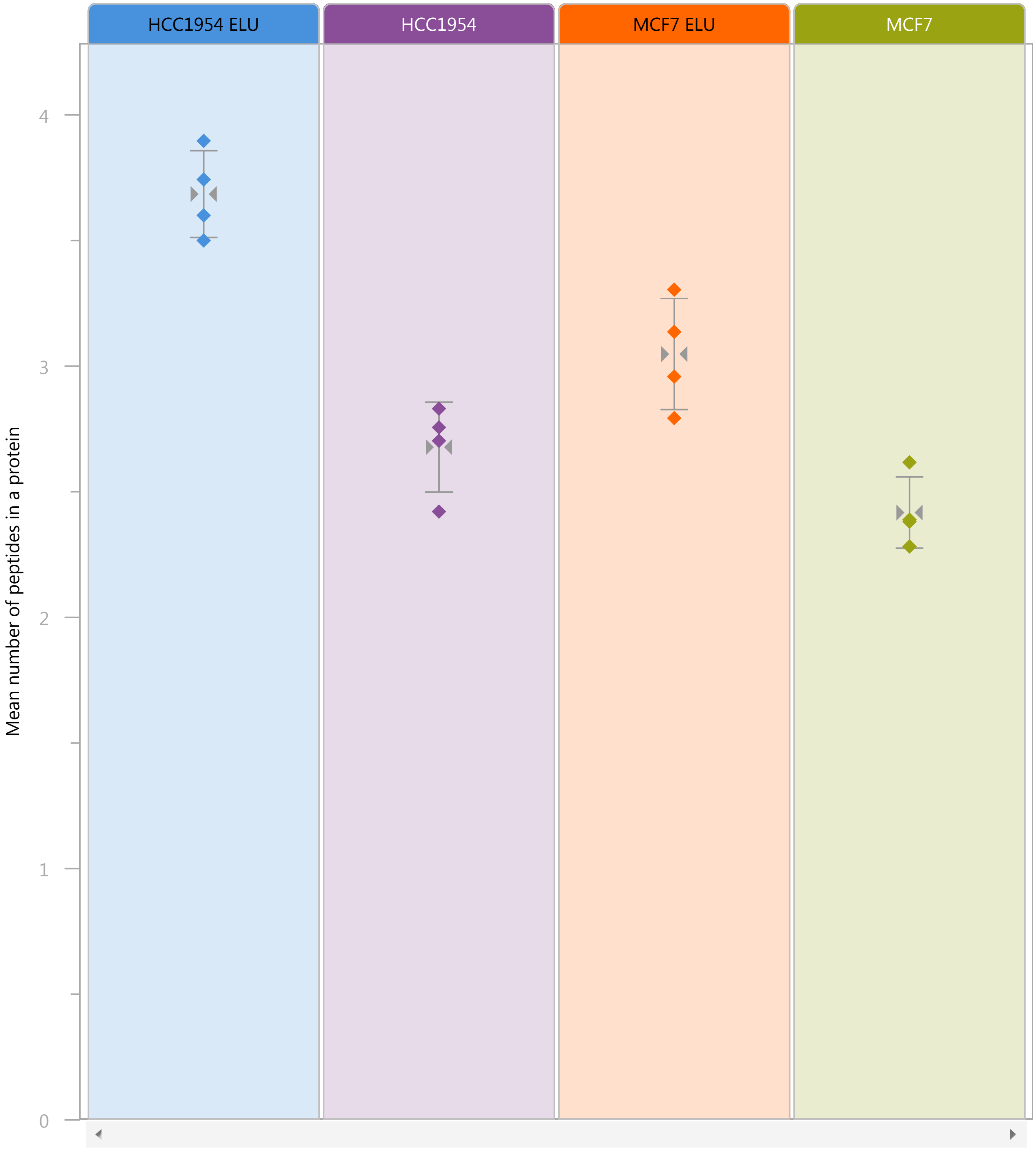

Percentage of peptide ions identified

The chart below shows the number of peptide ions identified in each of the runs within the selected experiment design, expressed as a percentage of the total number of peptide ions. Each run is coloured according to its experimental condition.

Potential problems:

- Any exceptionally high or low values may indicate problems with sample preparation, chromatography, or instrument settings.
- Very low values across all runs may indicate a problem with the database that was searched for identifications, or with other search parameters.

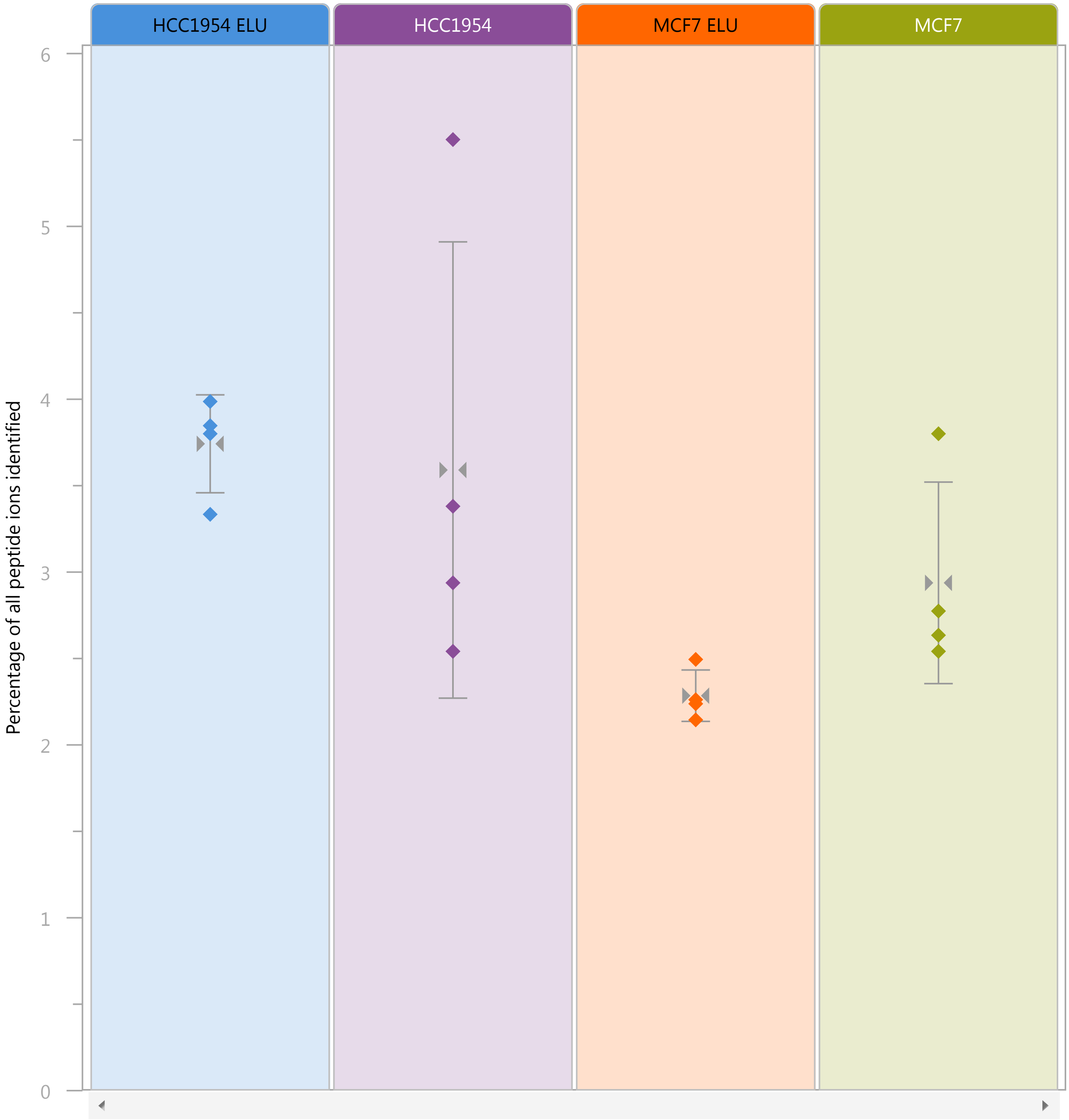

Protein overlap

The Venn diagram shows the number of proteins identified uniquely in the selected conditions and the number that are shared between conditions. Use the list on the right to select two or three conditions to explore.

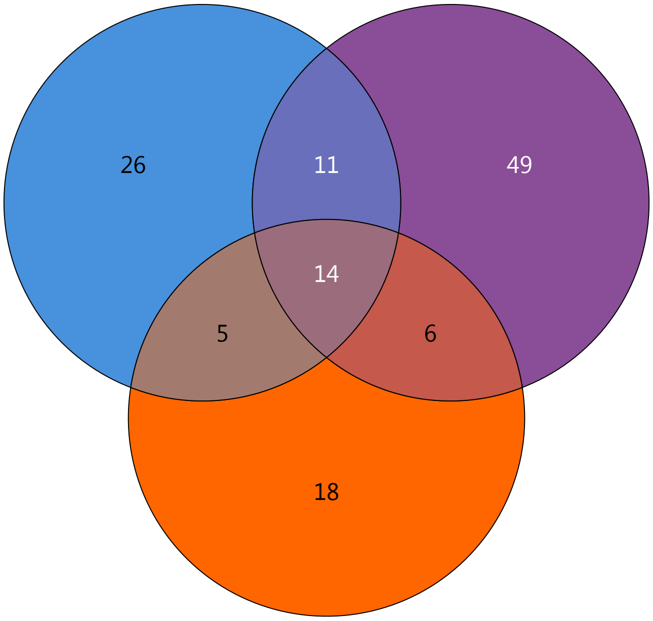

Select experimental conditions

- HCC1954 ELU
- HCC1954
- MCF7 ELU

HCC1954 x MCF7 STEP4

QC Metrics reported by Progenesis QI for proteomics v2.0.5556.29015
Date: 10-Jan-2020 at 09:27
Experiment design: HCC1954 x MCF7

This QC report contains **all metrics** for the experiment named above.

Reported metrics

0 of the 19 metrics reported have been manually flagged for attention.

- Identifications overview
- LC peak width
- Peptide ion dynamic range
- Precursor m/z
- Precursor retention time
- Mass errors
- Precursor charges
- Missed cleavages (assuming trypsin)
- Peptides per protein (overview)
- Modifications
- Missed cleavages
- Abundance dynamic range
- Mass accuracy
- Scan rates
- Proteins
- Peptides
- Peptides per protein (by condition)
- Percentage of peptide ions identified
- Protein overlap

Identifications overview

The chart below provides the number of identifications per run by category. Data are plotted for all runs in the current experiment design. The box represents the interquartile range and the whiskers the maximum and minimum.

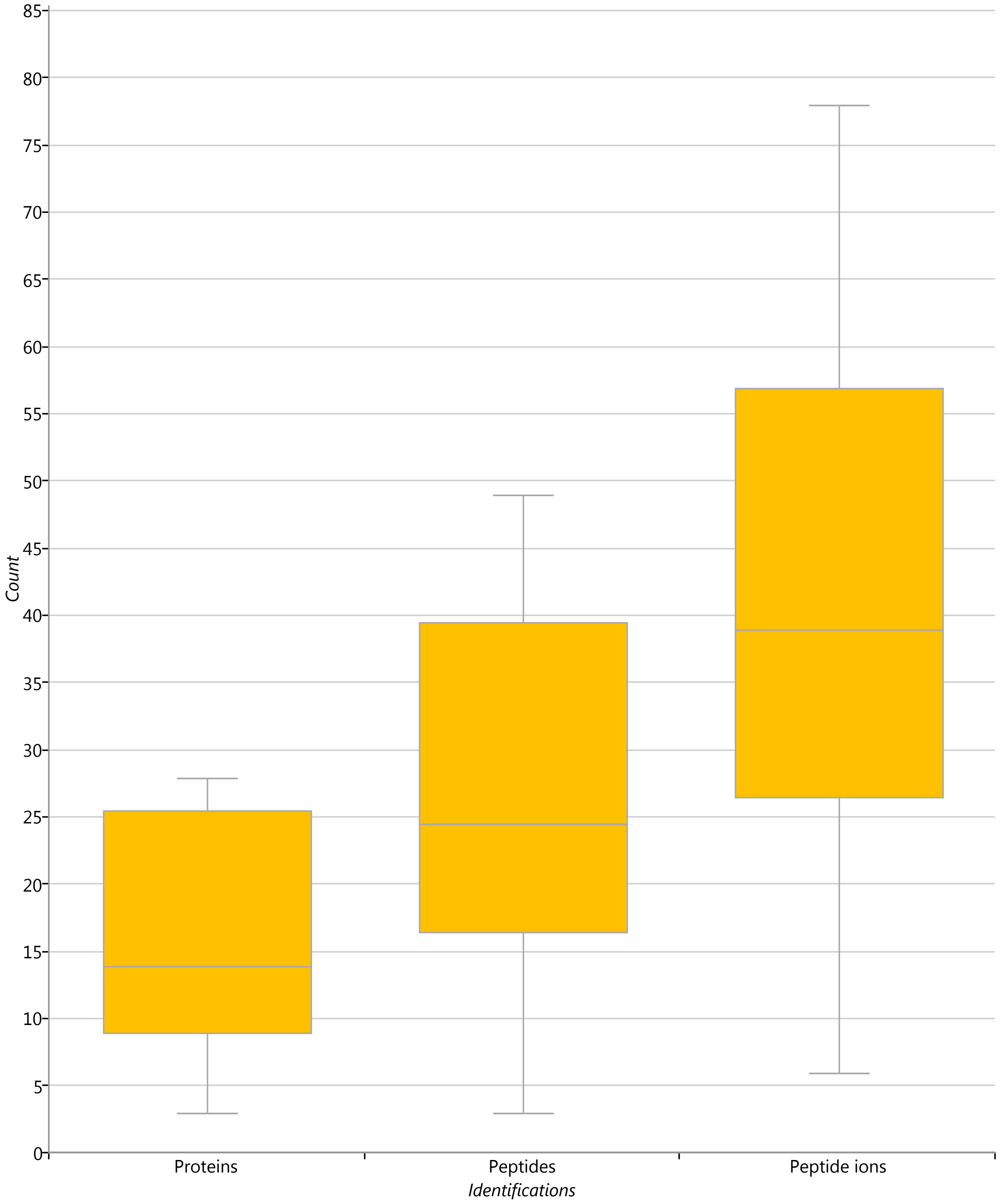

LC peak width

This chart shows the proportional distribution of peak widths in retention time, for all the detected peptide ions in the experiment. Widths are binned into categories for simple comparison. Identified peptide ions are shown in yellow.

Potential problems:

- A peak width that is different from that expected for your setup may indicate a problem with the chromatographic separation.

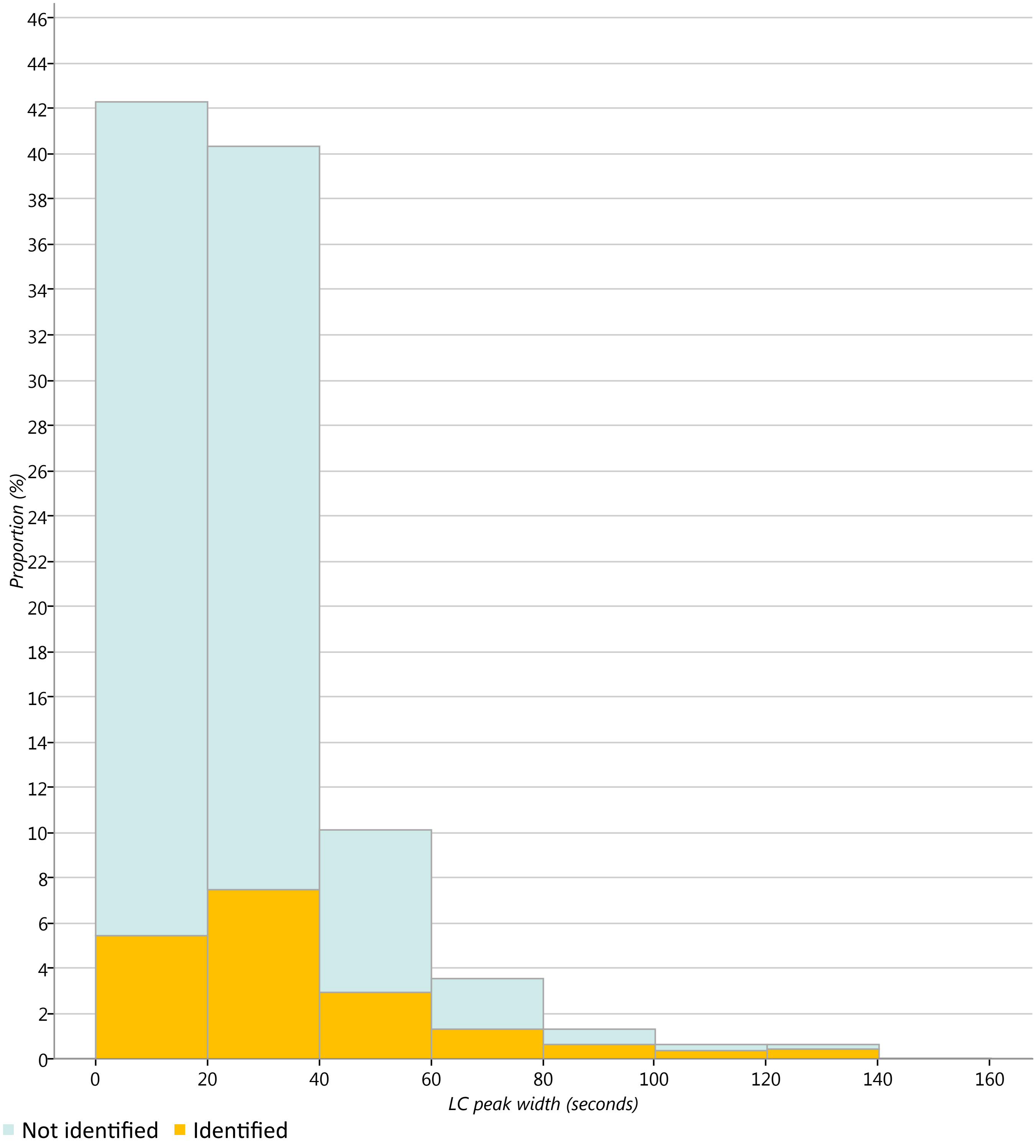

Peptide ion dynamic range

This chart shows log abundance for peptide ions, calculated using the current experiment design. Each peptide ion is assigned its abundance based on the highest mean value for any condition's runs. Data are binned and shown as a proportion. Identified peptide ions are shown in yellow.

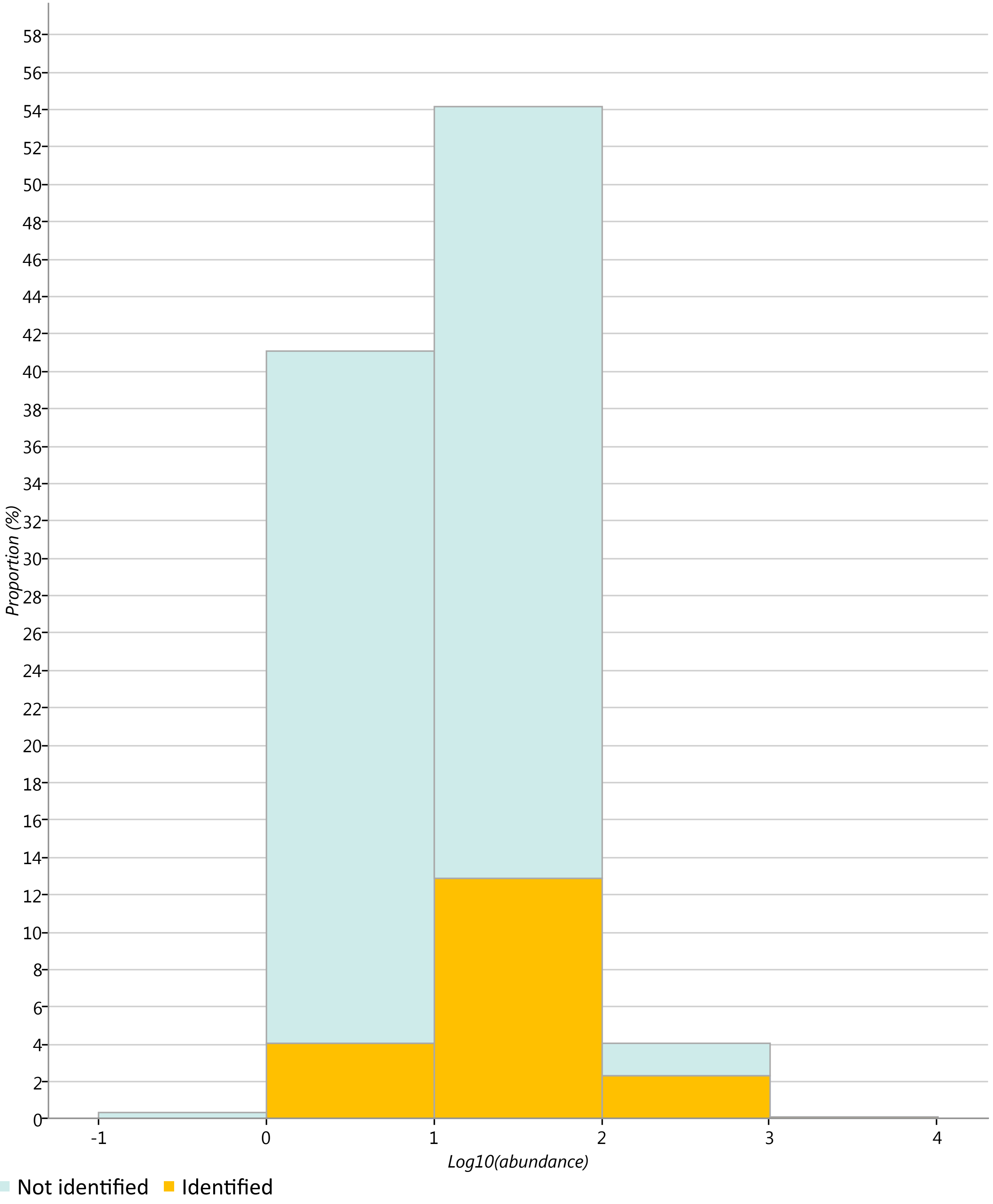

Precursor m/z

This chart shows the distribution of m/z values for all peptide ions in the experiment. Identified peptide ions are shown in yellow.

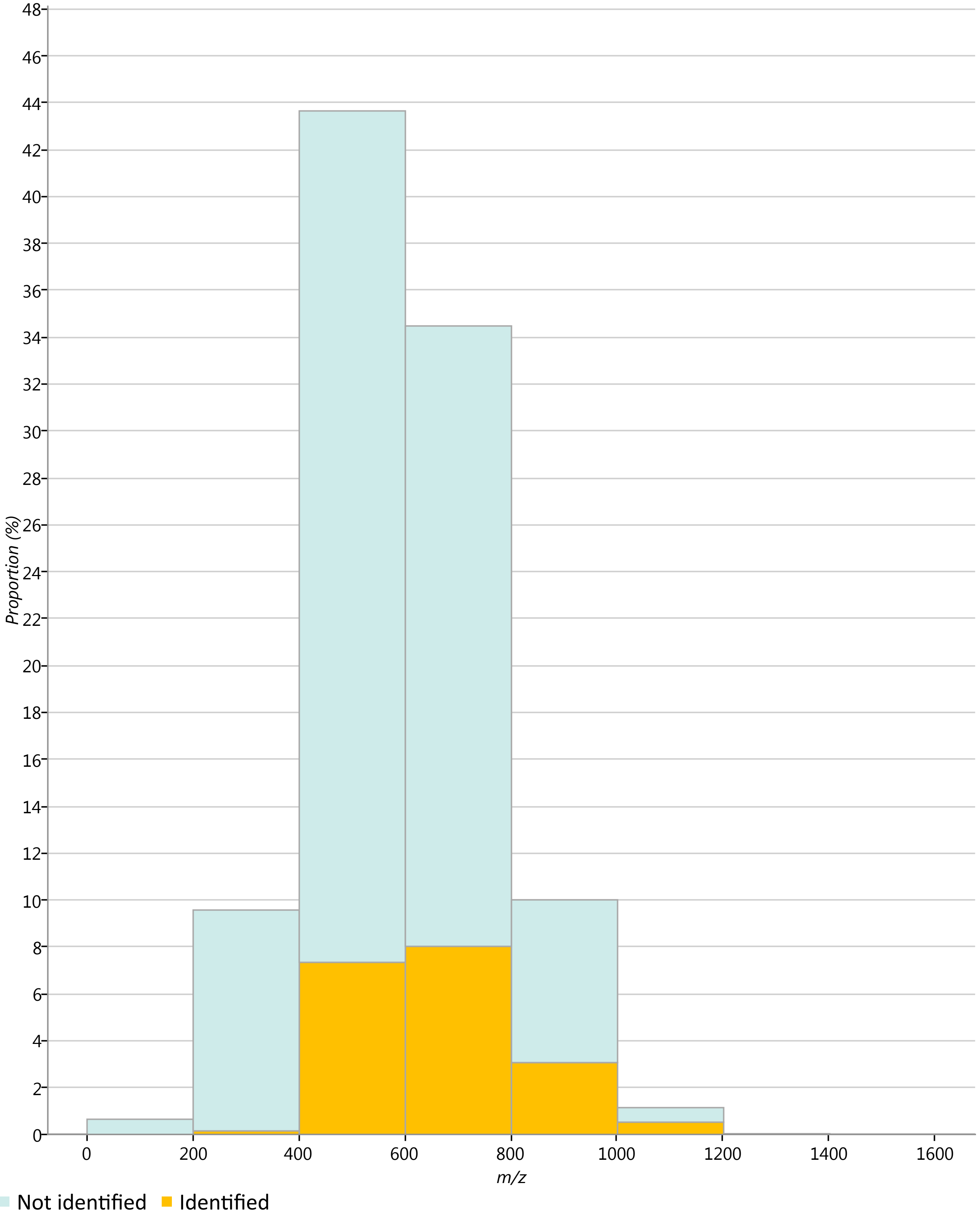

Precursor retention time

This chart shows the proportion of precursor peptide ions eluting into each quartile of the LC run. Identified peptide ions are shown in yellow.

Potential problems:

- A majority of peptide ions eluting at the start or end of the run may indicate problems with the chromatographic separation or that the setup is not ideal for a successful separation.

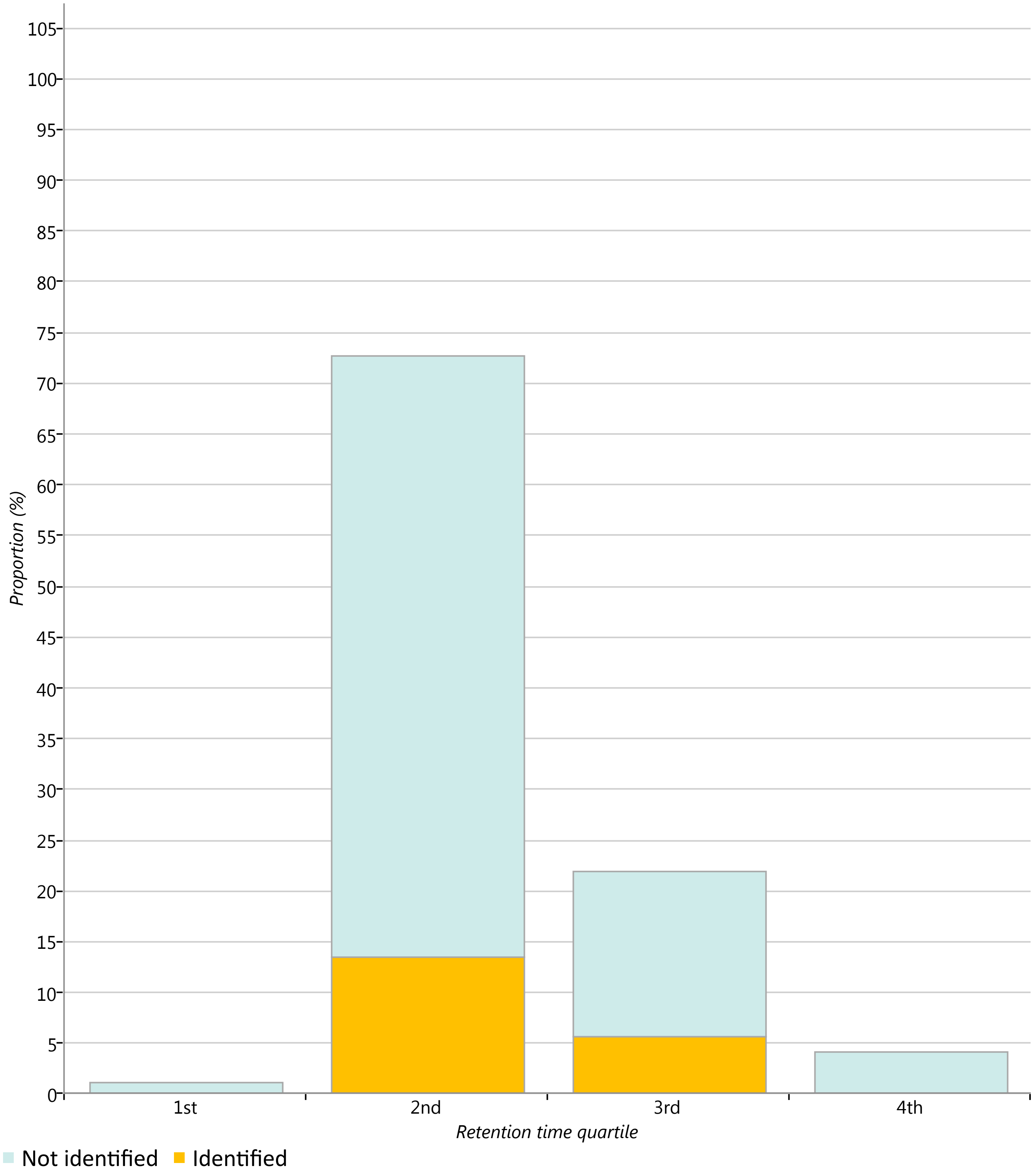

Mass errors

This chart shows the mass error calculated for identified peptide ions. The proportion of peptide ions with a given binned error value is plotted. For a more detailed plot, see the *Mass accuracy* metric.

Potential problems:

- A larger than expected spread could indicate that search parameters are too lenient.
- A bias of the peak from zero ppm could indicate instrument bias.

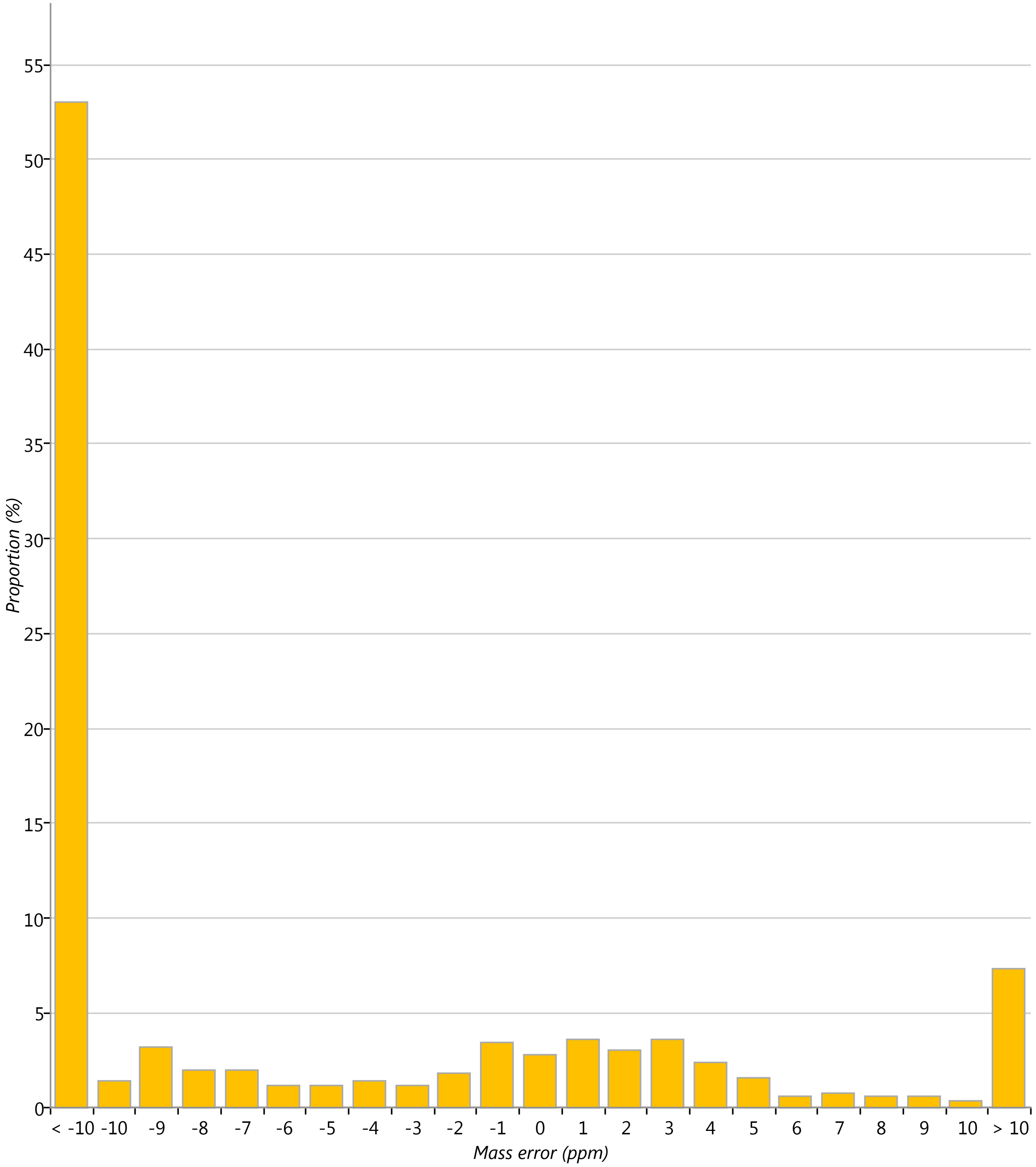

Precursor charges

This chart shows the percentage of peptide ions with various charge states.

Potential problems:

- A skew towards low charge peptide ions may indicate poor ionisation, suggesting that you may need to raise the collision voltage.
- A skew towards high charge peptide ions may indicate excessive ionisation, suggesting that you may need to lower the collision voltage.

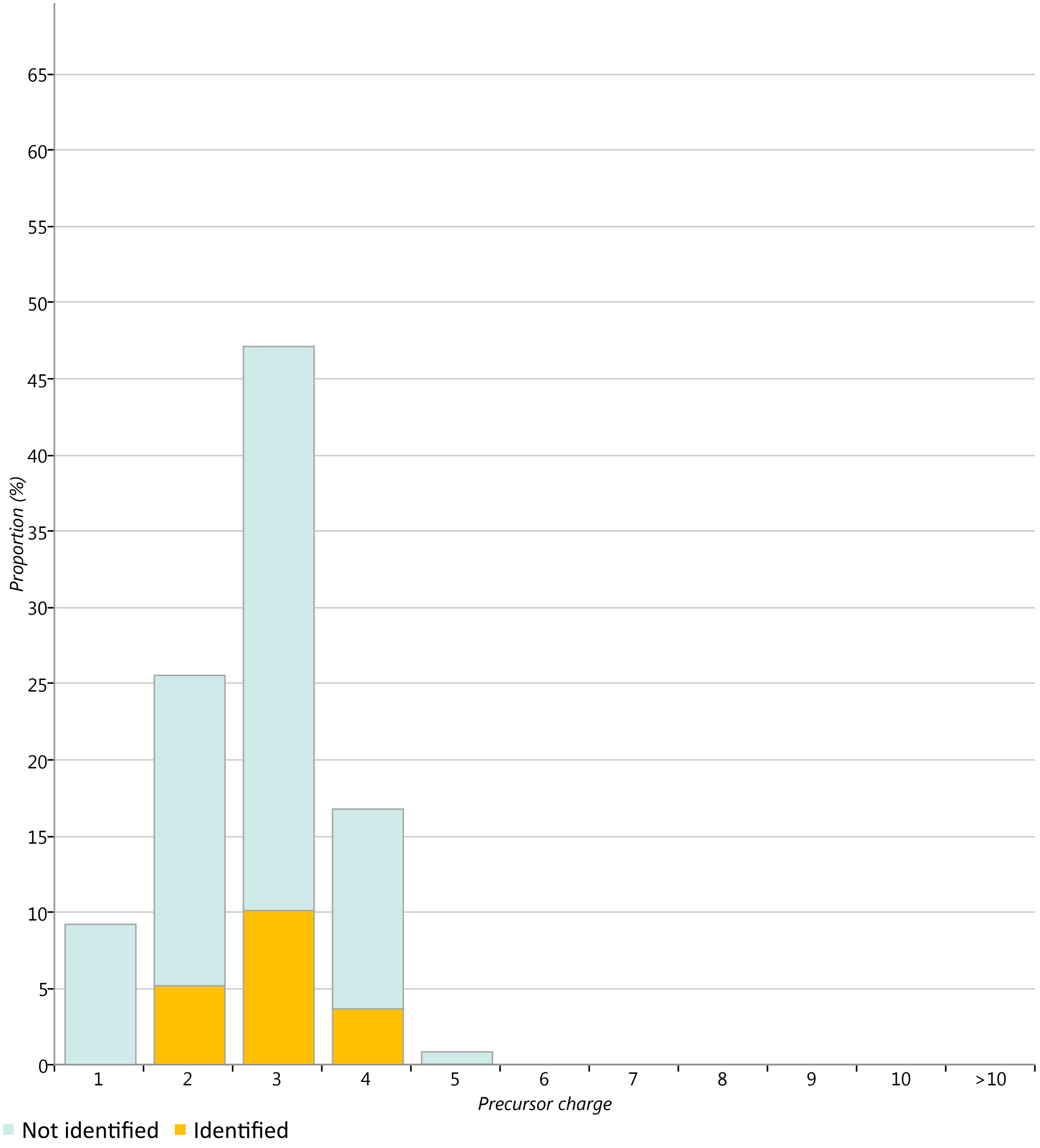

Missed cleavages (assuming trypsin)

This chart shows the distribution of missed cleavages per identified peptide ion across the whole experiment, assuming the proteins were treated with trypsin and using each peptide ion's highest scoring identification. For an alternative visualisation showing the data subdivided by experimental condition, see the *Missed cleavages* metric.

Potential problems:

- A high proportion of missed cleavages may indicate problems in trypsinization.
- An absence of missed cleavages may indicate that the database search parameters were too stringent.

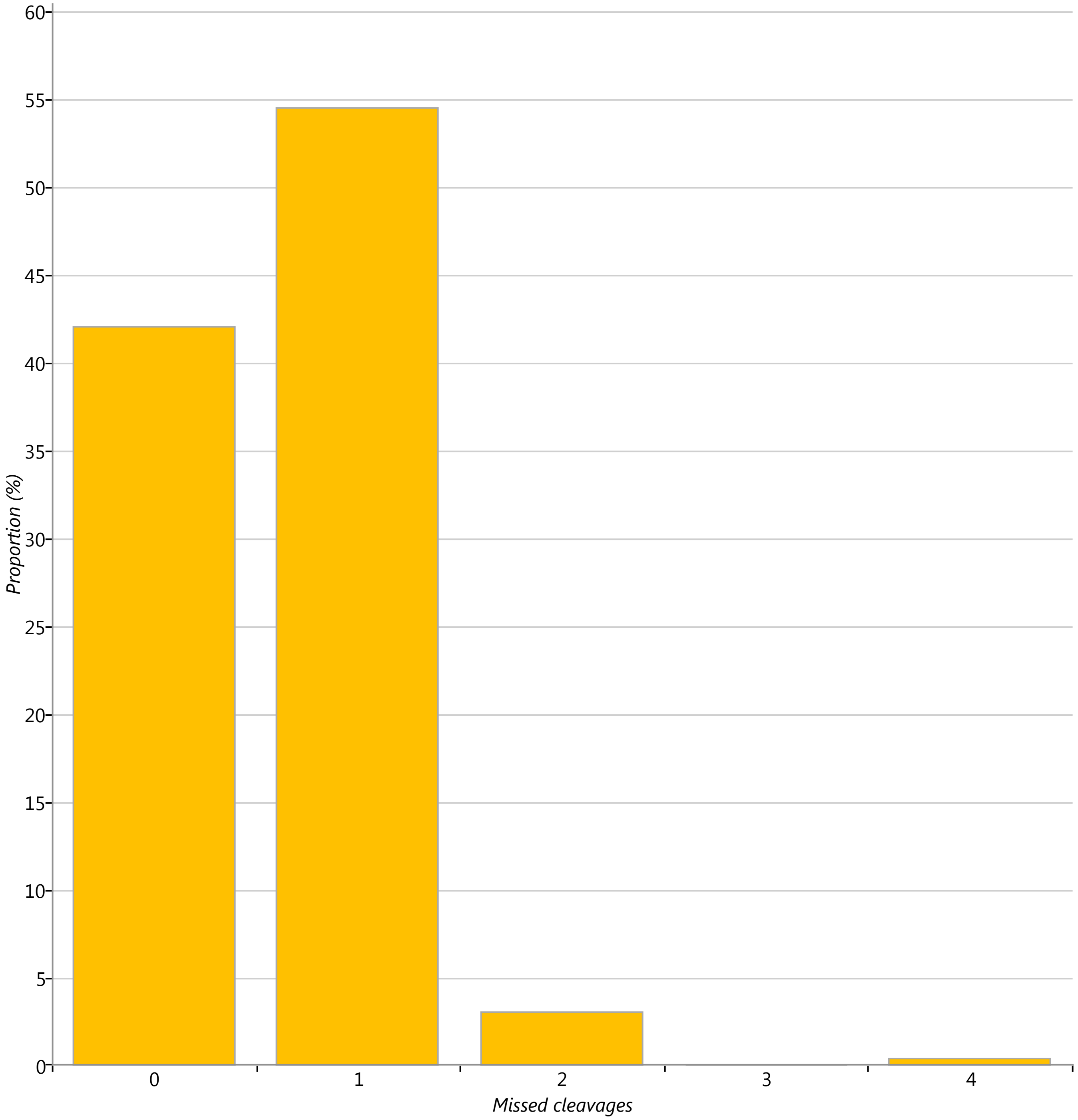

Peptides per protein (overview)

This chart shows the proportional distribution of the number of peptides identified per protein, combined across all runs in the experiment. For an alternative visualisation dividing these data by experimental condition, see the *Peptides per protein (by condition)* metric.

Potential problems:

- An unexpected bias to high or low numbers of peptides may indicate problems with sample preparation or peptide search settings.

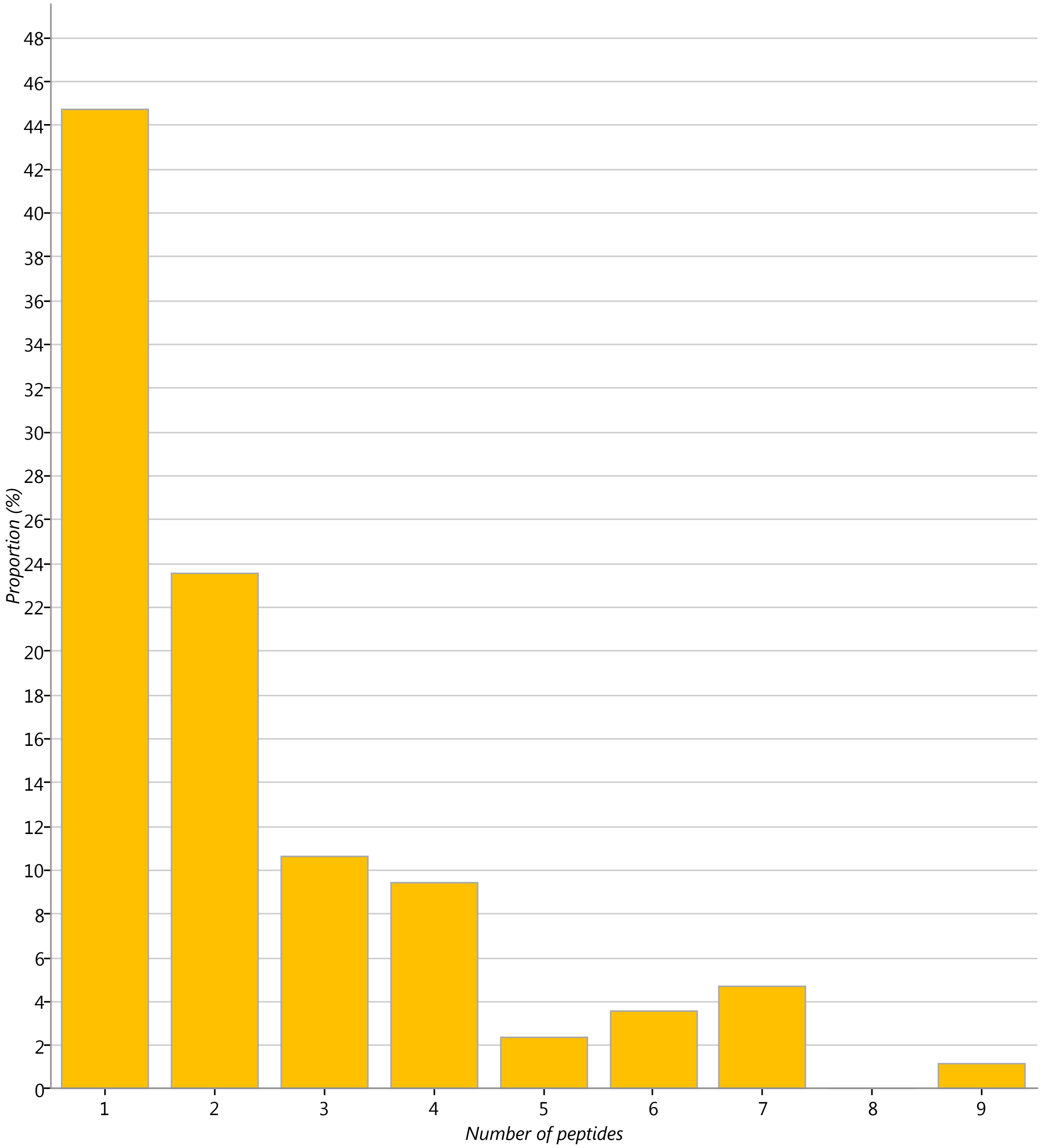

Modifications

The chart below shows the mean number of modifications per peptide ion for runs in the selected experiment design, based on each peptide ion's highest scoring identification. Each run is coloured according to its experimental condition.

Potential problems:

- Unexpectedly low numbers of modifications may indicate that one or more modifications were mistakenly not selected when searching for identifications.
- Variable numbers of modifications across the runs, especially within a given condition, may indicate problems with sample preparation.

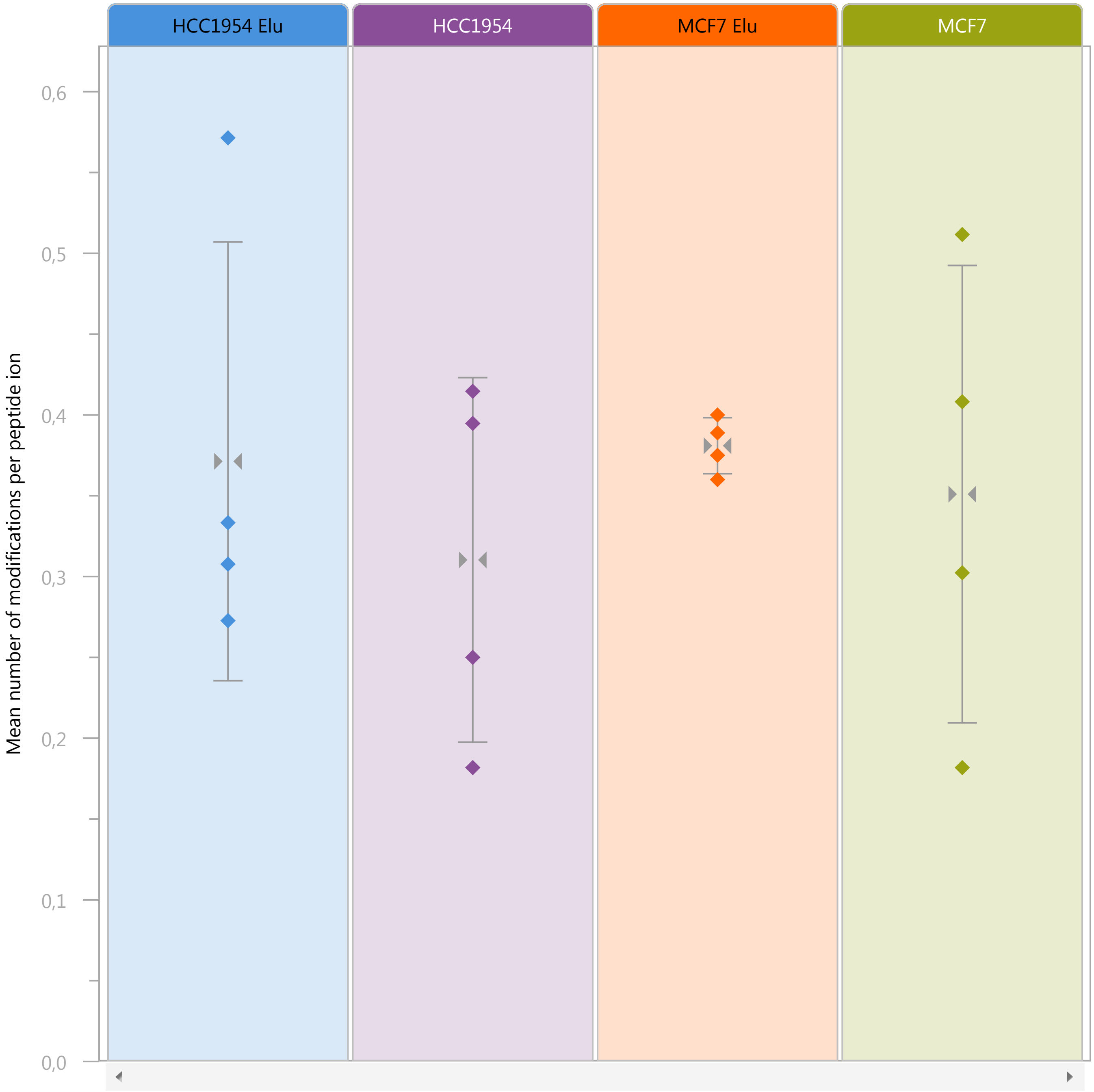

Included modifications Acetyl N-TERM Carbamidomethyl C Carbamyl N-TERM Deamidation N Dean

Missed cleavages

The chart below shows the mean number of missed cleavages per peptide ion for runs within the selected experiment design, based on each peptide ion's highest scoring identification. The chart assumes the use of trypsin for digestion. Each run is coloured according to its experimental condition.

Potential problems:

- A high number of missed cleavages may indicate problems in trypsinization.
- An absence of missed cleavages may indicate that database search parameters were too stringent.

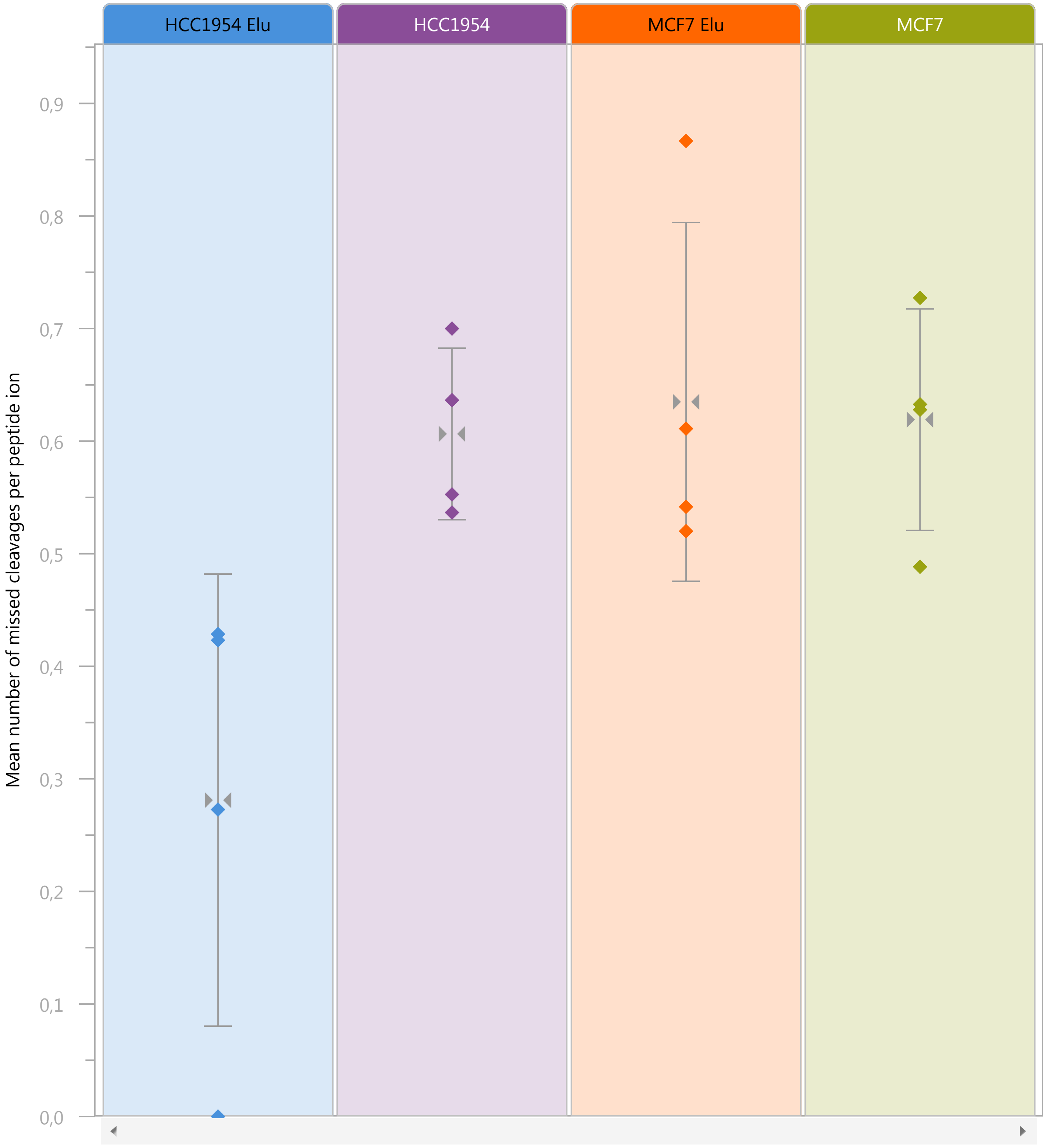

Abundance dynamic range

The charts below show abundance histograms for peptide ions, calculated using the current experiment design. Each chart shows the mean abundance for peptide ions within an experimental condition's runs. Data are binned by Log10 of their normalised abundance and shown as a proportion of the total. Identified peptide ions are shown in yellow.

Potential problems:

- A heavily right-skewed histogram of *identified* peptide ions indicates you may be missing identifications with low abundance.
- A heavily right skewed total (summed identified and unidentified) histogram may indicate saturation at high abundance, which will affect the accuracy of quantitation.

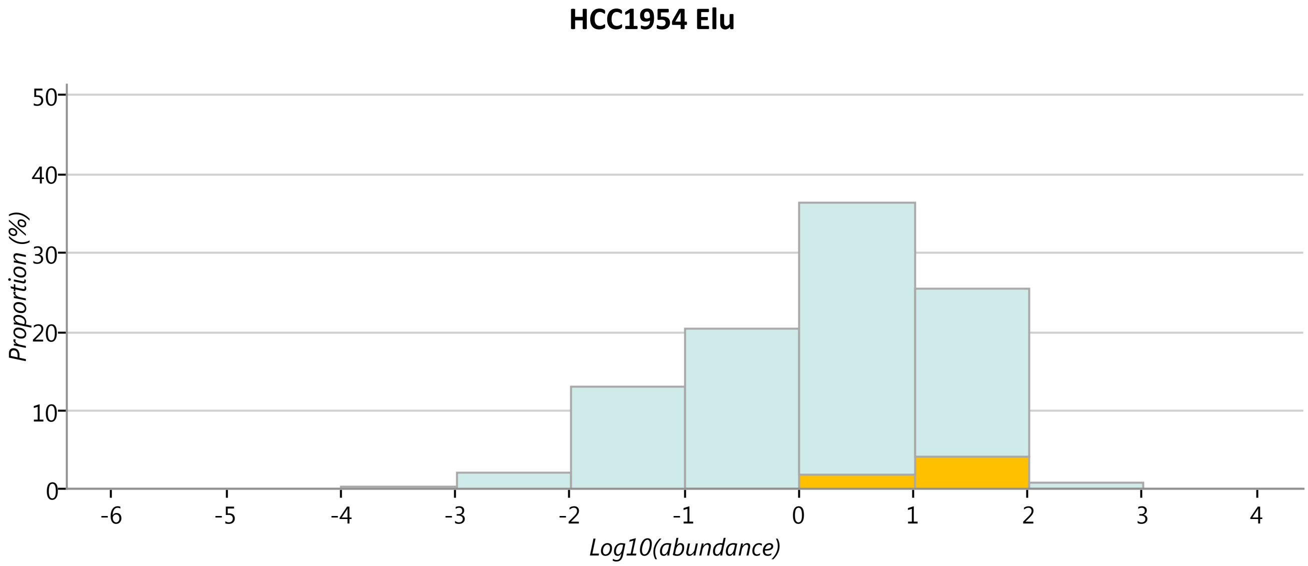

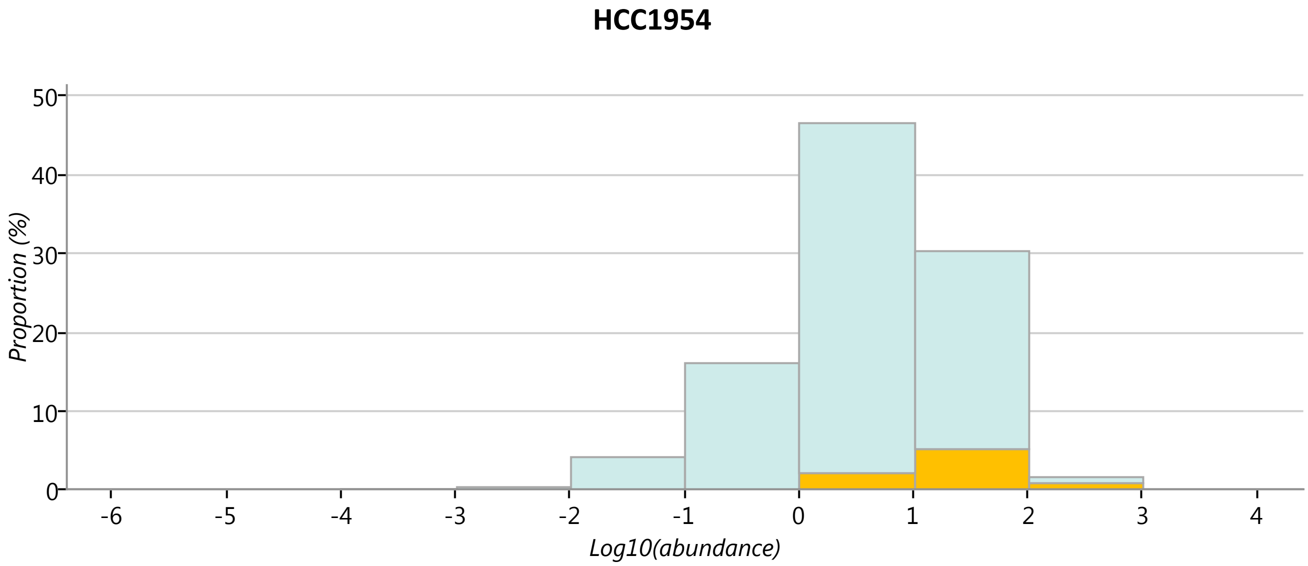

Not identified Identified

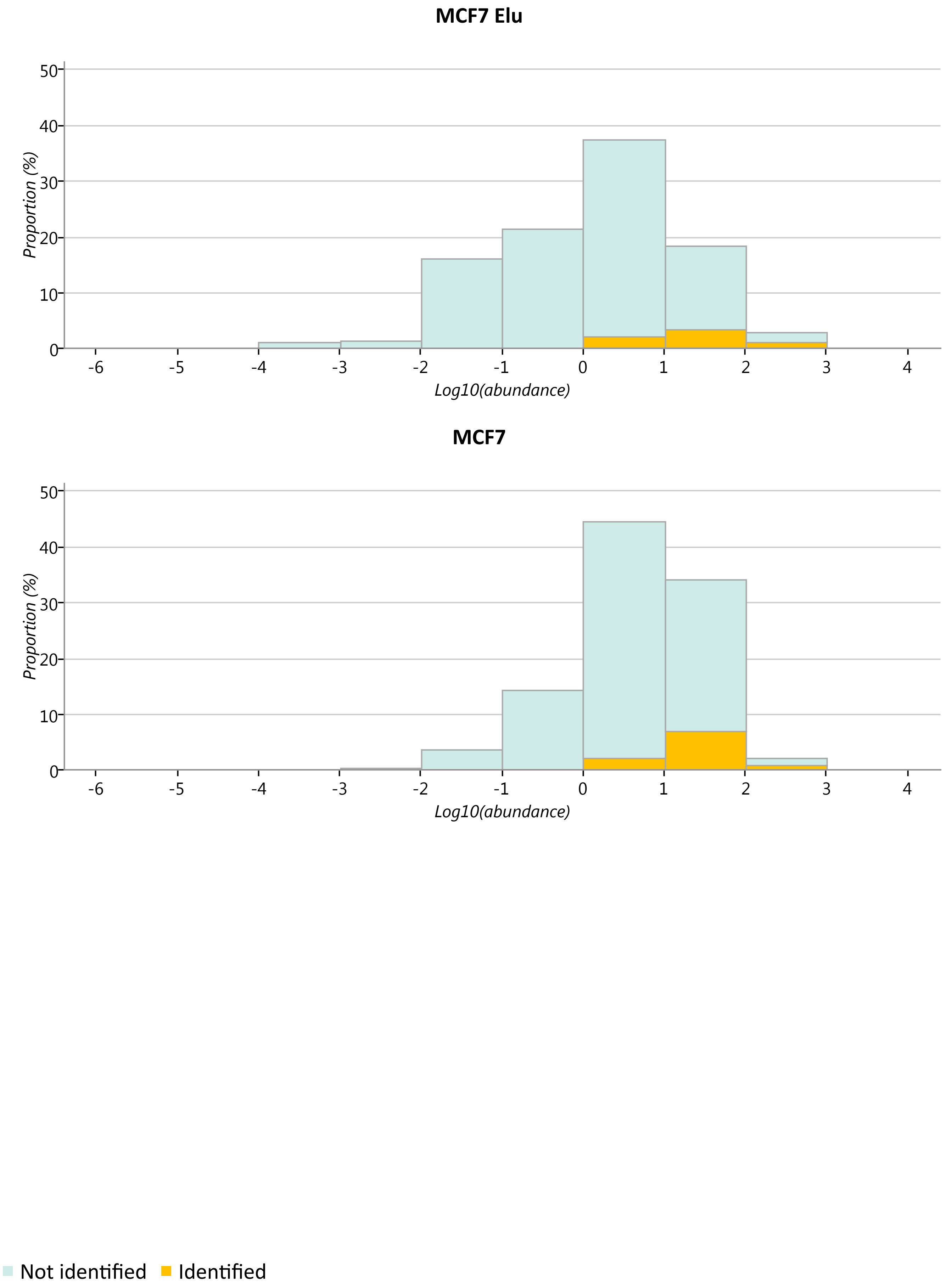
MCF7 Elu
Proportion (%)
50
40
30
20
10
0
-6
-5
-4
-3
-2
-1
0
1
2
3
4
Log10(abundance)
MCF7
Proportion (%)
50
40
30
20
10
0
-6
-5
-4
-3
-2
-1
0
1
2
3
4
Log10(abundance)
Not identified
Identified

Mass accuracy

The chart below shows all identified peptide ions in your experiment, plotting peptide ion m/z against the ppm error in its highest scoring identification. Blue points indicate peptide ions which have a mass error within 2 standard deviations of the mean, yellow points indicate peptide ions with a mass error more than 2 standard deviations from the mean.

Potential problems:

- A dependence of ppm mass error on m/z may indicate poor calibration.
- If the standard deviation of the data is large in ppm terms, this may indicate that your search parameters are too lenient.

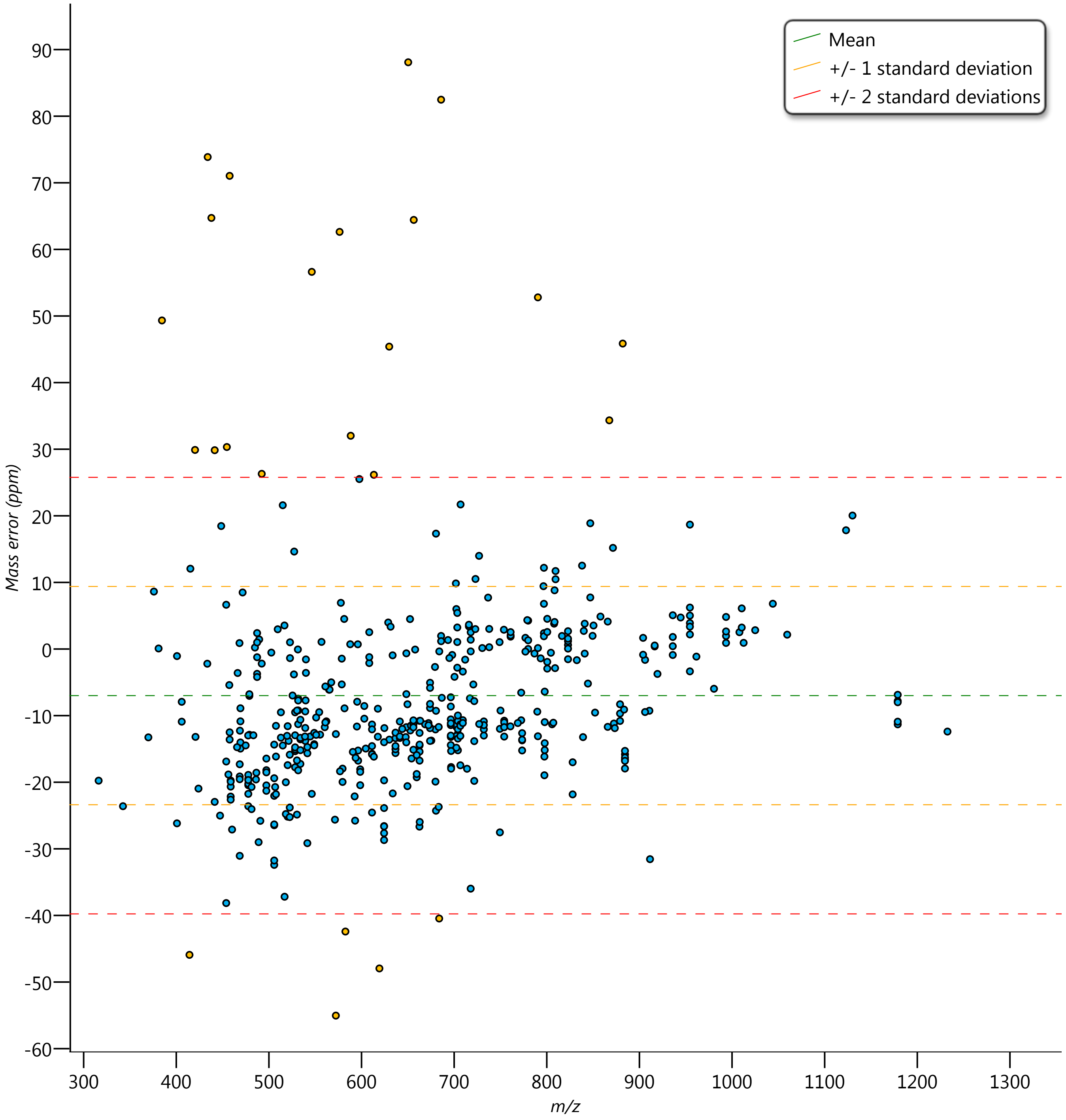

Scan rates

The charts below show the MS1 scan rate over the duration of your runs, using the current experiment design. Each chart shows the scan rate variation within an experimental condition's runs. The named run is highlighted in black. The variability between runs is shown as a grey area. The y-axis shows scans per second in a 30-second window centred on each x-axis time point.

Potential problems:

- A highly varying scan rate means quantitation accuracy may be negatively affected.

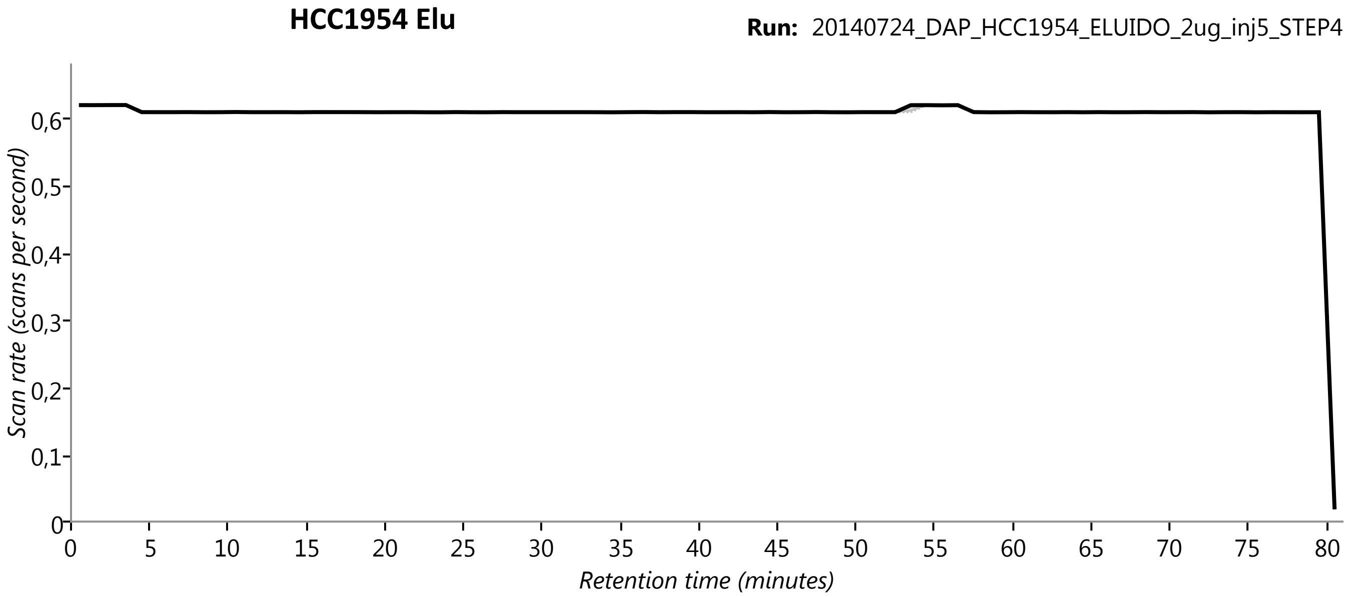

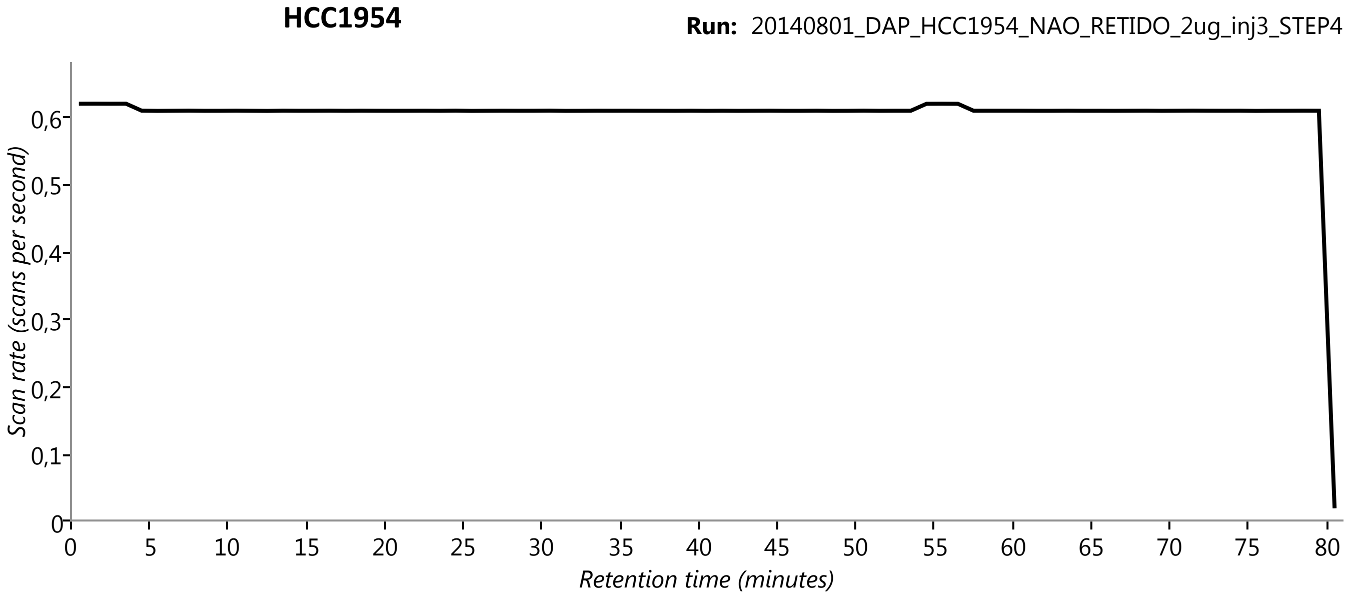

MCF7 Elu

Run: 20140724_DAP_MCF7_ELUIDO_2ug_inj4_STEP4

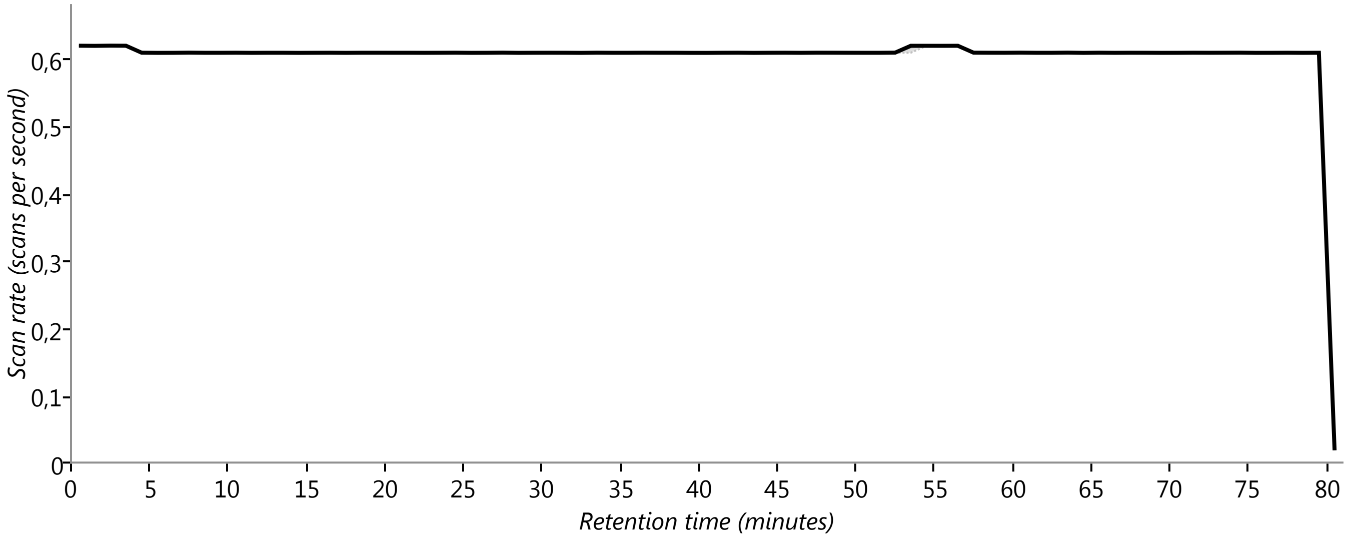

MCF7

Run: 20140801_DAP_MCF7_NAO_RETIDO_2ug_inj1_STEP4

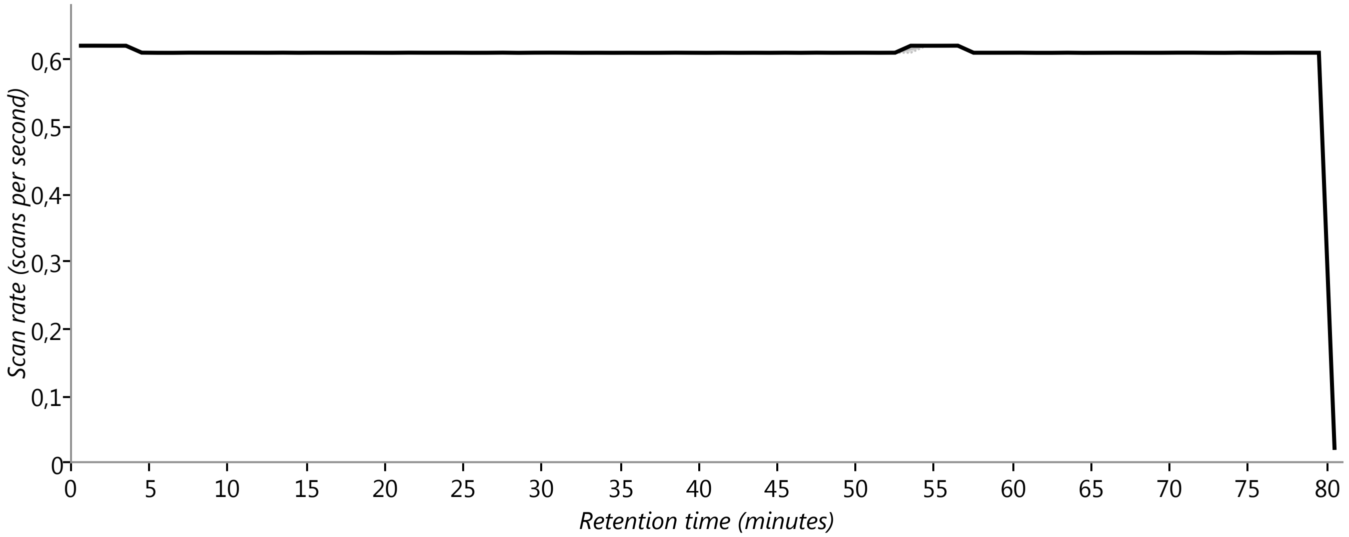

Proteins

The chart below shows the number of proteins identified in each of the runs within the selected experiment design. Each run is coloured according to its experimental condition.

Potential problems:

- Any exceptionally high or low values may indicate problems with sample preparation, chromatography, or instrument settings.

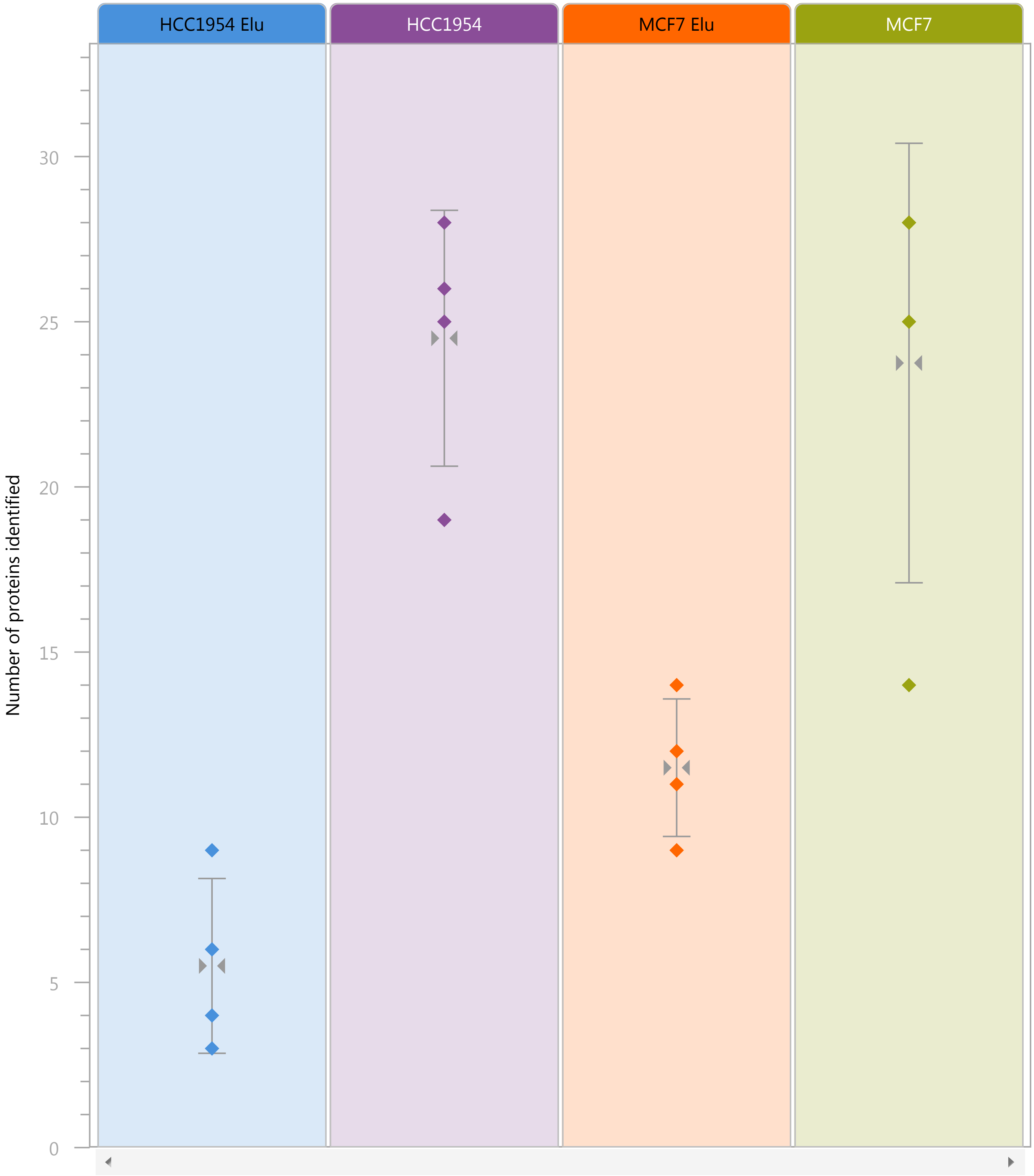

Peptides

The chart below shows the number of peptides identified in each of the experiment's runs. Each run is coloured according to its experimental condition.

Potential problems:

- Any exceptionally high or low values may indicate problems with sample preparation, chromatography, or instrument settings.

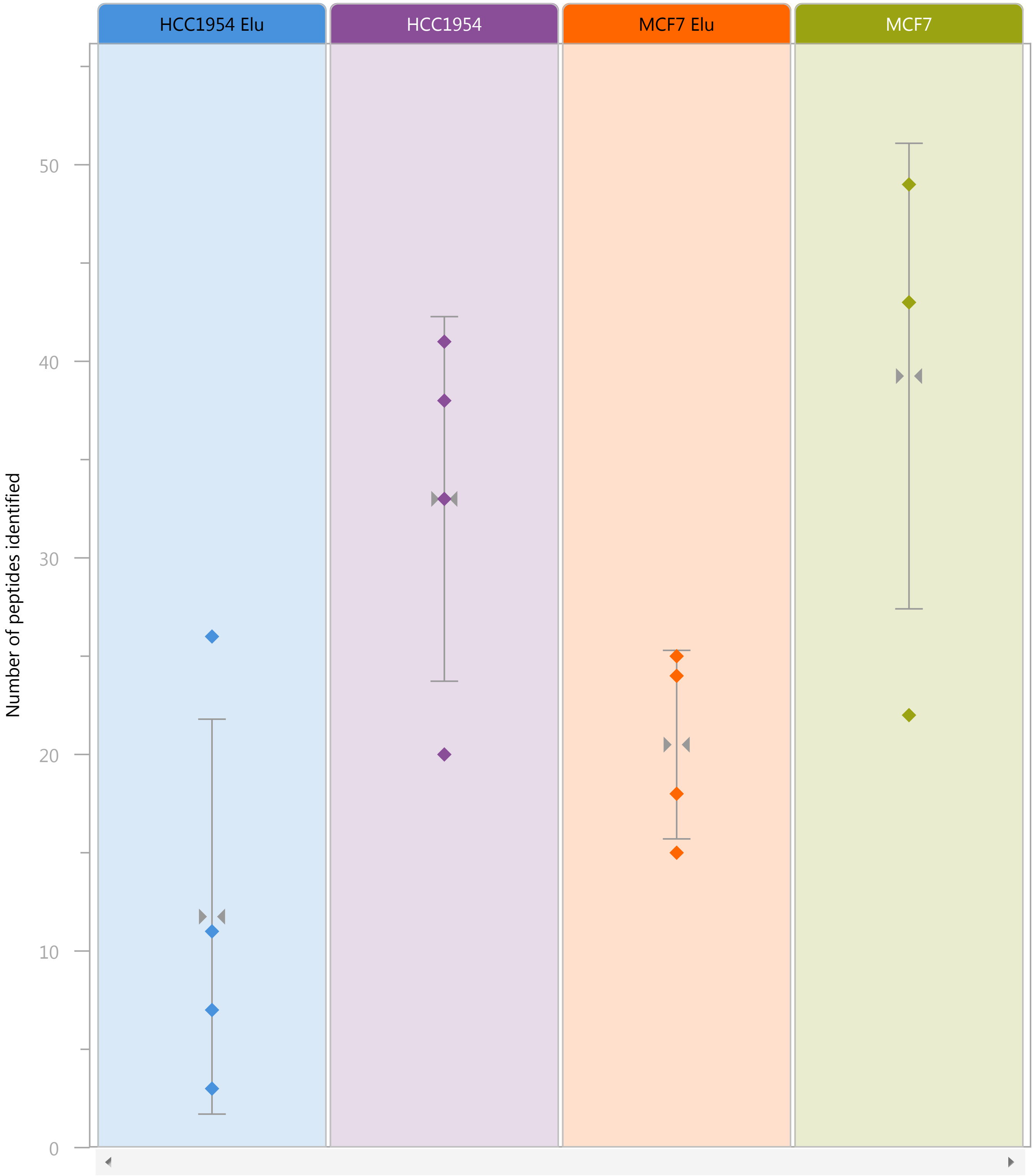

Peptides per protein (by condition)

The chart below shows the average number of peptides per protein in each run within the selected experiment design. For a given run, the value shown is calculated using only identifications from that run.

Potential problems:

- Any exceptionally high or low values may indicate problems with sample preparation or peptide search settings.

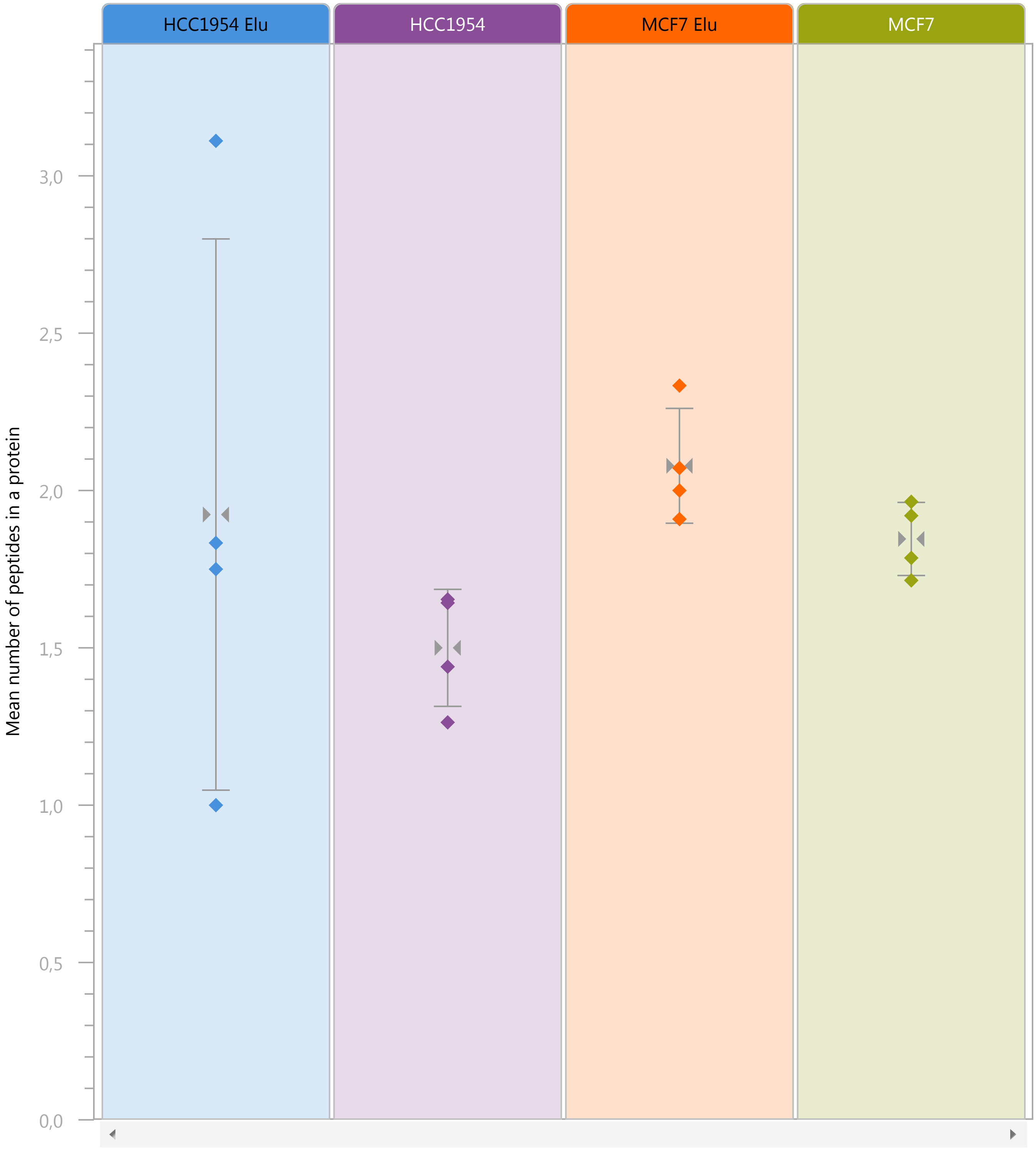

Percentage of peptide ions identified

The chart below shows the number of peptide ions identified in each of the runs within the selected experiment design, expressed as a percentage of the total number of peptide ions. Each run is coloured according to its experimental condition.

Potential problems:

- Any exceptionally high or low values may indicate problems with sample preparation, chromatography, or instrument settings.
- Very low values across all runs may indicate a problem with the database that was searched for identifications, or with other search parameters.

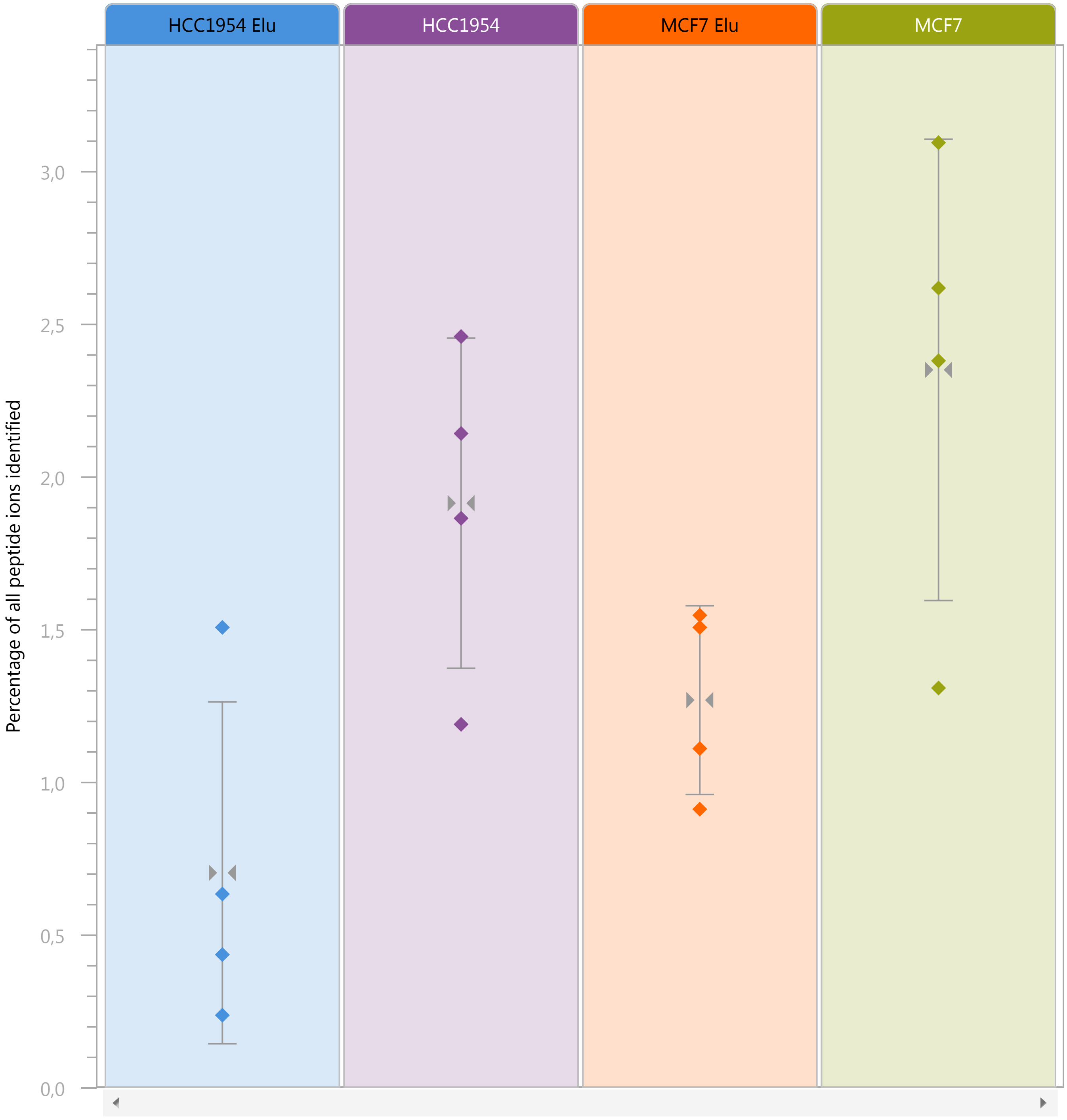

Protein overlap

The Venn diagram shows the number of proteins identified uniquely in the selected conditions and the number that are shared between conditions. Use the list on the right to select two or three conditions to explore.

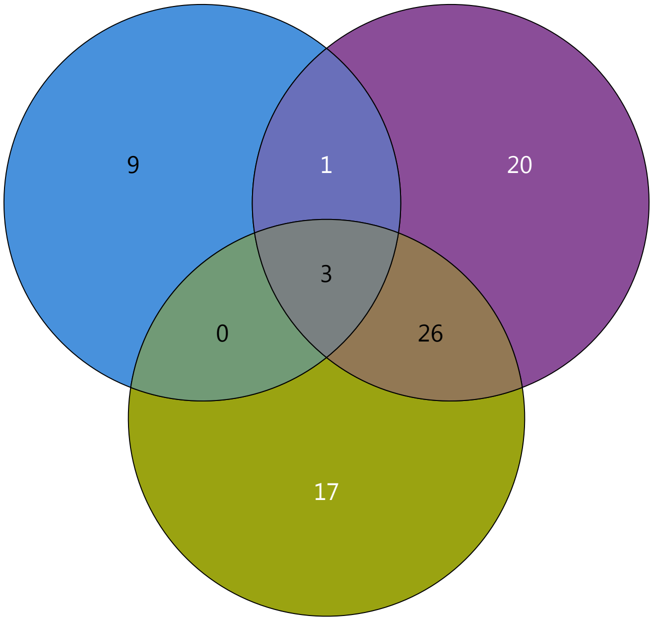

Select experimental conditions

- HCC1954 Elu
- HCC1954
- MCF7

HCC1954 X MCF7 STEP5

QC Metrics reported by Progenesis QI for proteomics v2.0.5556.29015
Date: 10-Jan-2020 at 09:30
Experiment design: HCC1954 X MCF7 STEP5

This QC report contains **all metrics** for the experiment named above.

Reported metrics

0 of the 19 metrics reported have been manually flagged for attention.

- Identifications overview
- LC peak width
- Peptide ion dynamic range
- Precursor m/z
- Precursor retention time
- Mass errors
- Precursor charges
- Missed cleavages (assuming trypsin)
- Peptides per protein (overview)
- Modifications
- Missed cleavages
- Abundance dynamic range
- Mass accuracy
- Scan rates
- Proteins
- Peptides
- Peptides per protein (by condition)
- Percentage of peptide ions identified
- Protein overlap

Identifications overview

The chart below provides the number of identifications per run by category. Data are plotted for all runs in the current experiment design. The box represents the interquartile range and the whiskers the maximum and minimum.

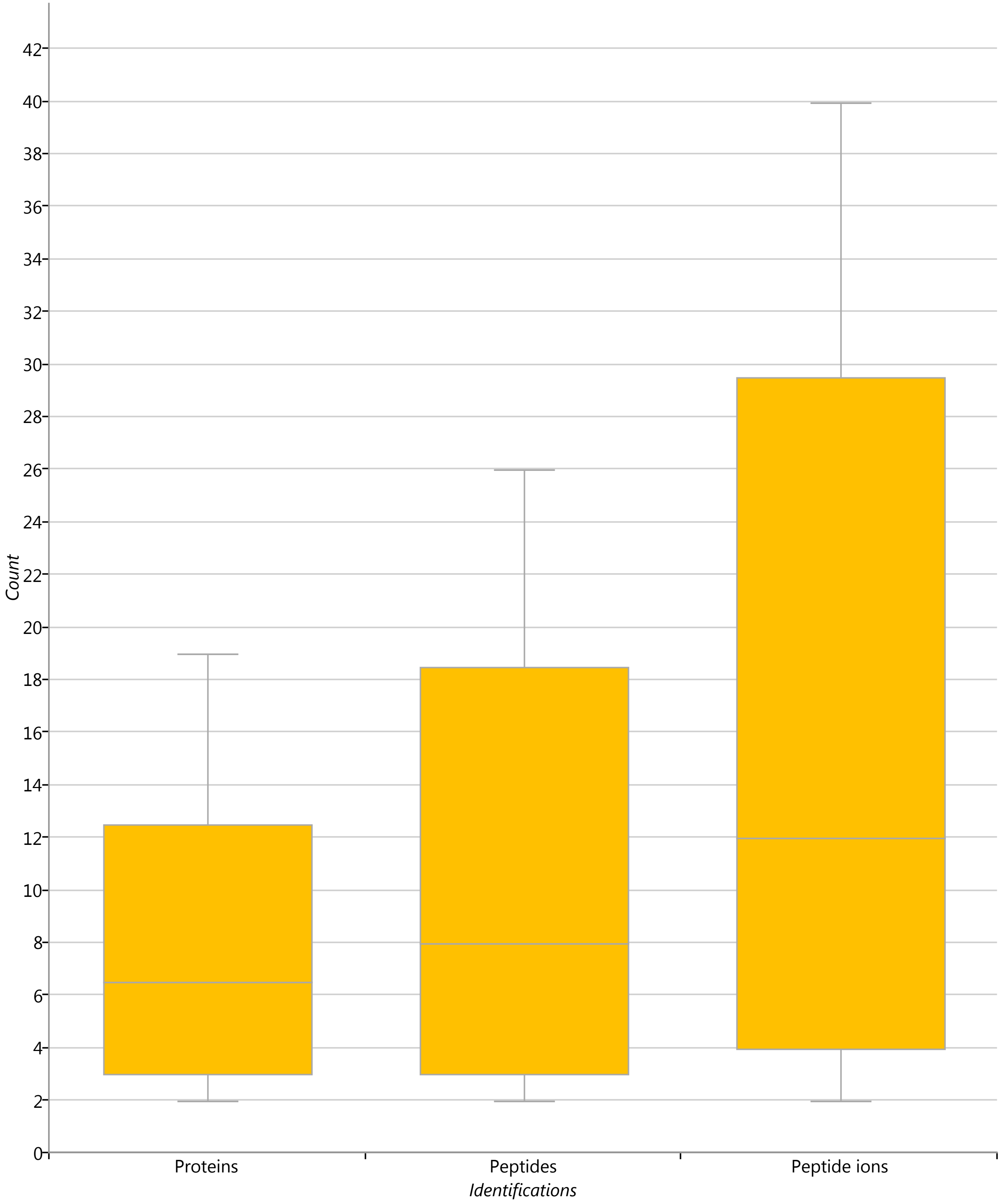

LC peak width

This chart shows the proportional distribution of peak widths in retention time, for all the detected peptide ions in the experiment. Widths are binned into categories for simple comparison. Identified peptide ions are shown in yellow.

Potential problems:

- A peak width that is different from that expected for your setup may indicate a problem with the chromatographic separation.

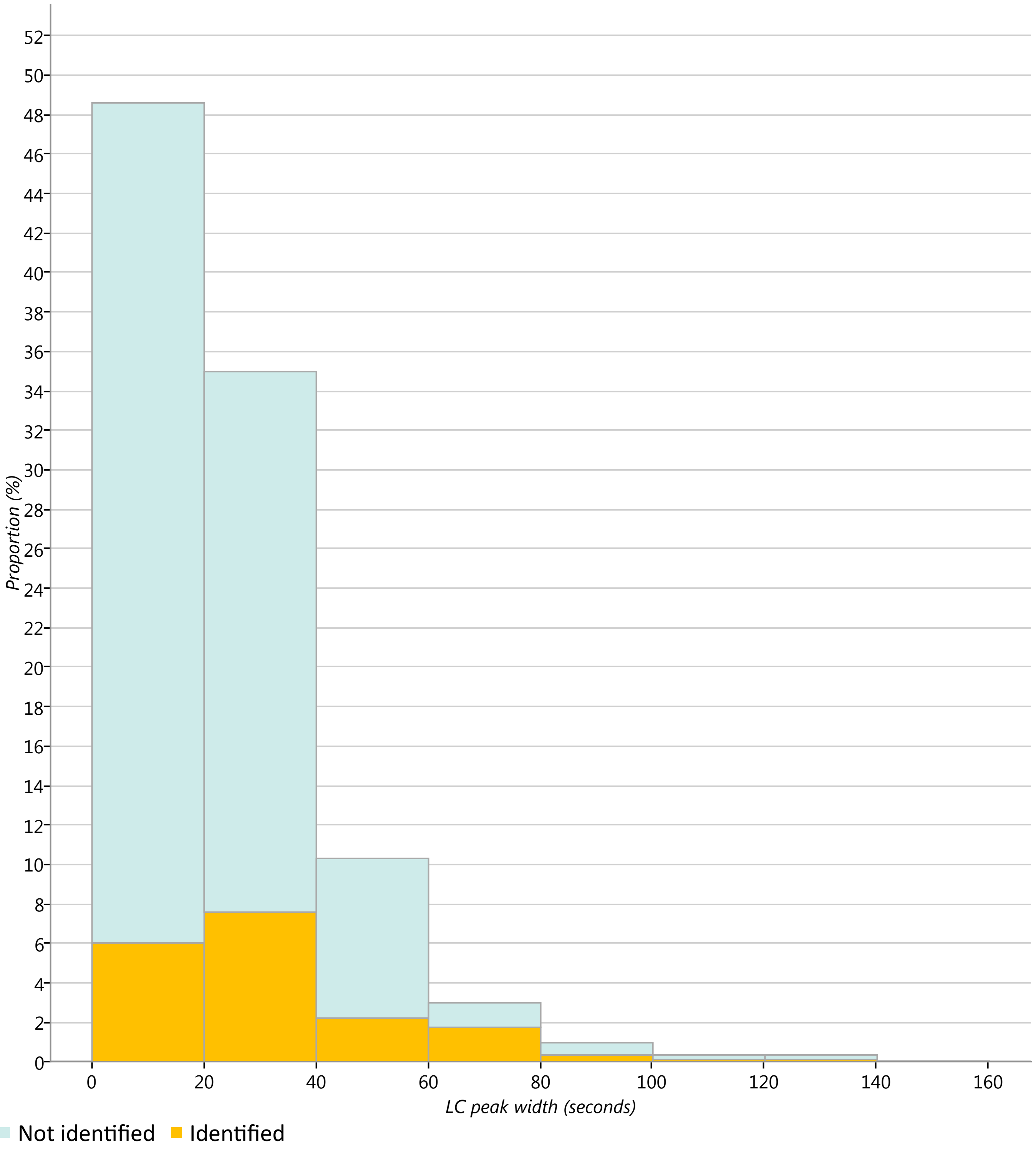

Peptide ion dynamic range

This chart shows log abundance for peptide ions, calculated using the current experiment design. Each peptide ion is assigned its abundance based on the highest mean value for any condition's runs. Data are binned and shown as a proportion. Identified peptide ions are shown in yellow.

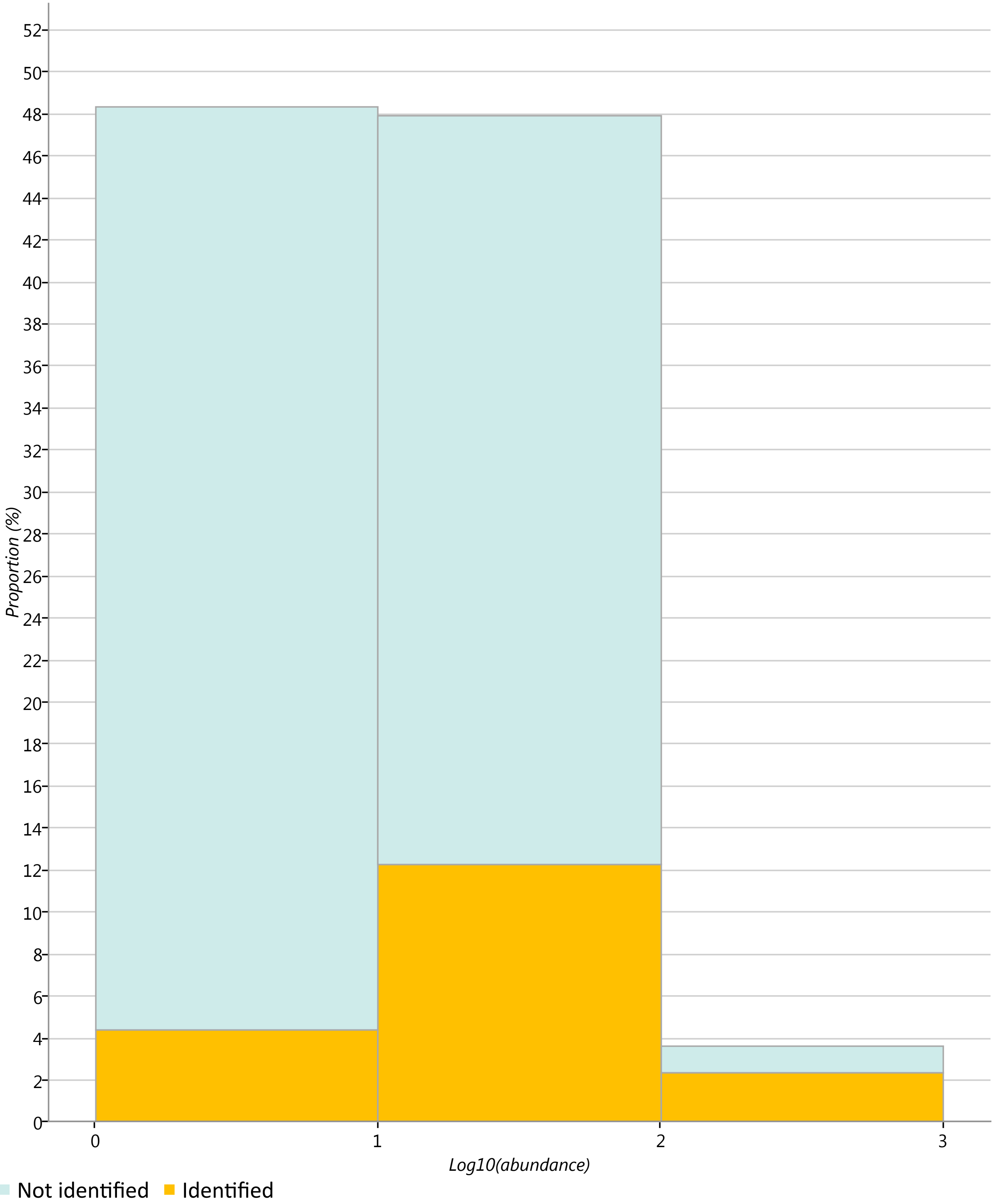

Precursor m/z

This chart shows the distribution of m/z values for all peptide ions in the experiment. Identified peptide ions are shown in yellow.

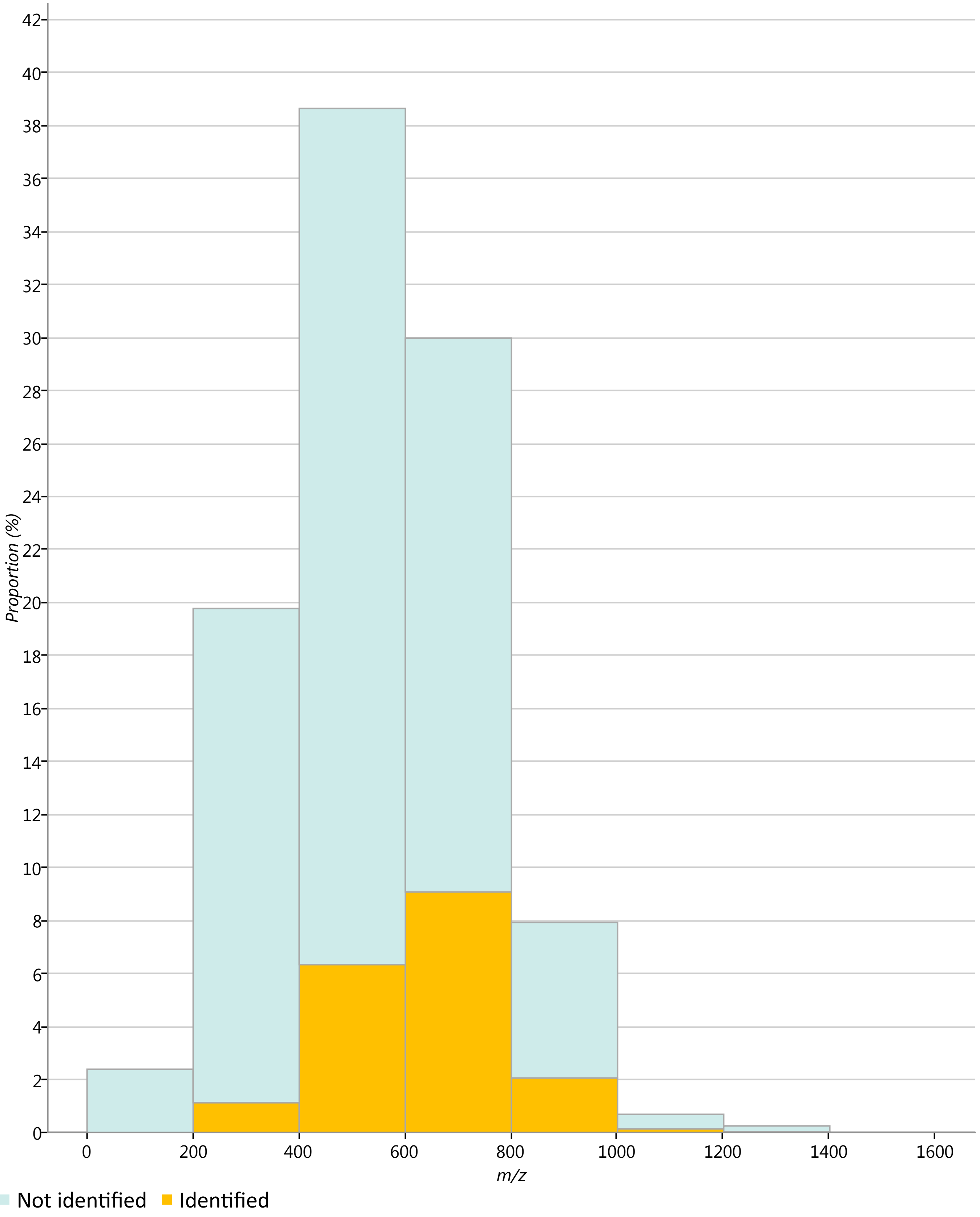

Precursor retention time

This chart shows the proportion of precursor peptide ions eluting into each quartile of the LC run. Identified peptide ions are shown in yellow.

Potential problems:

- A majority of peptide ions eluting at the start or end of the run may indicate problems with the chromatographic separation or that the setup is not ideal for a successful separation.

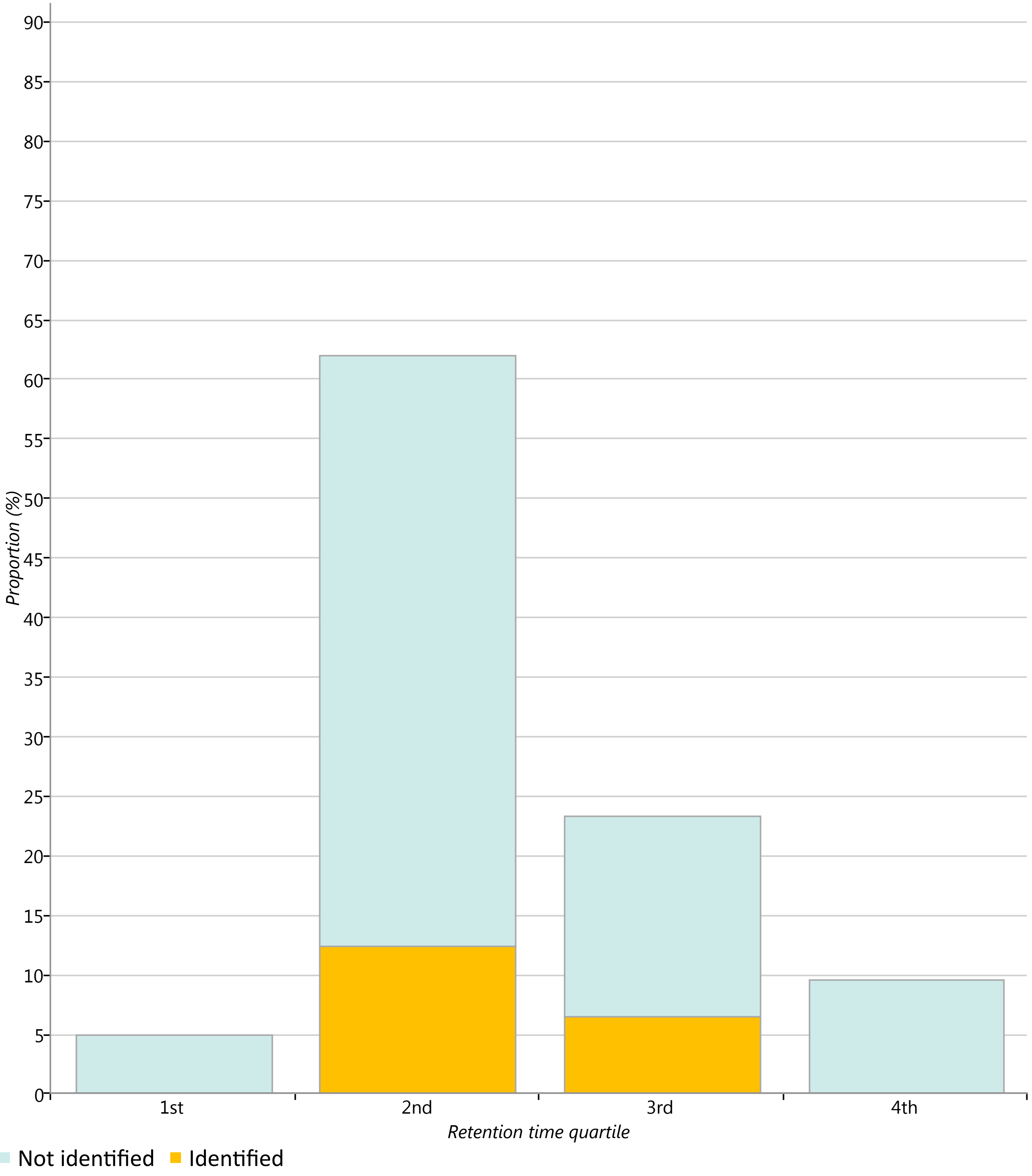

Mass errors

This chart shows the mass error calculated for identified peptide ions. The proportion of peptide ions with a given binned error value is plotted. For a more detailed plot, see the *Mass accuracy* metric.

Potential problems:

- A larger than expected spread could indicate that search parameters are too lenient.
- A bias of the peak from zero ppm could indicate instrument bias.

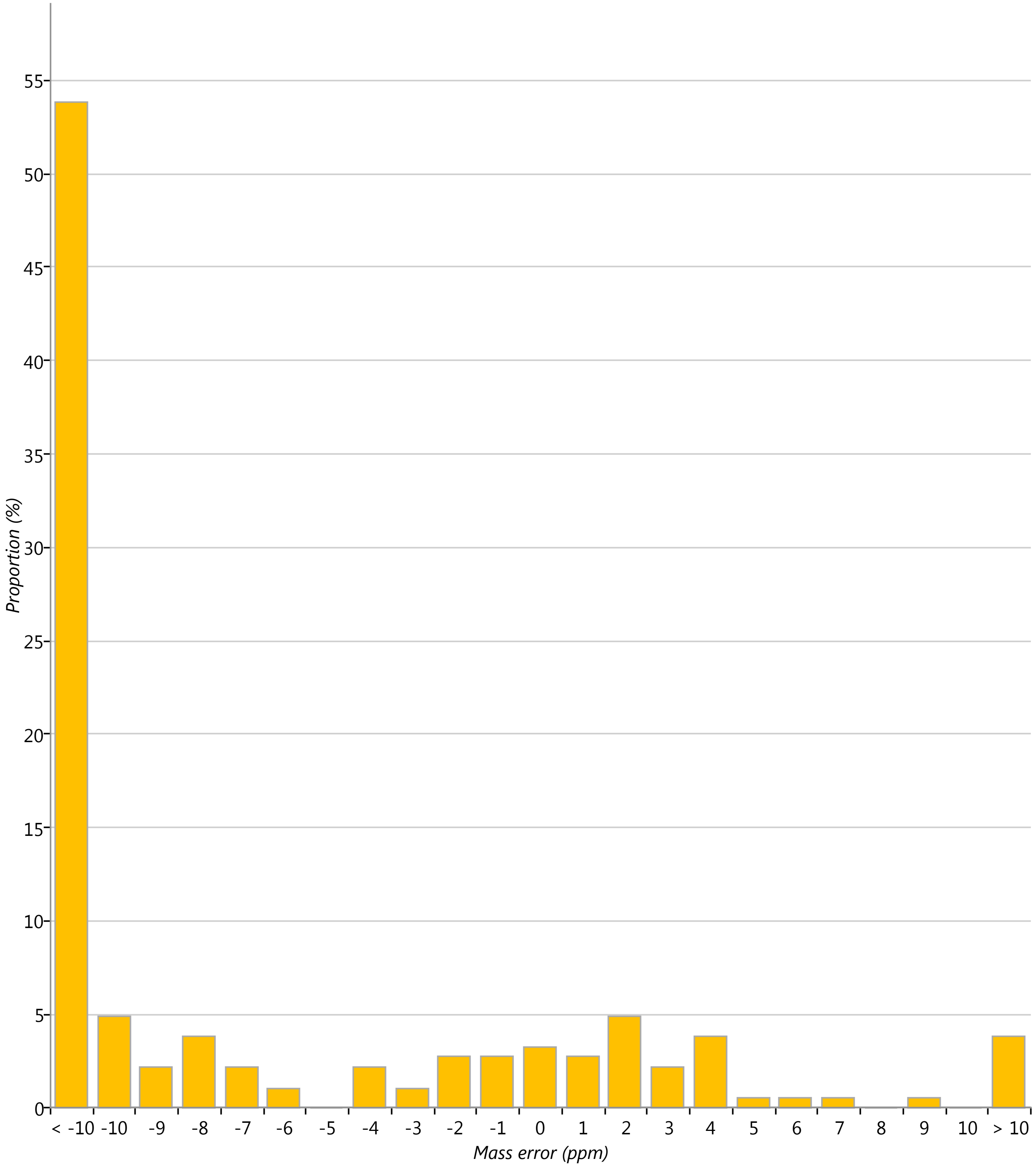

Precursor charges

This chart shows the percentage of peptide ions with various charge states.

Potential problems:

- A skew towards low charge peptide ions may indicate poor ionisation, suggesting that you may need to raise the collision voltage.
- A skew towards high charge peptide ions may indicate excessive ionisation, suggesting that you may need to lower the collision voltage.

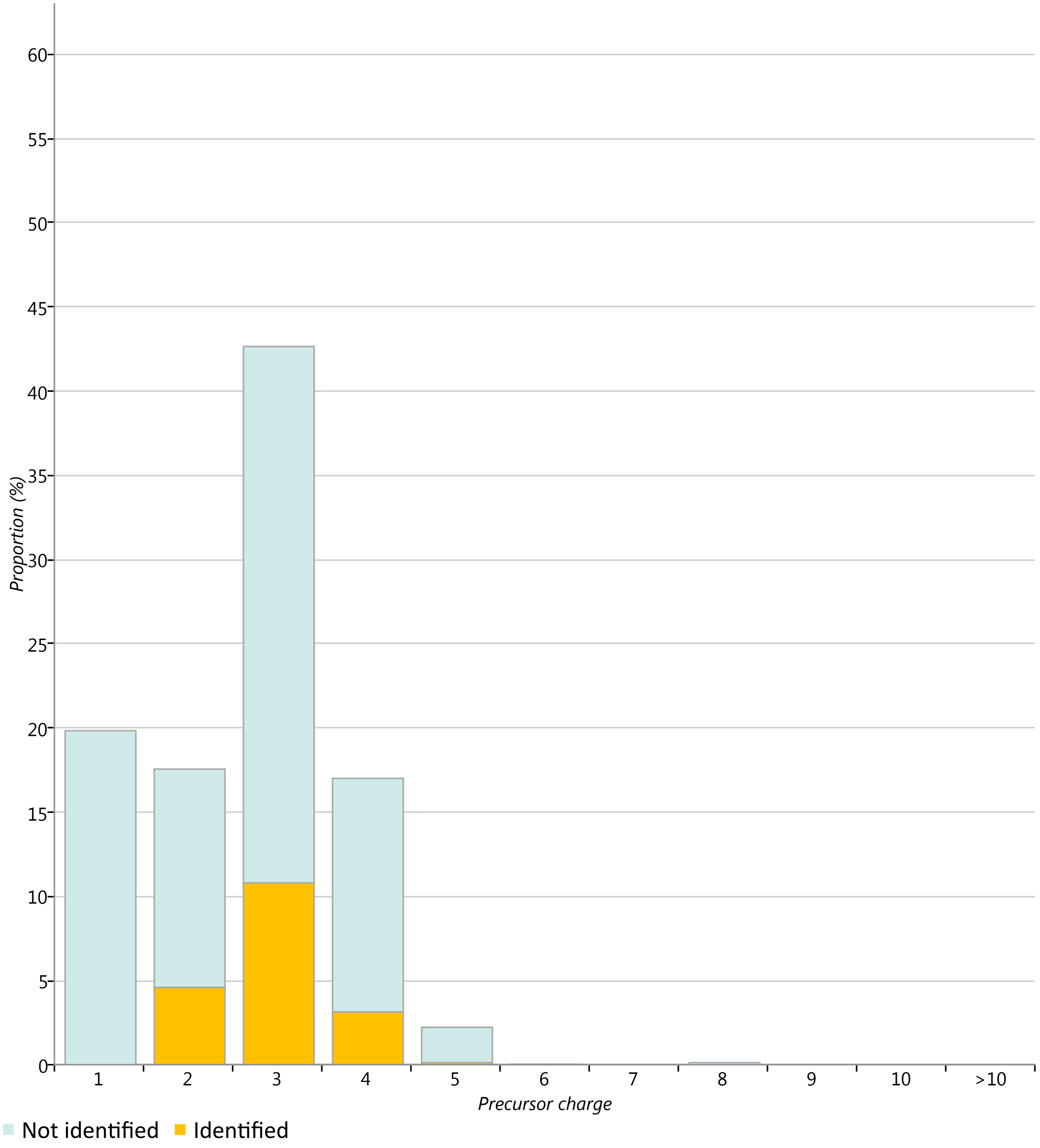

Missed cleavages (assuming trypsin)

This chart shows the distribution of missed cleavages per identified peptide ion across the whole experiment, assuming the proteins were treated with trypsin and using each peptide ion's highest scoring identification. For an alternative visualisation showing the data subdivided by experimental condition, see the *Missed cleavages* metric.

Potential problems:

- A high proportion of missed cleavages may indicate problems in trypsinization.
- An absence of missed cleavages may indicate that the database search parameters were too stringent.

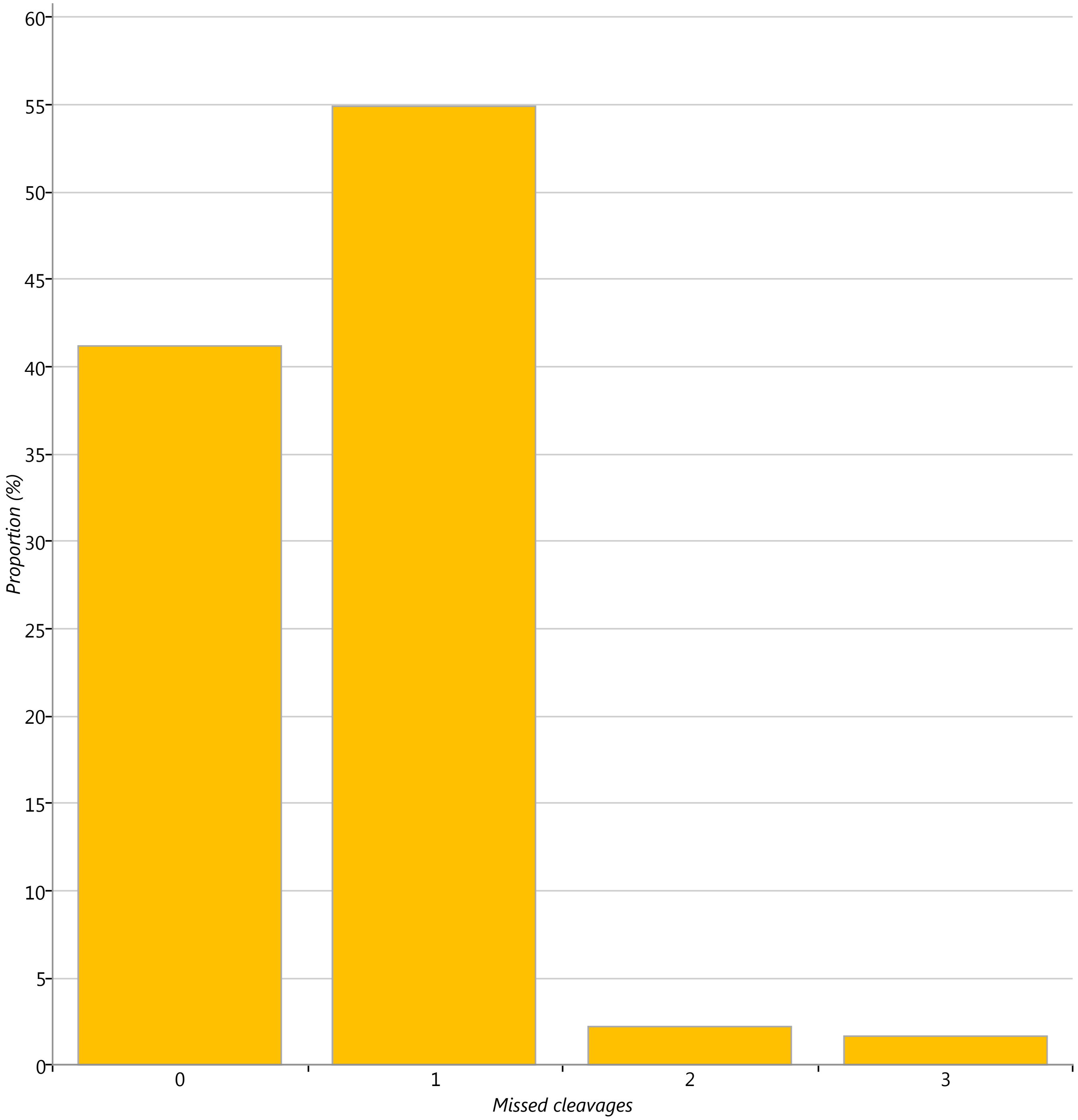

Peptides per protein (overview)

This chart shows the proportional distribution of the number of peptides identified per protein, combined across all runs in the experiment. For an alternative visualisation dividing these data by experimental condition, see the *Peptides per protein (by condition)* metric.

Potential problems:

- An unexpected bias to high or low numbers of peptides may indicate problems with sample preparation or peptide search settings.

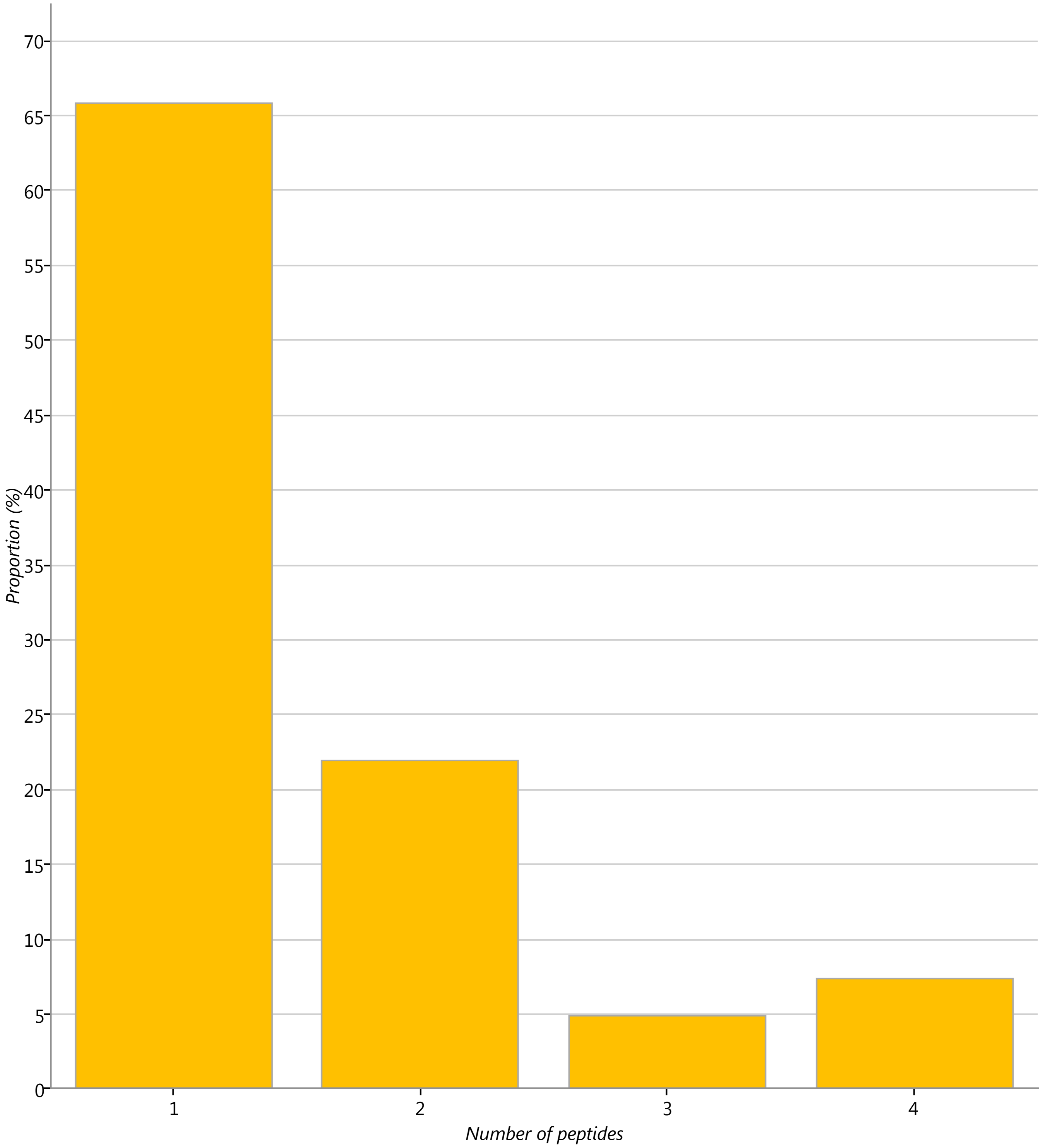

Modifications

The chart below shows the mean number of modifications per peptide ion for runs in the selected experiment design, based on each peptide ion's highest scoring identification. Each run is coloured according to its experimental condition.

Potential problems:

- Unexpectedly low numbers of modifications may indicate that one or more modifications were mistakenly not selected when searching for identifications.
- Variable numbers of modifications across the runs, especially within a given condition, may indicate problems with sample preparation.

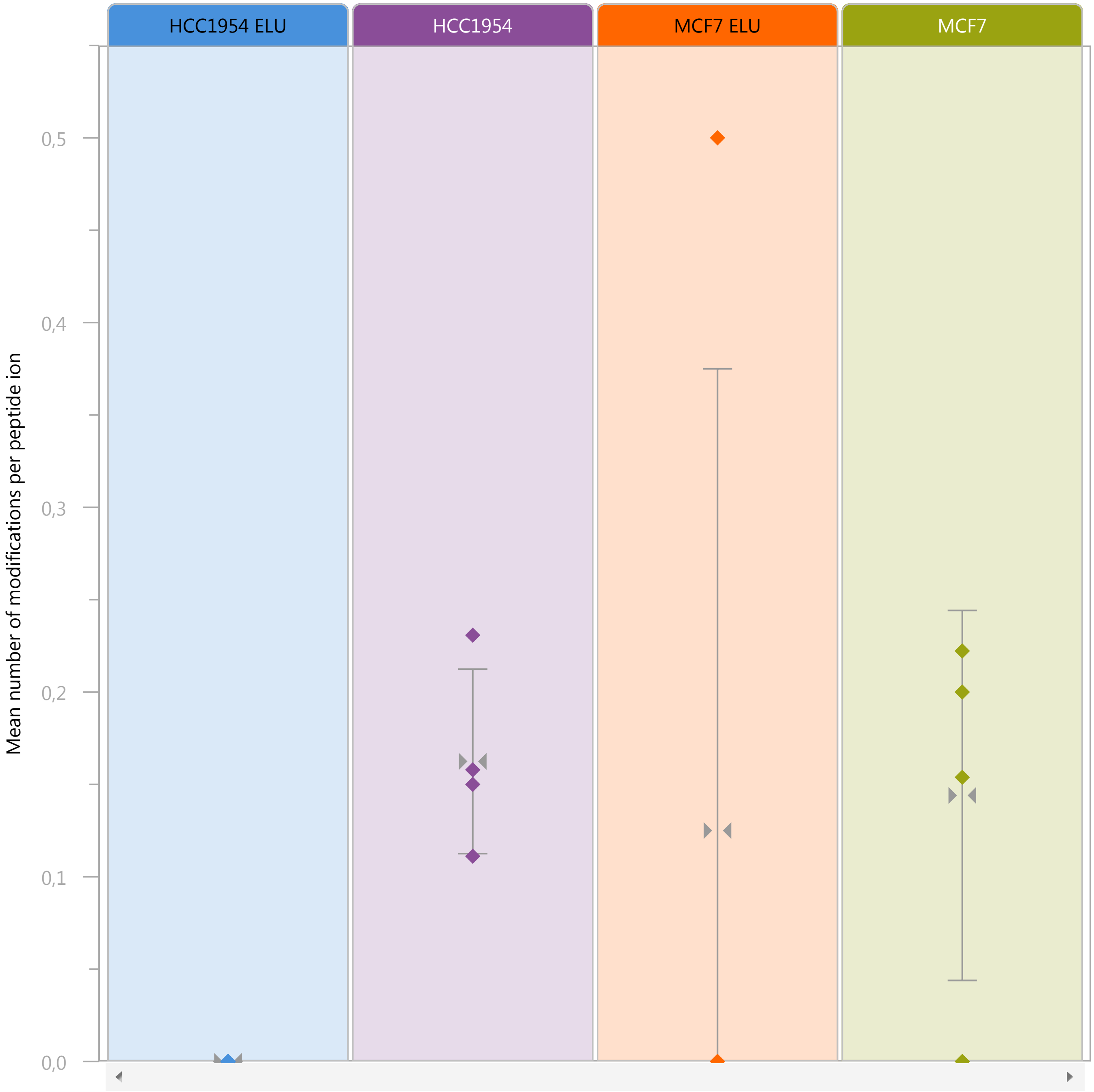

Included modifications Carbamidomethyl C Carbamyl N-TERM Methyl C-TERM Methyl N-TERM

Missed cleavages

The chart below shows the mean number of missed cleavages per peptide ion for runs within the selected experiment design, based on each peptide ion's highest scoring identification. The chart assumes the use of trypsin for digestion. Each run is coloured according to its experimental condition.

Potential problems:

- A high number of missed cleavages may indicate problems in trypsinization.
- An absence of missed cleavages may indicate that database search parameters were too stringent.

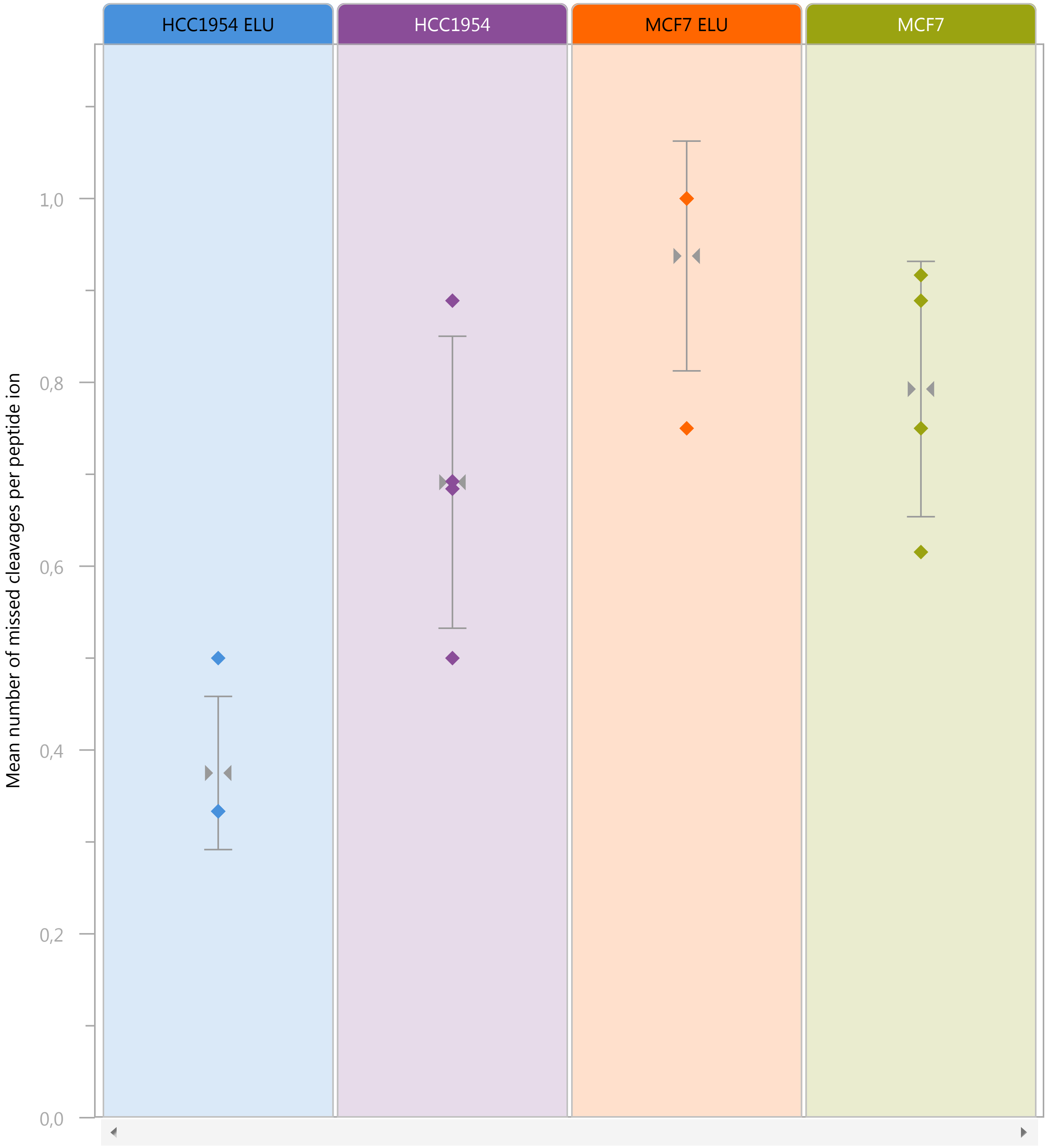

Abundance dynamic range

The charts below show abundance histograms for peptide ions, calculated using the current experiment design. Each chart shows the mean abundance for peptide ions within an experimental condition's runs. Data are binned by Log10 of their normalised abundance and shown as a proportion of the total. Identified peptide ions are shown in yellow.

Potential problems:

- A heavily right-skewed histogram of *identified* peptide ions indicates you may be missing identifications with low abundance.
- A heavily right skewed total (summed identified and unidentified) histogram may indicate saturation at high abundance, which will affect the accuracy of quantitation.

HCC1954 ELU

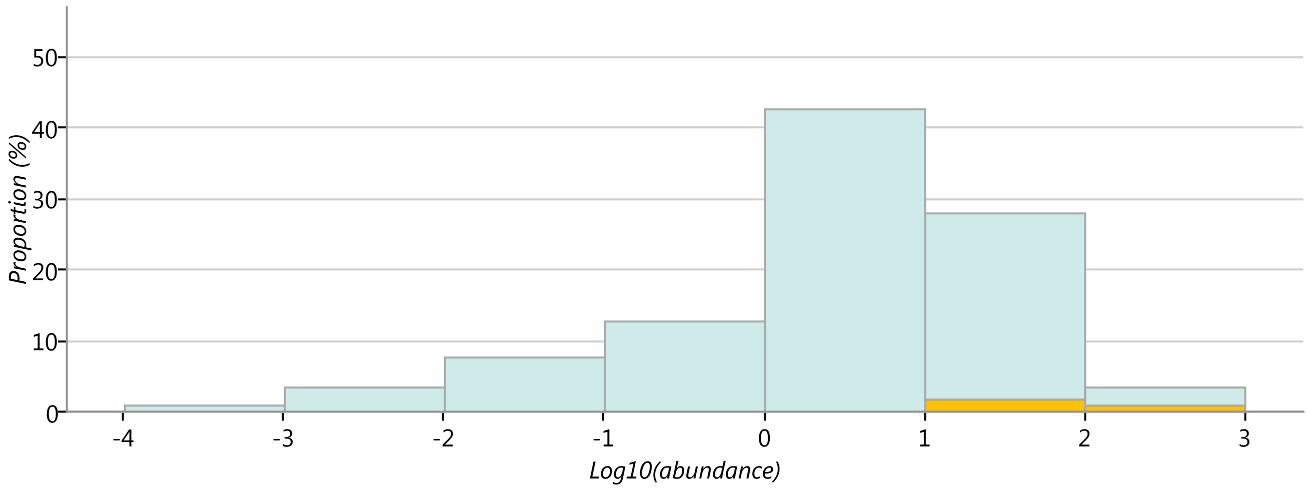

HCC1954

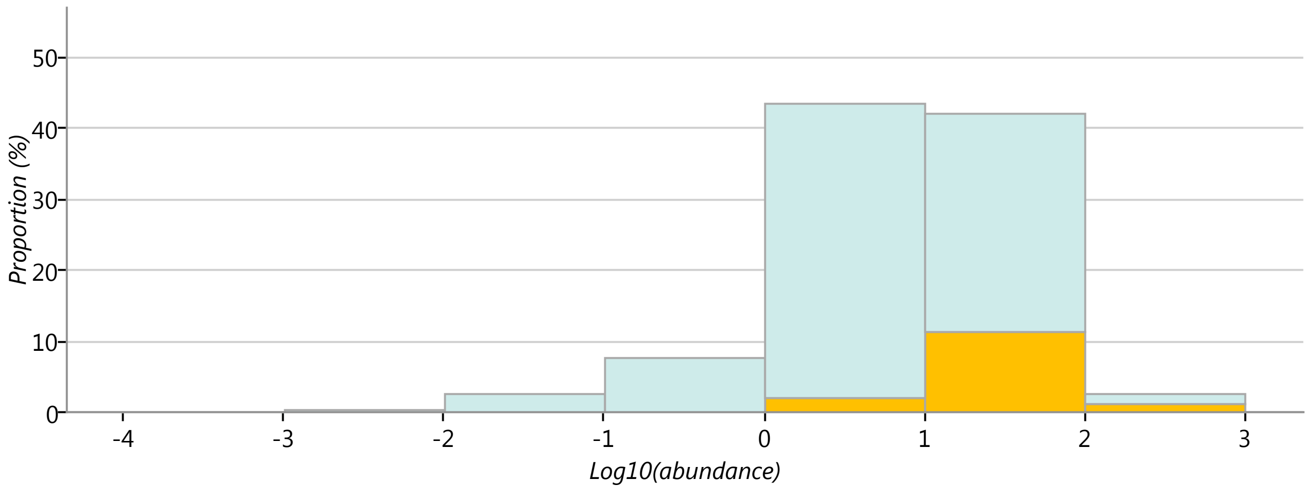

Not identified ■ Identified

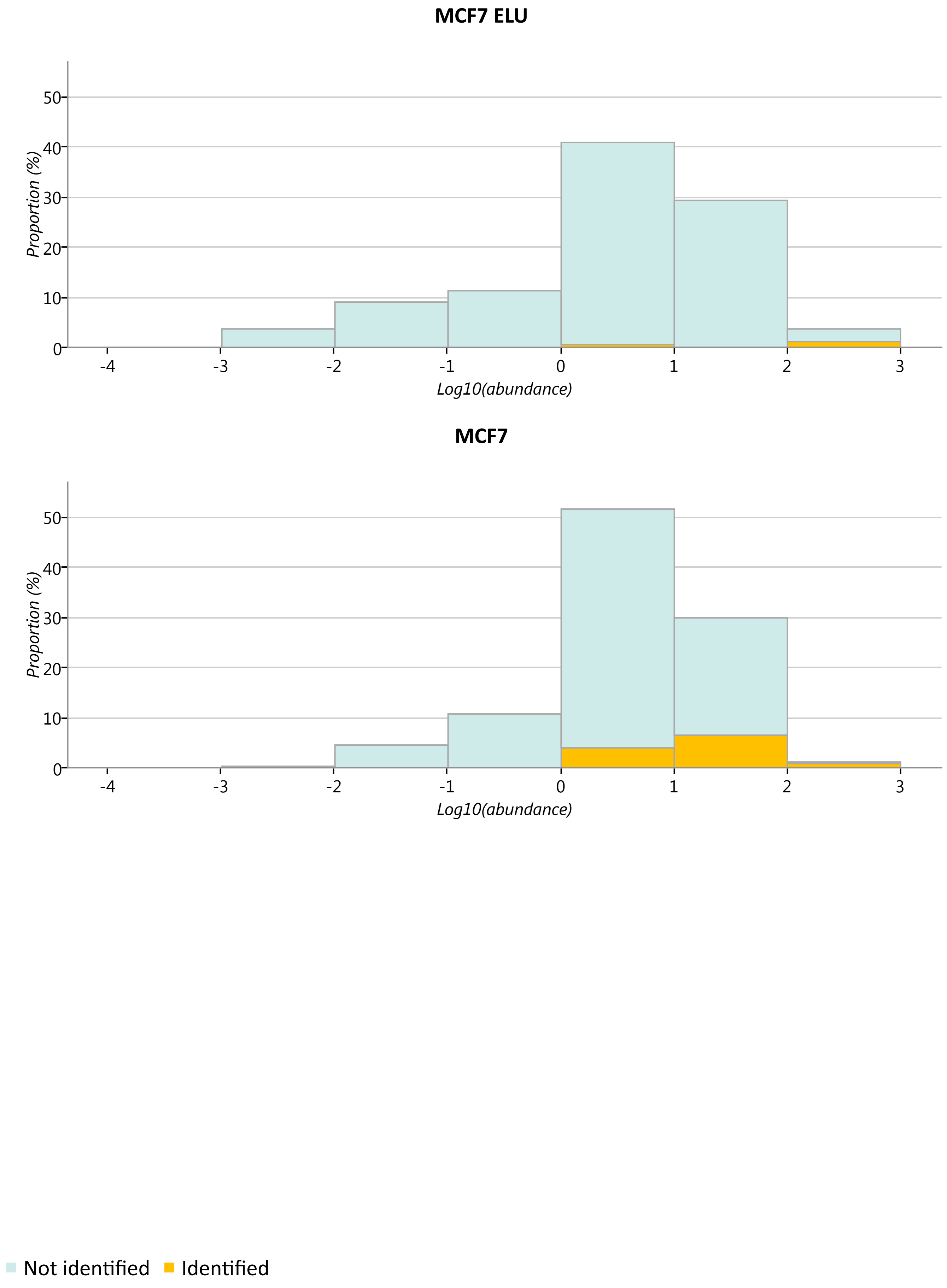
MCF7 ELU
Proportion (%)
0
10
20
30
40
50
-4
-3
-2
-1
0
1
2
3
Log10(abundance)
MCF7
Proportion (%)
0
10
20
30
40
50
-4
-3
-2
-1
0
1
2
3
Log10(abundance)
Not identified
Identified

Mass accuracy

The chart below shows all identified peptide ions in your experiment, plotting peptide ion m/z against the ppm error in its highest scoring identification. Blue points indicate peptide ions which have a mass error within 2 standard deviations of the mean, yellow points indicate peptide ions with a mass error more than 2 standard deviations from the mean.

Potential problems:

- A dependence of ppm mass error on m/z may indicate poor calibration.
- If the standard deviation of the data is large in ppm terms, this may indicate that your search parameters are too lenient.

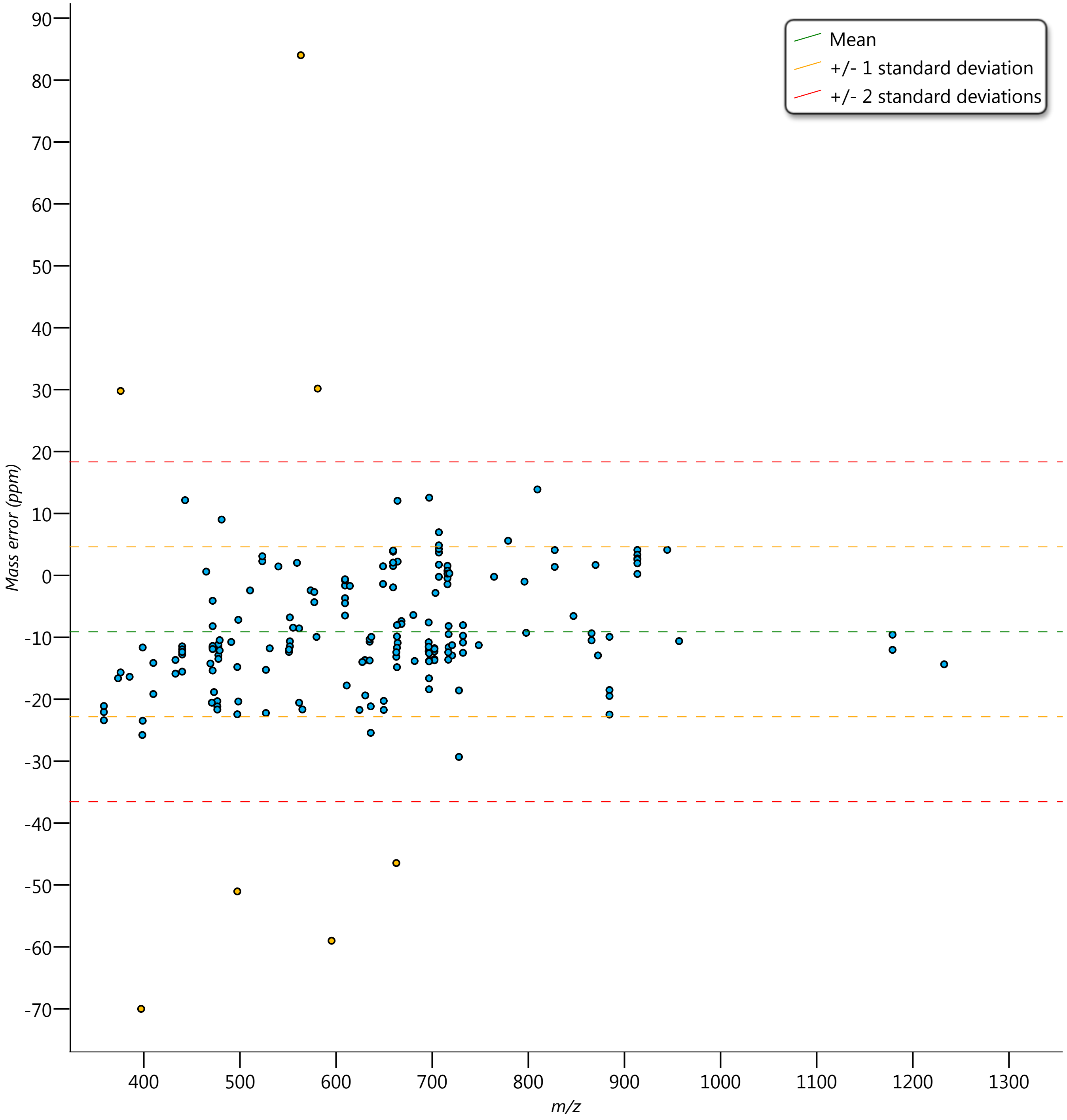

Scan rates

The charts below show the MS1 scan rate over the duration of your runs, using the current experiment design. Each chart shows the scan rate variation within an experimental condition's runs. The named run is highlighted in black. The variability between runs is shown as a grey area. The y-axis shows scans per second in a 30-second window centred on each x-axis time point.

Potential problems:

- A highly varying scan rate means quantitation accuracy may be negatively affected.

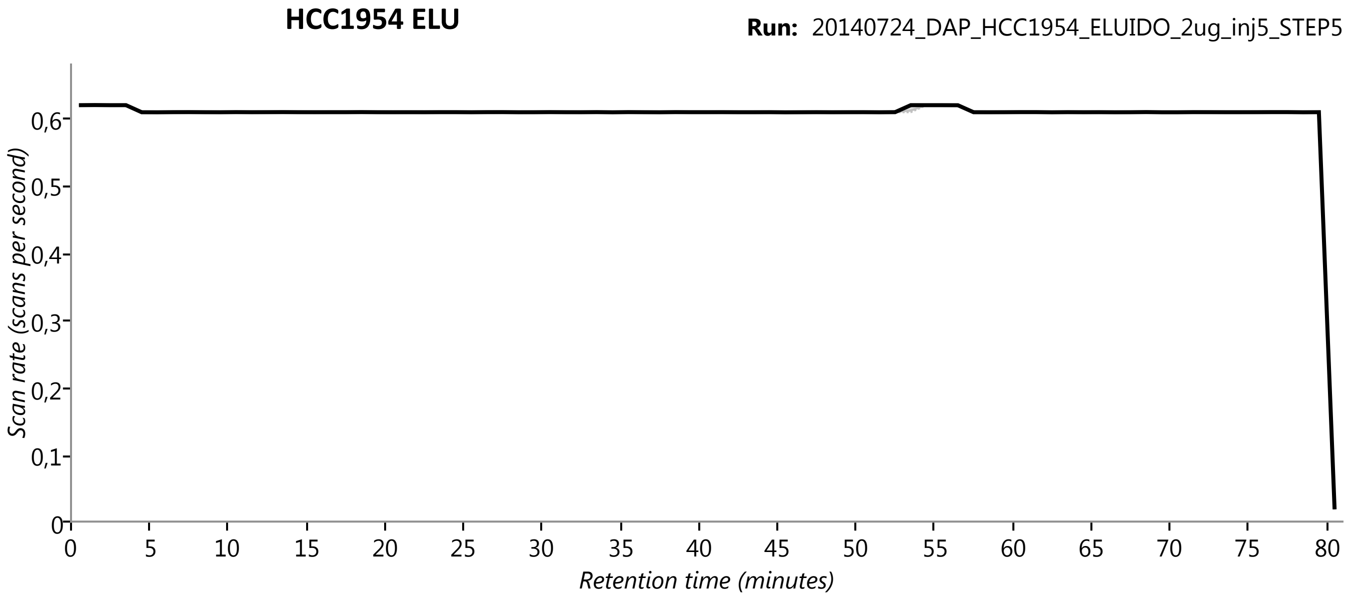

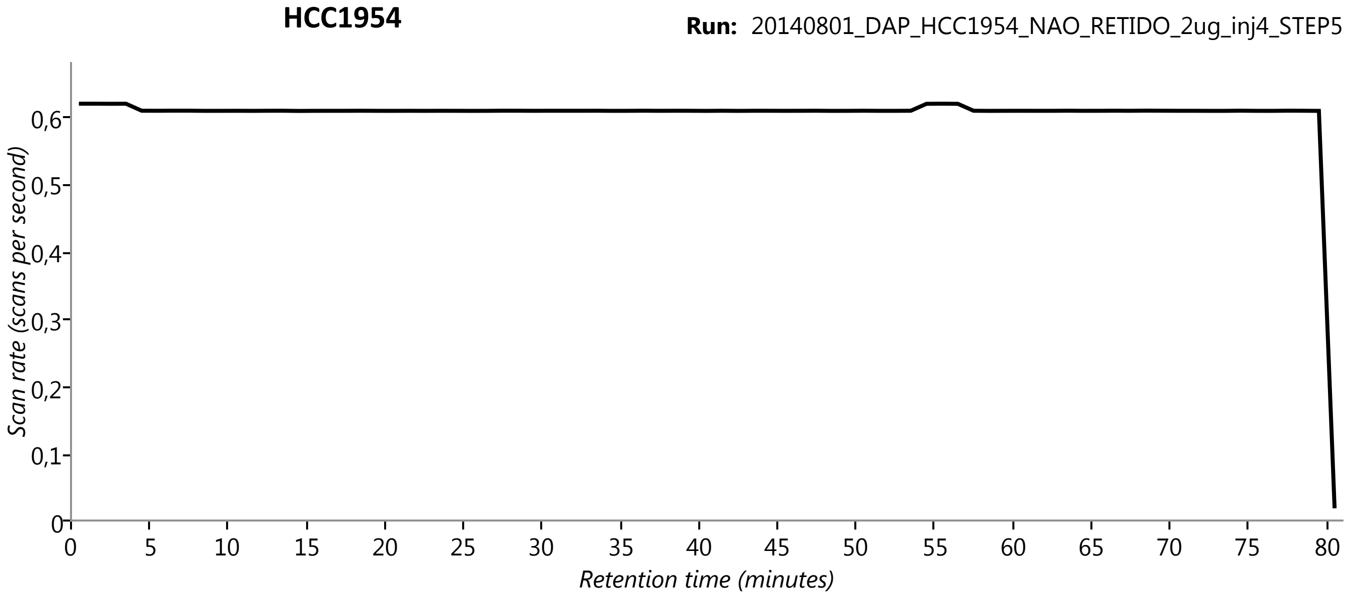

MCF7 ELU

Run: 20140724_DAP_MCF7_ELUIDO_2ug_inj3_STEP5

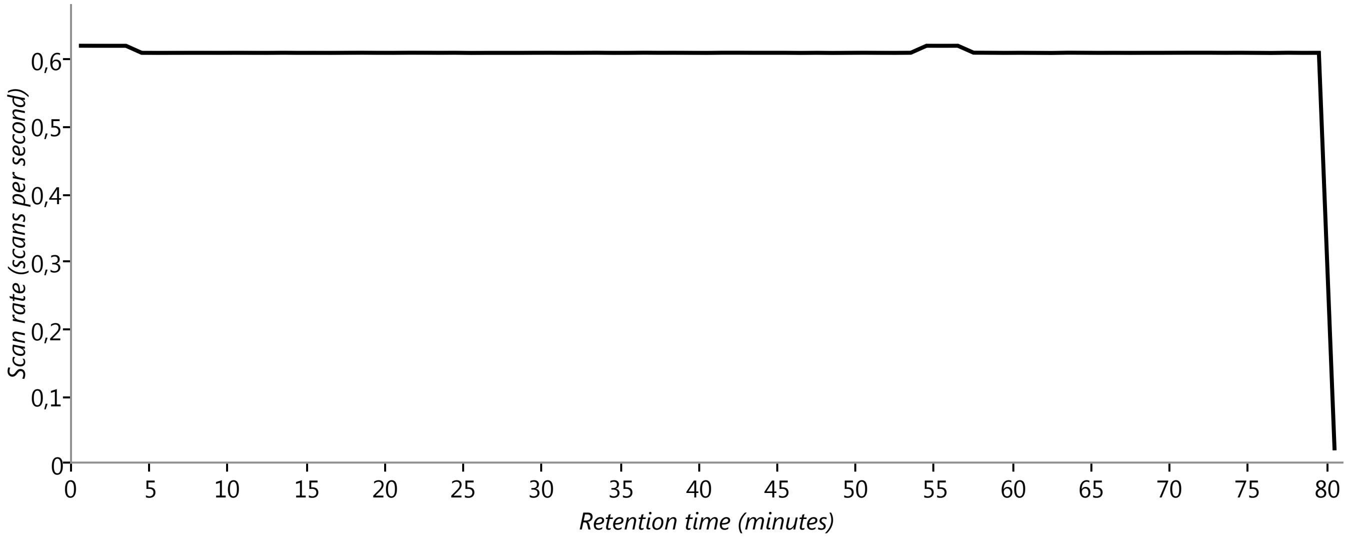

MCF7

Run: 20140801_DAP_MCF7_NAO_RETIDO_2ug_inj2_STEP5

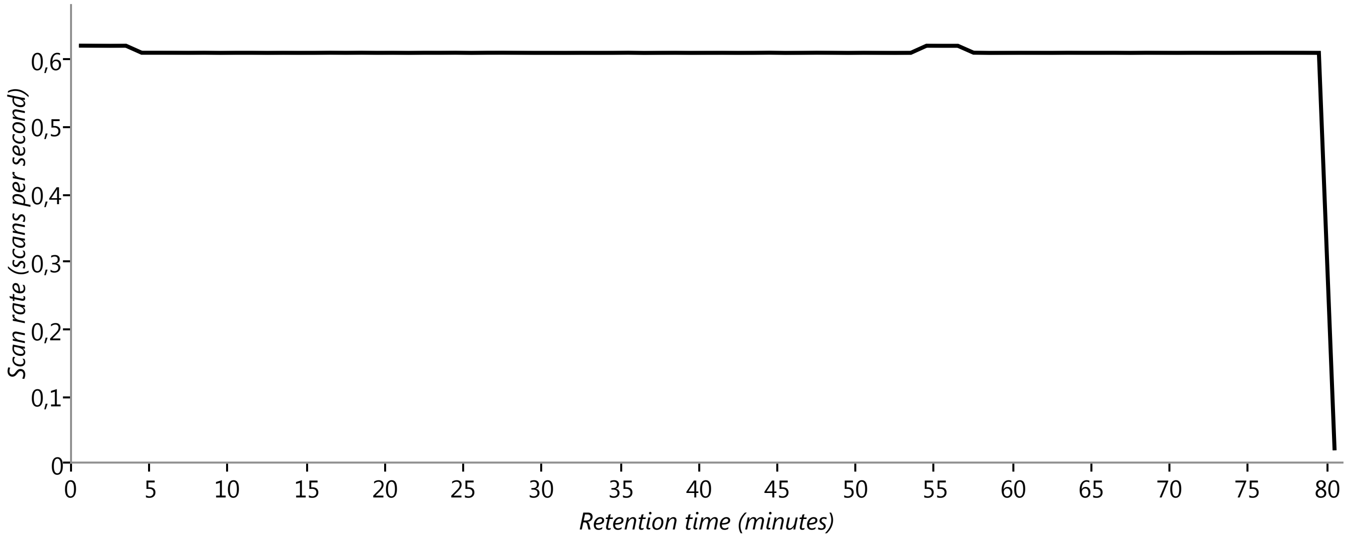

Proteins

The chart below shows the number of proteins identified in each of the runs within the selected experiment design. Each run is coloured according to its experimental condition.

Potential problems:

- Any exceptionally high or low values may indicate problems with sample preparation, chromatography, or instrument settings.

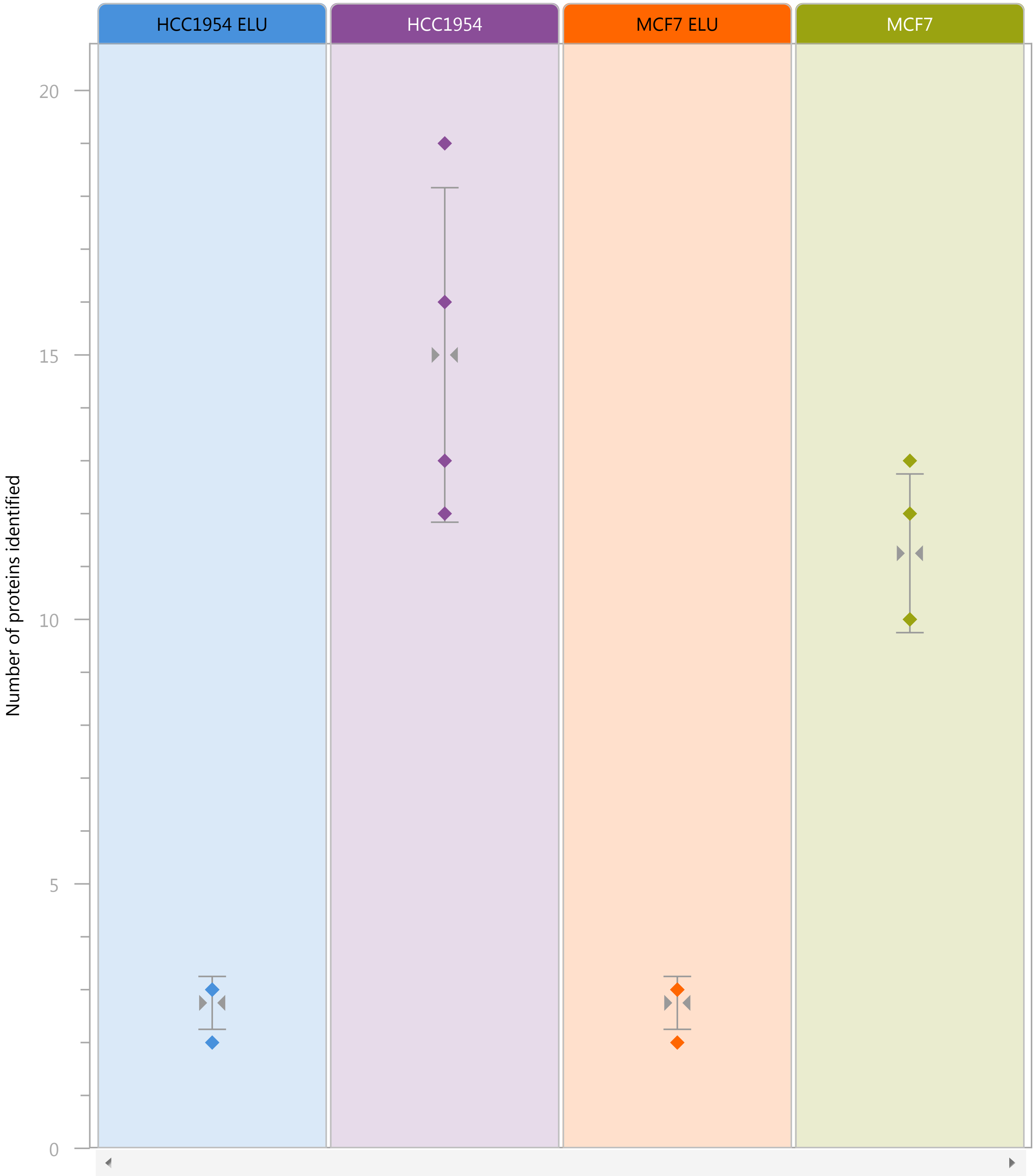

Peptides

The chart below shows the number of peptides identified in each of the experiment's runs. Each run is coloured according to its experimental condition.

Potential problems:

- Any exceptionally high or low values may indicate problems with sample preparation, chromatography, or instrument settings.

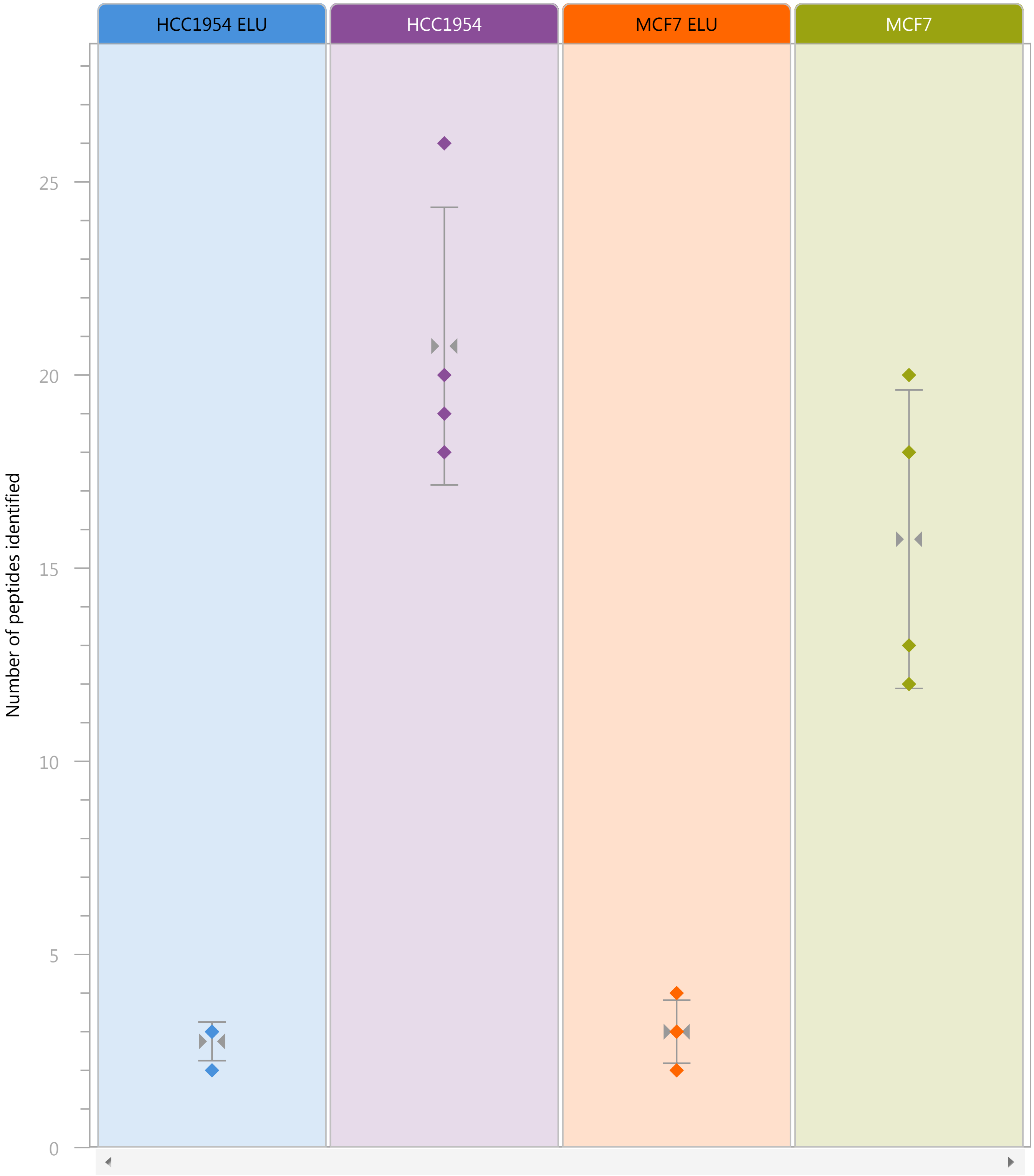

Peptides per protein (by condition)

The chart below shows the average number of peptides per protein in each run within the selected experiment design. For a given run, the value shown is calculated using only identifications from that run.

Potential problems:

- Any exceptionally high or low values may indicate problems with sample preparation or peptide search settings.

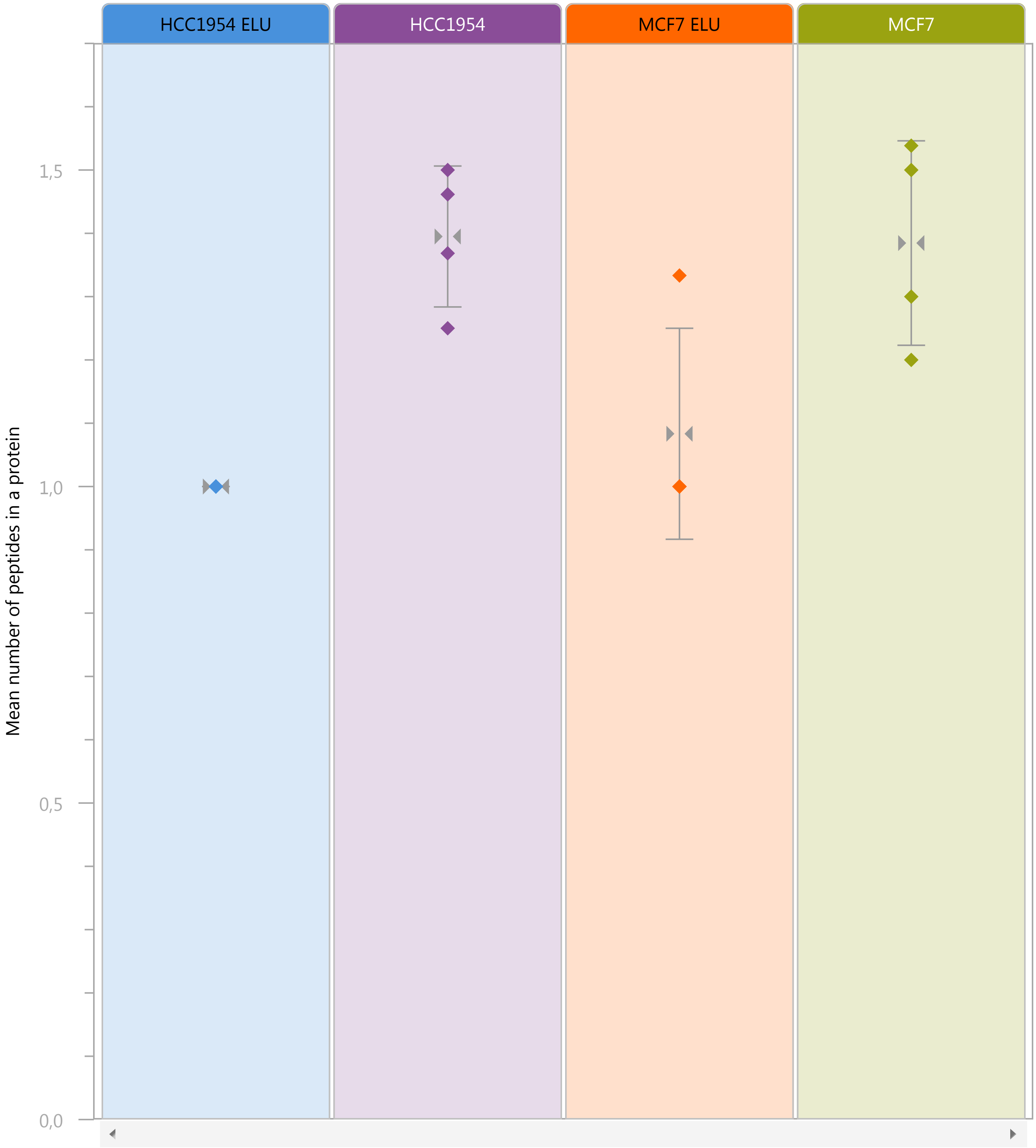

Percentage of peptide ions identified

The chart below shows the number of peptide ions identified in each of the runs within the selected experiment design, expressed as a percentage of the total number of peptide ions. Each run is coloured according to its experimental condition.

Potential problems:

- Any exceptionally high or low values may indicate problems with sample preparation, chromatography, or instrument settings.
- Very low values across all runs may indicate a problem with the database that was searched for identifications, or with other search parameters.

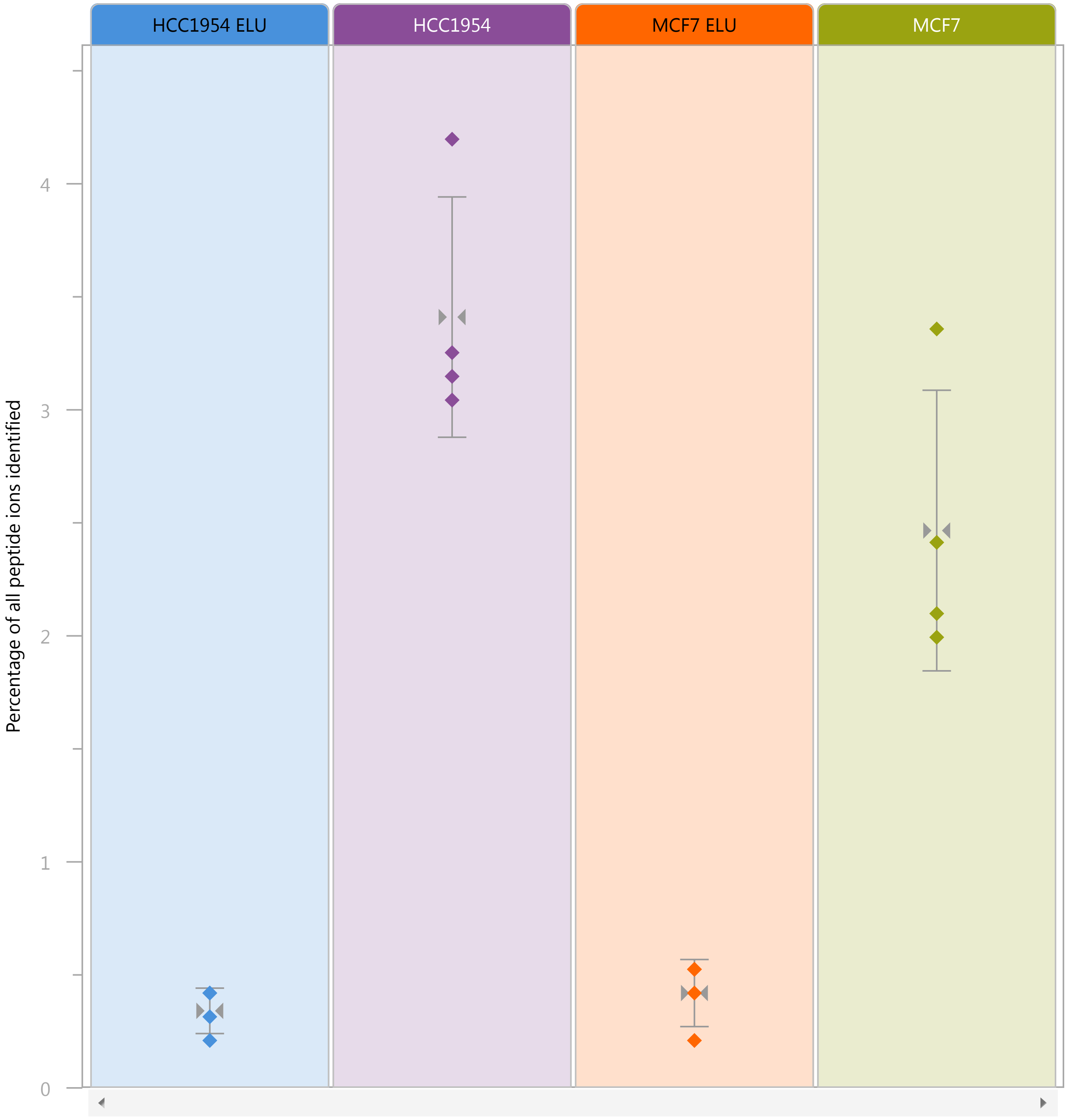

Protein overlap

The Venn diagram shows the number of proteins identified uniquely in the selected conditions and the number that are shared between conditions. Use the list on the right to select two or three conditions to explore.

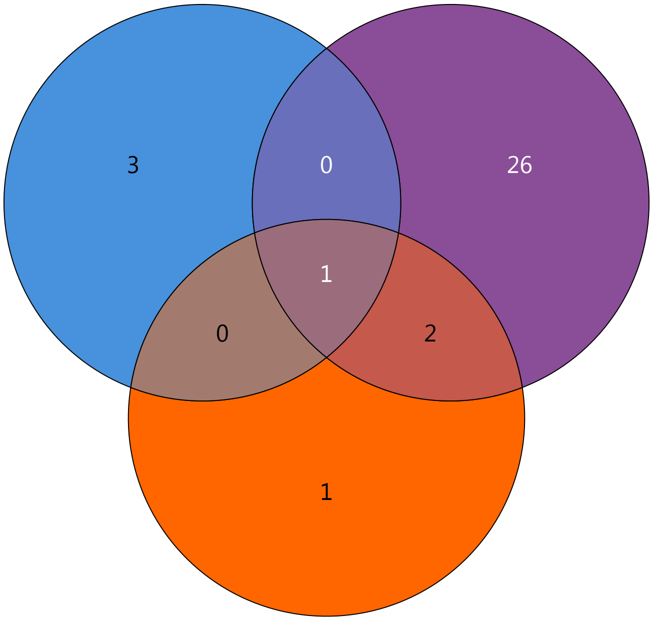

Select experimental conditions

- HCC1954 ELU
- HCC1954
- MCF7 ELU

HCC1954 X MCF7 STEP6

QC Metrics reported by Progenesis QI for proteomics v2.0.5556.29015
Date: 10-Jan-2020 at 09:33
Experiment design: HCC1954 X MCF7 STEP6

This QC report contains **all metrics** for the experiment named above.

Reported metrics

0 of the 19 metrics reported have been manually flagged for attention.

- Identifications overview
- LC peak width
- Peptide ion dynamic range
- Precursor m/z
- Precursor retention time
- Mass errors
- Precursor charges
- Missed cleavages (assuming trypsin)
- Peptides per protein (overview)
- Modifications
- Missed cleavages
- Abundance dynamic range
- Mass accuracy
- Scan rates
- Proteins
- Peptides
- Peptides per protein (by condition)
- Percentage of peptide ions identified
- Protein overlap

Identifications overview

The chart below provides the number of identifications per run by category. Data are plotted for all runs in the current experiment design. The box represents the interquartile range and the whiskers the maximum and minimum.

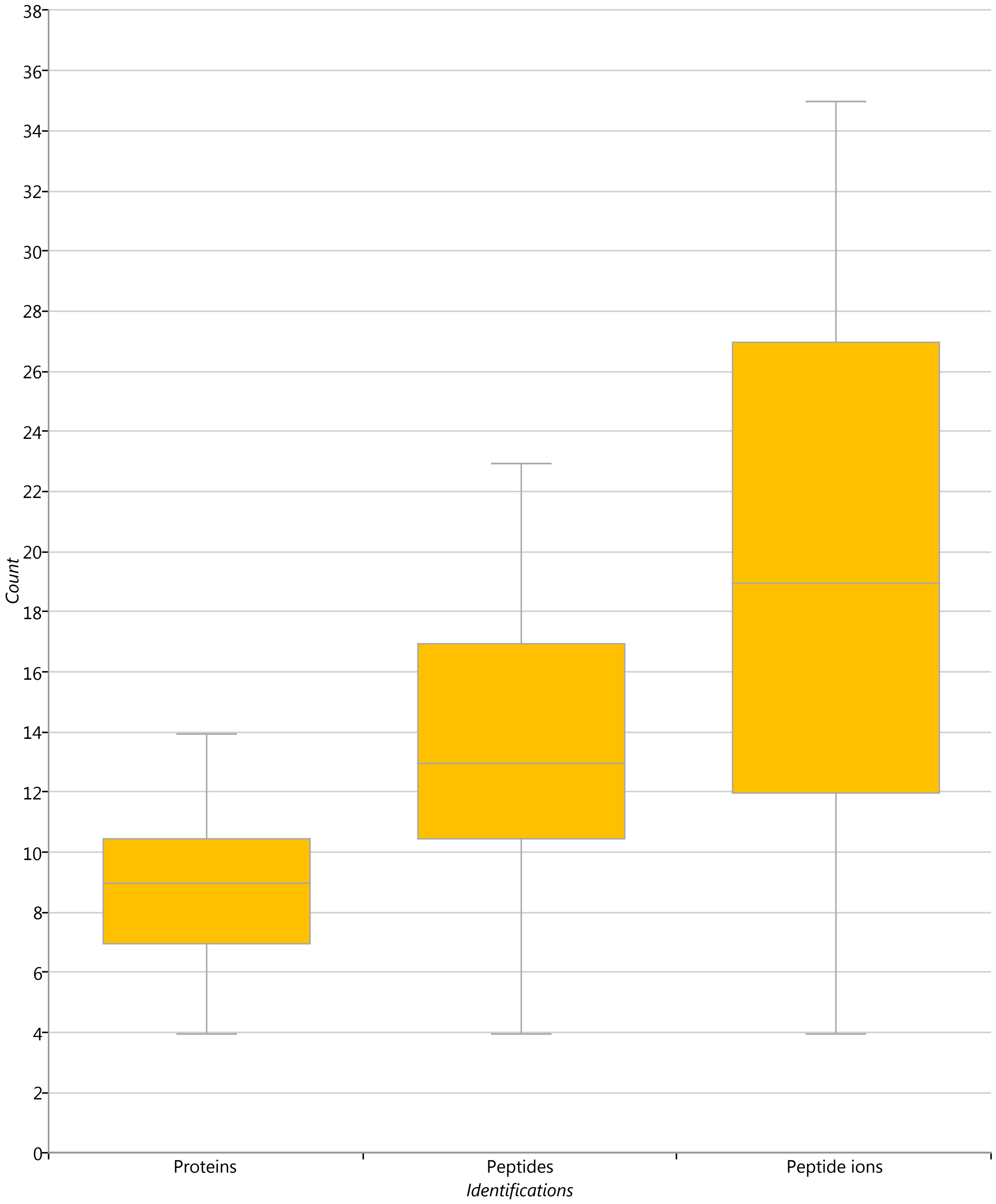

LC peak width

This chart shows the proportional distribution of peak widths in retention time, for all the detected peptide ions in the experiment. Widths are binned into categories for simple comparison. Identified peptide ions are shown in yellow.

Potential problems:

- A peak width that is different from that expected for your setup may indicate a problem with the chromatographic separation.

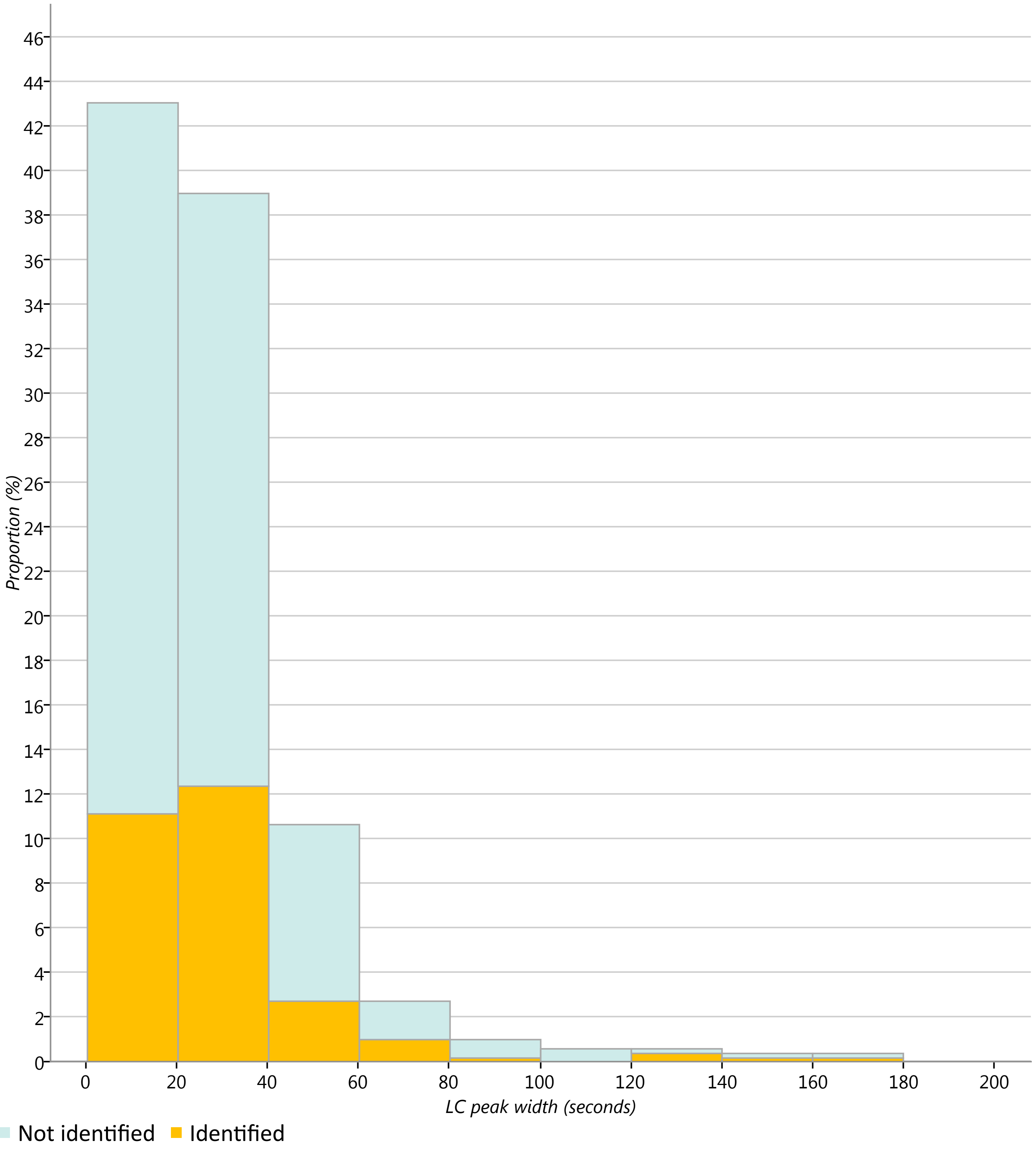

Peptide ion dynamic range

This chart shows log abundance for peptide ions, calculated using the current experiment design. Each peptide ion is assigned its abundance based on the highest mean value for any condition's runs. Data are binned and shown as a proportion. Identified peptide ions are shown in yellow.

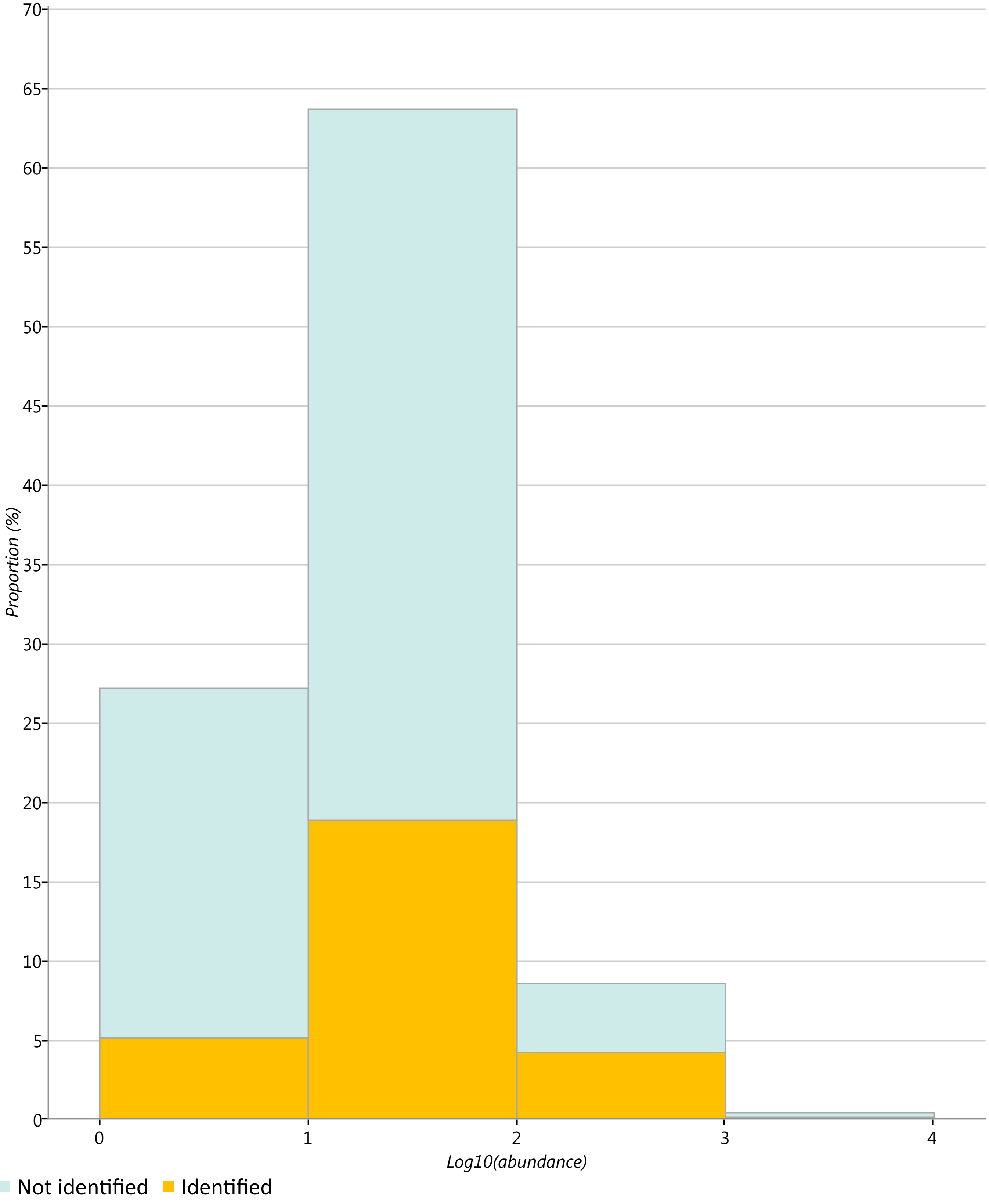

Precursor m/z

This chart shows the distribution of m/z values for all peptide ions in the experiment. Identified peptide ions are shown in yellow.

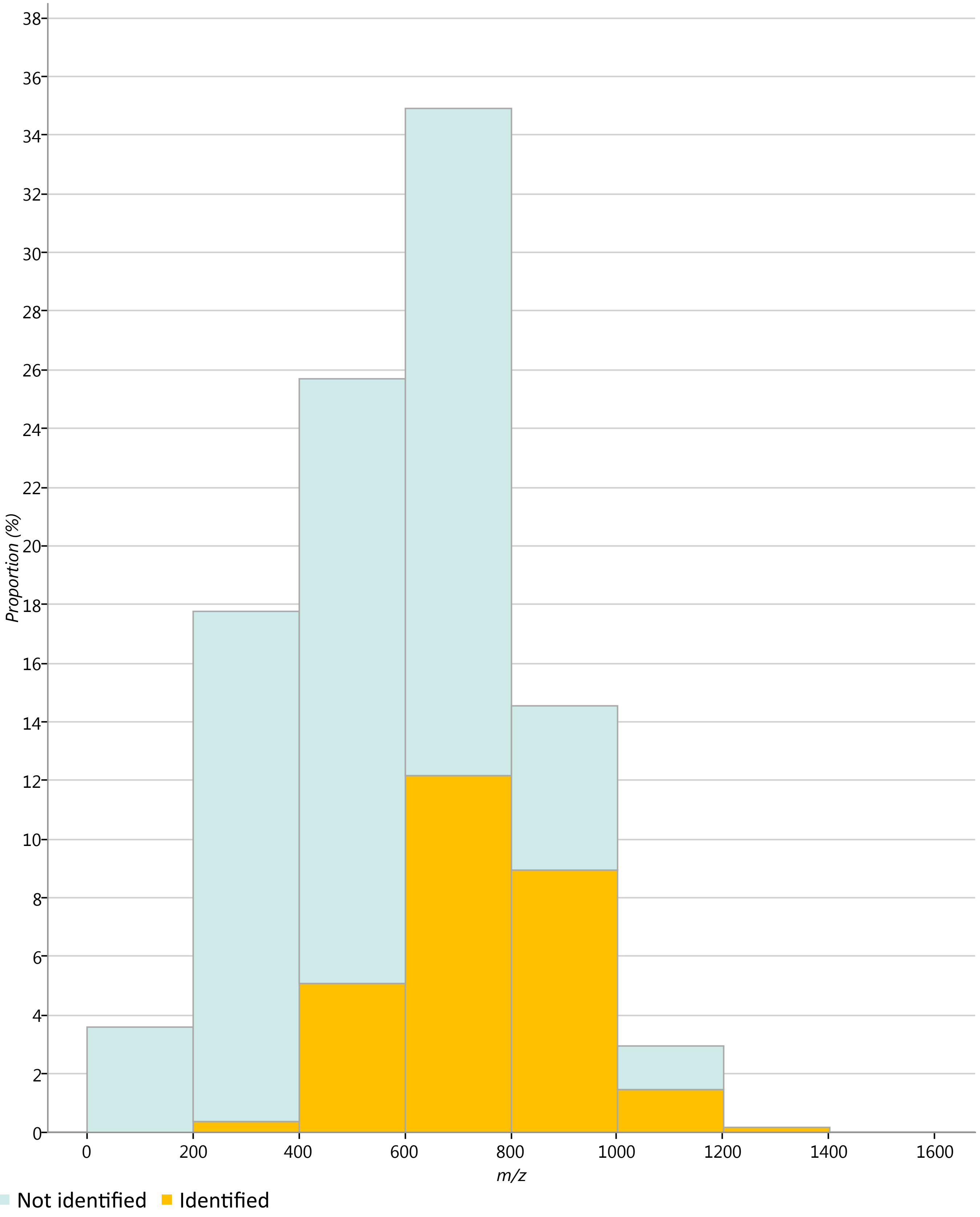

Precursor retention time

This chart shows the proportion of precursor peptide ions eluting into each quartile of the LC run. Identified peptide ions are shown in yellow.

Potential problems:

- A majority of peptide ions eluting at the start or end of the run may indicate problems with the chromatographic separation or that the setup is not ideal for a successful separation.

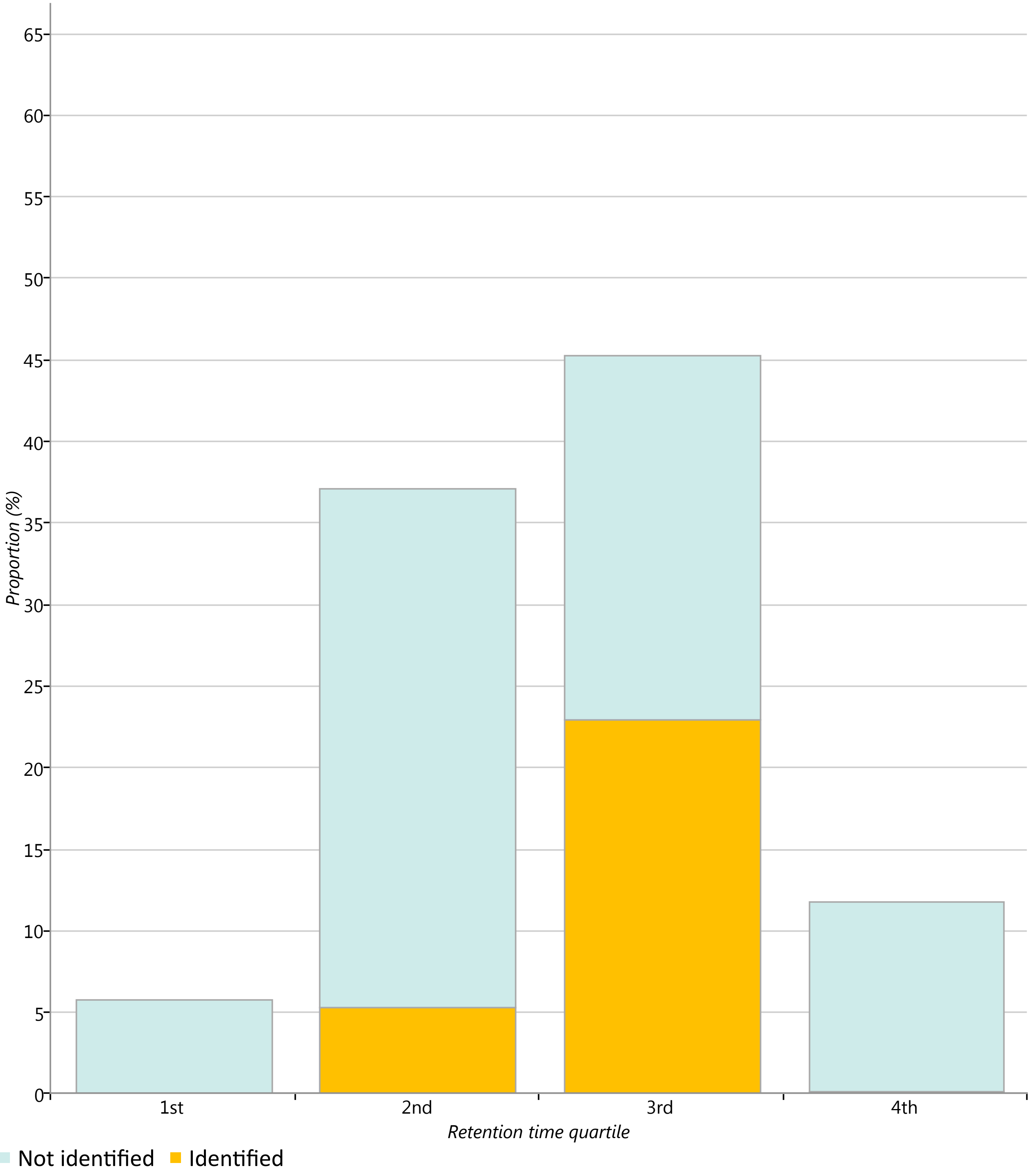

Mass errors

This chart shows the mass error calculated for identified peptide ions. The proportion of peptide ions with a given binned error value is plotted. For a more detailed plot, see the *Mass accuracy* metric.

Potential problems:

- A larger than expected spread could indicate that search parameters are too lenient.
- A bias of the peak from zero ppm could indicate instrument bias.

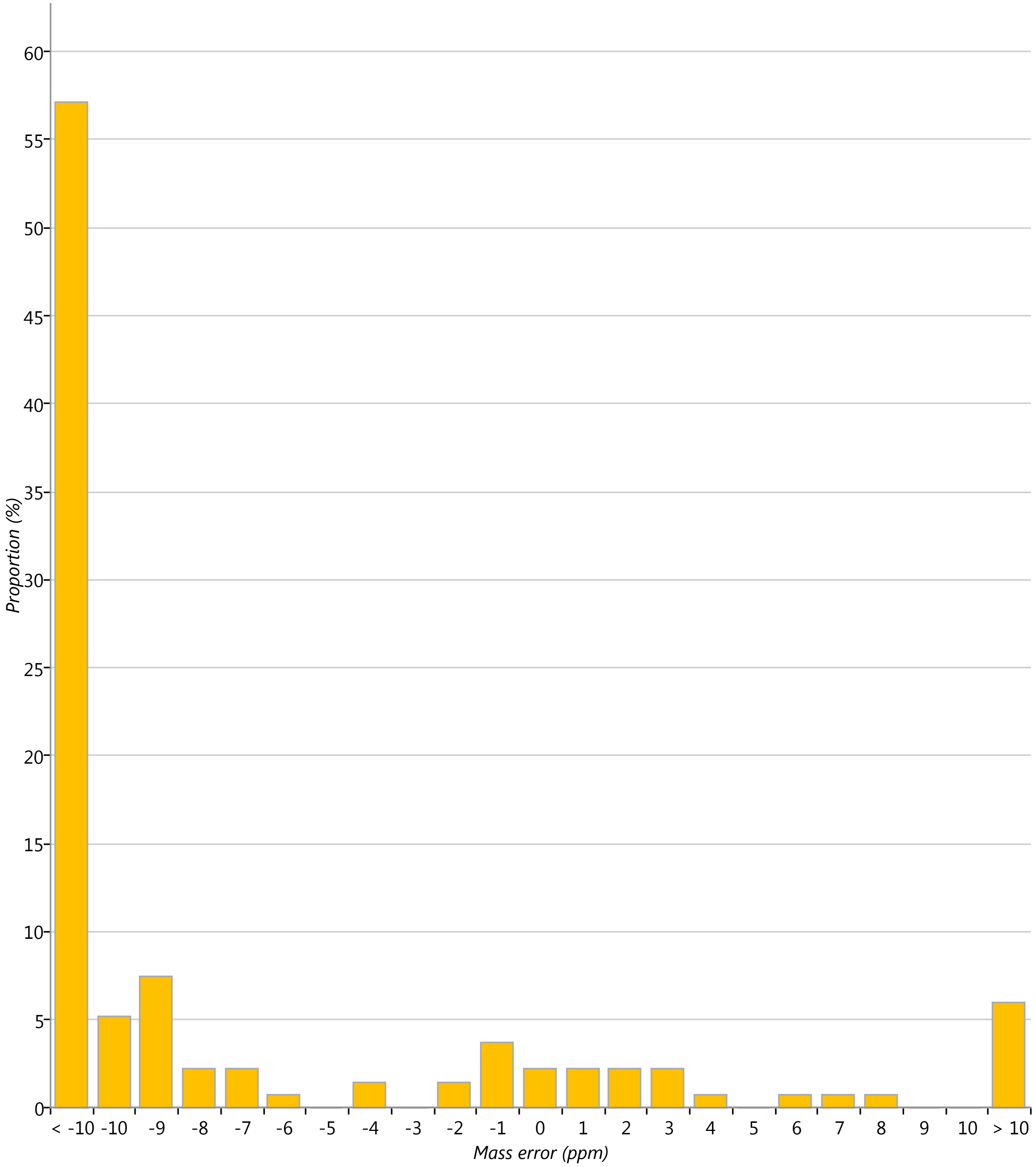

Precursor charges

This chart shows the percentage of peptide ions with various charge states.

Potential problems:

- A skew towards low charge peptide ions may indicate poor ionisation, suggesting that you may need to raise the collision voltage.
- A skew towards high charge peptide ions may indicate excessive ionisation, suggesting that you may need to lower the collision voltage.

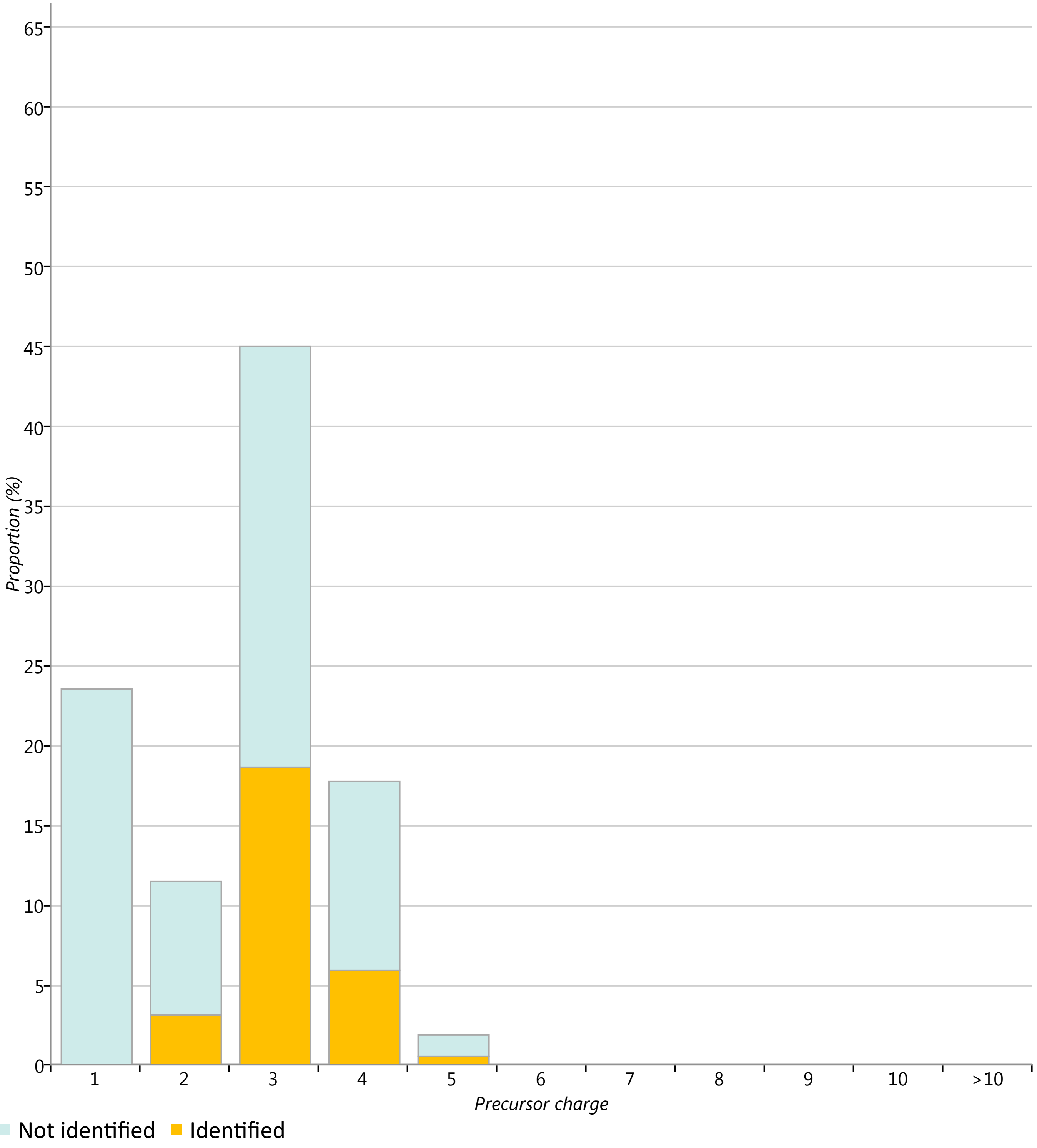

Missed cleavages (assuming trypsin)

This chart shows the distribution of missed cleavages per identified peptide ion across the whole experiment, assuming the proteins were treated with trypsin and using each peptide ion's highest scoring identification. For an alternative visualisation showing the data subdivided by experimental condition, see the *Missed cleavages* metric.

Potential problems:

- A high proportion of missed cleavages may indicate problems in trypsinization.
- An absence of missed cleavages may indicate that the database search parameters were too stringent.

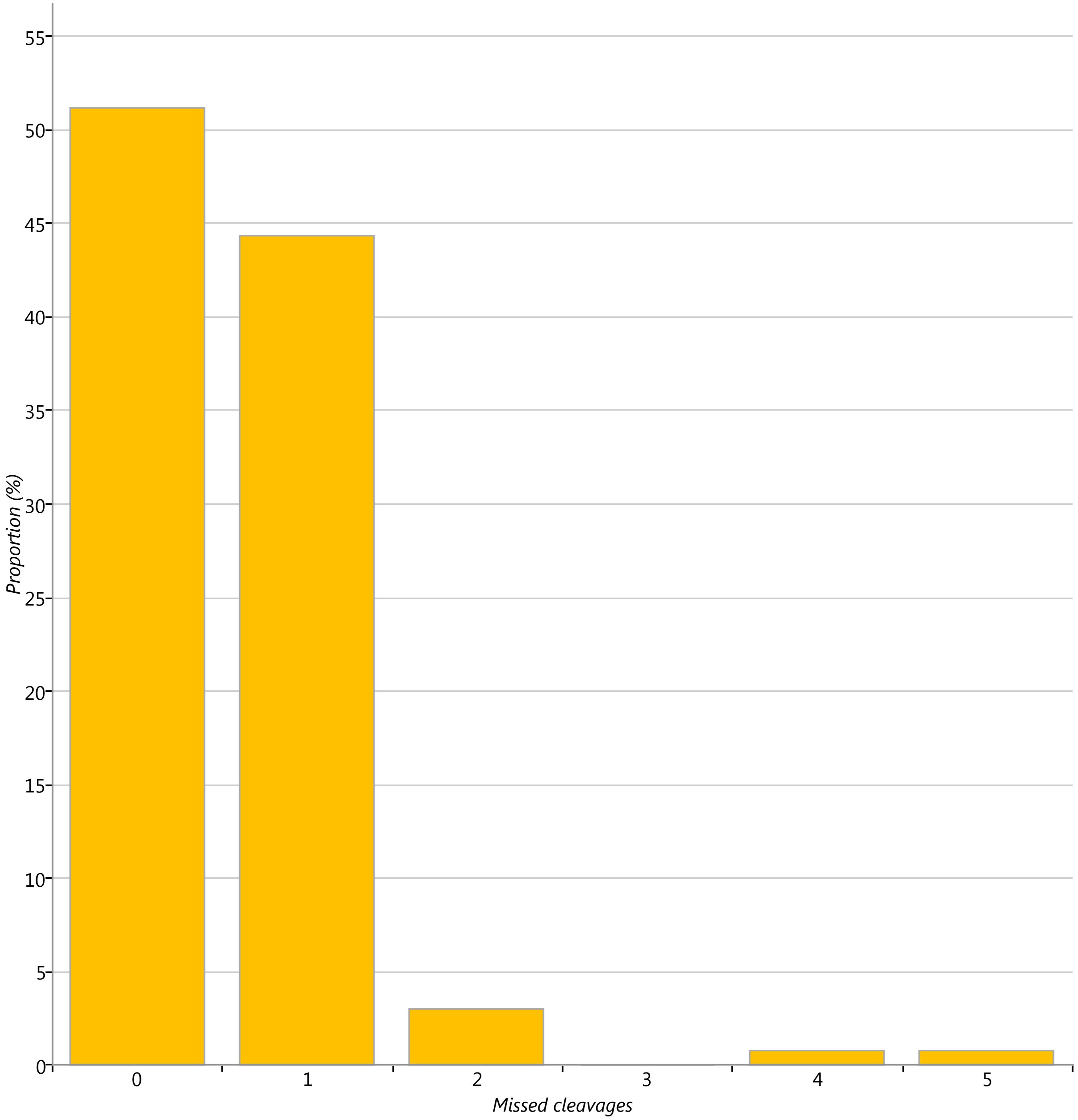

Peptides per protein (overview)

This chart shows the proportional distribution of the number of peptides identified per protein, combined across all runs in the experiment. For an alternative visualisation dividing these data by experimental condition, see the *Peptides per protein (by condition)* metric.

Potential problems:

- An unexpected bias to high or low numbers of peptides may indicate problems with sample preparation or peptide search settings.

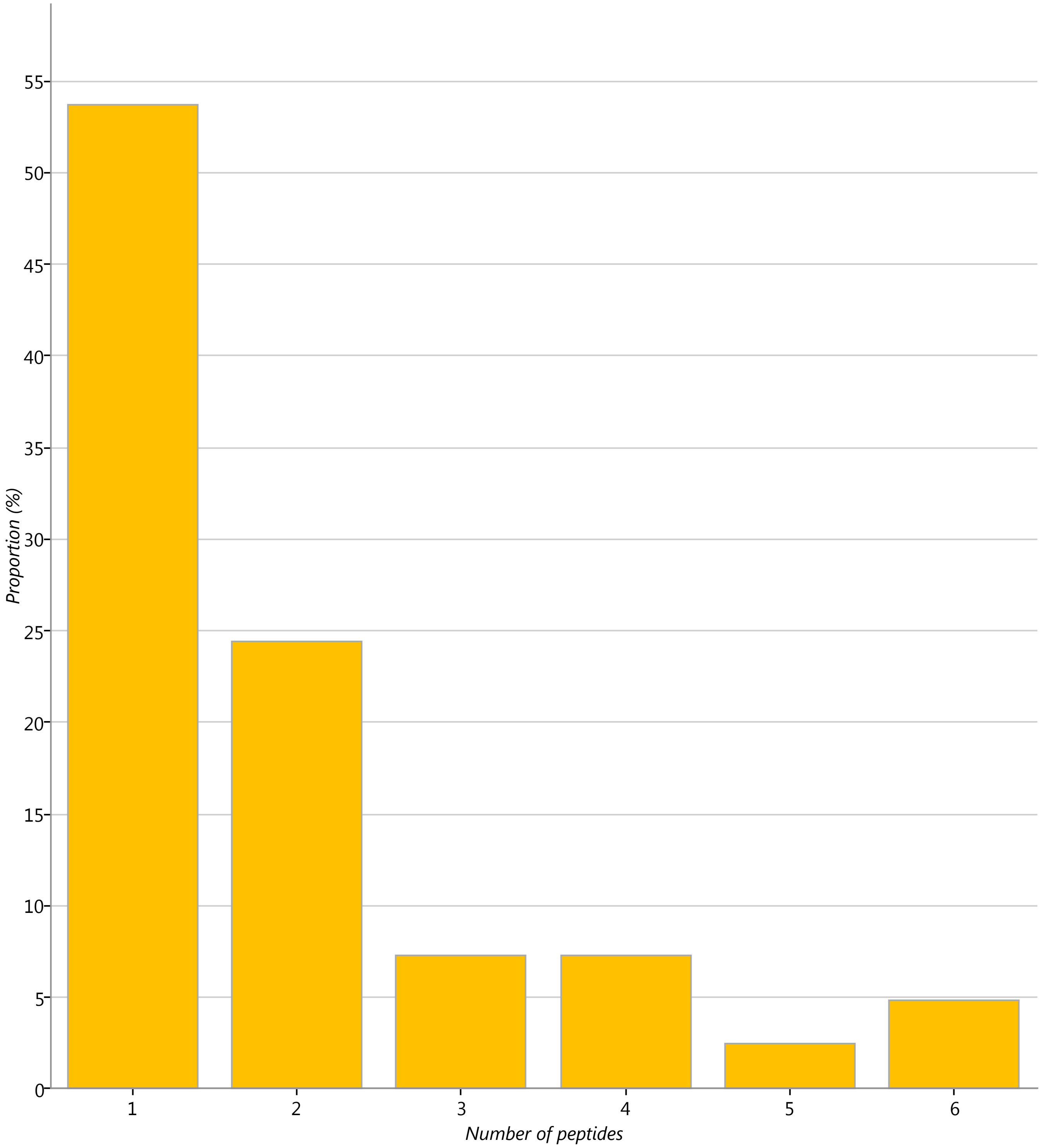

Modifications

The chart below shows the mean number of modifications per peptide ion for runs in the selected experiment design, based on each peptide ion's highest scoring identification. Each run is coloured according to its experimental condition.

Potential problems:

- Unexpectedly low numbers of modifications may indicate that one or more modifications were mistakenly not selected when searching for identifications.
- Variable numbers of modifications across the runs, especially within a given condition, may indicate problems with sample preparation.

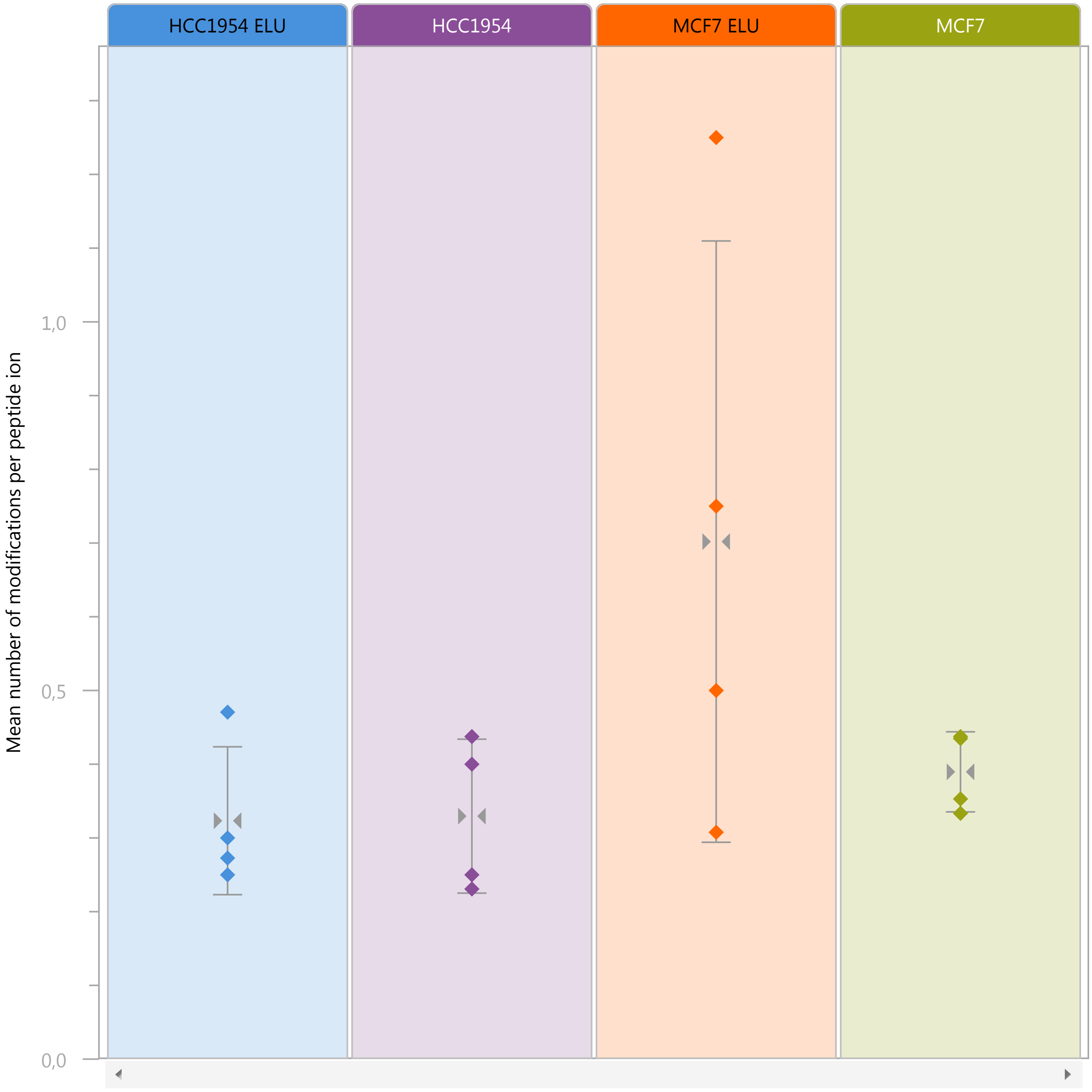

Included modifications Acetyl N-TERM Carbamidomethyl C Carbamyl N-TERM Deamidation N Dean

Missed cleavages

The chart below shows the mean number of missed cleavages per peptide ion for runs within the selected experiment design, based on each peptide ion's highest scoring identification. The chart assumes the use of trypsin for digestion. Each run is coloured according to its experimental condition.

Potential problems:

- A high number of missed cleavages may indicate problems in trypsinization.
- An absence of missed cleavages may indicate that database search parameters were too stringent.

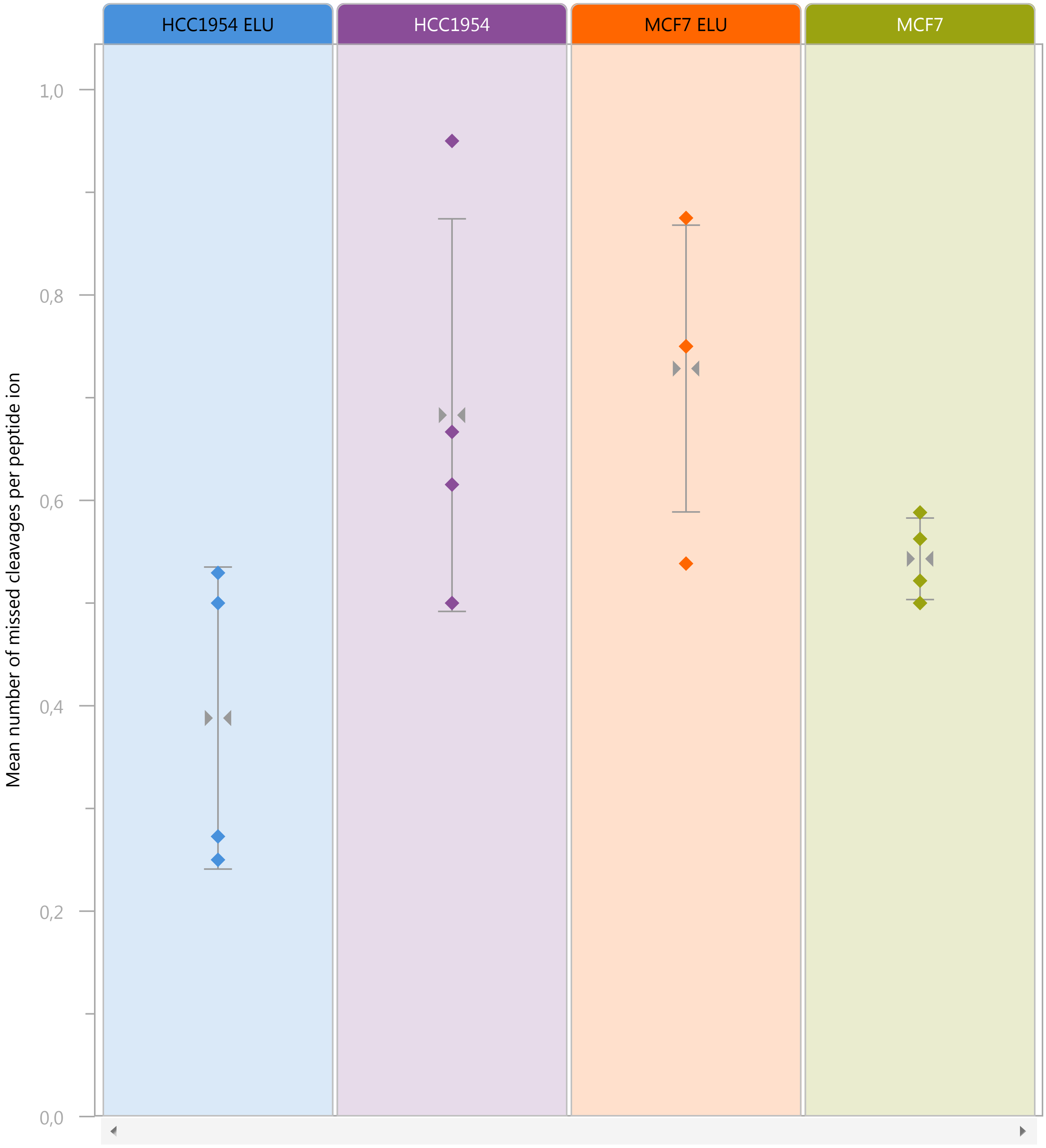

Abundance dynamic range

The charts below show abundance histograms for peptide ions, calculated using the current experiment design. Each chart shows the mean abundance for peptide ions within an experimental condition's runs. Data are binned by Log10 of their normalised abundance and shown as a proportion of the total. Identified peptide ions are shown in yellow.

Potential problems:

- A heavily right-skewed histogram of *identified* peptide ions indicates you may be missing identifications with low abundance.
- A heavily right skewed total (summed identified and unidentified) histogram may indicate saturation at high abundance, which will affect the accuracy of quantitation.

HCC1954 ELU

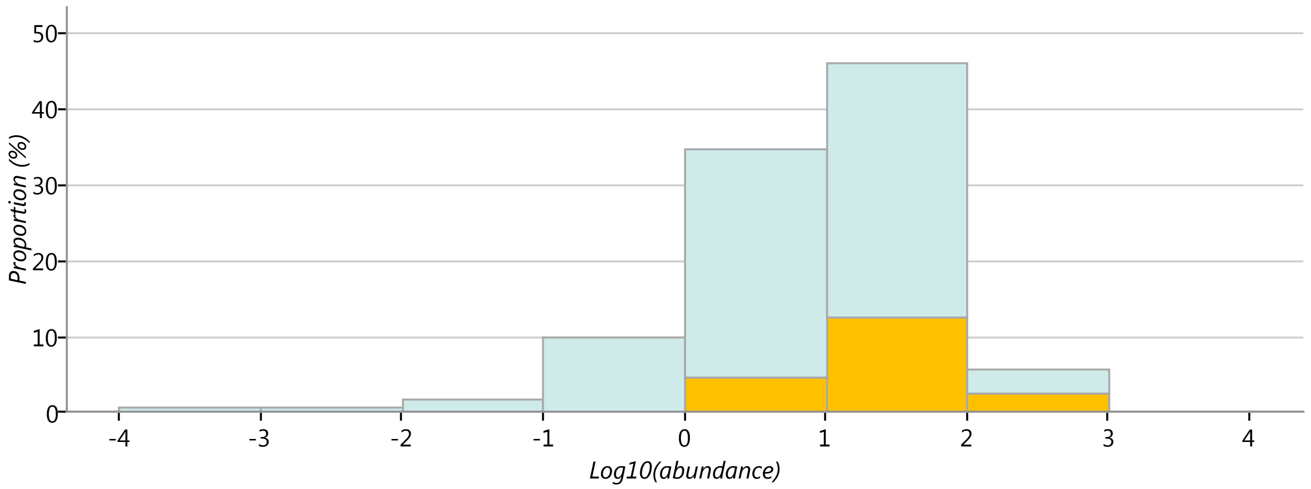

HCC1954

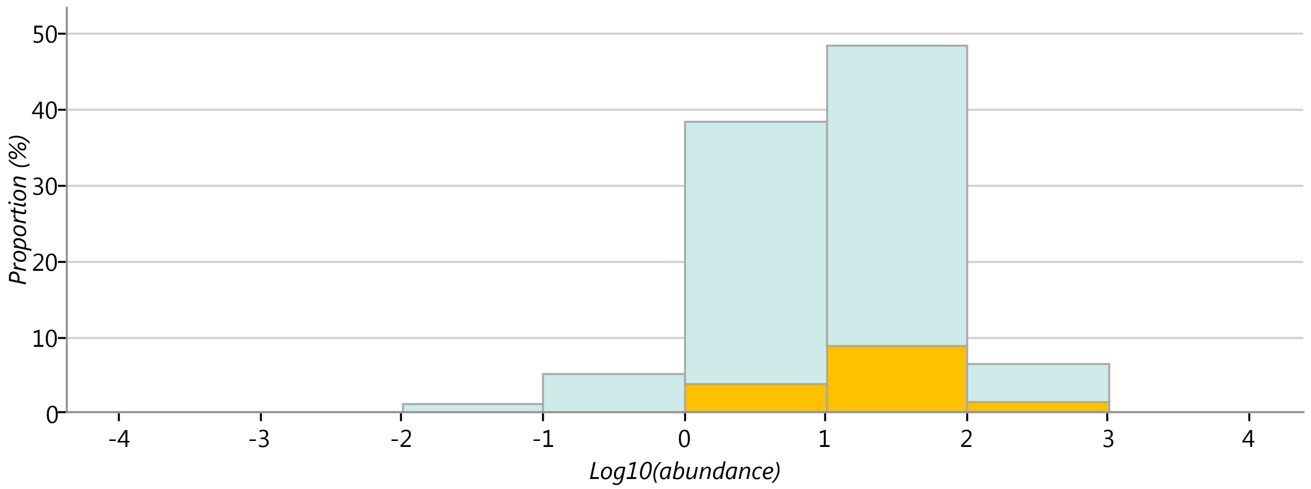

Not identified ■ Identified

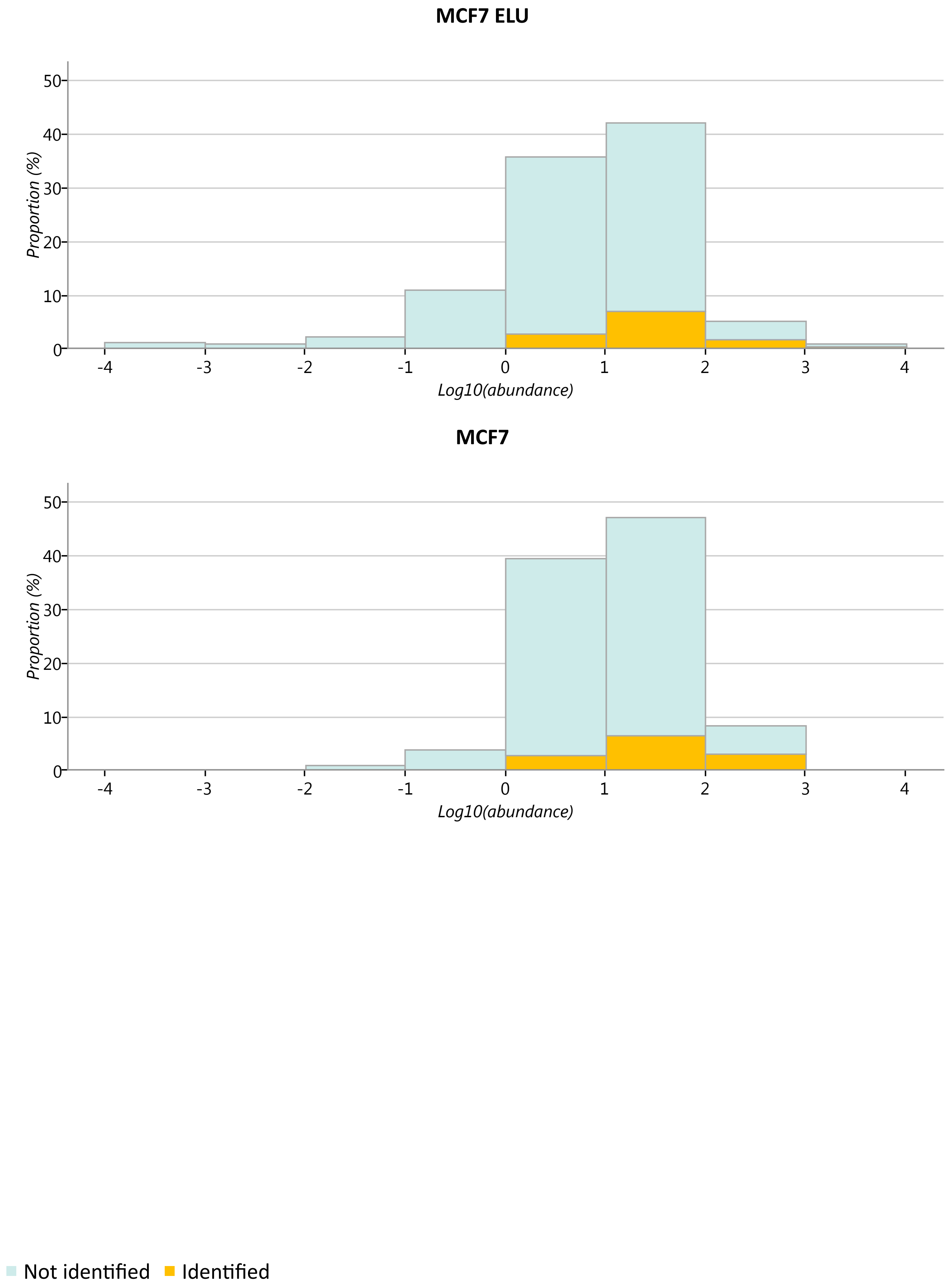
MCF7 ELU
Proportion (%)
50
40
30
20
10
0
-4
-3
-2
-1
0
1
2
3
4
Log10(abundance)
MCF7
Proportion (%)
50
40
30
20
10
0
-4
-3
-2
-1
0
1
2
3
4
Log10(abundance)
Not identified
Identified

Mass accuracy

The chart below shows all identified peptide ions in your experiment, plotting peptide ion m/z against the ppm error in its highest scoring identification. Blue points indicate peptide ions which have a mass error within 2 standard deviations of the mean, yellow points indicate peptide ions with a mass error more than 2 standard deviations from the mean.

Potential problems:

- A dependence of ppm mass error on m/z may indicate poor calibration.
- If the standard deviation of the data is large in ppm terms, this may indicate that your search parameters are too lenient.

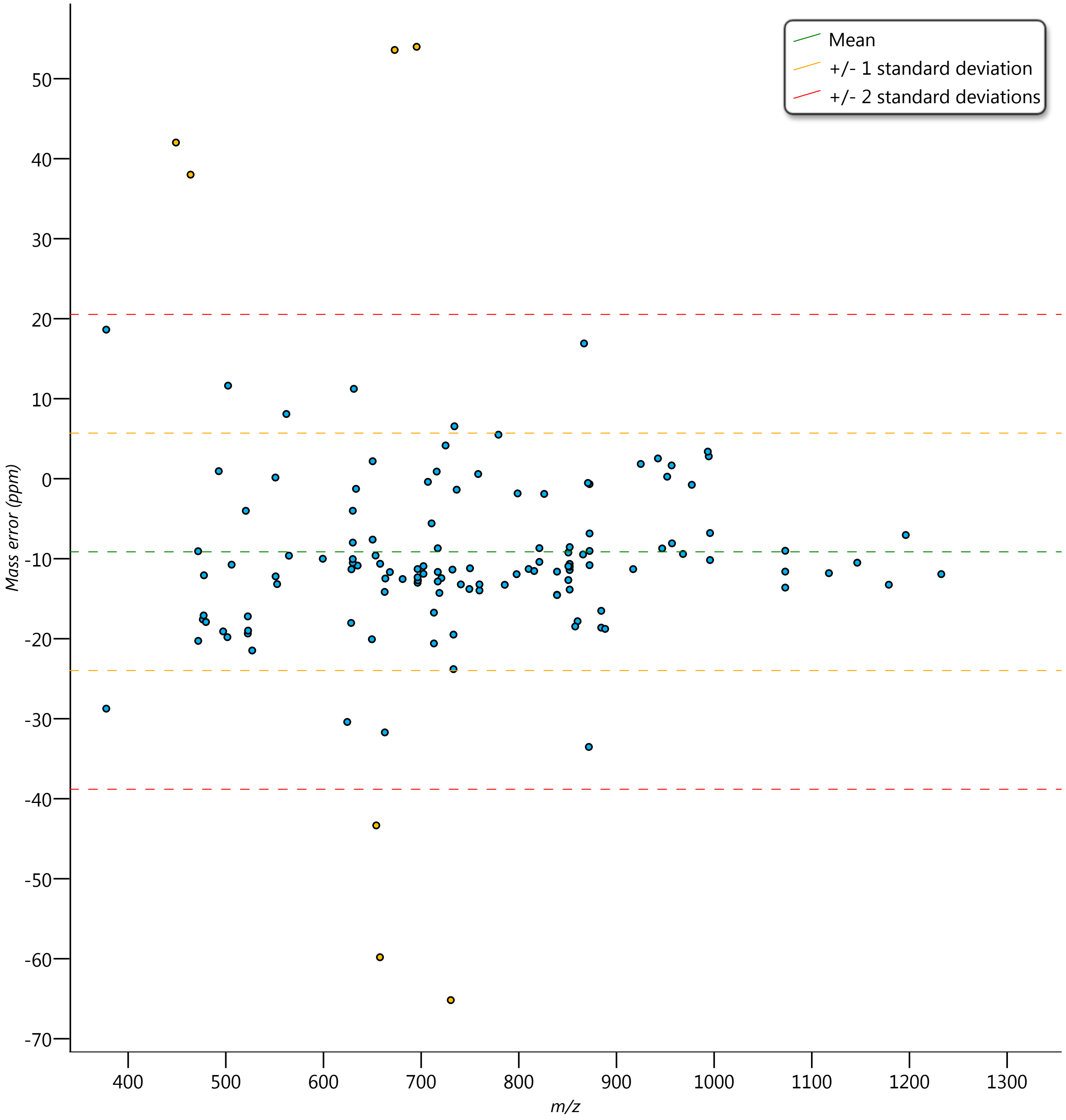

Scan rates

The charts below show the MS1 scan rate over the duration of your runs, using the current experiment design. Each chart shows the scan rate variation within an experimental condition's runs. The named run is highlighted in black. The variability between runs is shown as a grey area. The y-axis shows scans per second in a 30-second window centred on each x-axis time point.

Potential problems:

- A highly varying scan rate means quantitation accuracy may be negatively affected.

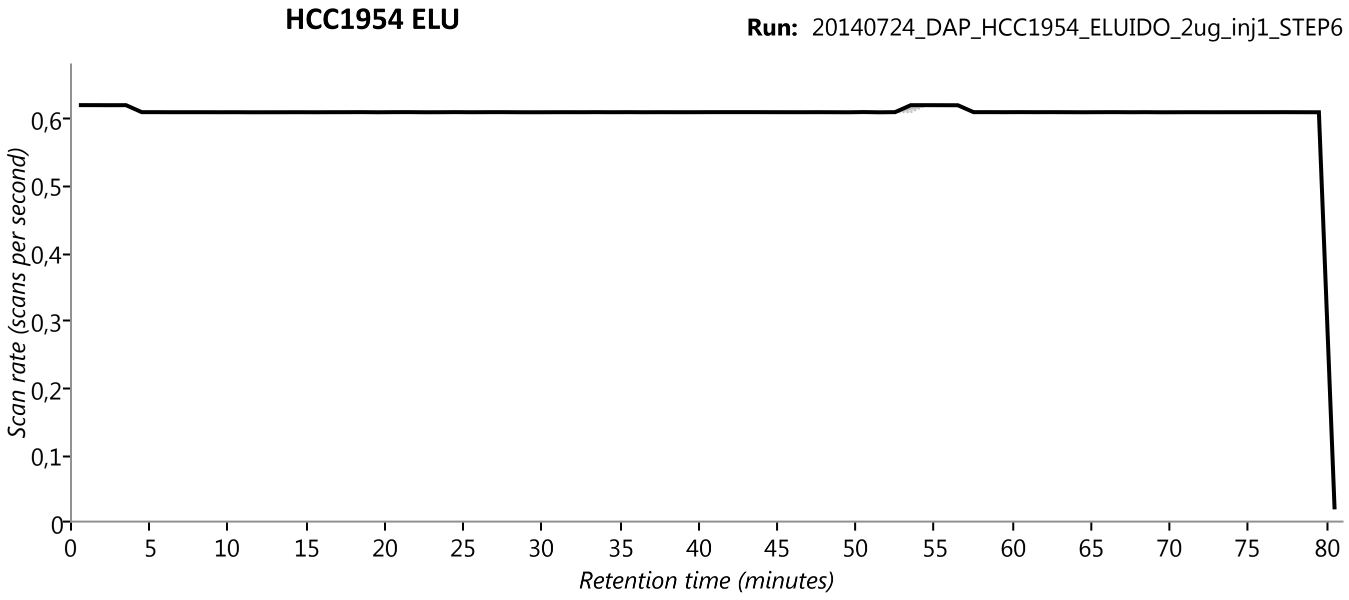

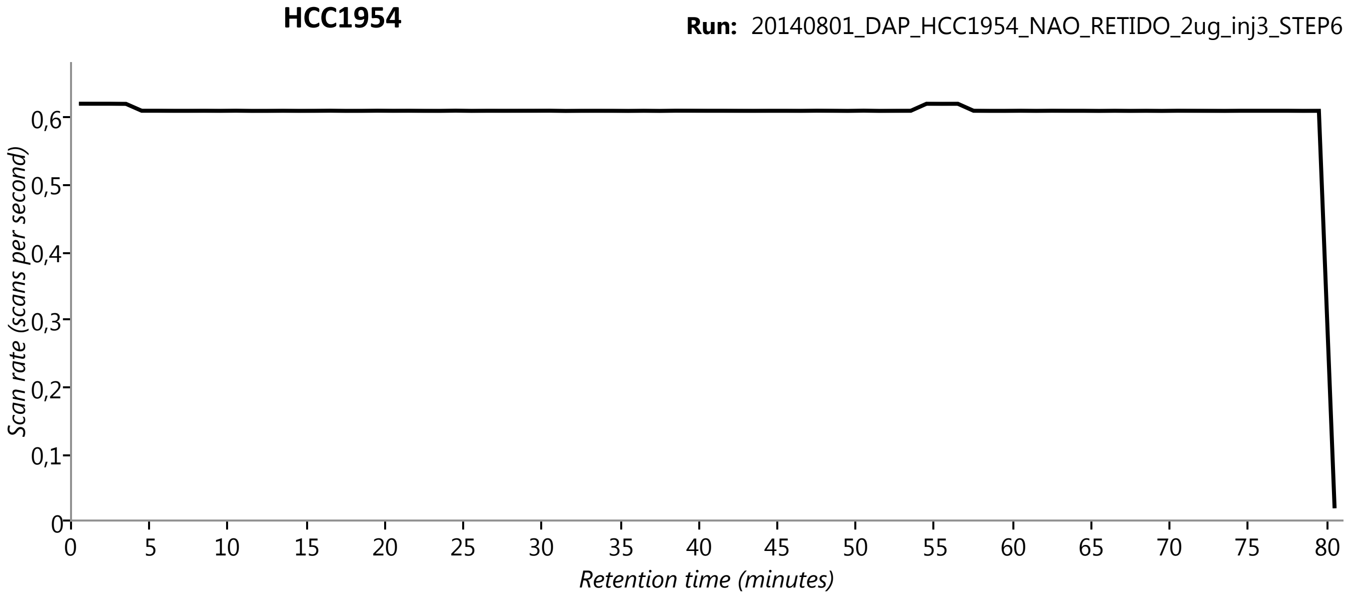

MCF7 ELU

Run: 20140724_DAP_MCF7_ELUIDO_2ug_inj4_STEP6

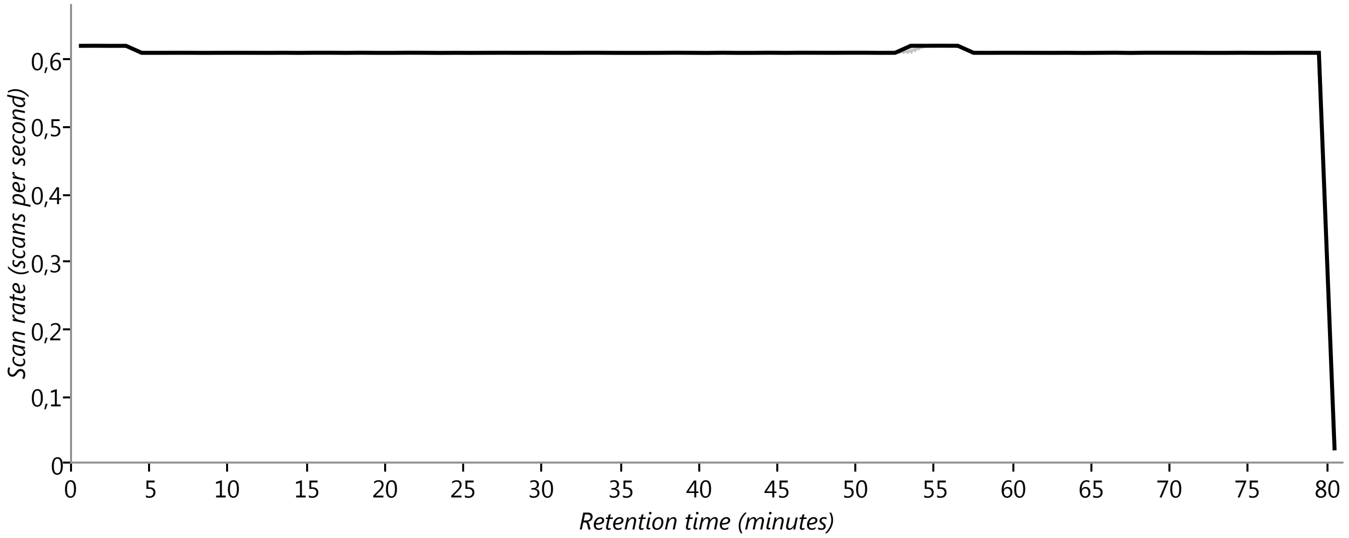

MCF7

Run: 20140801_DAP_MCF7_NAO_RETIDO_2ug_inj4_STEP6

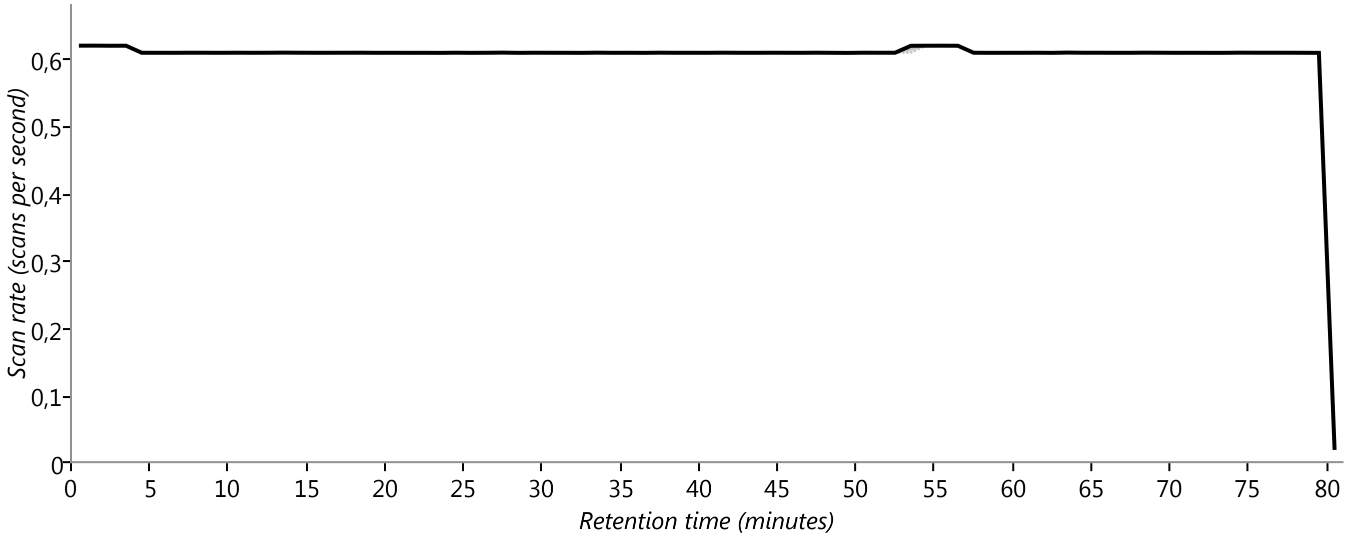

Proteins

The chart below shows the number of proteins identified in each of the runs within the selected experiment design. Each run is coloured according to its experimental condition.

Potential problems:

- Any exceptionally high or low values may indicate problems with sample preparation, chromatography, or instrument settings.

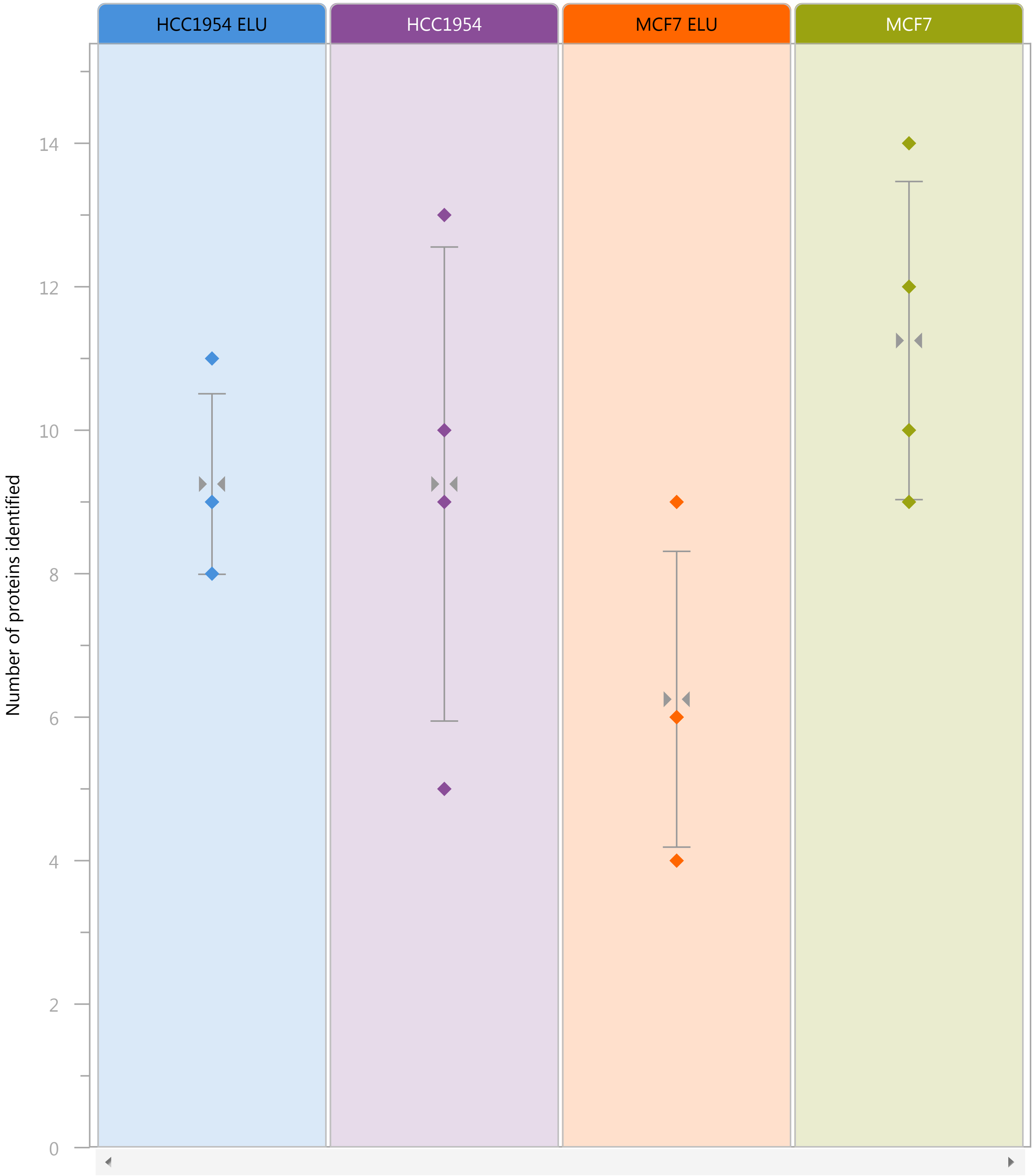

Peptides

The chart below shows the number of peptides identified in each of the experiment's runs. Each run is coloured according to its experimental condition.

Potential problems:

- Any exceptionally high or low values may indicate problems with sample preparation, chromatography, or instrument settings.

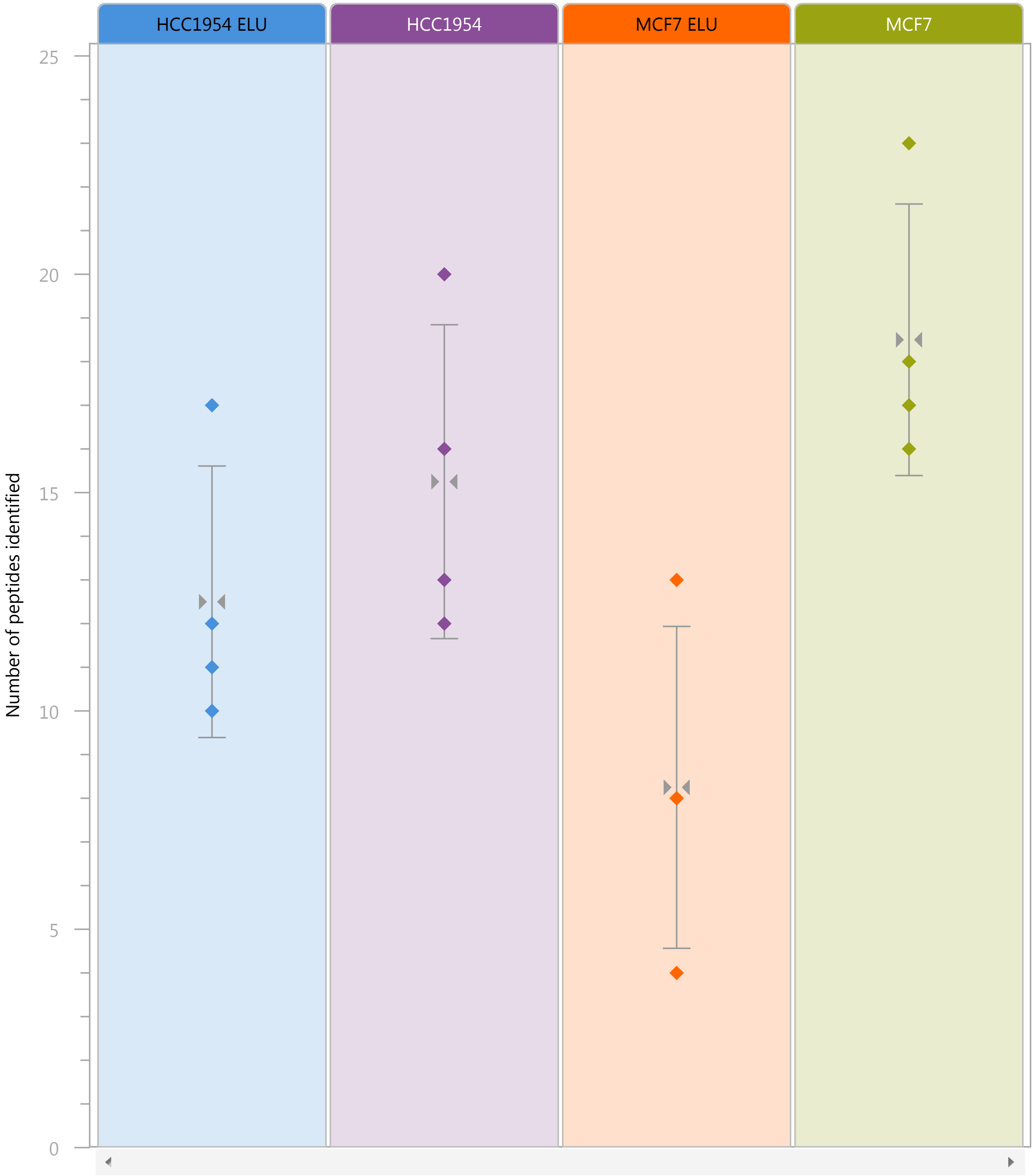

Peptides per protein (by condition)

The chart below shows the average number of peptides per protein in each run within the selected experiment design. For a given run, the value shown is calculated using only identifications from that run.

Potential problems:

- Any exceptionally high or low values may indicate problems with sample preparation or peptide search settings.

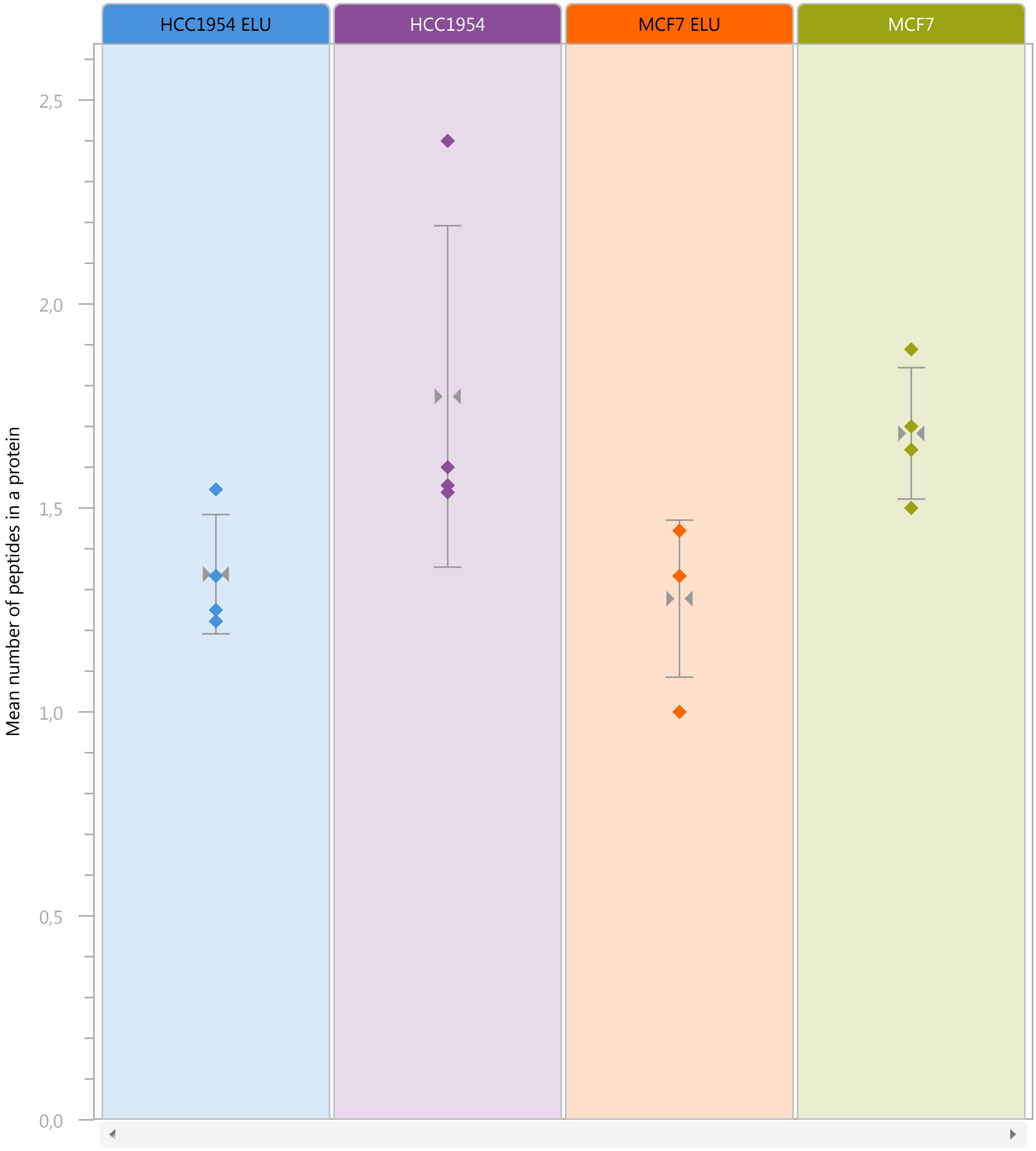

Percentage of peptide ions identified

The chart below shows the number of peptide ions identified in each of the runs within the selected experiment design, expressed as a percentage of the total number of peptide ions. Each run is coloured according to its experimental condition.

Potential problems:

- Any exceptionally high or low values may indicate problems with sample preparation, chromatography, or instrument settings.
- Very low values across all runs may indicate a problem with the database that was searched for identifications, or with other search parameters.

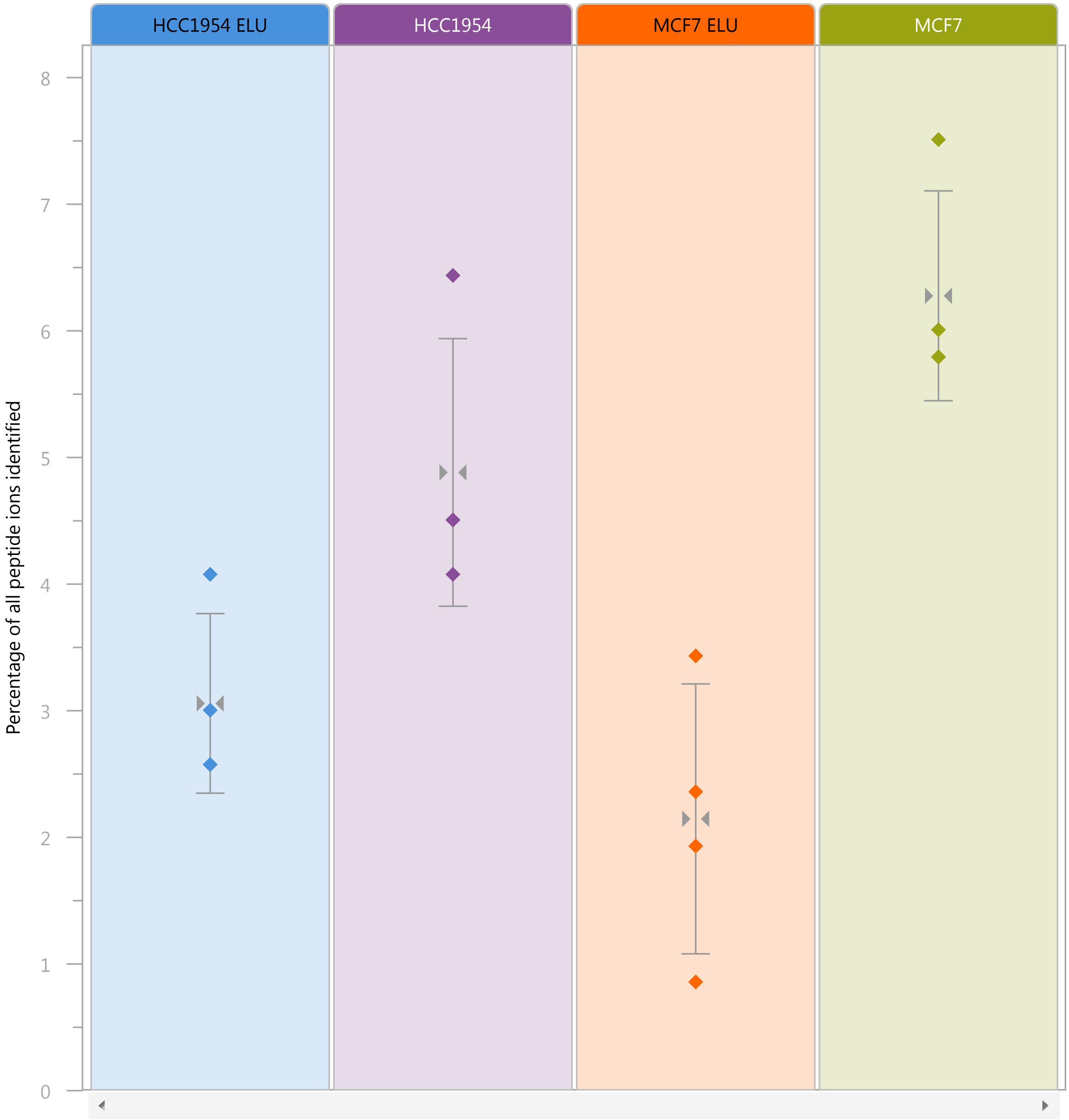

Protein overlap

The Venn diagram shows the number of proteins identified uniquely in the selected conditions and the number that are shared between conditions. Use the list on the right to select two or three conditions to explore.

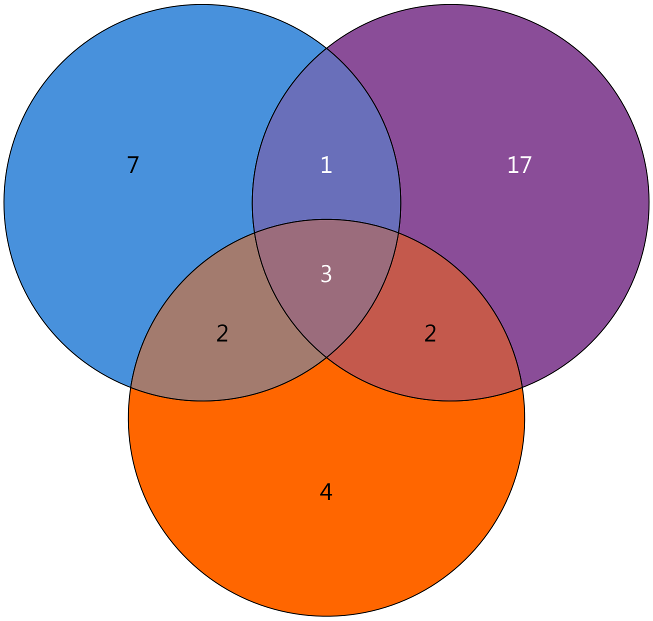

Select experimental conditions

- HCC1954 ELU
- HCC1954
- MCF7 ELU

HCC1954 X MCF7 STEP7

QC Metrics reported by Progenesis QI for proteomics v2.0.5556.29015
Date: 10-Jan-2020 at 09:36
Experiment design: HCC1954 XMCF7 STEP7

This QC report contains **all metrics** for the experiment named above.

Reported metrics

0 of the 19 metrics reported have been manually flagged for attention.

- Identifications overview
- LC peak width
- Peptide ion dynamic range
- Precursor m/z
- Precursor retention time
- Mass errors
- Precursor charges
- Missed cleavages (assuming trypsin)
- Peptides per protein (overview)
- Modifications
- Missed cleavages
- Abundance dynamic range
- Mass accuracy
- Scan rates
- Proteins
- Peptides
- Peptides per protein (by condition)
- Percentage of peptide ions identified
- Protein overlap

Identifications overview

The chart below provides the number of identifications per run by category. Data are plotted for all runs in the current experiment design. The box represents the interquartile range and the whiskers the maximum and minimum.

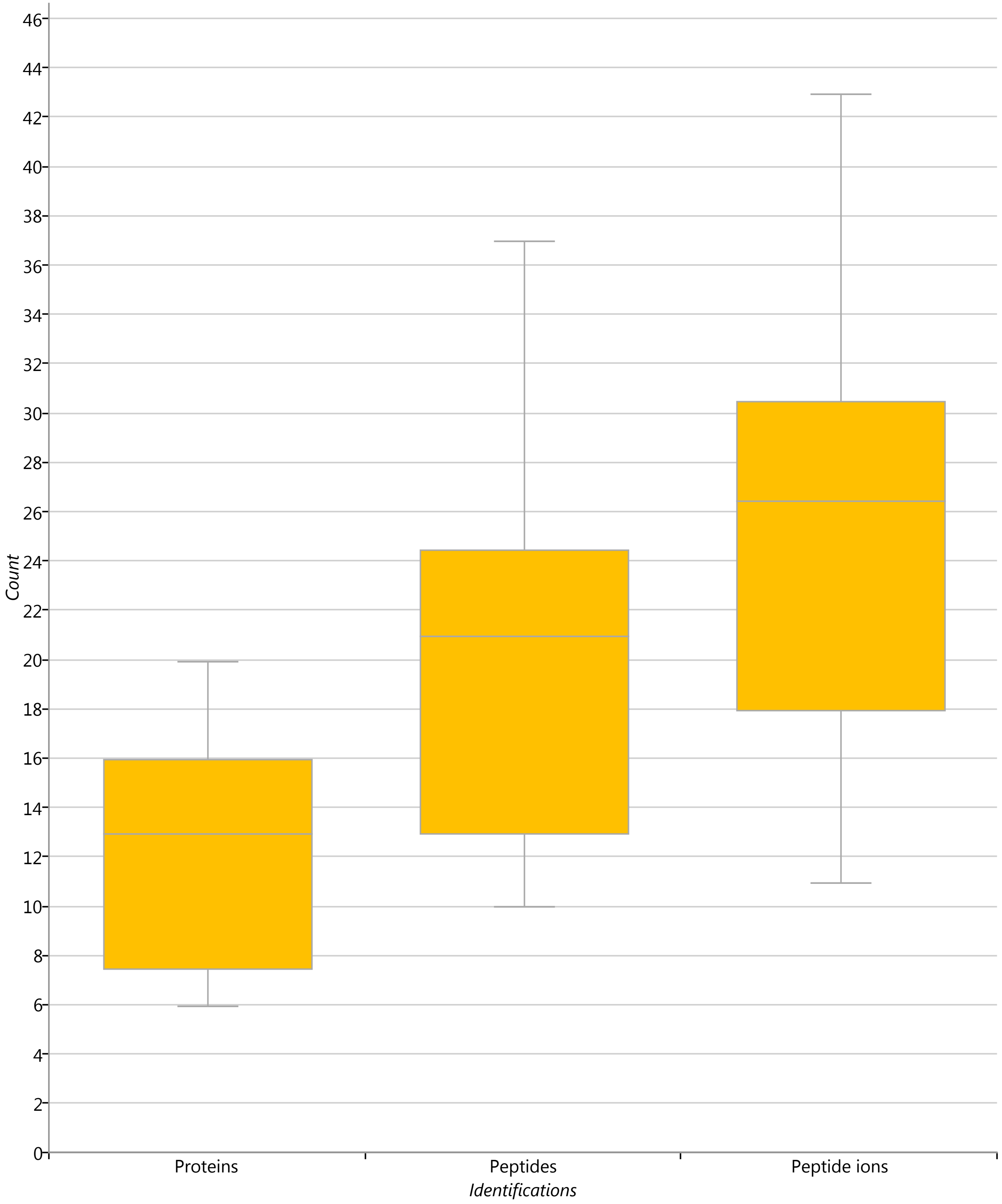

LC peak width

This chart shows the proportional distribution of peak widths in retention time, for all the detected peptide ions in the experiment. Widths are binned into categories for simple comparison. Identified peptide ions are shown in yellow.

Potential problems:

- A peak width that is different from that expected for your setup may indicate a problem with the chromatographic separation.

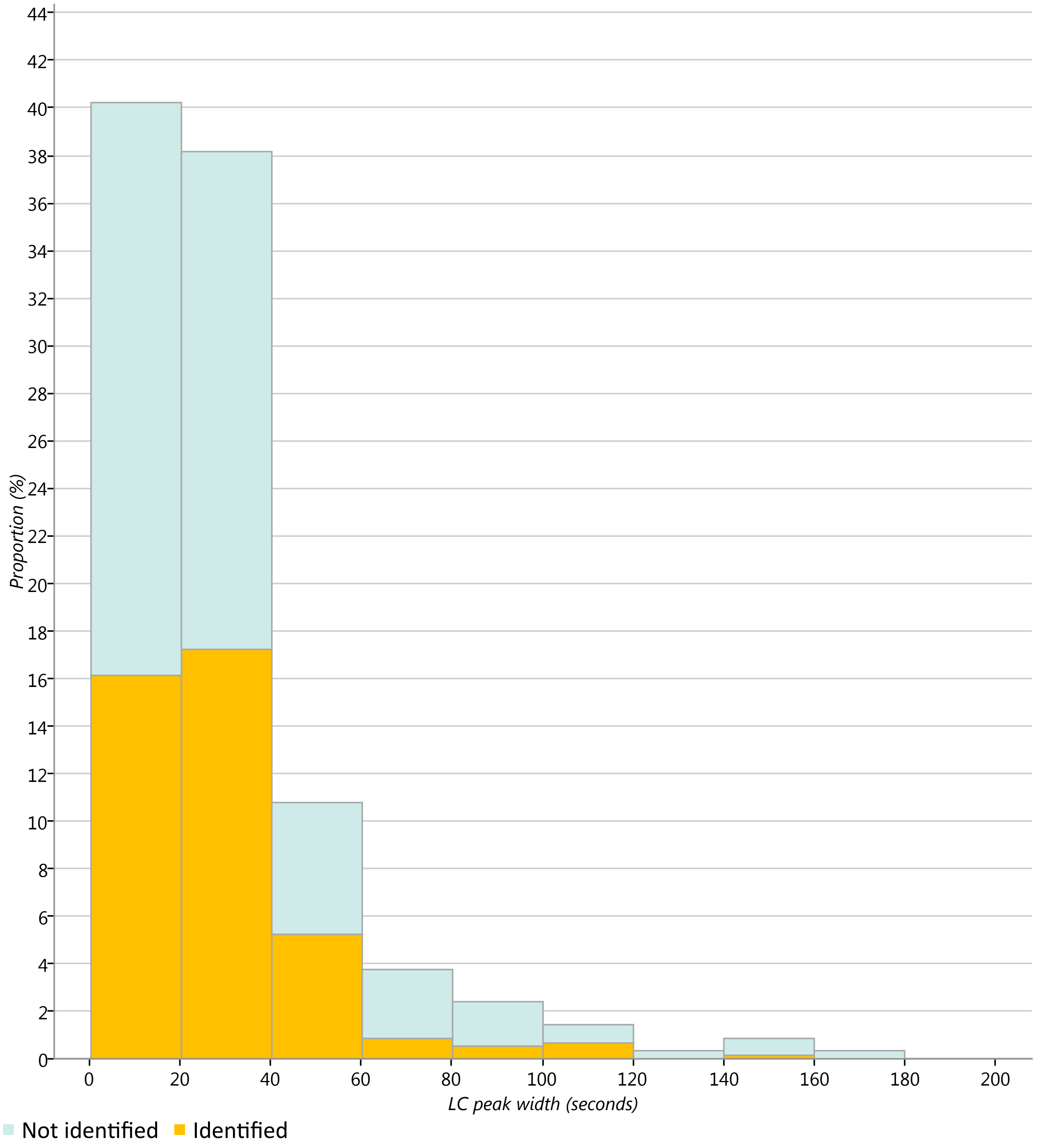

Peptide ion dynamic range

This chart shows log abundance for peptide ions, calculated using the current experiment design. Each peptide ion is assigned its abundance based on the highest mean value for any condition's runs. Data are binned and shown as a proportion. Identified peptide ions are shown in yellow.

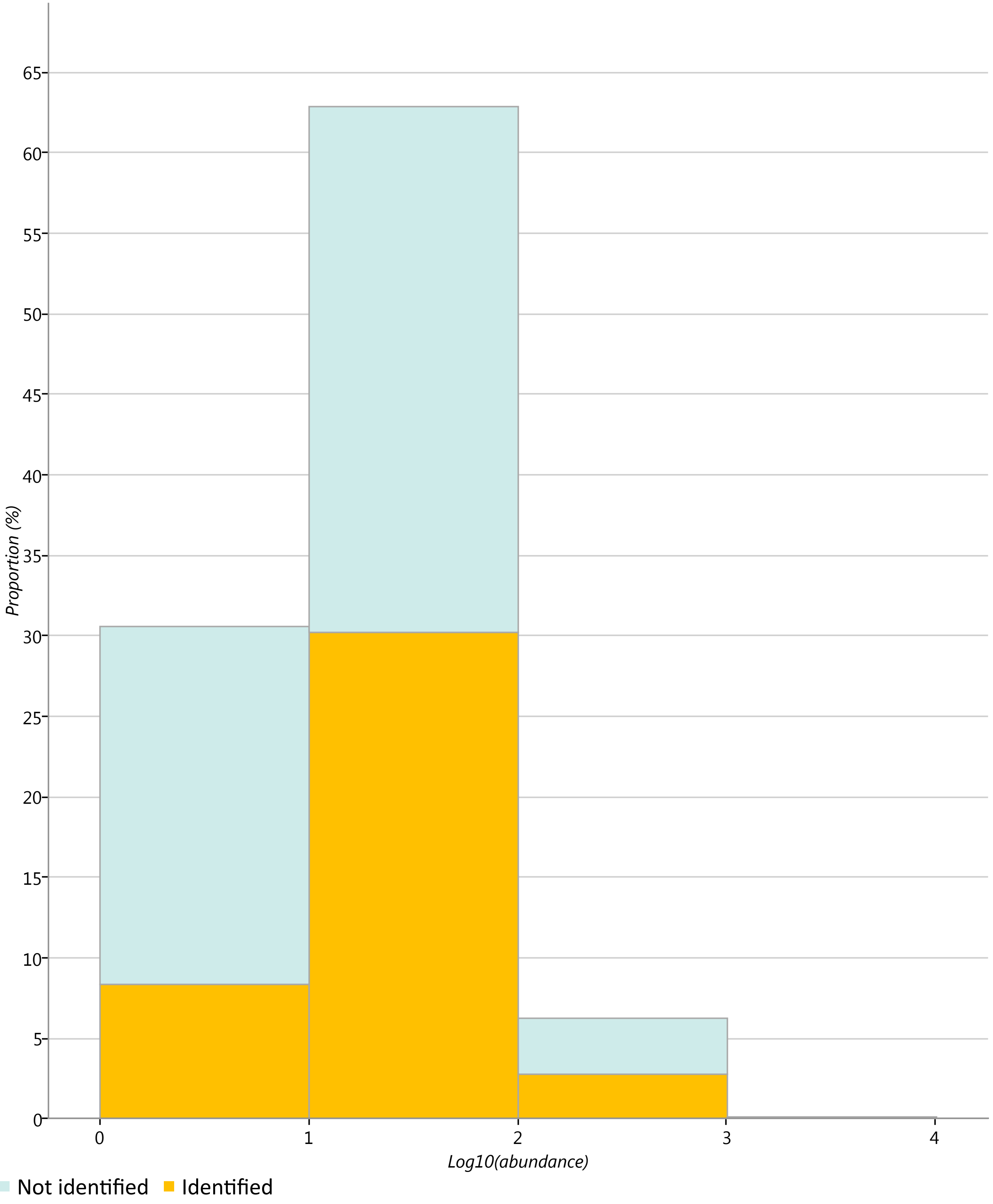

Precursor m/z

This chart shows the distribution of m/z values for all peptide ions in the experiment. Identified peptide ions are shown in yellow.

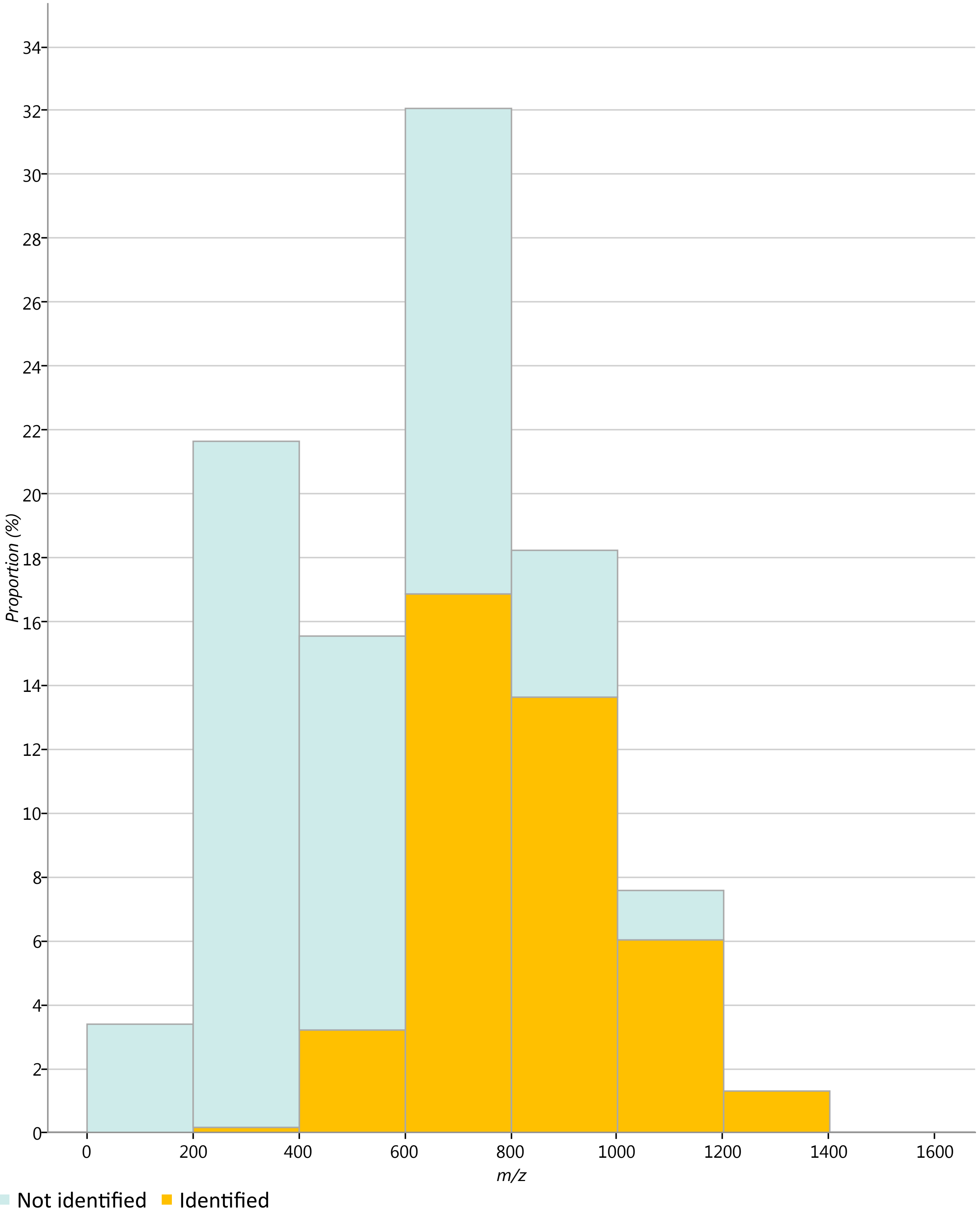

Precursor retention time

This chart shows the proportion of precursor peptide ions eluting into each quartile of the LC run. Identified peptide ions are shown in yellow.

Potential problems:

- A majority of peptide ions eluting at the start or end of the run may indicate problems with the chromatographic separation or that the setup is not ideal for a successful separation.

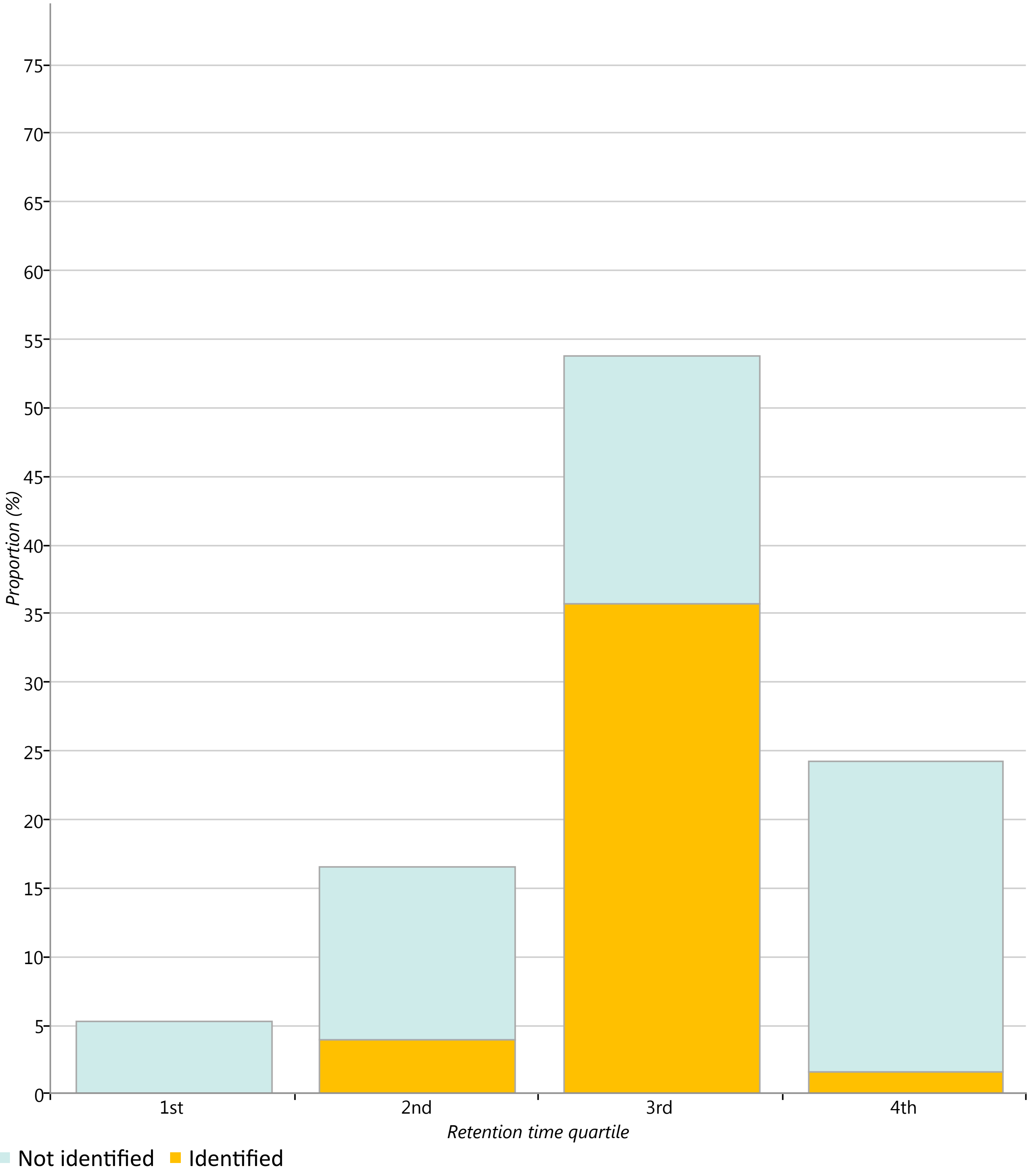

Mass errors

This chart shows the mass error calculated for identified peptide ions. The proportion of peptide ions with a given binned error value is plotted. For a more detailed plot, see the *Mass accuracy* metric.

Potential problems:

- A larger than expected spread could indicate that search parameters are too lenient.
- A bias of the peak from zero ppm could indicate instrument bias.

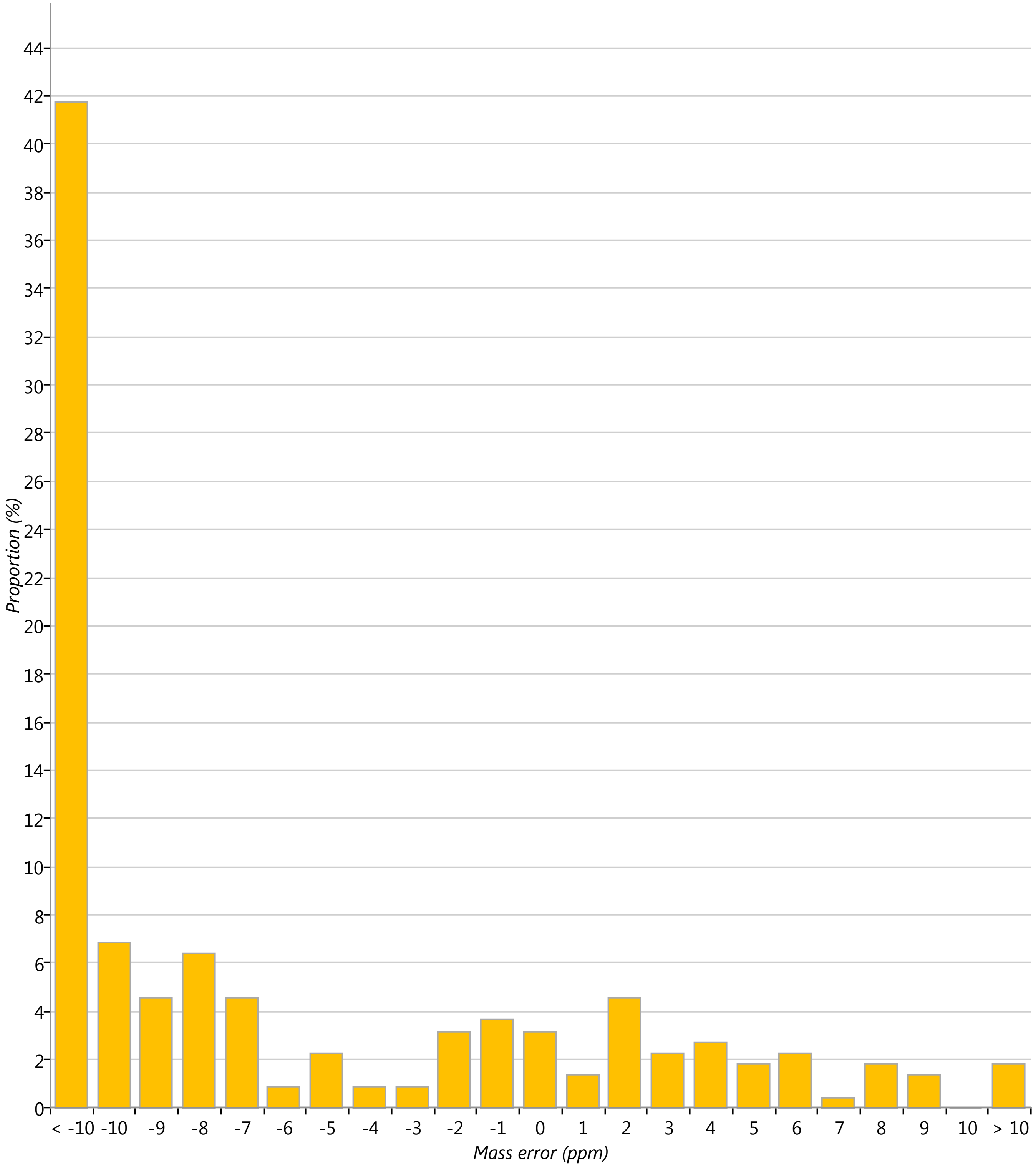

Precursor charges

This chart shows the percentage of peptide ions with various charge states.

Potential problems:

- A skew towards low charge peptide ions may indicate poor ionisation, suggesting that you may need to raise the collision voltage.
- A skew towards high charge peptide ions may indicate excessive ionisation, suggesting that you may need to lower the collision voltage.

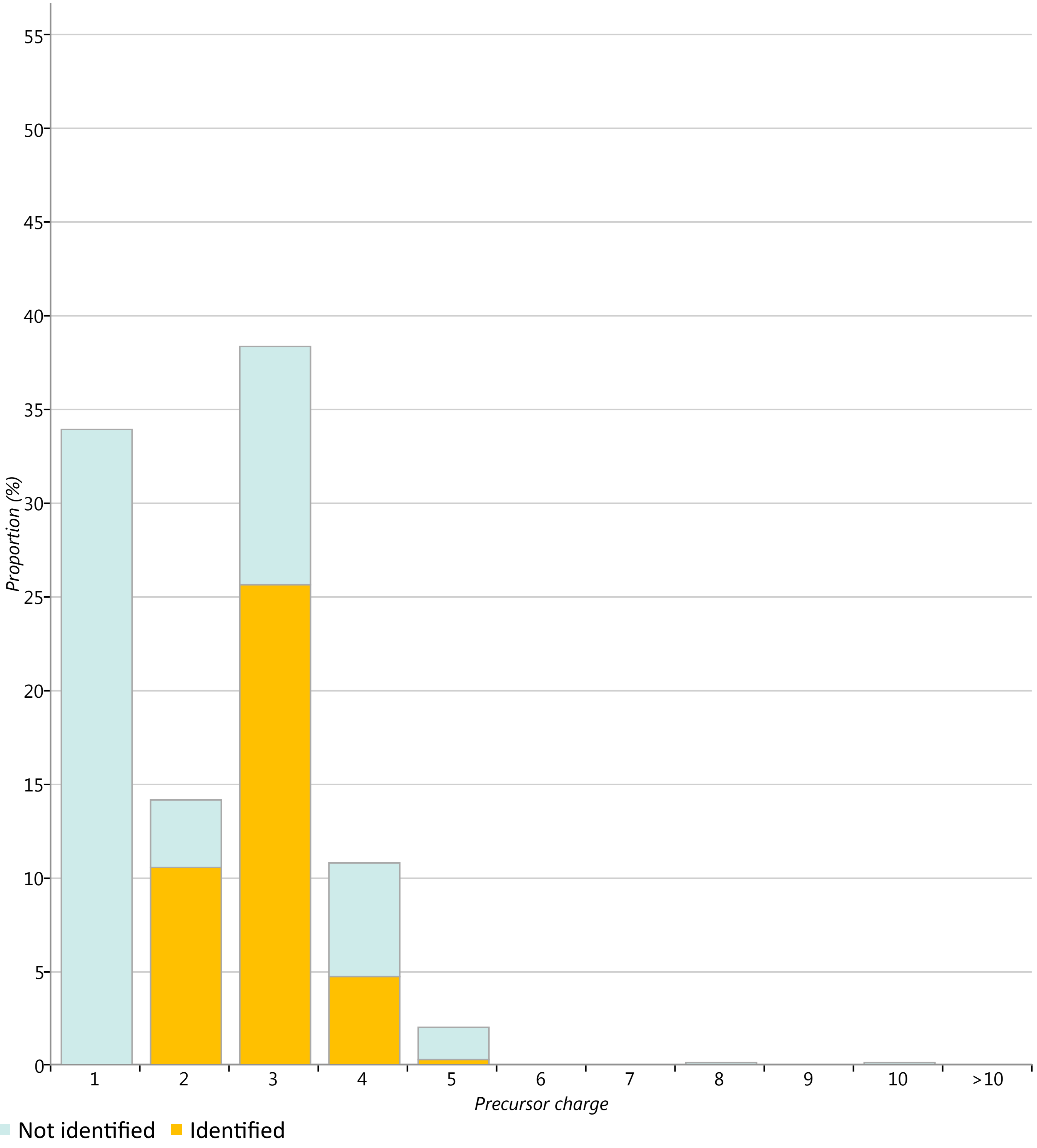

Missed cleavages (assuming trypsin)

This chart shows the distribution of missed cleavages per identified peptide ion across the whole experiment, assuming the proteins were treated with trypsin and using each peptide ion's highest scoring identification. For an alternative visualisation showing the data subdivided by experimental condition, see the *Missed cleavages* metric.

Potential problems:

- A high proportion of missed cleavages may indicate problems in trypsinization.
- An absence of missed cleavages may indicate that the database search parameters were too stringent.

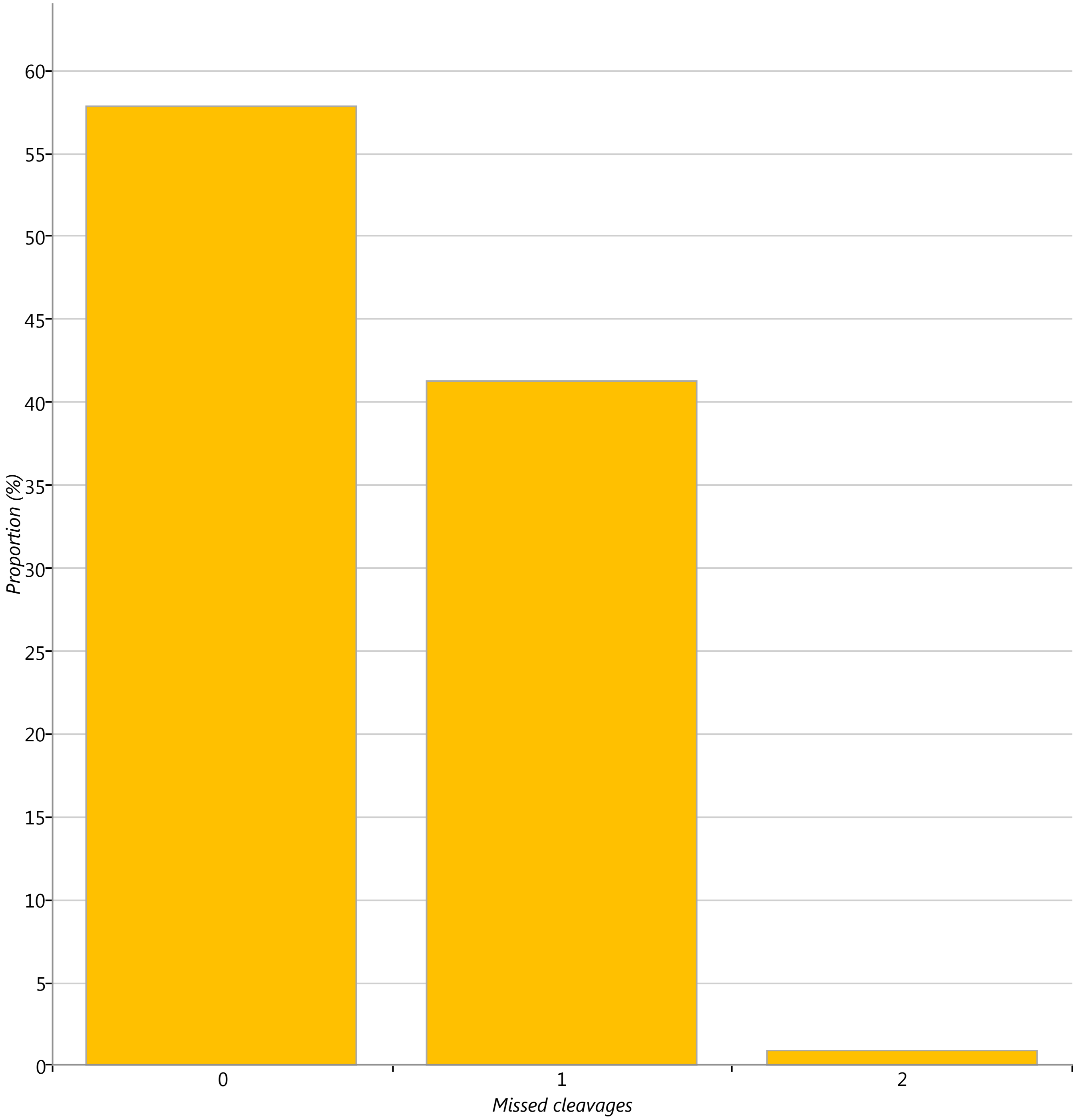

Peptides per protein (overview)

This chart shows the proportional distribution of the number of peptides identified per protein, combined across all runs in the experiment. For an alternative visualisation dividing these data by experimental condition, see the *Peptides per protein (by condition)* metric.

Potential problems:

- An unexpected bias to high or low numbers of peptides may indicate problems with sample preparation or peptide search settings.

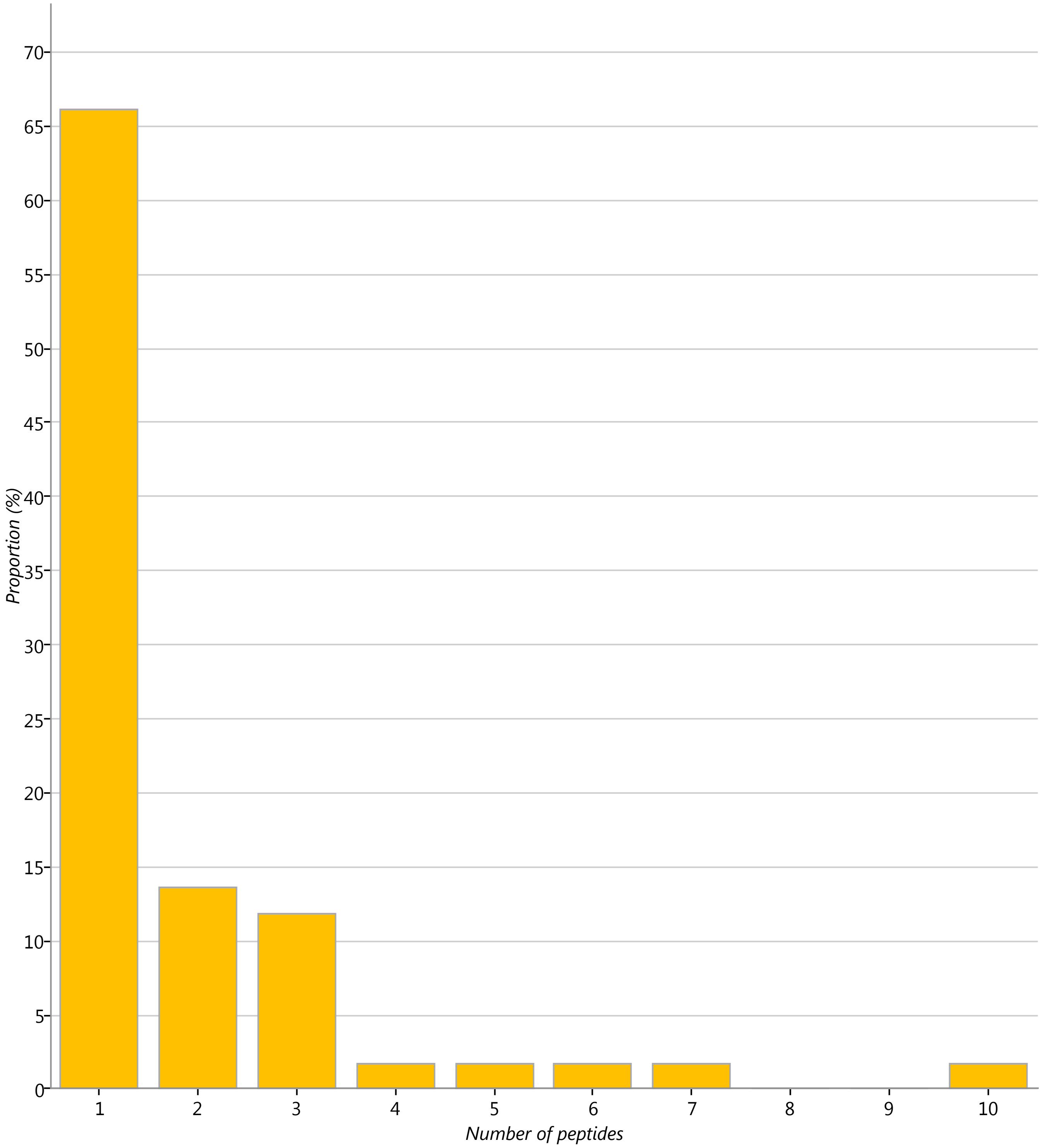

Modifications

The chart below shows the mean number of modifications per peptide ion for runs in the selected experiment design, based on each peptide ion's highest scoring identification. Each run is coloured according to its experimental condition.

Potential problems:

- Unexpectedly low numbers of modifications may indicate that one or more modifications were mistakenly not selected when searching for identifications.
- Variable numbers of modifications across the runs, especially within a given condition, may indicate problems with sample preparation.

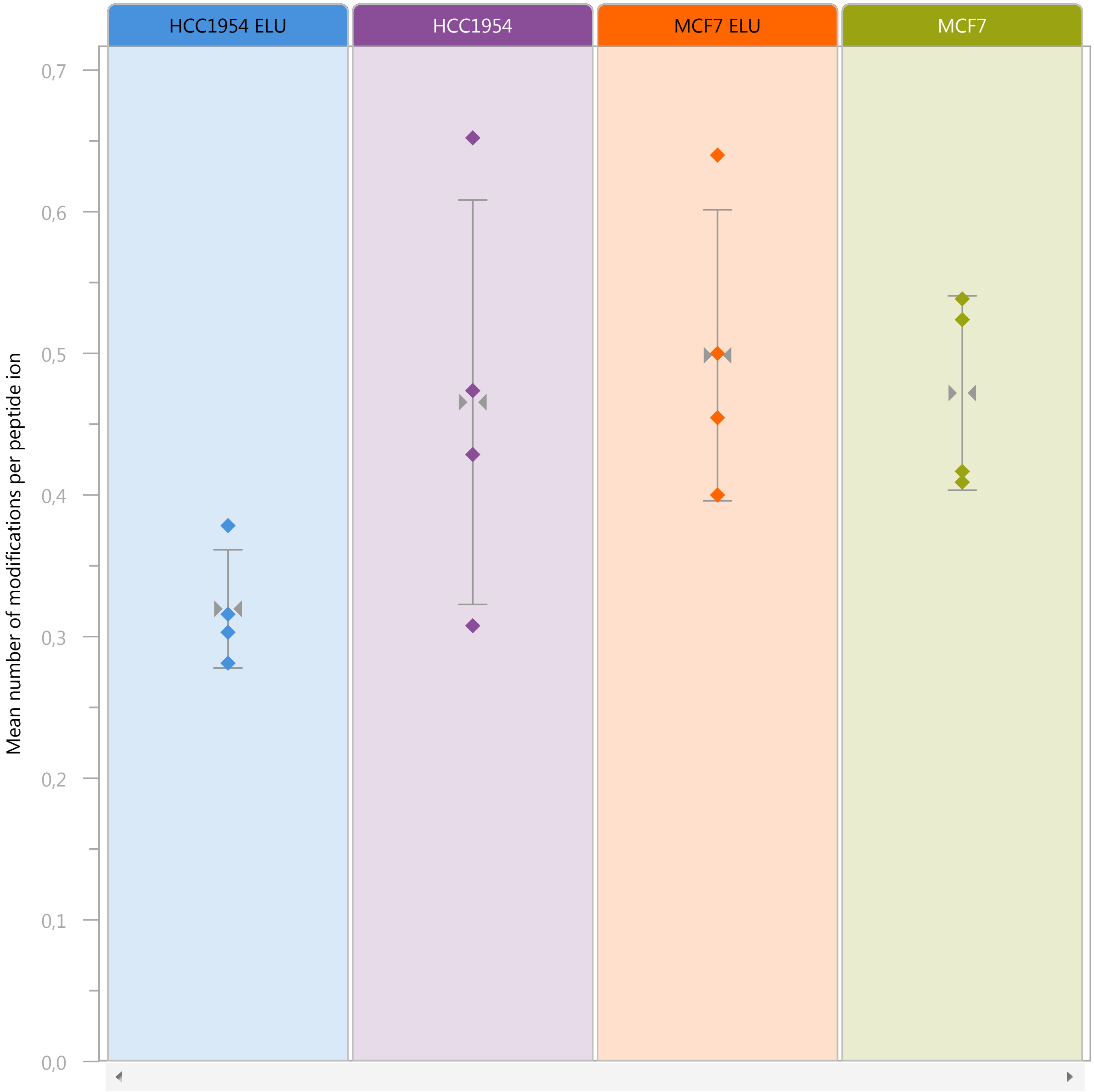

Included modifications Acetyl N-TERM Carbamidomethyl C Carbamyl N-TERM Deamidation N Dean

Missed cleavages

The chart below shows the mean number of missed cleavages per peptide ion for runs within the selected experiment design, based on each peptide ion's highest scoring identification. The chart assumes the use of trypsin for digestion. Each run is coloured according to its experimental condition.

Potential problems:

- A high number of missed cleavages may indicate problems in trypsinization.
- An absence of missed cleavages may indicate that database search parameters were too stringent.

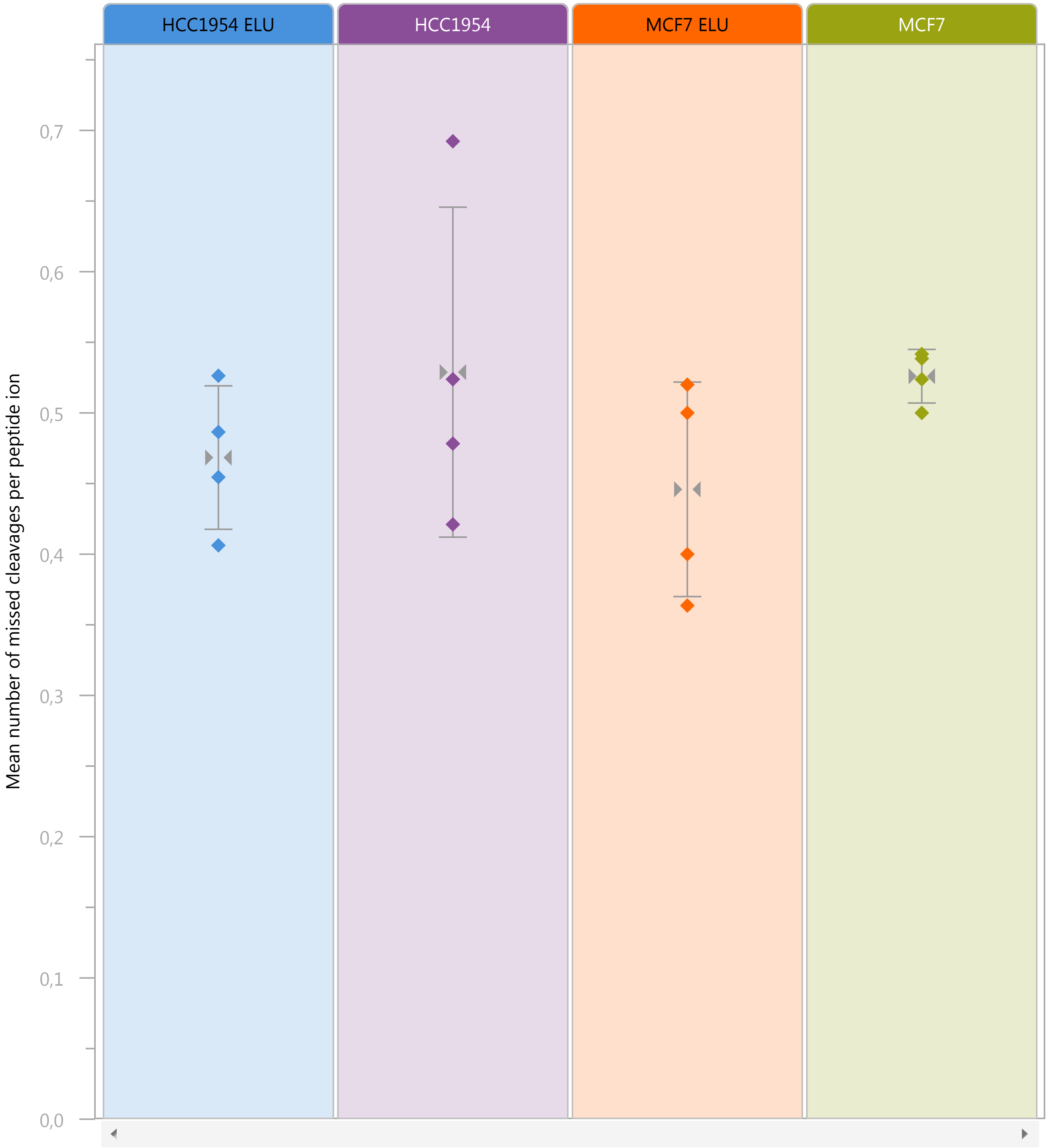

Abundance dynamic range

The charts below show abundance histograms for peptide ions, calculated using the current experiment design. Each chart shows the mean abundance for peptide ions within an experimental condition's runs. Data are binned by Log10 of their normalised abundance and shown as a proportion of the total. Identified peptide ions are shown in yellow.

Potential problems:

- A heavily right-skewed histogram of *identified* peptide ions indicates you may be missing identifications with low abundance.
- A heavily right skewed total (summed identified and unidentified) histogram may indicate saturation at high abundance, which will affect the accuracy of quantitation.

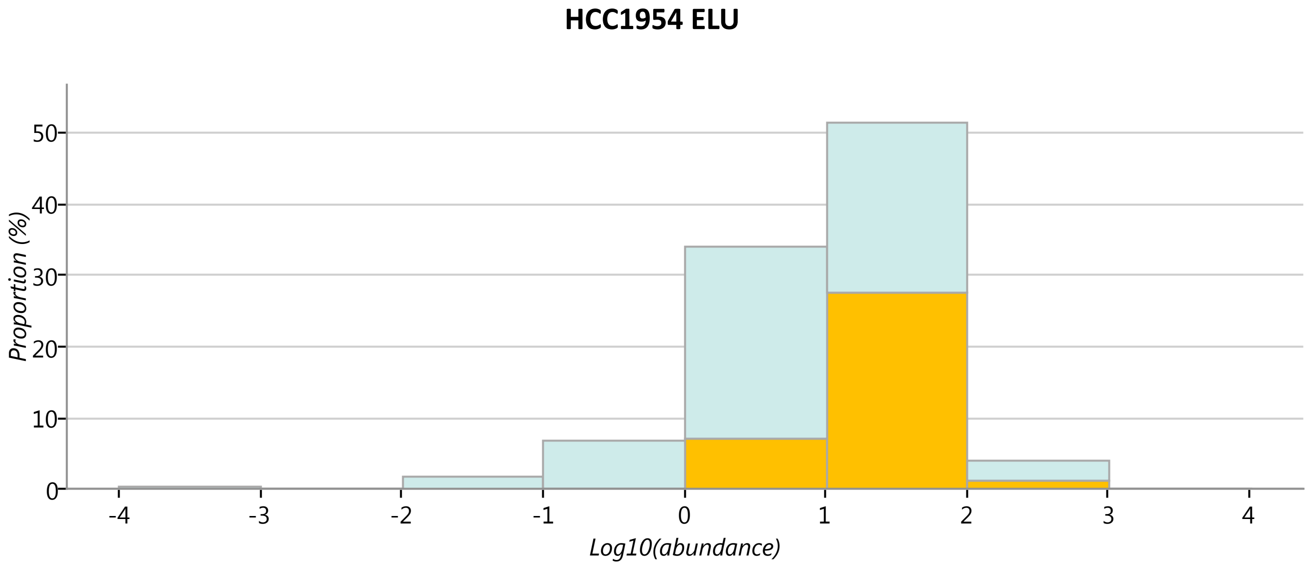

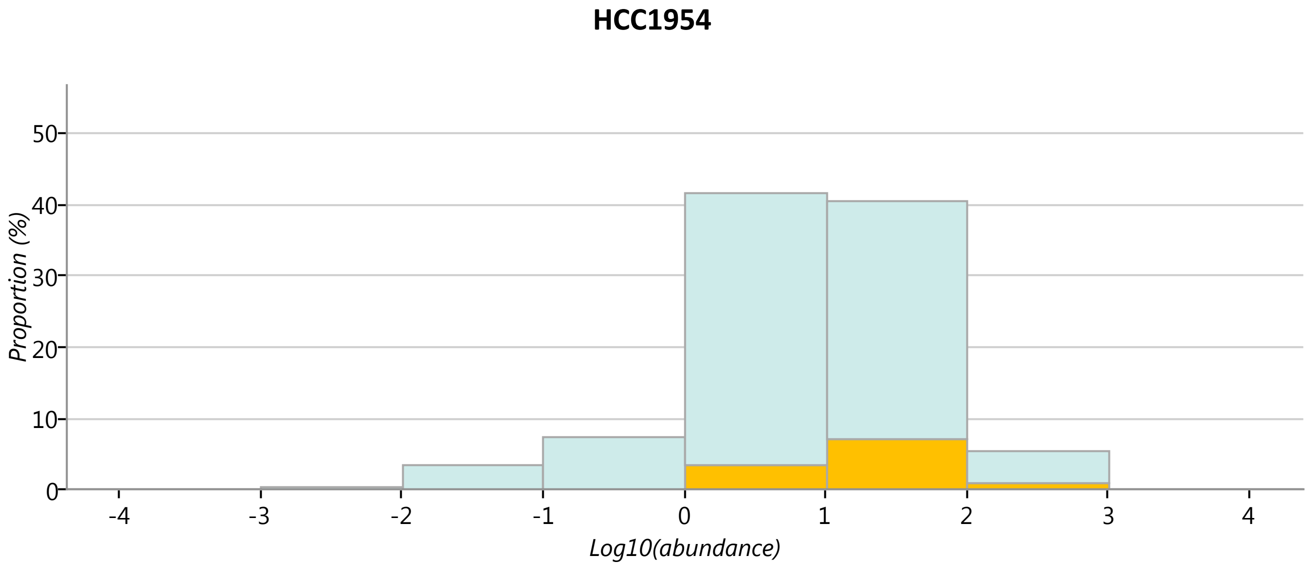

Not identified ■ Identified

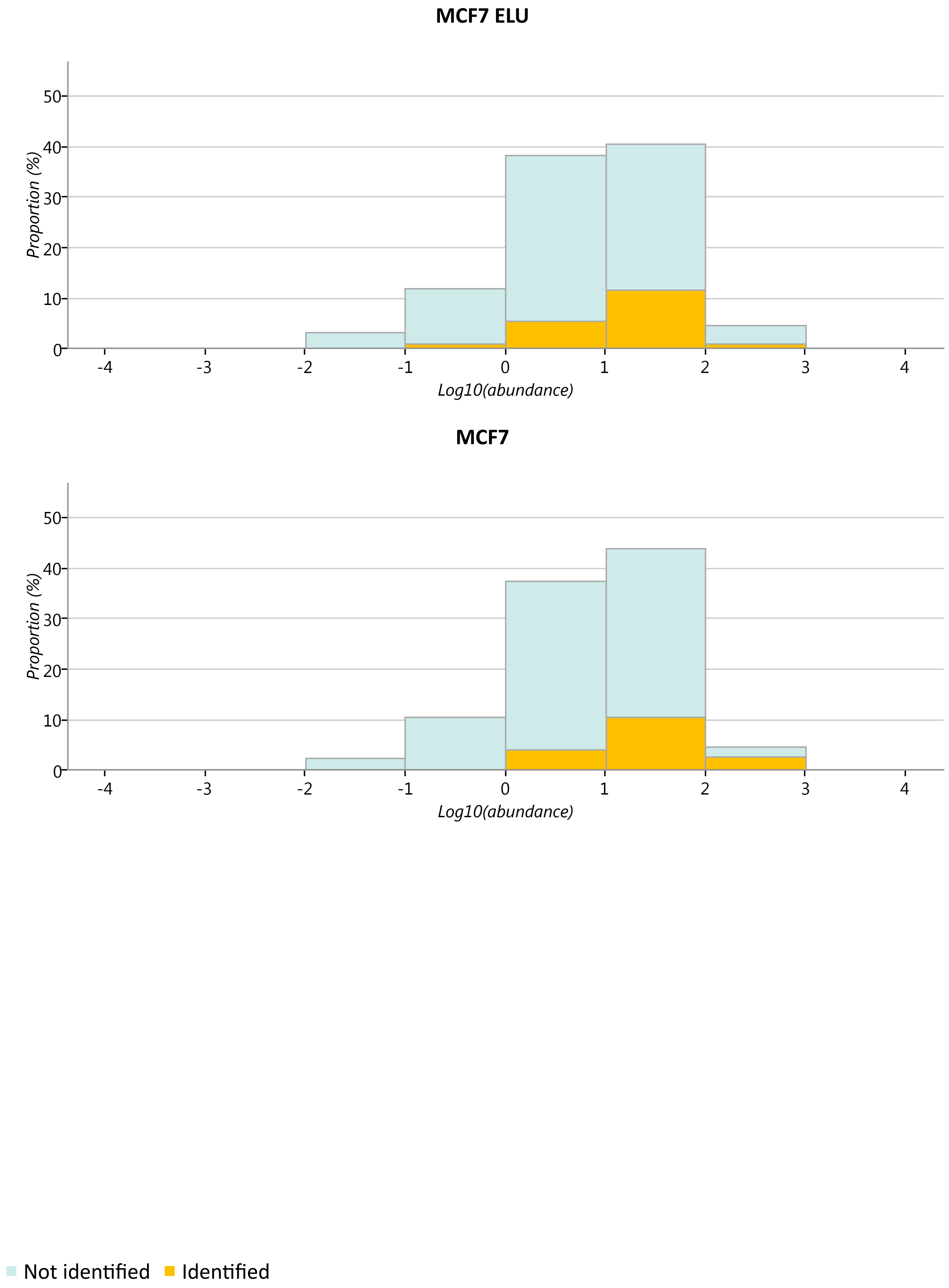
MCF7 ELU
Proportion (%)
0
10
20
30
40
50
-4
-3
-2
-1
0
1
2
3
4
Log10(abundance)
MCF7
Proportion (%)
0
10
20
30
40
50
-4
-3
-2
-1
0
1
2
3
4
Log10(abundance)
Not identified
Identified

Mass accuracy

The chart below shows all identified peptide ions in your experiment, plotting peptide ion m/z against the ppm error in its highest scoring identification. Blue points indicate peptide ions which have a mass error within 2 standard deviations of the mean, yellow points indicate peptide ions with a mass error more than 2 standard deviations from the mean.

Potential problems:

- A dependence of ppm mass error on m/z may indicate poor calibration.
- If the standard deviation of the data is large in ppm terms, this may indicate that your search parameters are too lenient.

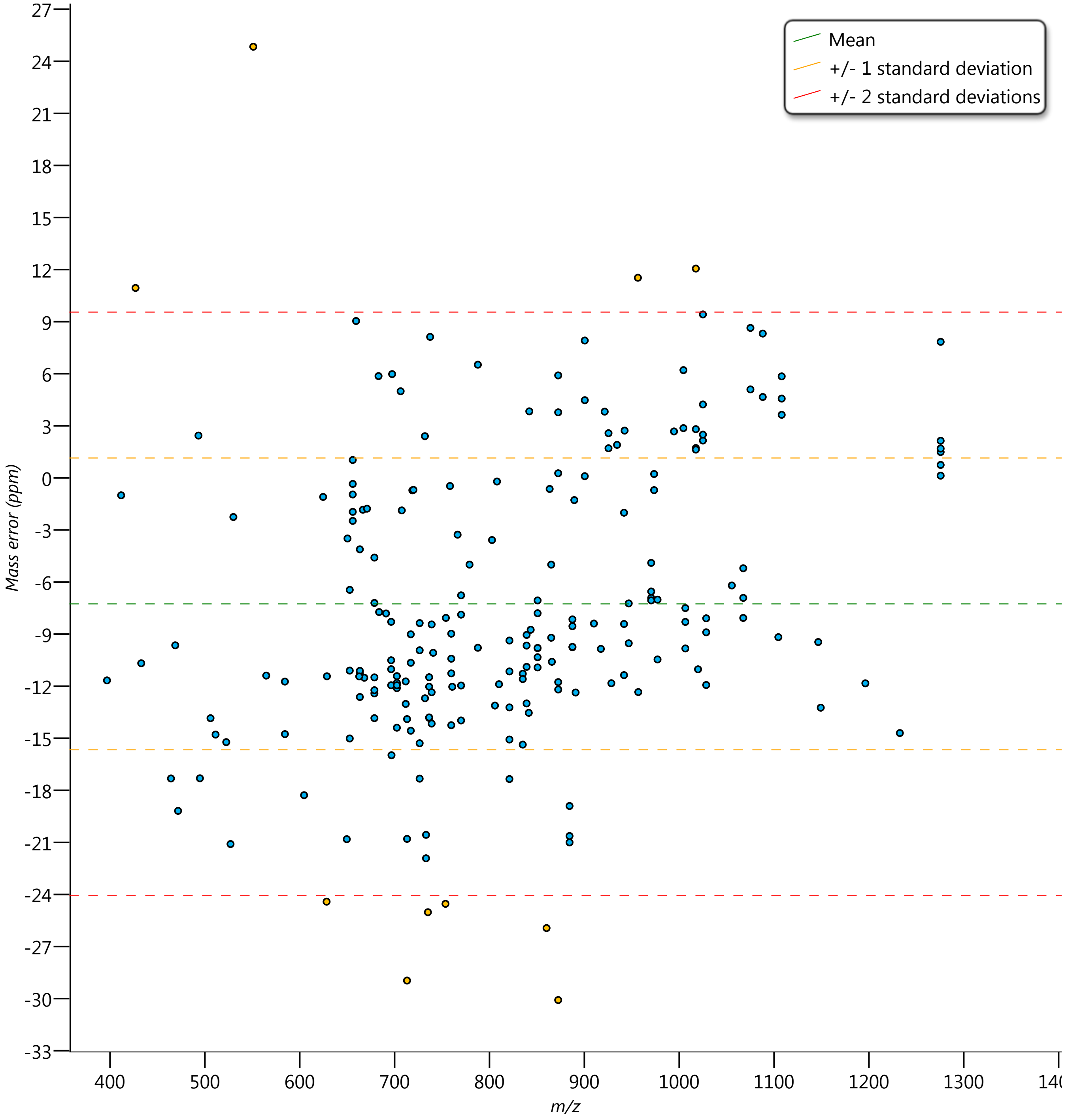

Scan rates

The charts below show the MS1 scan rate over the duration of your runs, using the current experiment design. Each chart shows the scan rate variation within an experimental condition's runs. The named run is highlighted in black. The variability between runs is shown as a grey area. The y-axis shows scans per second in a 30-second window centred on each x-axis time point.

Potential problems:

- A highly varying scan rate means quantitation accuracy may be negatively affected.

HCC1954 ELU **Run:** 20140724_DAP_HCC1954_ELUIDO_2ug_inj5_STEP7

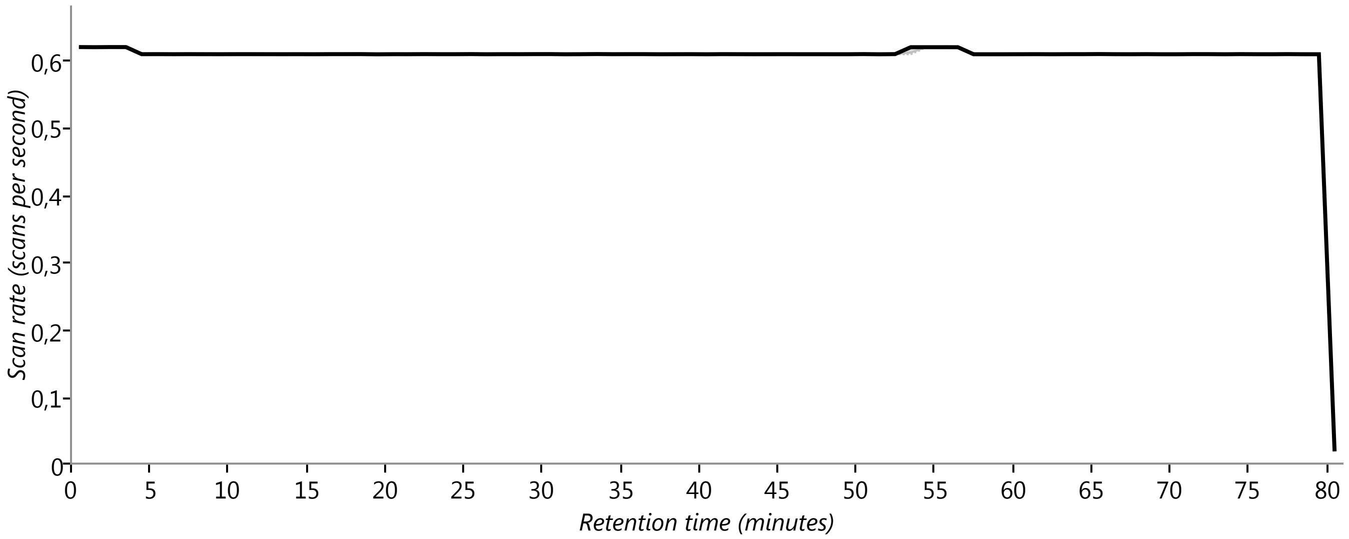

HCC1954 **Run:** 20140801_DAP_HCC1954_NAO_RETIDO_2ug_inj1_STEP7

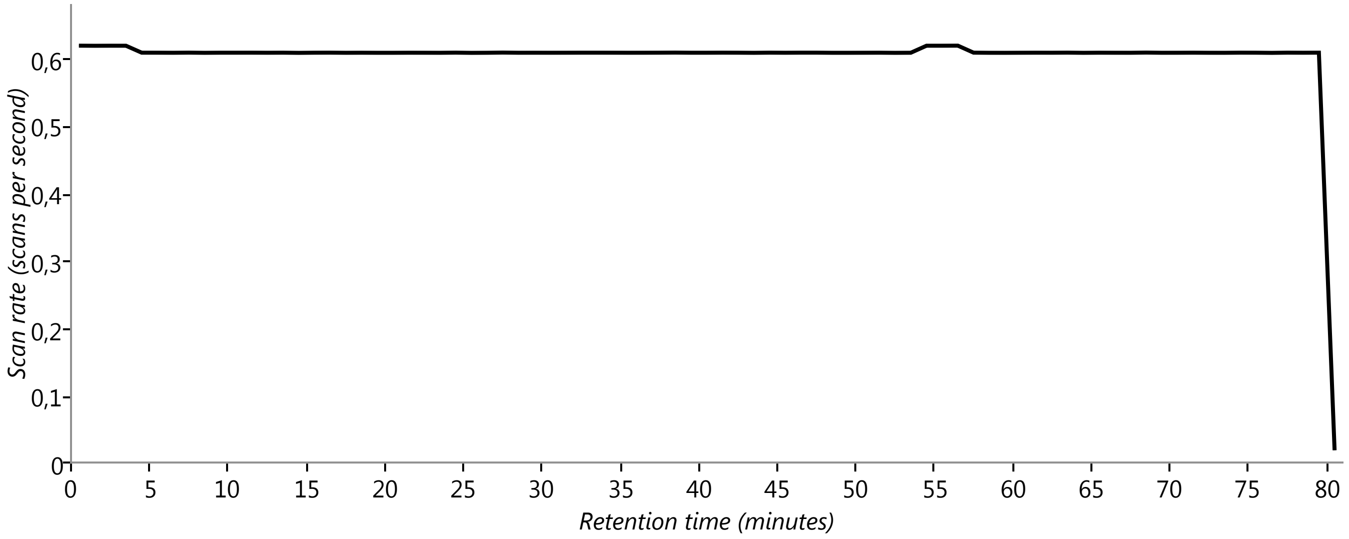

MCF7 ELU

Run: 20140724_DAP_MCF7_ELUIDO_2ug_inj3_STEP7

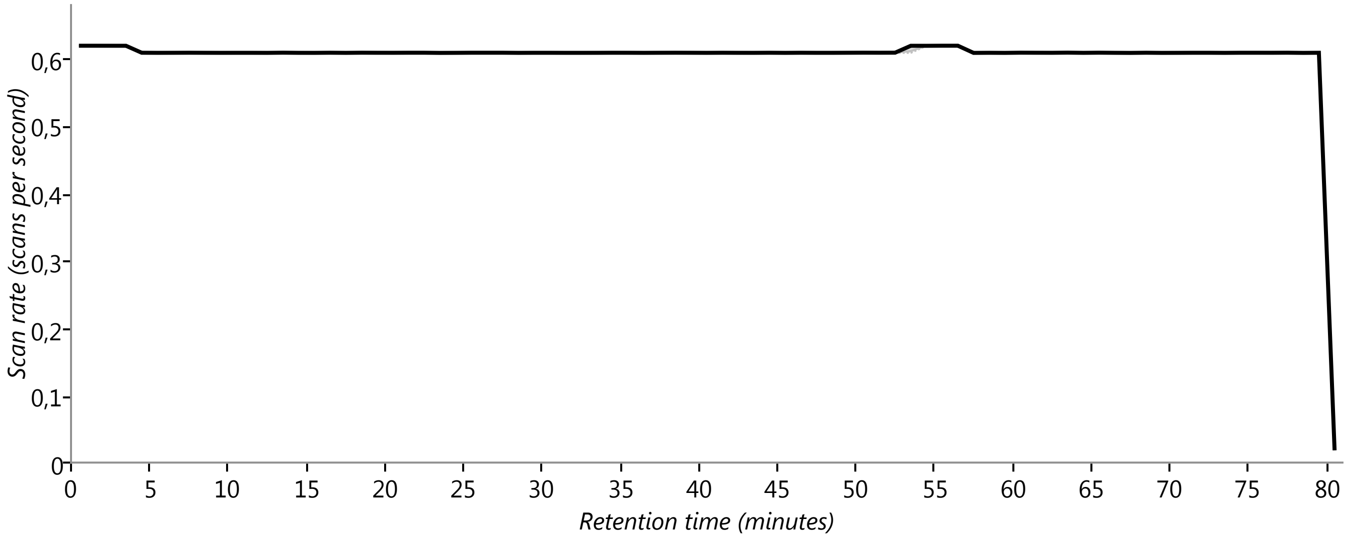

MCF7

Run: 20140801_DAP_MCF7_NAO_RETIDO_2ug_inj2_STEP7

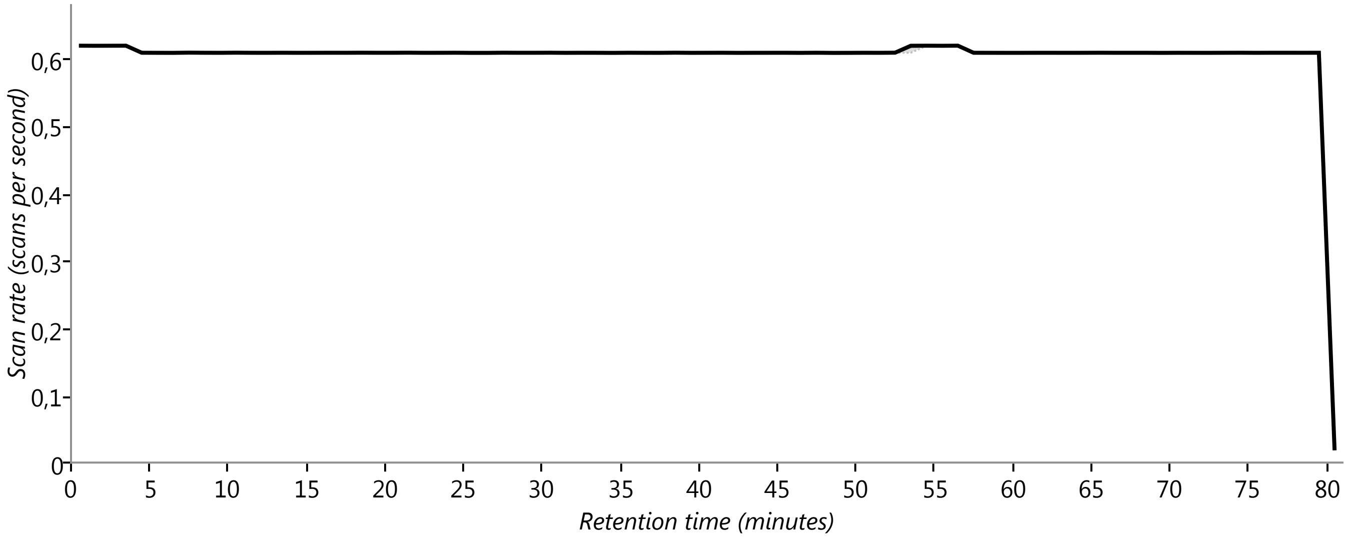

Proteins

The chart below shows the number of proteins identified in each of the runs within the selected experiment design. Each run is coloured according to its experimental condition.

Potential problems:

- Any exceptionally high or low values may indicate problems with sample preparation, chromatography, or instrument settings.

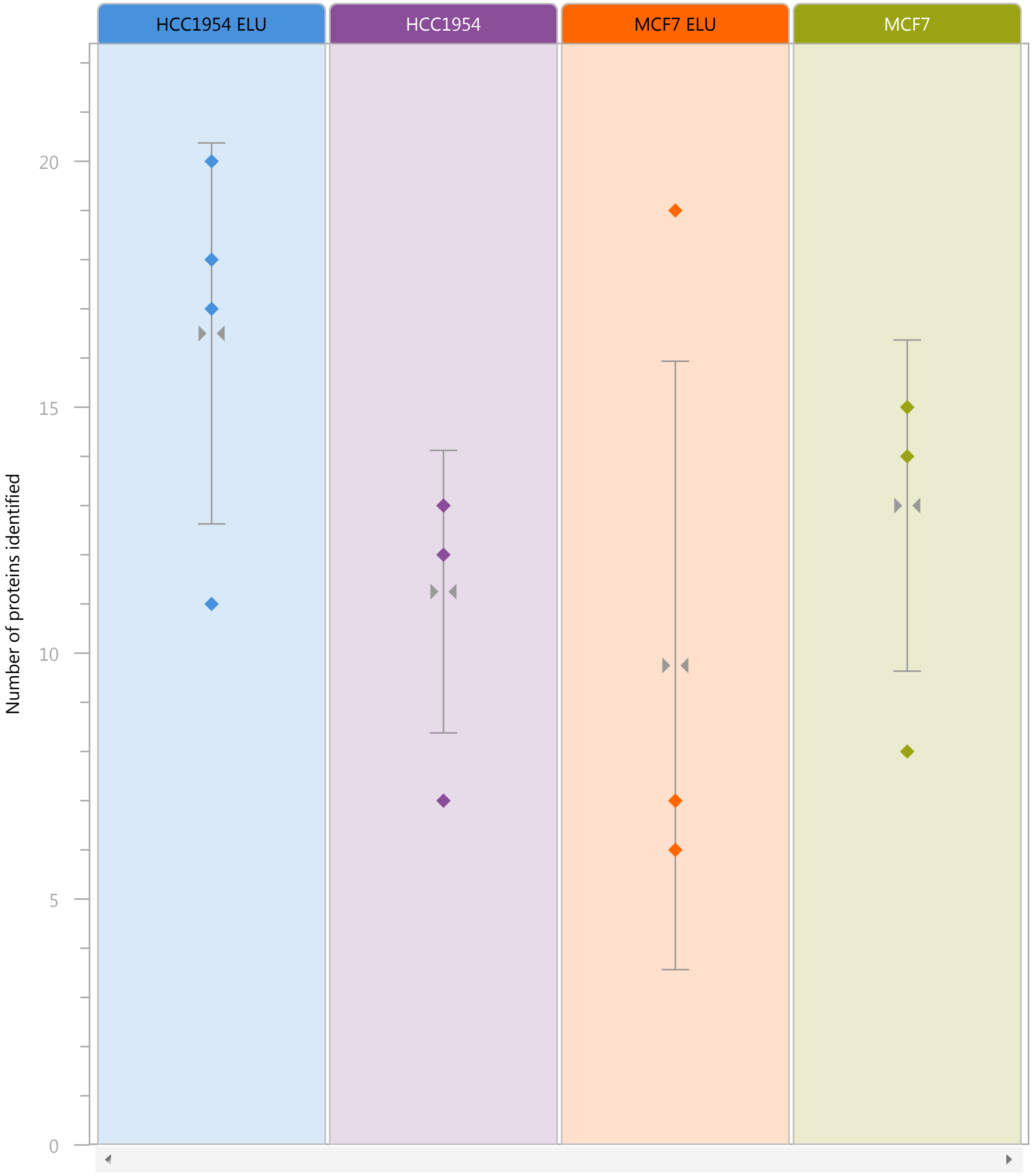

Peptides

The chart below shows the number of peptides identified in each of the experiment's runs. Each run is coloured according to its experimental condition.

Potential problems:

- Any exceptionally high or low values may indicate problems with sample preparation, chromatography, or instrument settings.

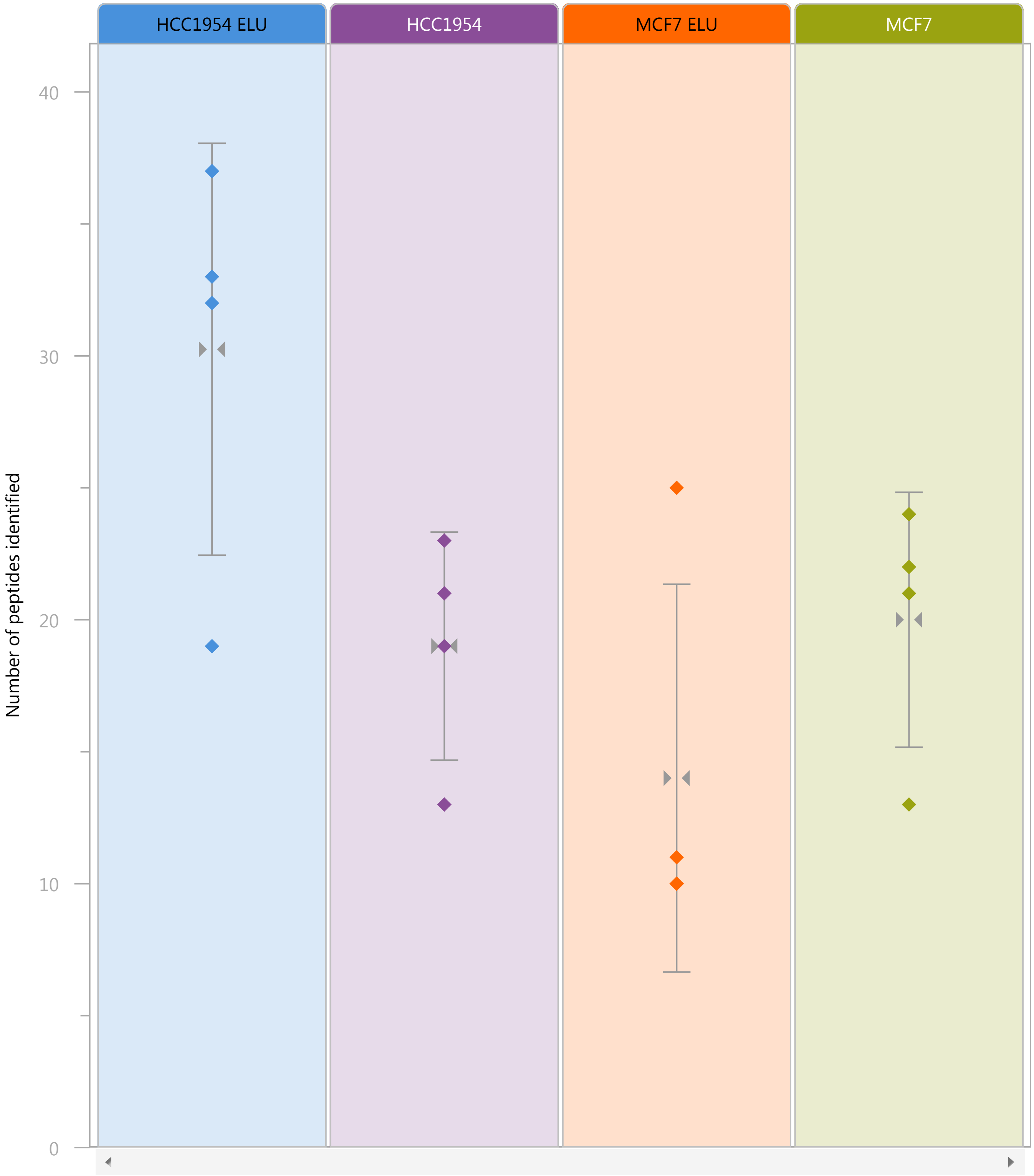

Peptides per protein (by condition)

The chart below shows the average number of peptides per protein in each run within the selected experiment design. For a given run, the value shown is calculated using only identifications from that run.

Potential problems:

- Any exceptionally high or low values may indicate problems with sample preparation or peptide search settings.

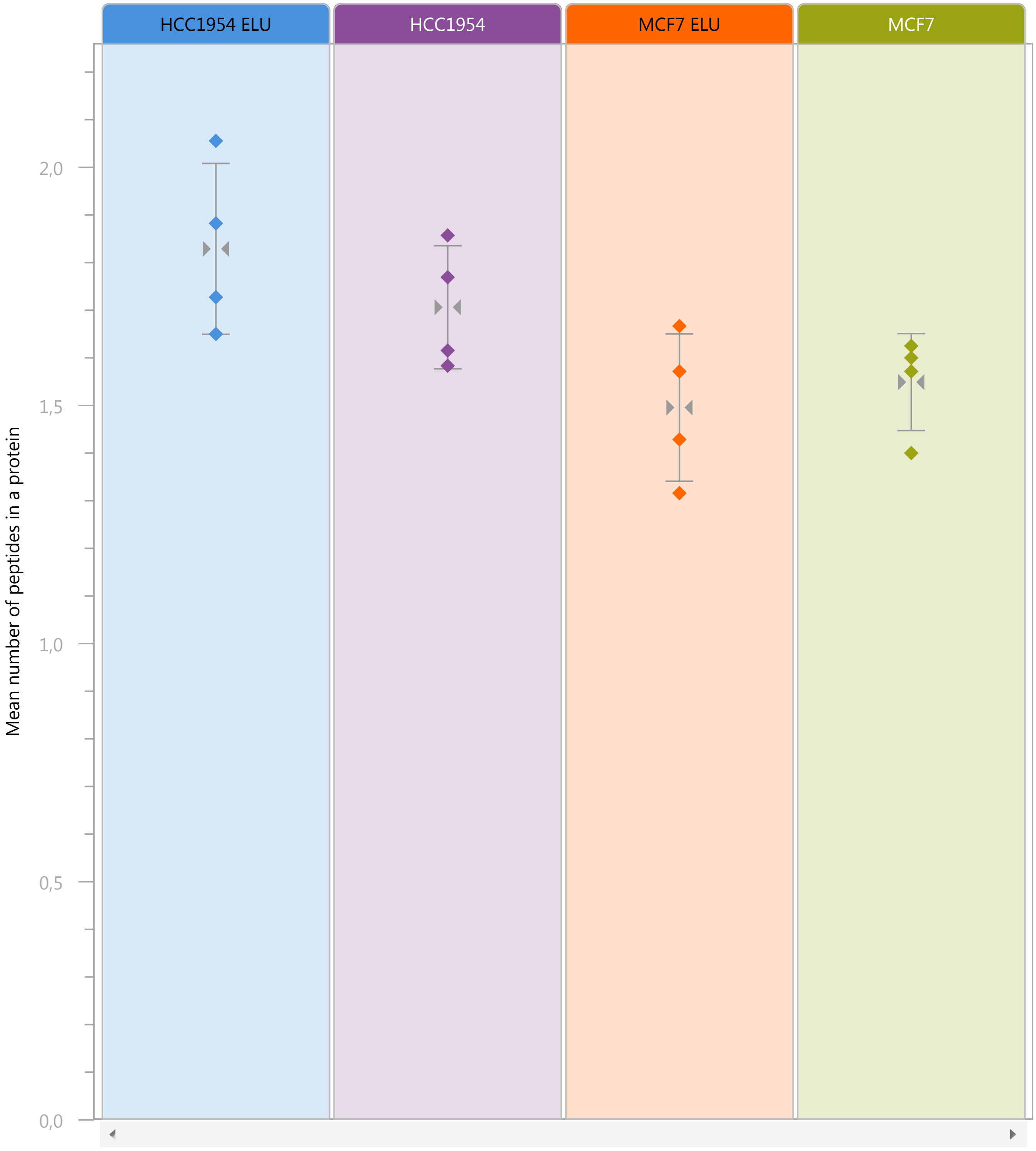

Percentage of peptide ions identified

The chart below shows the number of peptide ions identified in each of the runs within the selected experiment design, expressed as a percentage of the total number of peptide ions. Each run is coloured according to its experimental condition.

Potential problems:

- Any exceptionally high or low values may indicate problems with sample preparation, chromatography, or instrument settings.
- Very low values across all runs may indicate a problem with the database that was searched for identifications, or with other search parameters.

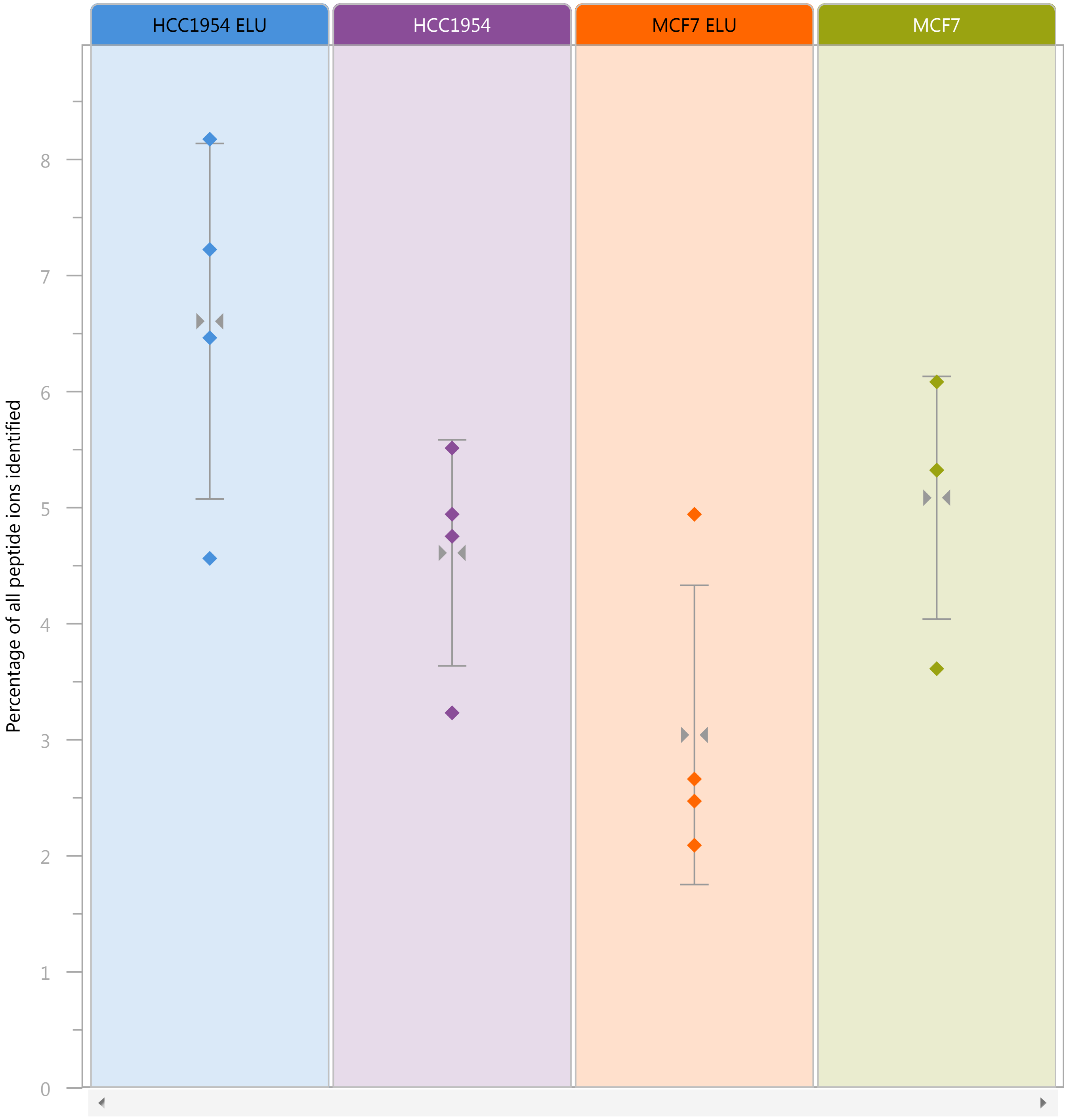

Protein overlap

The Venn diagram shows the number of proteins identified uniquely in the selected conditions and the number that are shared between conditions. Use the list on the right to select two or three conditions to explore.

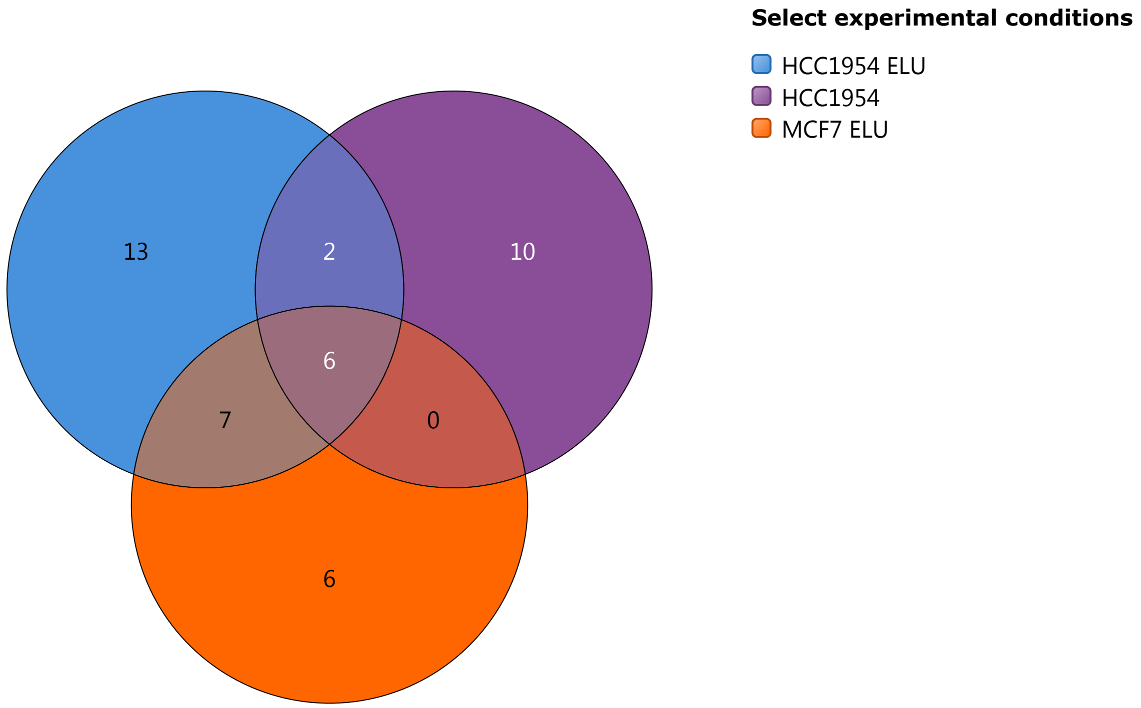

HCC1954 X MCF7 STEP8

QC Metrics reported by Progenesis QI for proteomics v2.0.5556.29015
Date: 10-Jan-2020 at 09:42
Experiment design: HCC1954 X MCF7 STEP8

This QC report contains **all metrics** for the experiment named above.

Reported metrics

0 of the 19 metrics reported have been manually flagged for attention.

- Identifications overview
- LC peak width
- Peptide ion dynamic range
- Precursor m/z
- Precursor retention time
- Mass errors
- Precursor charges
- Missed cleavages (assuming trypsin)
- Peptides per protein (overview)
- Modifications
- Missed cleavages
- Abundance dynamic range
- Mass accuracy
- Scan rates
- Proteins
- Peptides
- Peptides per protein (by condition)
- Percentage of peptide ions identified
- Protein overlap

Identifications overview

The chart below provides the number of identifications per run by category. Data are plotted for all runs in the current experiment design. The box represents the interquartile range and the whiskers the maximum and minimum.

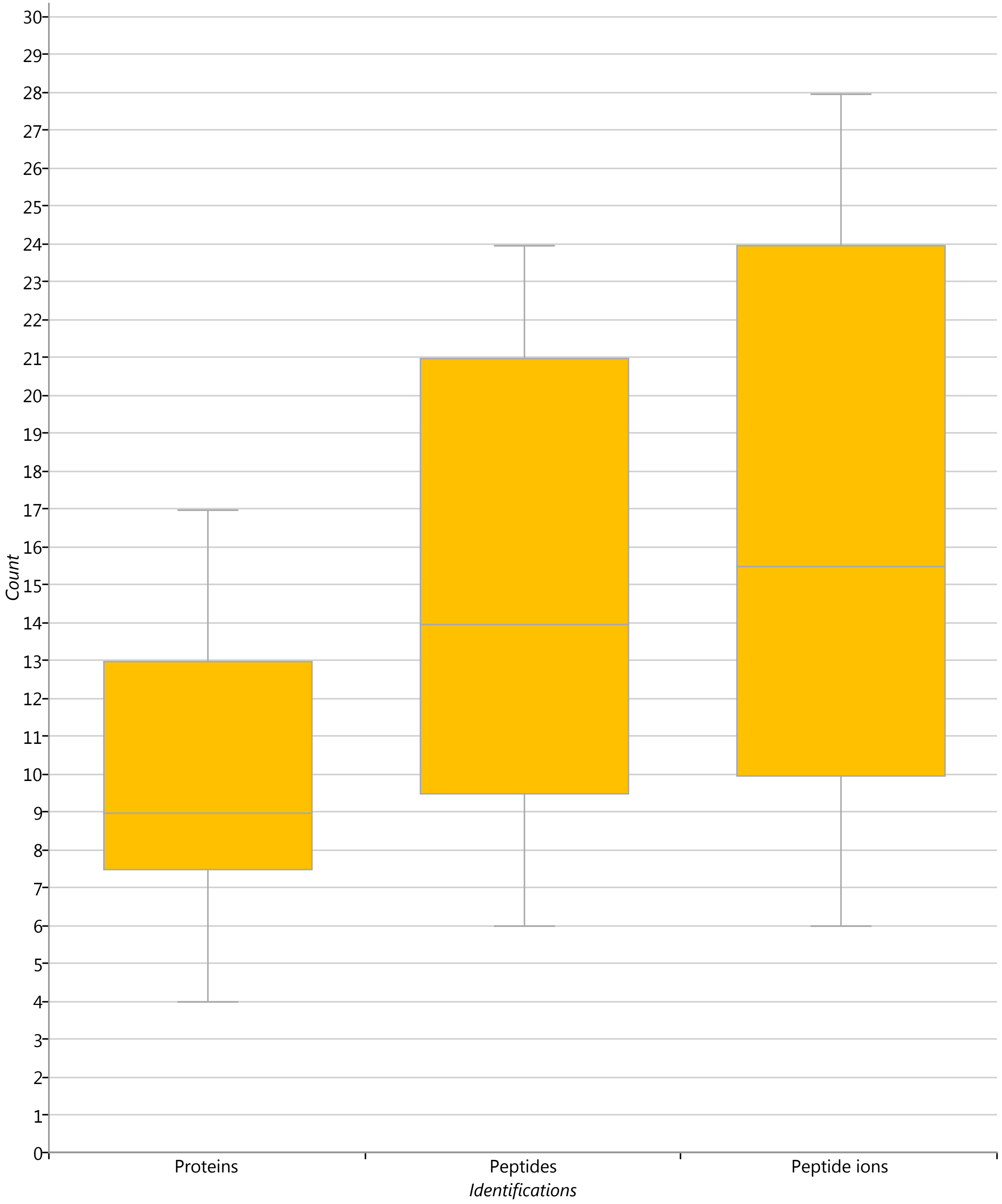

LC peak width

This chart shows the proportional distribution of peak widths in retention time, for all the detected peptide ions in the experiment. Widths are binned into categories for simple comparison. Identified peptide ions are shown in yellow.

Potential problems:

- A peak width that is different from that expected for your setup may indicate a problem with the chromatographic separation.

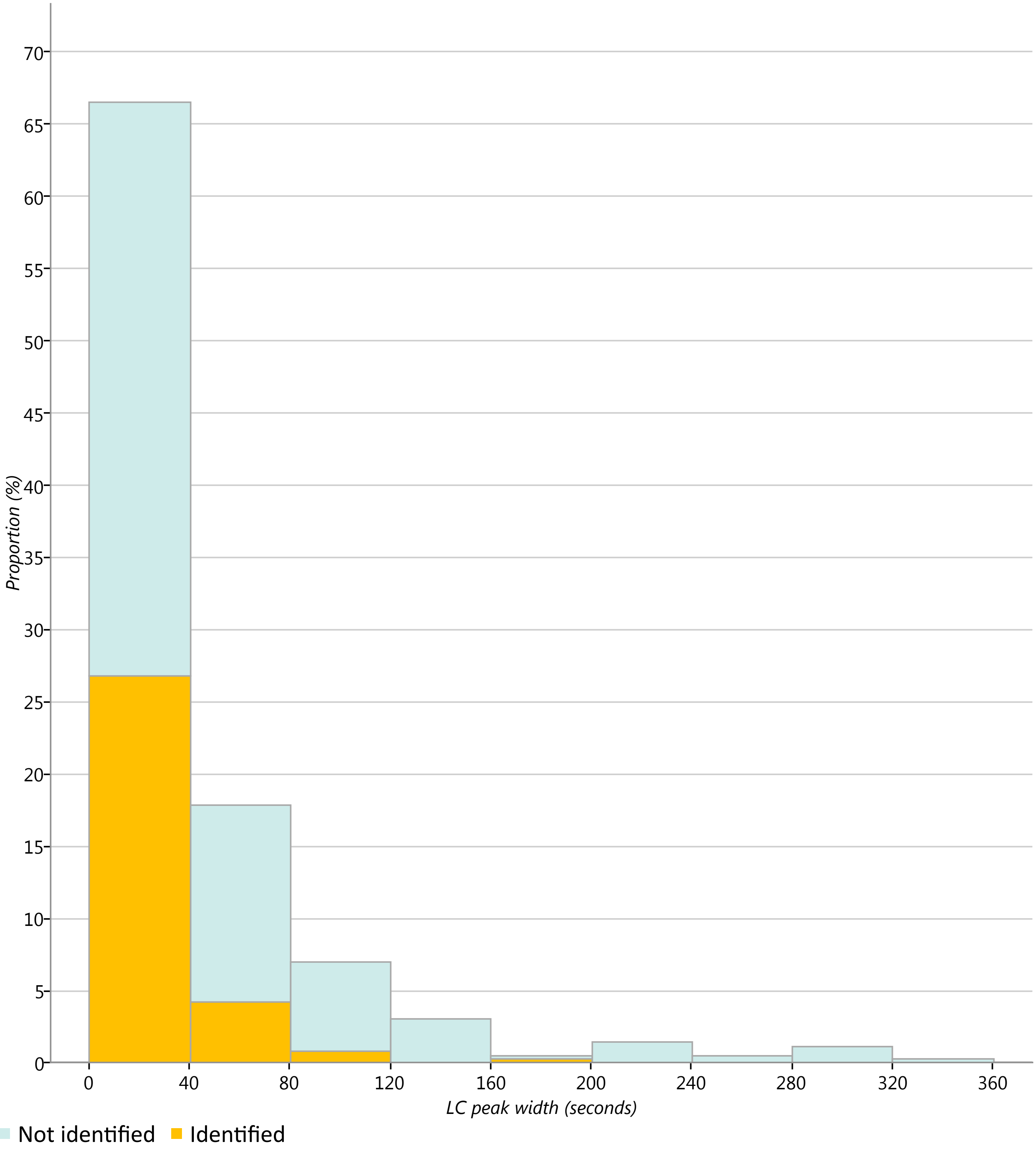

Peptide ion dynamic range

This chart shows log abundance for peptide ions, calculated using the current experiment design. Each peptide ion is assigned its abundance based on the highest mean value for any condition's runs. Data are binned and shown as a proportion. Identified peptide ions are shown in yellow.

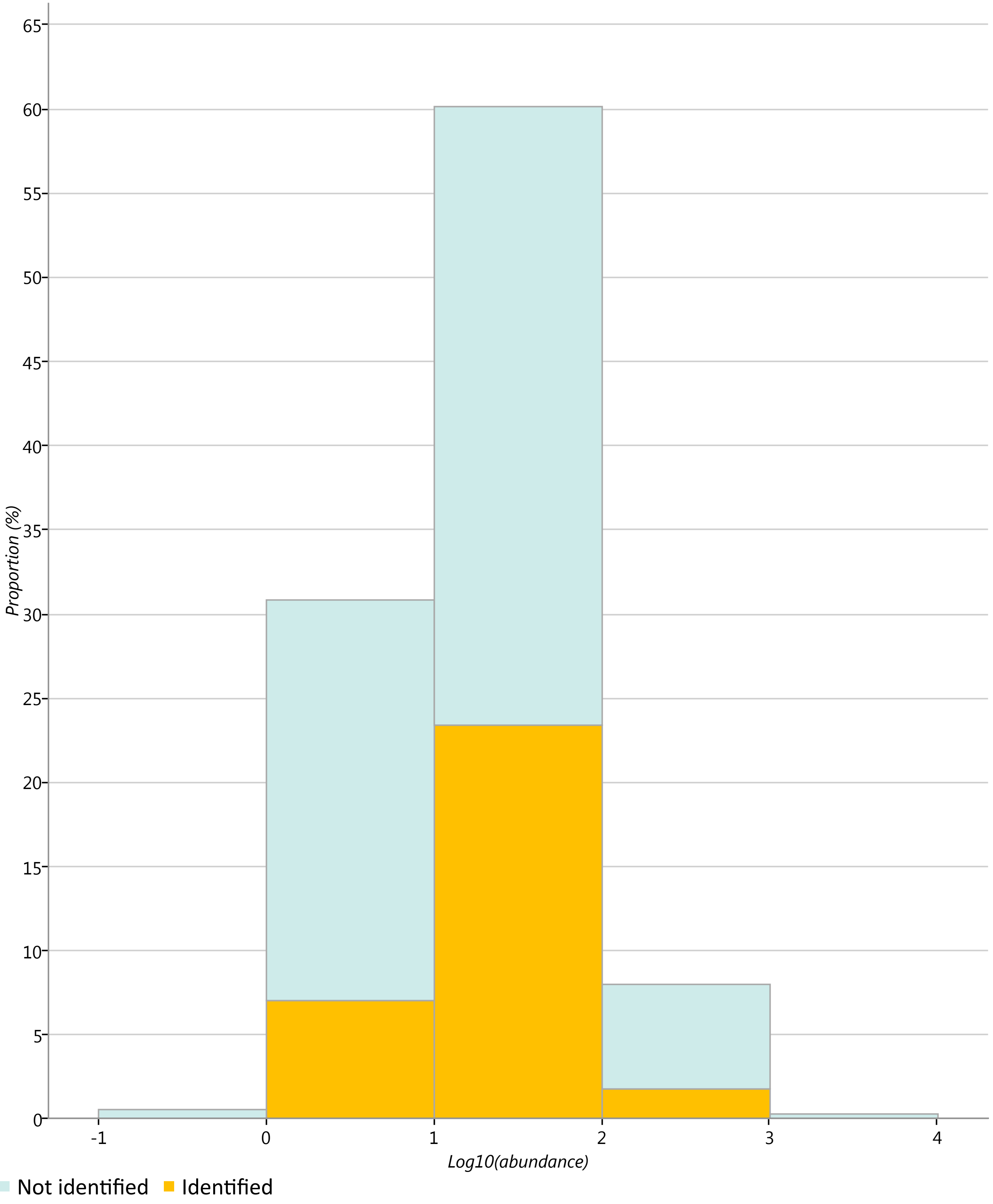

Precursor m/z

This chart shows the distribution of m/z values for all peptide ions in the experiment. Identified peptide ions are shown in yellow.

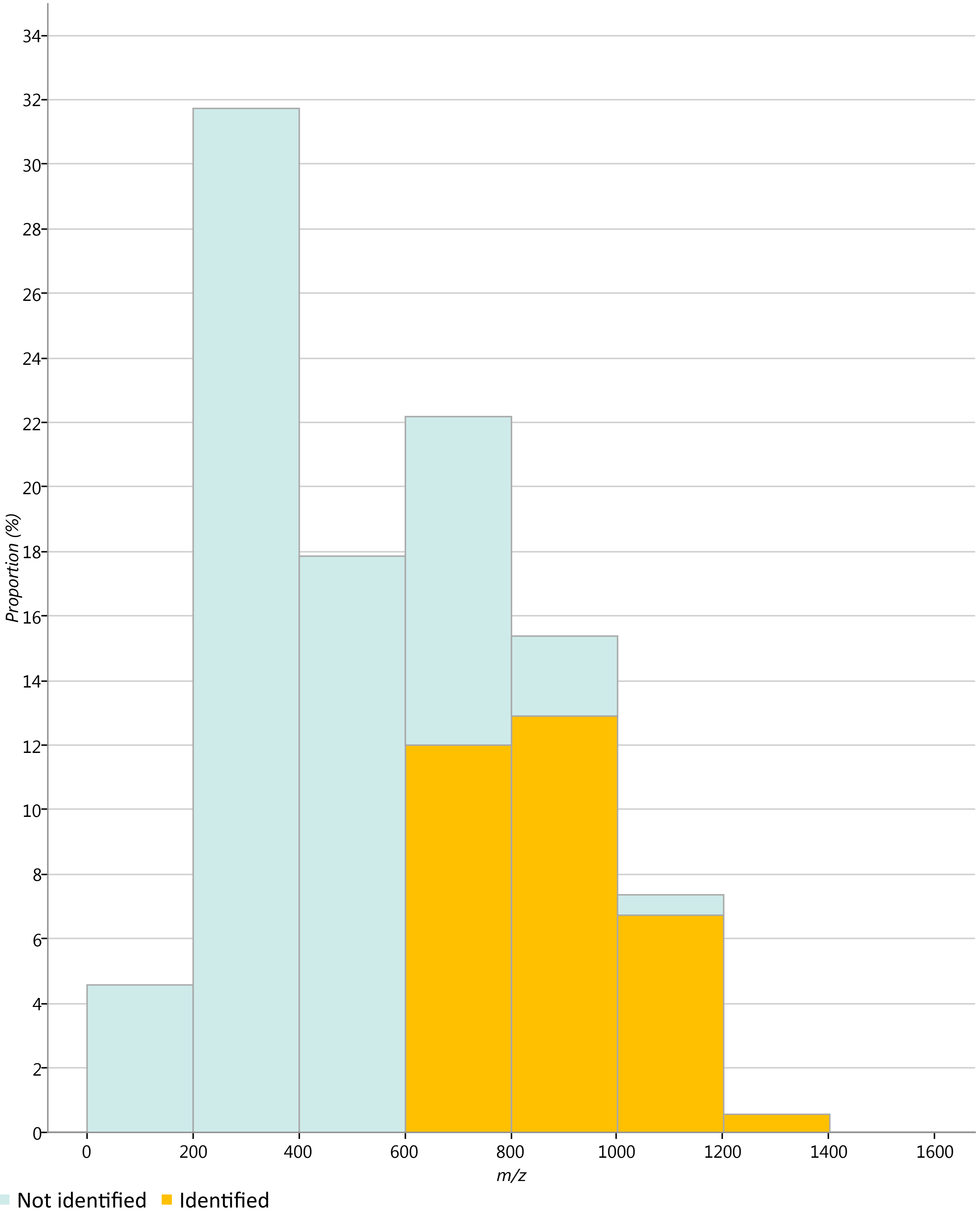

Precursor retention time

This chart shows the proportion of precursor peptide ions eluting into each quartile of the LC run. Identified peptide ions are shown in yellow.

Potential problems:

- A majority of peptide ions eluting at the start or end of the run may indicate problems with the chromatographic separation or that the setup is not ideal for a successful separation.

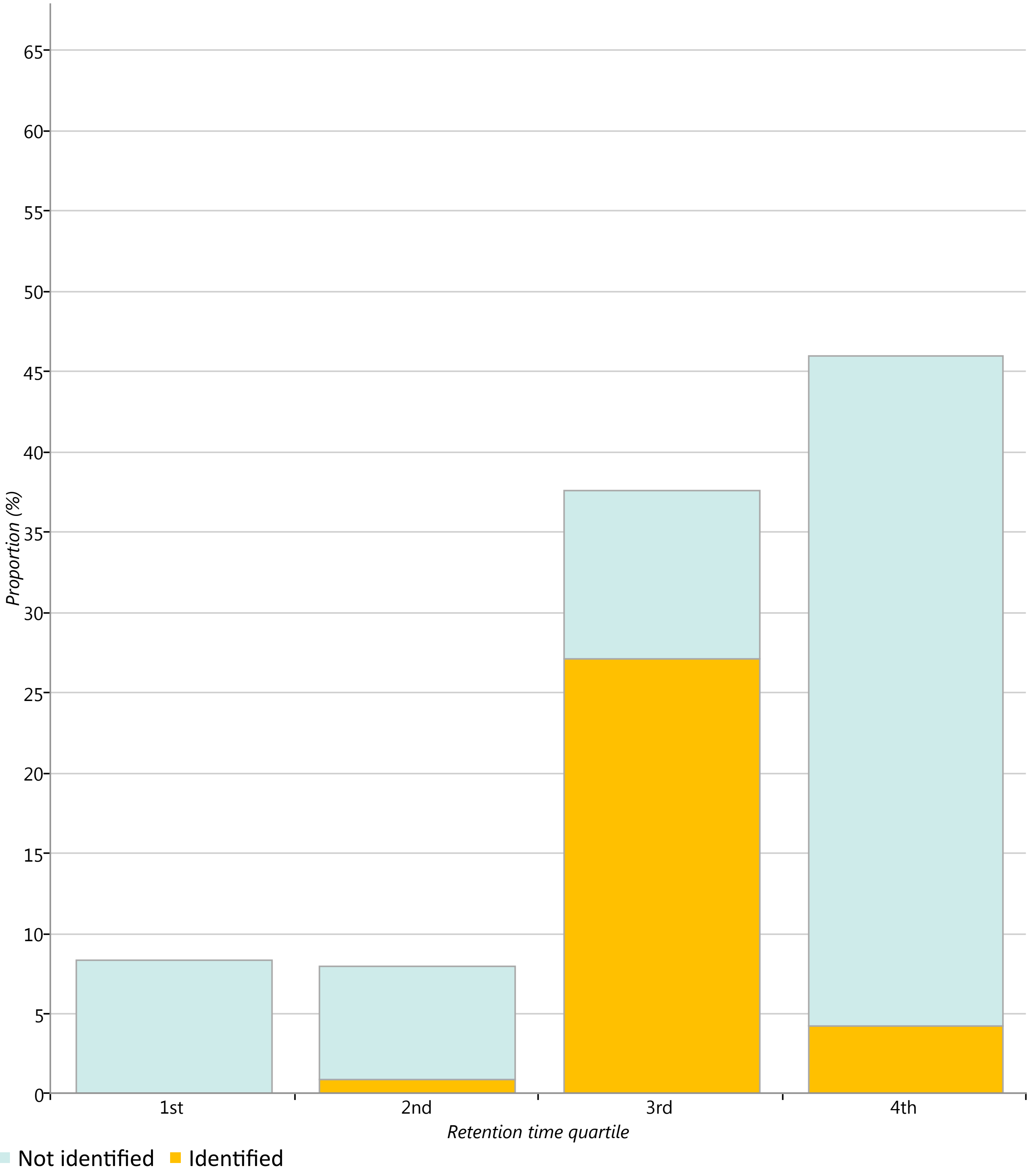

Mass errors

This chart shows the mass error calculated for identified peptide ions. The proportion of peptide ions with a given binned error value is plotted. For a more detailed plot, see the *Mass accuracy* metric.

Potential problems:

- A larger than expected spread could indicate that search parameters are too lenient.
- A bias of the peak from zero ppm could indicate instrument bias.

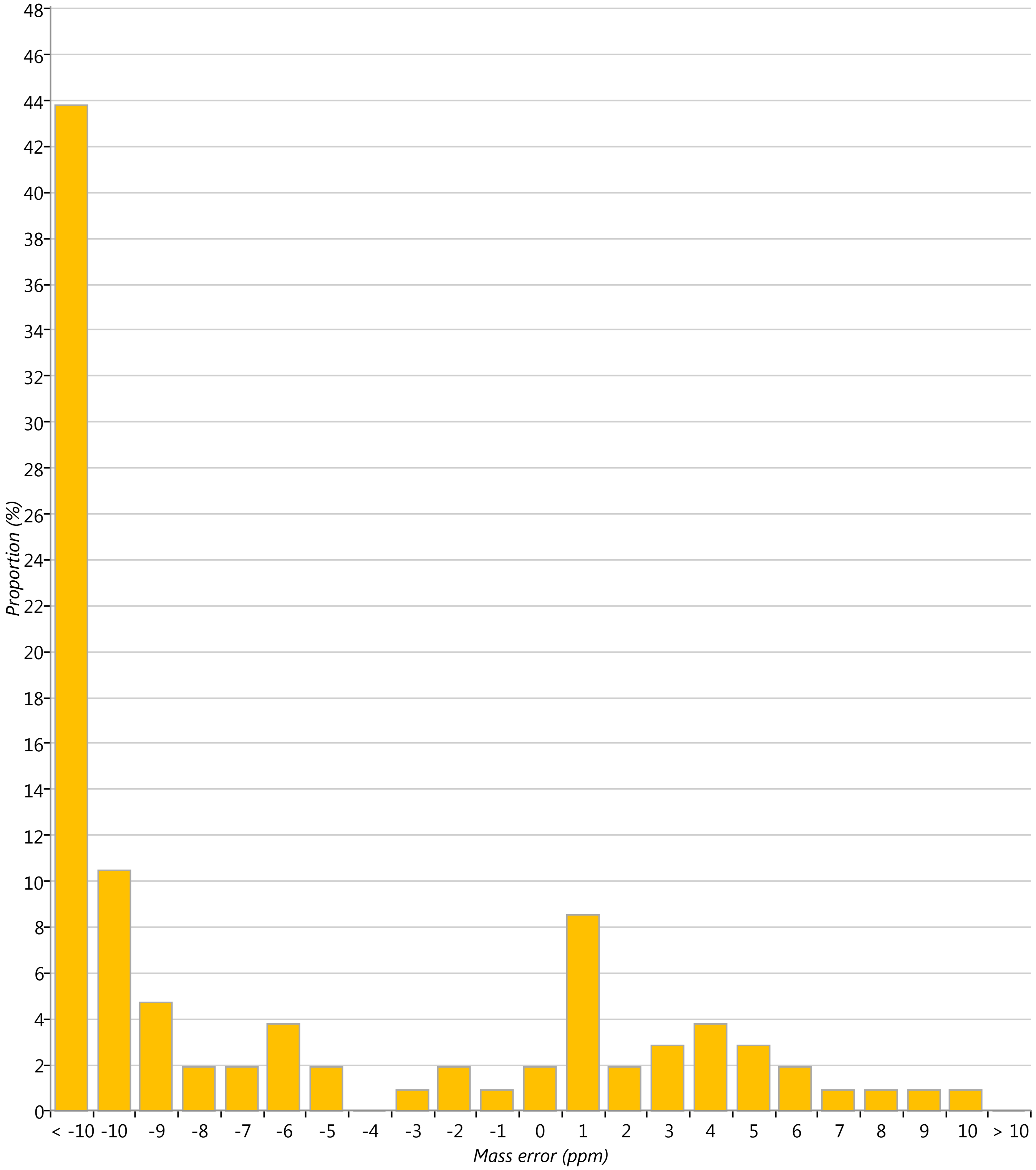

Precursor charges

This chart shows the percentage of peptide ions with various charge states.

Potential problems:

- A skew towards low charge peptide ions may indicate poor ionisation, suggesting that you may need to raise the collision voltage.
- A skew towards high charge peptide ions may indicate excessive ionisation, suggesting that you may need to lower the collision voltage.

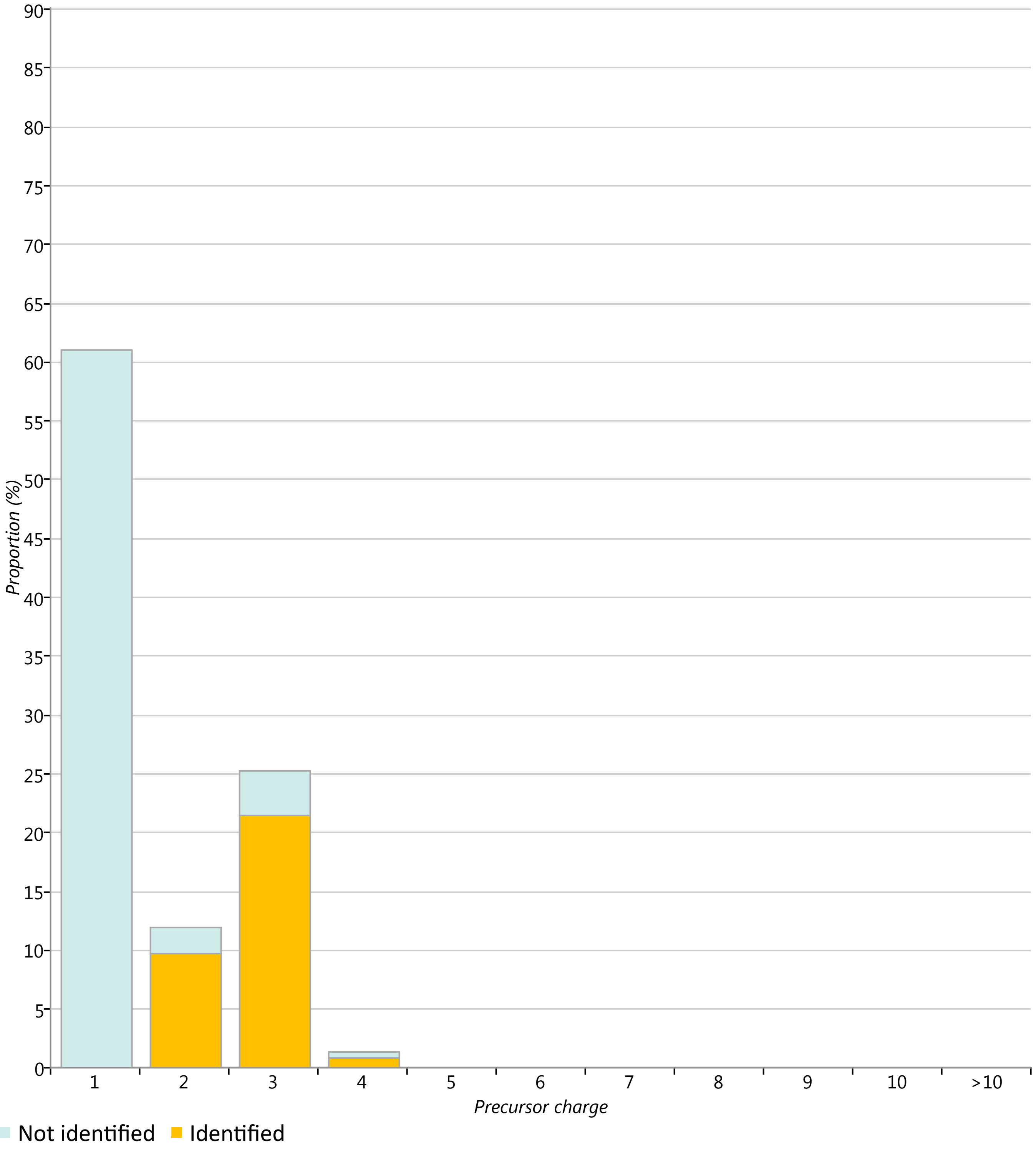

Missed cleavages (assuming trypsin)

This chart shows the distribution of missed cleavages per identified peptide ion across the whole experiment, assuming the proteins were treated with trypsin and using each peptide ion's highest scoring identification. For an alternative visualisation showing the data subdivided by experimental condition, see the *Missed cleavages* metric.

Potential problems:

- A high proportion of missed cleavages may indicate problems in trypsinization.
- An absence of missed cleavages may indicate that the database search parameters were too stringent.

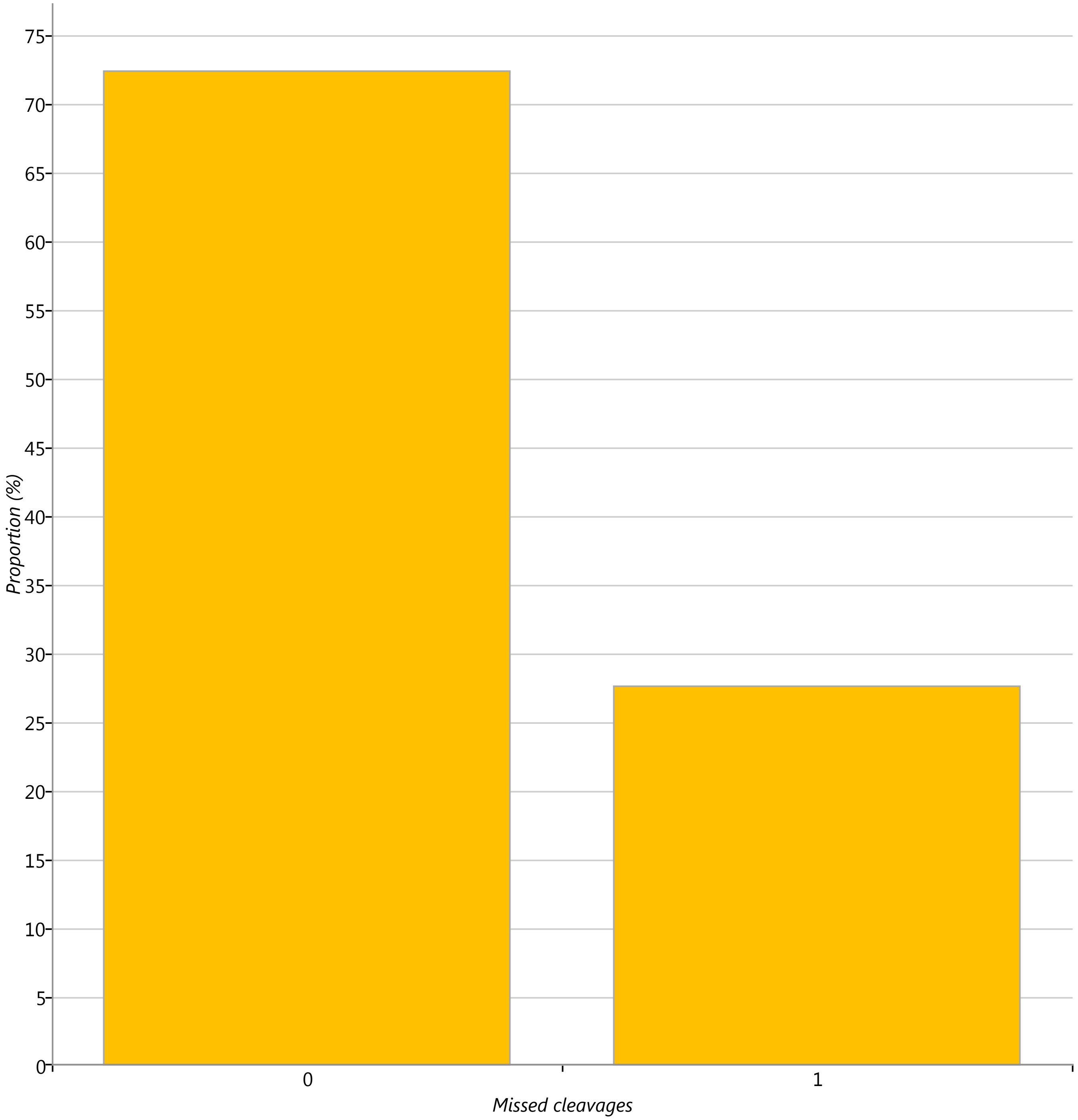

Peptides per protein (overview)

This chart shows the proportional distribution of the number of peptides identified per protein, combined across all runs in the experiment. For an alternative visualisation dividing these data by experimental condition, see the *Peptides per protein (by condition)* metric.

Potential problems:

- An unexpected bias to high or low numbers of peptides may indicate problems with sample preparation or peptide search settings.

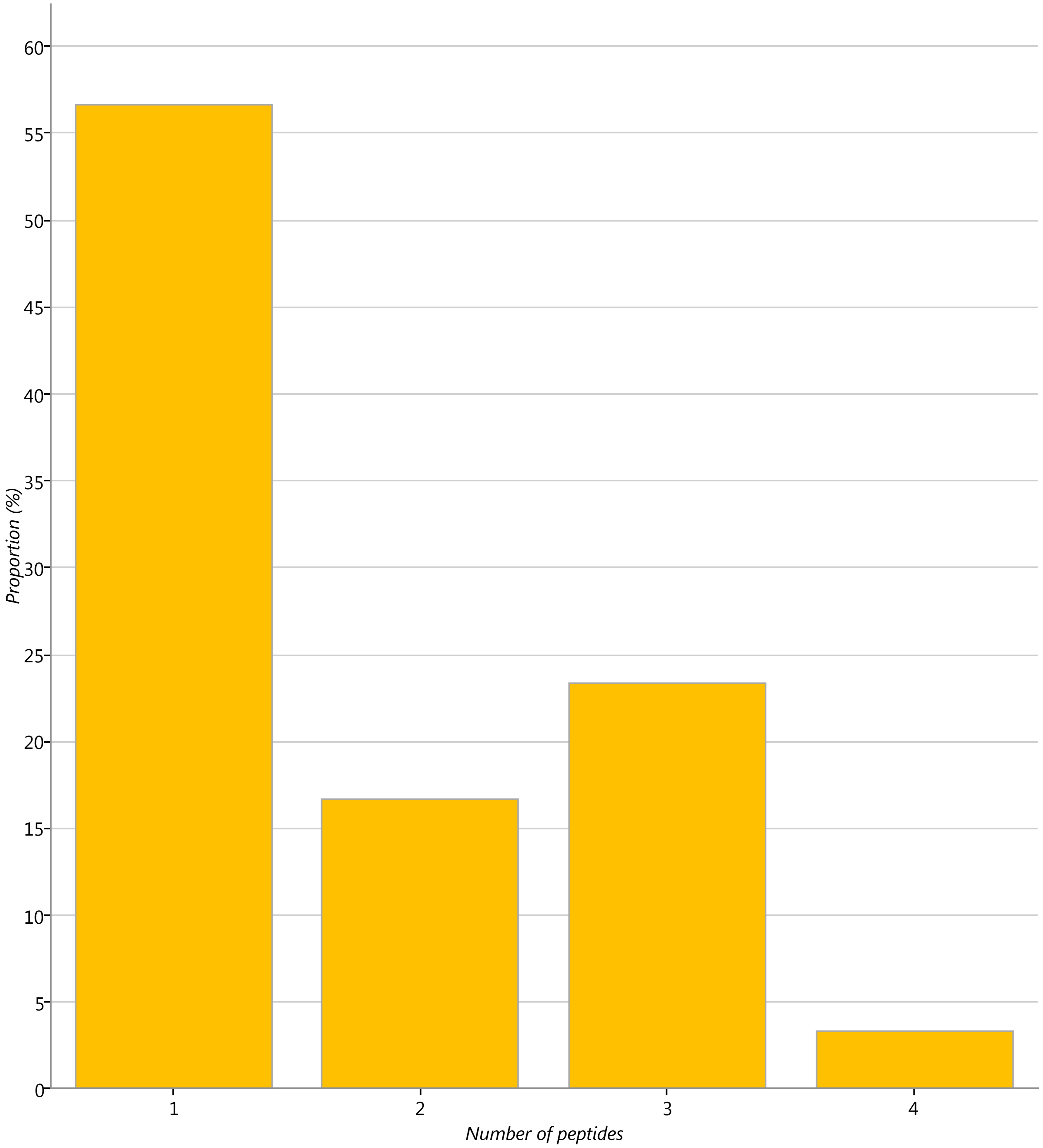

Modifications

The chart below shows the mean number of modifications per peptide ion for runs in the selected experiment design, based on each peptide ion's highest scoring identification. Each run is coloured according to its experimental condition.

Potential problems:

- Unexpectedly low numbers of modifications may indicate that one or more modifications were mistakenly not selected when searching for identifications.
- Variable numbers of modifications across the runs, especially within a given condition, may indicate problems with sample preparation.

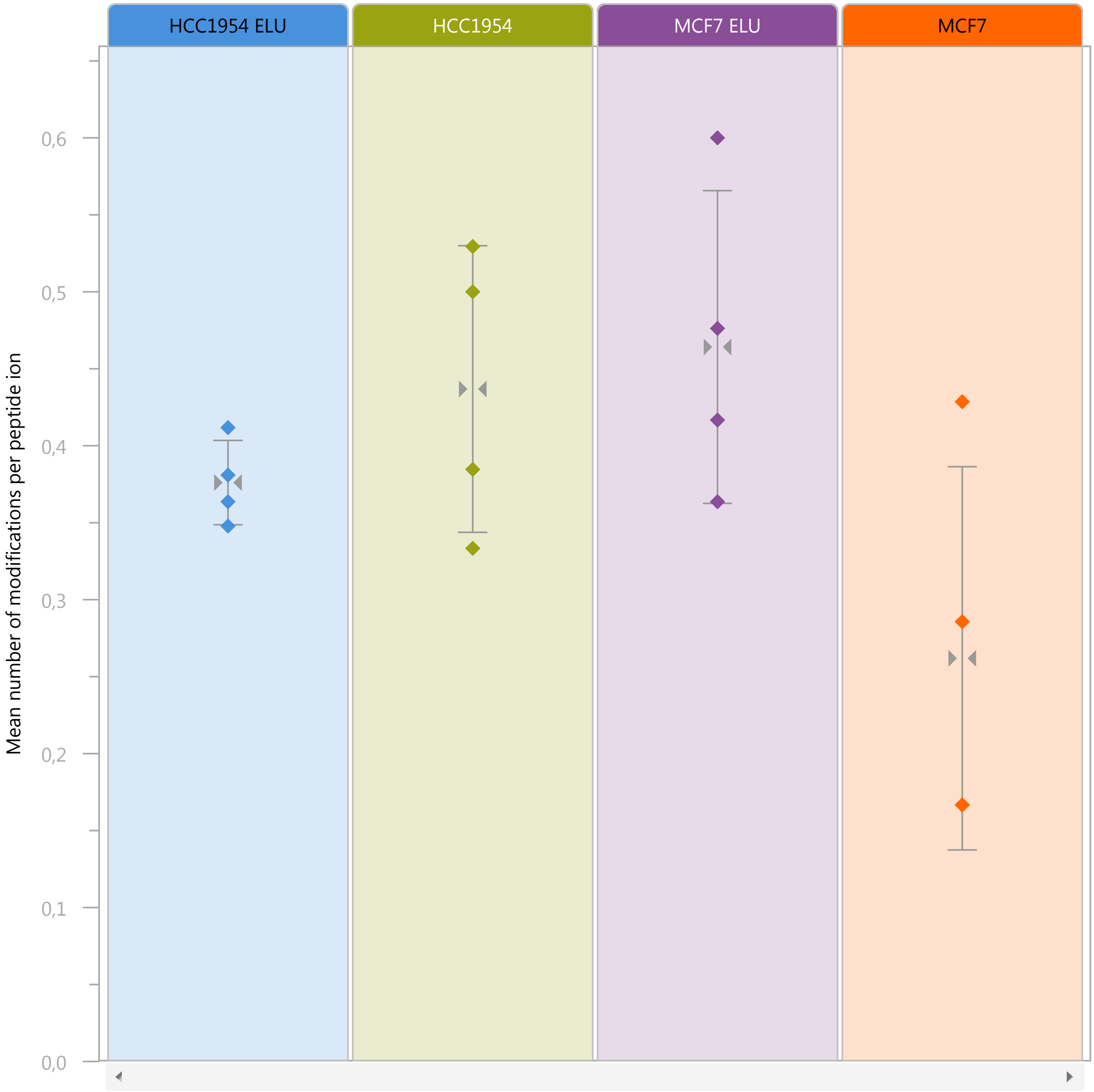

Included modifications Acetyl N-TERM Carbamidomethyl C Carbamyl N-TERM Deamidation Q Oxida

Missed cleavages

The chart below shows the mean number of missed cleavages per peptide ion for runs within the selected experiment design, based on each peptide ion's highest scoring identification. The chart assumes the use of trypsin for digestion. Each run is coloured according to its experimental condition.

Potential problems:

- A high number of missed cleavages may indicate problems in trypsinization.
- An absence of missed cleavages may indicate that database search parameters were too stringent.

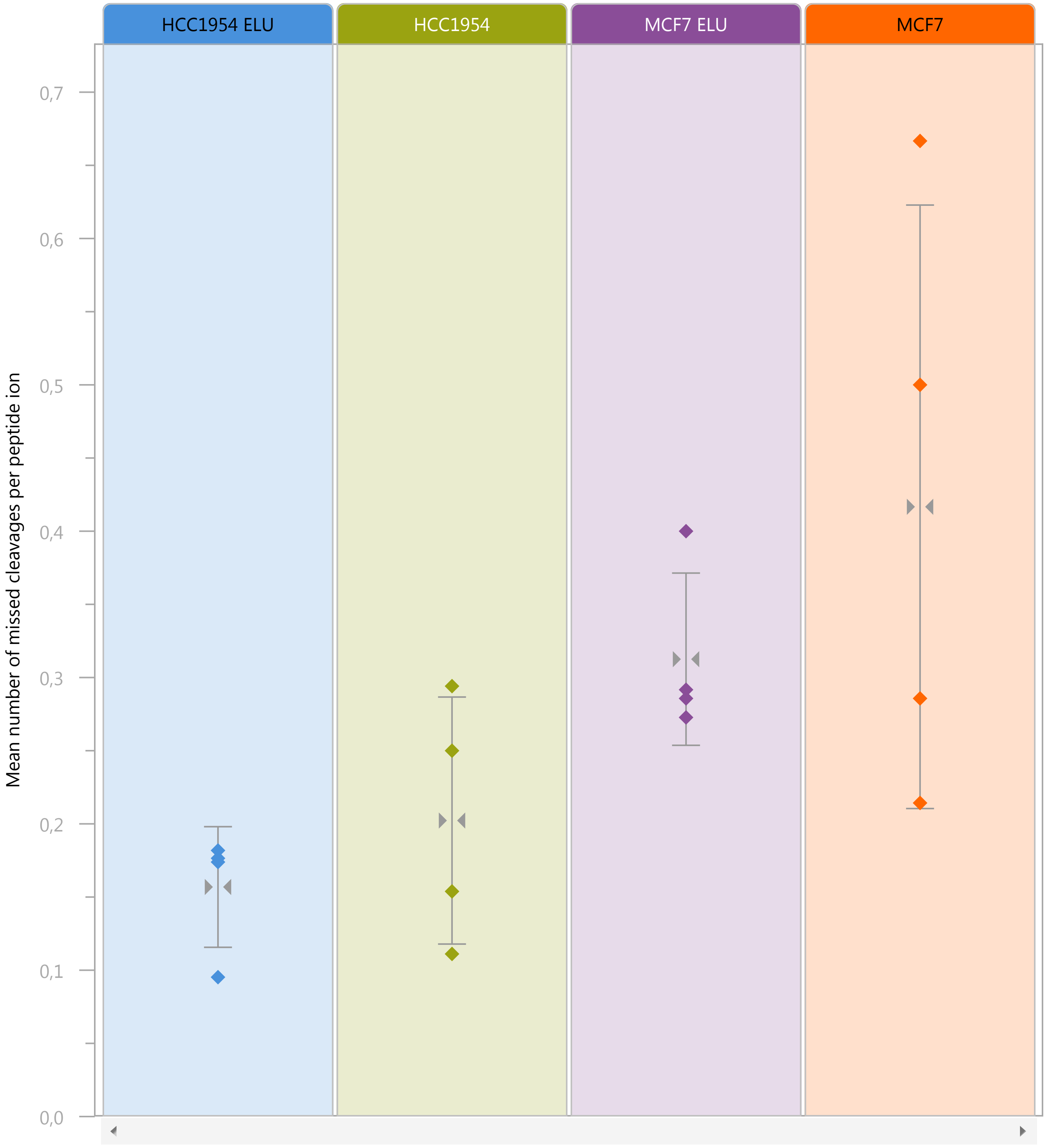

Abundance dynamic range

The charts below show abundance histograms for peptide ions, calculated using the current experiment design. Each chart shows the mean abundance for peptide ions within an experimental condition's runs. Data are binned by Log10 of their normalised abundance and shown as a proportion of the total. Identified peptide ions are shown in yellow.

Potential problems:

- A heavily right-skewed histogram of *identified* peptide ions indicates you may be missing identifications with low abundance.
- A heavily right skewed total (summed identified and unidentified) histogram may indicate saturation at high abundance, which will affect the accuracy of quantitation.

HCC1954 ELU

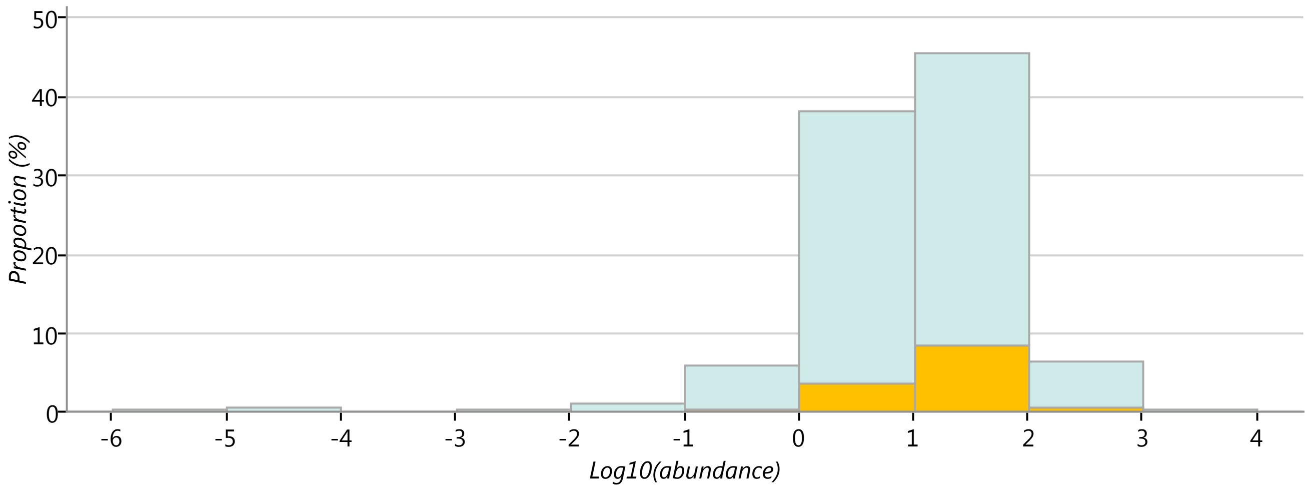

HCC1954

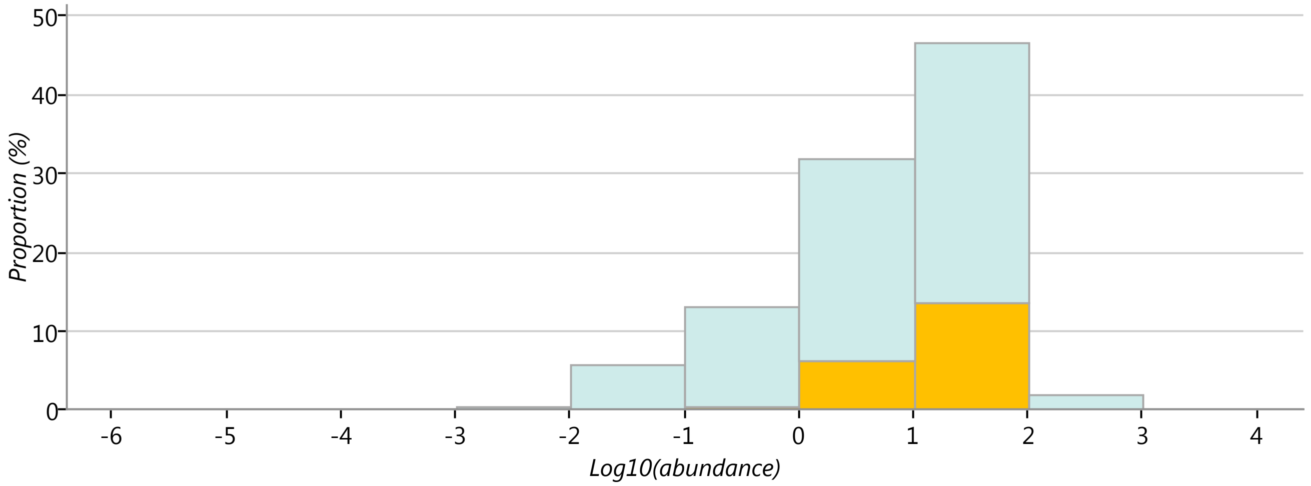

Not identified Identified

MCF7 ELU

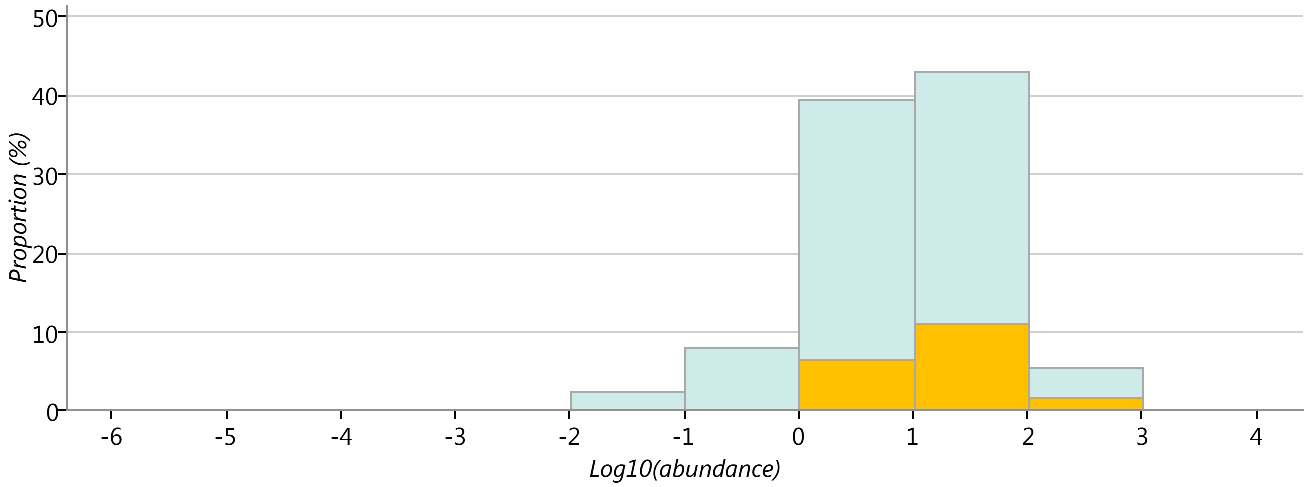

MCF7

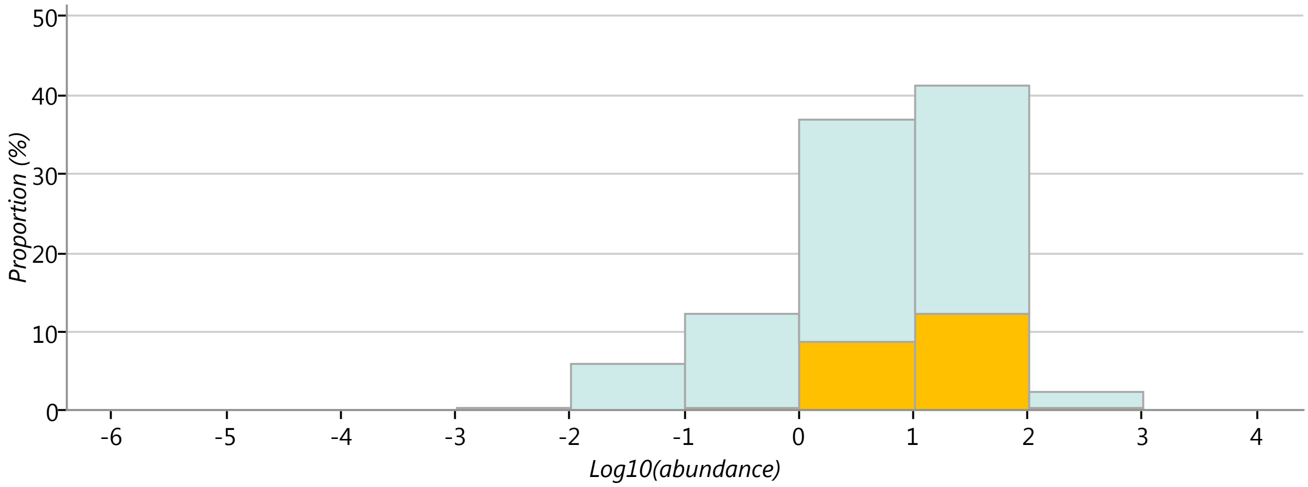

Not identified Identified

Mass accuracy

The chart below shows all identified peptide ions in your experiment, plotting peptide ion m/z against the ppm error in its highest scoring identification. Blue points indicate peptide ions which have a mass error within 2 standard deviations of the mean, yellow points indicate peptide ions with a mass error more than 2 standard deviations from the mean.

Potential problems:

- A dependence of ppm mass error on m/z may indicate poor calibration.
- If the standard deviation of the data is large in ppm terms, this may indicate that your search parameters are too lenient.

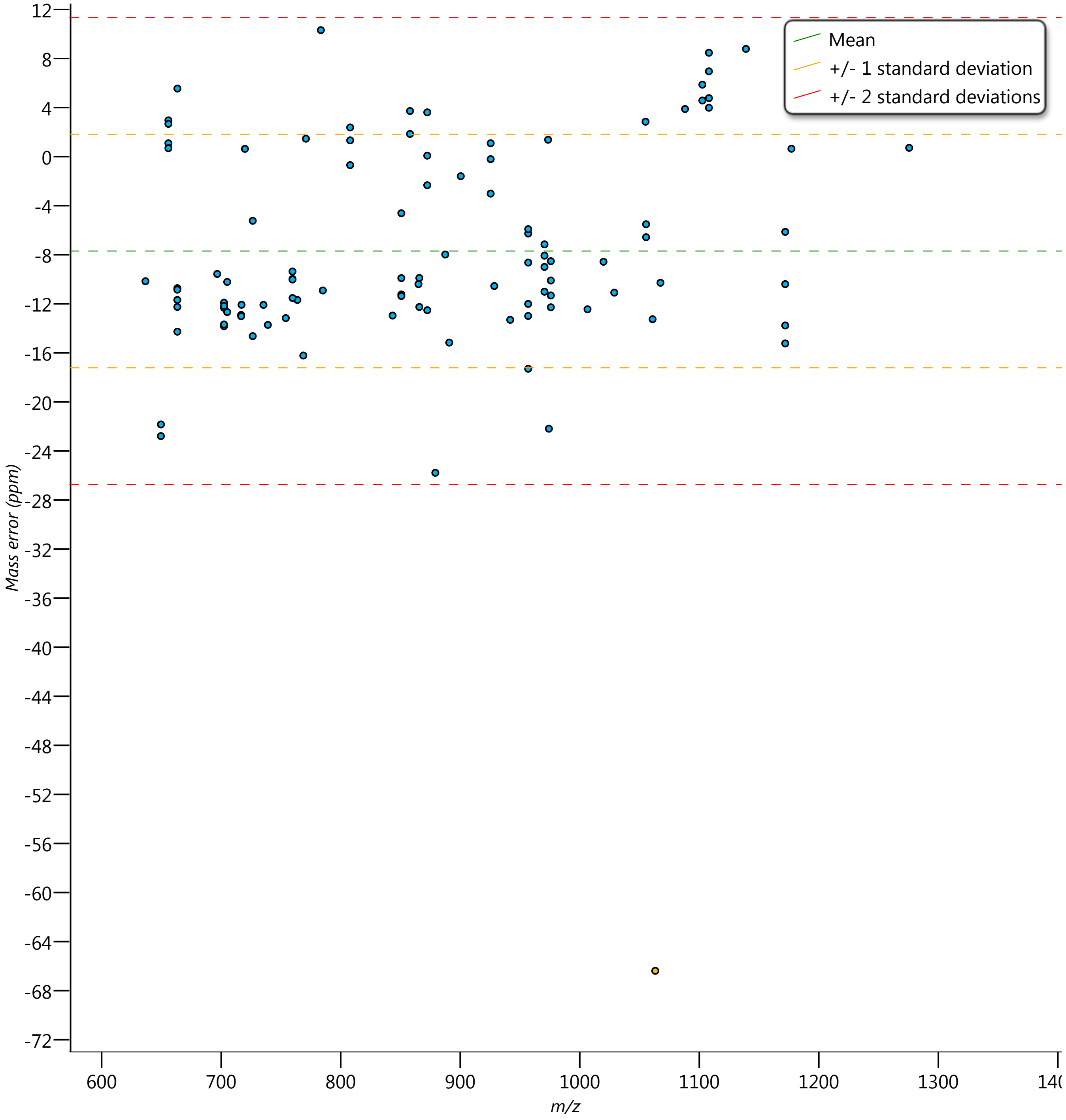

Scan rates

The charts below show the MS1 scan rate over the duration of your runs, using the current experiment design. Each chart shows the scan rate variation within an experimental condition's runs. The named run is highlighted in black. The variability between runs is shown as a grey area. The y-axis shows scans per second in a 30-second window centred on each x-axis time point.

Potential problems:

- A highly varying scan rate means quantitation accuracy may be negatively affected.

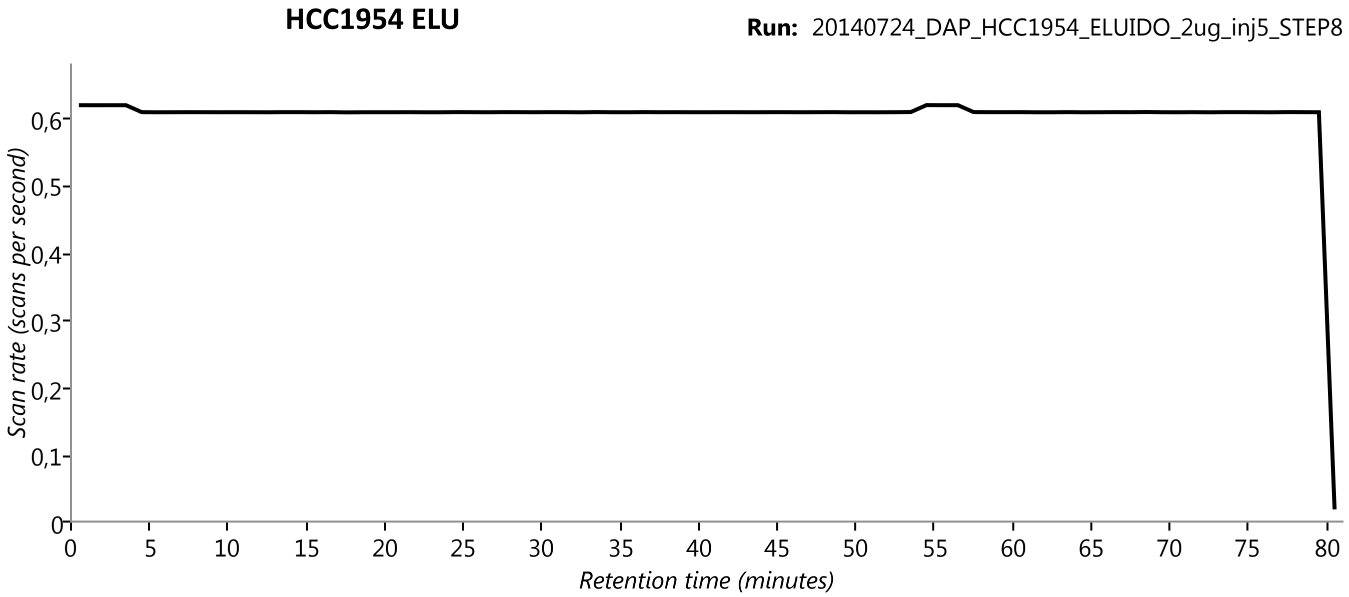

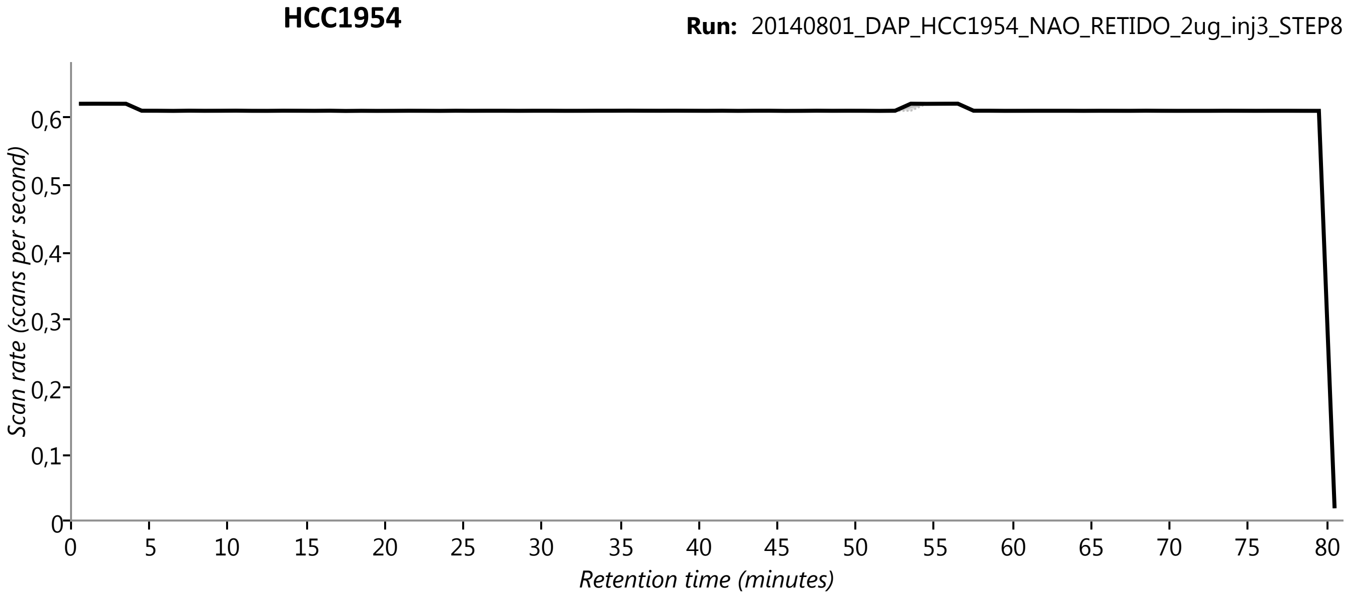

MCF7 ELU

Run: 20140724_DAP_MCF7_ELUIDO_2ug_inj5_STEP8

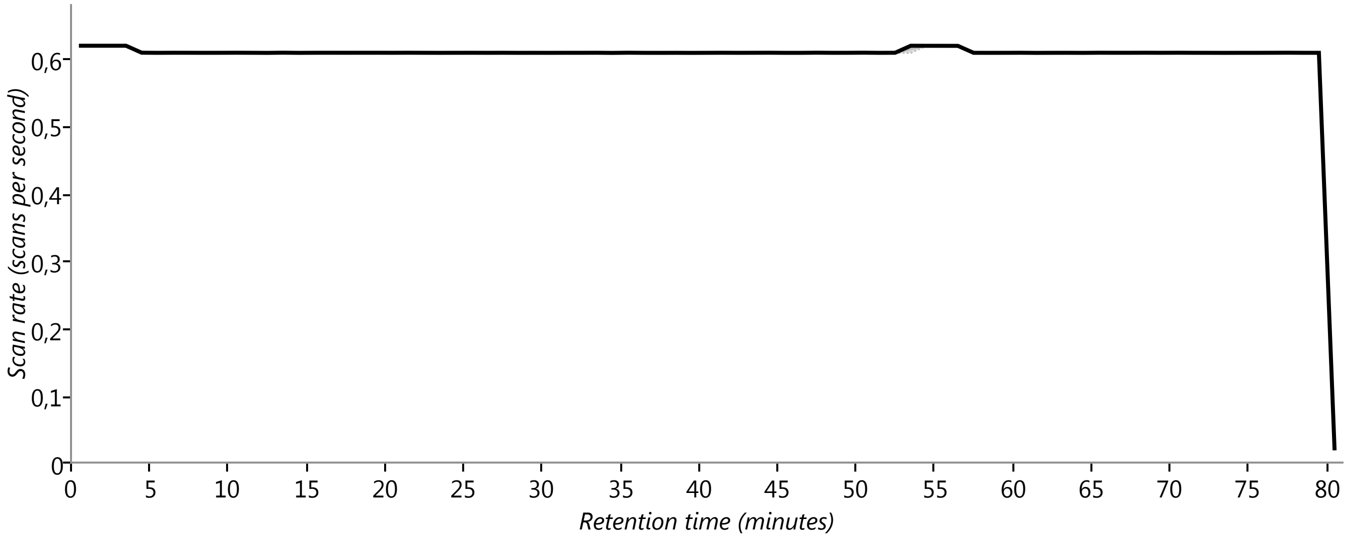

MCF7

Run: 20140801_DAP_MCF7_NAO_RETIDO_2ug_inj2_STEP8

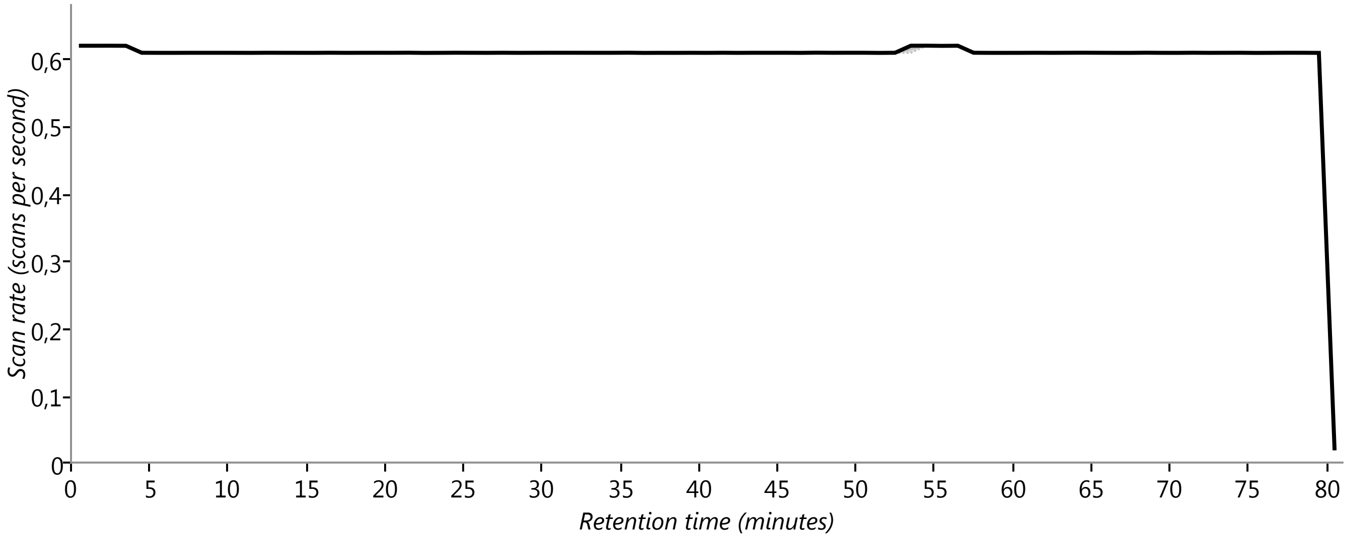

Proteins

The chart below shows the number of proteins identified in each of the runs within the selected experiment design. Each run is coloured according to its experimental condition.

Potential problems:

- Any exceptionally high or low values may indicate problems with sample preparation, chromatography, or instrument settings.

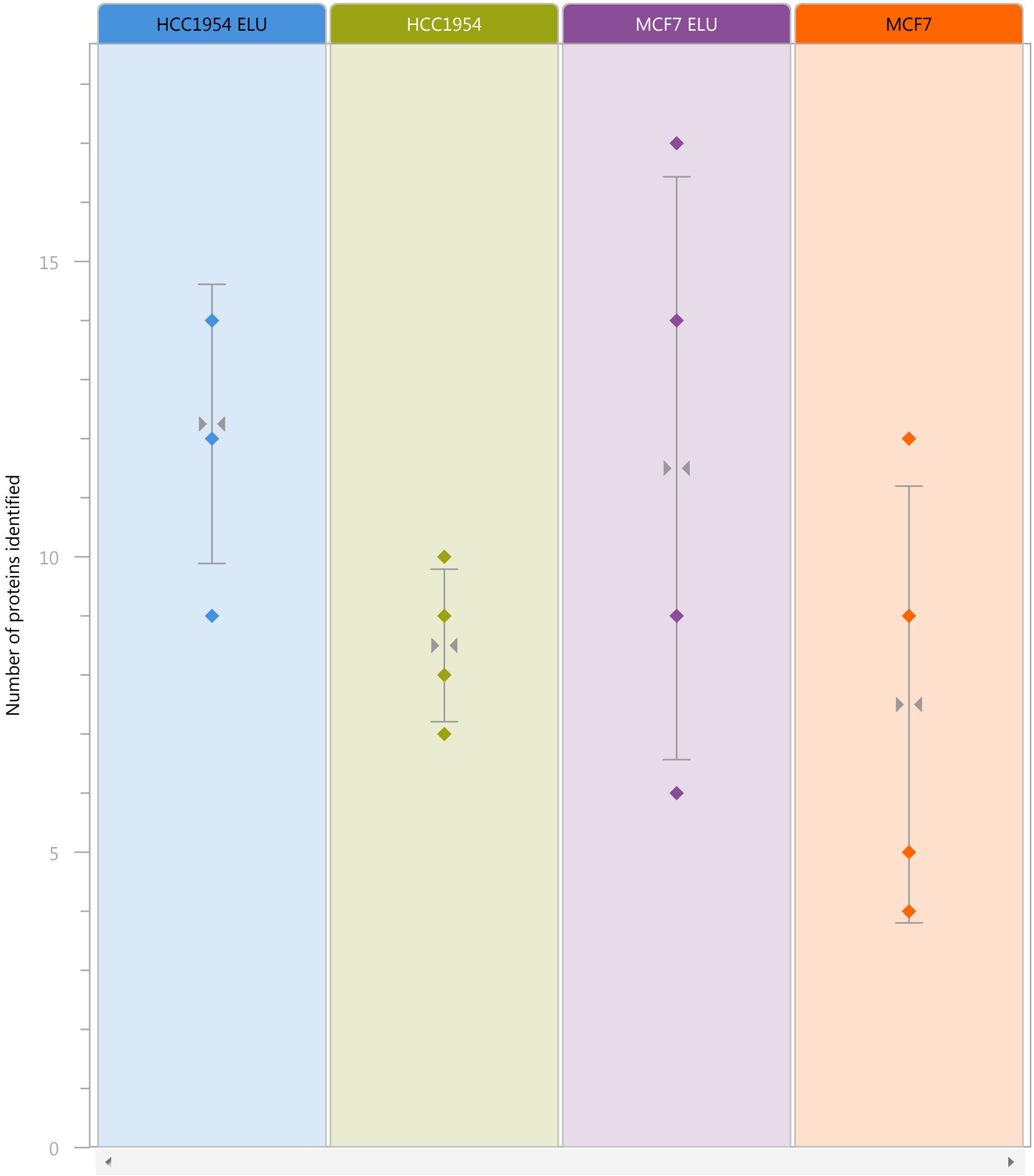

Peptides

The chart below shows the number of peptides identified in each of the experiment's runs. Each run is coloured according to its experimental condition.

Potential problems:

- Any exceptionally high or low values may indicate problems with sample preparation, chromatography, or instrument settings.

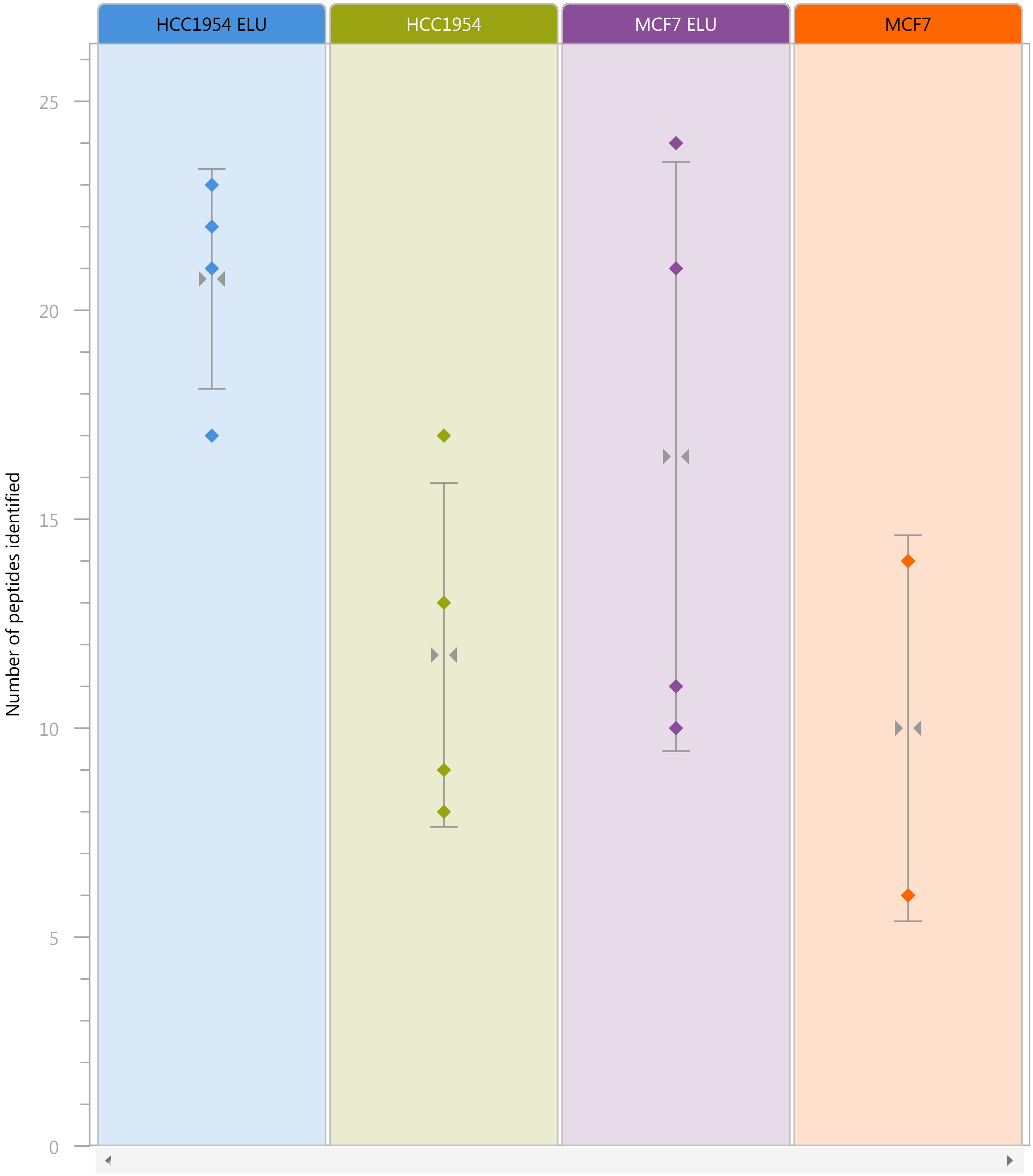

Peptides per protein (by condition)

The chart below shows the average number of peptides per protein in each run within the selected experiment design. For a given run, the value shown is calculated using only identifications from that run.

Potential problems:

- Any exceptionally high or low values may indicate problems with sample preparation or peptide search settings.

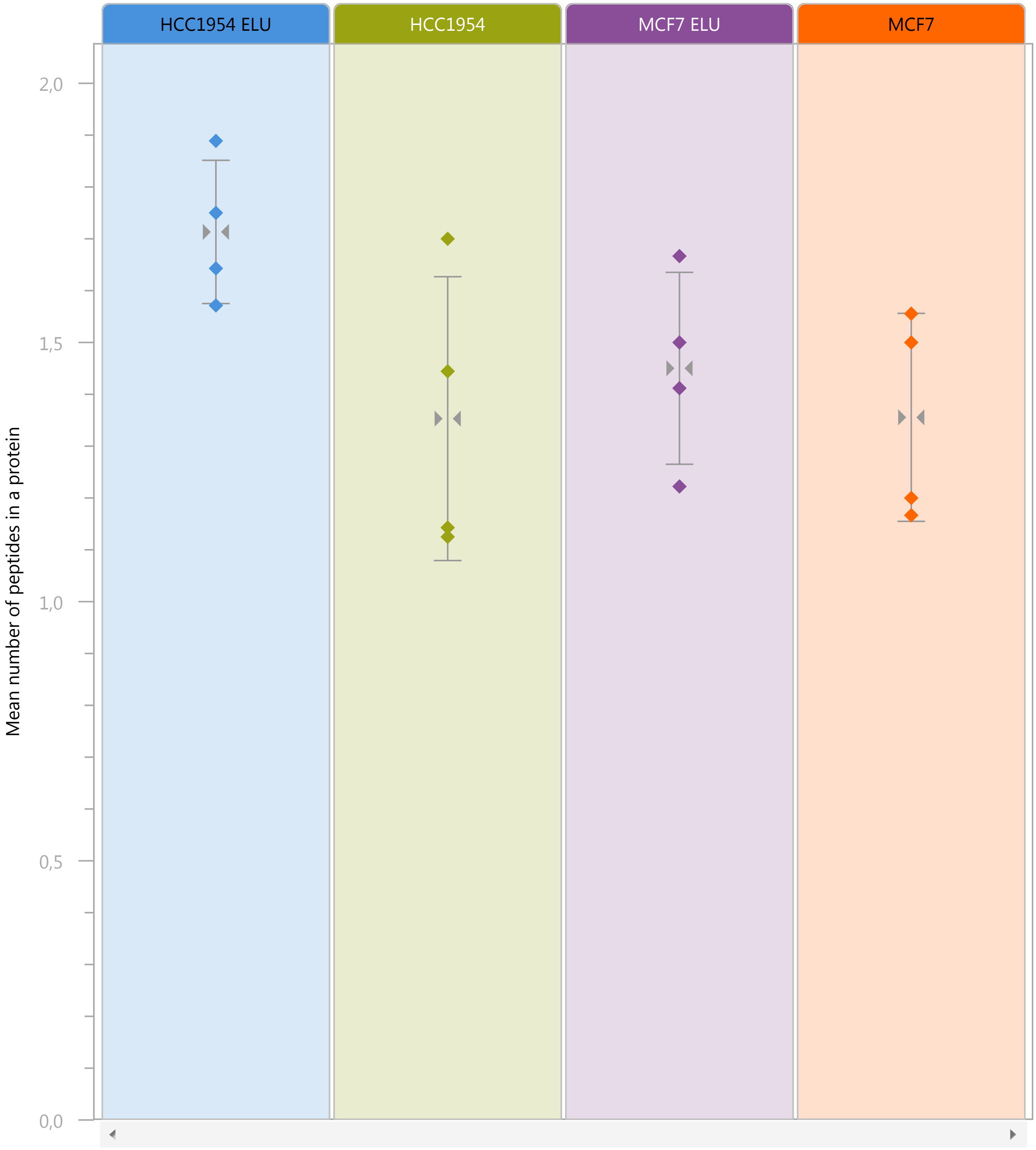

Percentage of peptide ions identified

The chart below shows the number of peptide ions identified in each of the runs within the selected experiment design, expressed as a percentage of the total number of peptide ions. Each run is coloured according to its experimental condition.

Potential problems:

- Any exceptionally high or low values may indicate problems with sample preparation, chromatography, or instrument settings.
- Very low values across all runs may indicate a problem with the database that was searched for identifications, or with other search parameters.

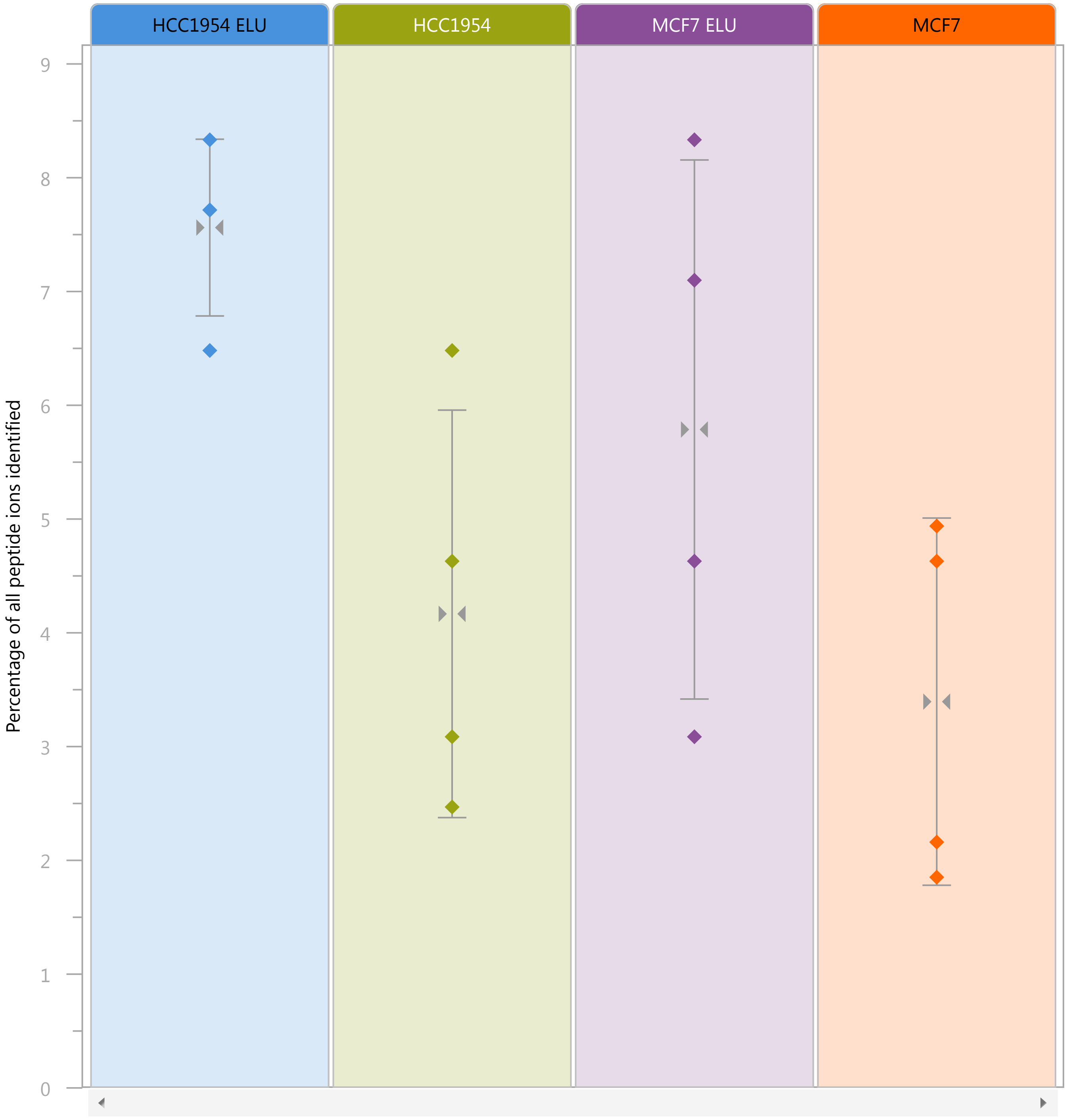

Protein overlap

The Venn diagram shows the number of proteins identified uniquely in the selected conditions and the number that are shared between conditions. Use the list on the right to select two or three conditions to explore.

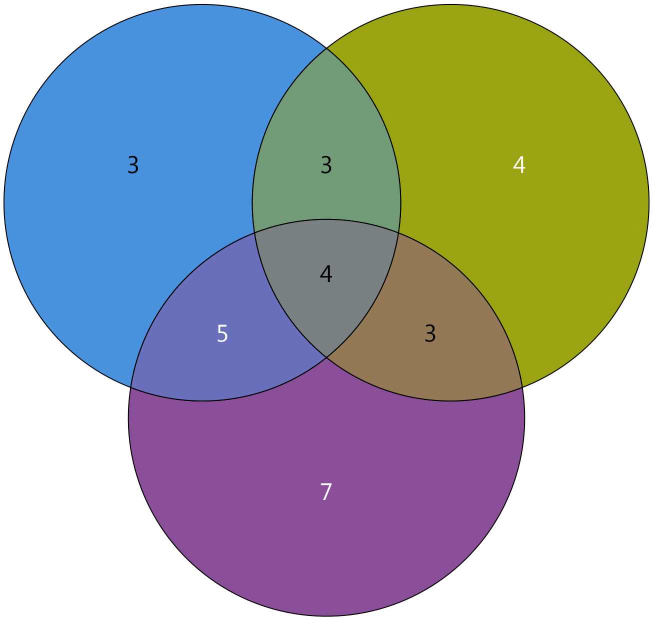

Select experimental conditions

- HCC1954 ELU
- HCC1954
- MCF7 ELU
